# Examining the impact of a community-based exercise intervention on cardiorespiratory fitness, cardiovascular health, strength, flexibility and physical activity among adults living with HIV: A three-phased intervention study

**DOI:** 10.1371/journal.pone.0257639

**Published:** 2021-09-24

**Authors:** Kelly K. O’Brien, Aileen M. Davis, Soo Chan Carusone, Lisa Avery, Ada Tang, Patricia Solomon, Rachel Aubry, Mehdi Zobeiry, Ivan Ilic, Zoran Pandovski, Ahmed M. Bayoumi

**Affiliations:** 1 Department of Physical Therapy, University of Toronto, Toronto, Ontario, Canada; 2 Institute of Health Policy, Management and Evaluation (IHPME), University of Toronto, Toronto, Ontario, Canada; 3 Rehabilitation Sciences Institute (RSI), University of Toronto, Toronto, Ontario, Canada; 4 University Health Network, Toronto, Ontario, Canada; 5 Casey House, Toronto, Ontario, Canada; 6 Avery Information, Oshawa, Ontario, Canada; 7 School of Rehabilitation Science, Institute of Applied Health Sciences, McMaster University, Hamilton, Ontario, Canada; 8 Central Toronto YMCA, Toronto, Ontario, Canada; 9 MAP Centre for Urban Health Solutions, Li Ka Shing Knowledge Institute, St. Michael’s Hospital, Toronto, Ontario, Canada; 10 Department of Medicine, Faculty of Medicine, University of Toronto, Toronto, Ontario, Canada; Prince Sattam Bin Abdulaziz University, College of Applied Medical Sciences, SAUDI ARABIA

## Abstract

**Purpose:**

Our aim was to examine the impact of a community-based exercise (CBE) intervention on cardiorespiratory fitness, cardiovascular health, strength, flexibility, and physical activity outcomes among adults living with HIV.

**Methods:**

We conducted a longitudinal intervention study with community-dwelling adults living with HIV in Toronto, Canada. We measured cardiopulmonary fitness (V̇O_2_peak (primary outcome), heart rate, blood pressure), strength (grip strength, vertical jump, back extension, push-ups, curl ups), flexibility (sit and reach test), and self-reported physical activity bimonthly across three phases. Phase 1 included baseline monitoring (8 months); Phase 2 included the CBE Intervention (6 months): participants were asked to exercise (aerobic, strength, balance and flexibility training) for 90 minutes, 3 times/week, with weekly supervised coaching at a community-based fitness centre; and Phase 3 included follow-up (8 months) where participants were expected to continue with thrice weekly exercise independently. We used segmented regression (adjusted for baseline age and sex) to assess the change in trend (slope) among phases. Our main estimates of effect were the estimated change in slope, relative to baseline values, over the 6 month CBE intervention.

**Results:**

Of the 108 participants who initiated Phase 1, 80 (74%) started and 67/80 (84%) completed the intervention and 52/67 (77%) completed the study. Most participants were males (87%), with median age of 51 years (interquartile range (IQR): 45, 59). Participants reported a median of 4 concurrent health conditions in addition to HIV (IQR: 2,7). Participants attended a median of 18/25 (72%) weekly supervised sessions. Change in V̇O_2_peak attributed to the six-month Phase 2 CBE intervention was 0.56 ml/kg/min (95% Confidence Interval (CI): -1.27, 2.39). Significant effects of the intervention were observed for systolic blood pressure (-5.18 mmHg; 95% CI: -9.66, -0.71), push-ups (2.30 additional push-ups; 95% CI: 0.69, 3.91), curl ups (2.89 additional curl ups; 95% CI: 0.61, 5.17), and sit and reach test (1.74 cm; 95% CI: 0.21, 3.28). More participants engaged in self-reported strength (p<0.001) and flexibility (p = 0.02) physical activity at the end of intervention. During Phase 3 follow-up, there was a significant reduction in trend of benefits observed during the intervention phase for systolic blood pressure (1.52 mmHg/month; 95% CI: 0.67, 2.37) and sit and reach test (-0.42 cm/month; 95% CI: -0.68, -0.16).

**Conclusion:**

Adults living with HIV who engaged in this six-month CBE intervention demonstrated inconclusive results in relation to V̇O_2_peak, and potential improvements in other outcomes of cardiovascular health, strength, flexibility and self-reported physical activity. Future research should consider features tailored to promote uptake and sustained engagement in independent exercise among adults living with HIV.

**ClinicalTrials.gov Identifier:**

NCT02794415. https://clinicaltrials.gov/ct2/show/record/NCT02794415.

## Introduction

People living with HIV with access to combination antiretroviral therapy are living longer and can experience an increased prevalence of multimorbidity associated with HIV, medications and aging [1]. Globally, the number of people living with HIV over the age of 50 years tripled between 2000 and 2020 [2]. Adults aging with HIV can experience cardiovascular disease, diabetes, bone and joint disorders, diabetes, liver disease, and non-HIV-related cancers that have implications on health and survival [3–7]. Multimorbidity can have important implications on the physical and mental health and well-being of adults aging with HIV [8].

Exercise can reduce the risk of multimorbidity and improve health outcomes for adults living with HIV [9–11]. Systematic review evidence demonstrated that engaging in aerobic, resistive or combined aerobic and resistive exercise at least three times per week can improve cardiorespiratory fitness, strength and quality of life outcomes among adults living with HIV [12–14]. However, only 51% of people living with HIV met recommended guidelines for moderate to vigorous physical activity (MVPA) of at least 150 minutes per week and people living with HIV were less active than people with other chronic illnesses [15]. Barriers to engaging in exercise for people living with HIV are multifactorial. Environmental (location, physical accessibility, cost), personal (multimorbidity, physical health, lack of knowledge or self-efficacy, anxiety), and social factors (competing priorities, caregiver responsibilities, and fear of social stigma) can prevent engagement in exercise for adults living with HIV [16–22]. Most systematic reviews did not consider these factors, and focused primarily on highly supervised therapeutic interventions by physiotherapists and exercise physiologists that may be unsustainable in community settings [12–14, 23].

Community-based exercise (CBE) involves individuals exercising under the supervision of a health or fitness instructor with the goal of promoting regular exercise in the community [24–26]. CBE can foster social interaction, support and encouragement to exercise while improving health outcomes (pain, function) among older adults, and persons with mobility issues, stroke, multiple sclerosis, and arthritis [24–30]. CBE may be an ideal self-management strategy to help people living with HIV manage health challenges associated with chronic conditions. However, the impact of CBE when translated into the community setting with adults living with HIV, and its sustainability over the long term are unknown.

Our aim was to examine the impact of a CBE intervention on cardiorespiratory fitness, cardiovascular health, strength, flexibility, and physical activity outcomes among adults living with HIV.

## Materials and methods

We conducted a single group prospective three-phased intervention study to measure the effect of a CBE intervention at the YMCA among adults with HIV in Toronto, Ontario [31, 32] from August 2016 to December 2018. Our primary outcome was cardiorespiratory fitness (peak oxygen consumption measured by V̇O_2_peak). Maximum or peak oxygen consumption (V̇O_2_peak) is a direct measure of cardiorespiratory fitness [33]. We considered 2 ml/kg/min to be a clinically important change in V̇O_2_peak, based on combination of clinical experience and interpretations of systematic review evidence examining exercise in adults living with HIV [13].

Secondary outcomes included resting heart rate (beats/min), blood pressure (systolic and diastolic; mmHg), strength (upper and lower extremity muscle strength and endurance), flexibility (hamstrings) and self-reported physical activity. These secondary outcomes were chosen because they are related to the primary outcome of interest, V̇O_2_peak, and commonly reported in previous systematic reviews on HIV and exercise [12, 13]. Outcomes were assessed every 8 weeks across three phases: 1) Phase 1 Baseline Monitoring (8 months); 2) Phase 2 CBE Intervention (6 months) and 3) Phase 3 Follow-Up (8 months), for a total of 12 time points across the 22-month study. The 8 month baseline and 8 month follow-up ensured equal timeframes prior to and after the intervention, ensured an equal number of data points in each phase (Baseline: T1–T4; Intervention: T5–T8; Follow-Up: T9-12).

The protocol was approved by the HIV/AIDS Research Ethics Board at the University of Toronto (Protocol #32910; ClinicalTrials.gov Identifier: NCT02794415). Written informed consent was received from all participants in the study. This study was part of a larger implementation science study using the RE-AIM (Reach-Evaluation-Adoption-Implementation-Maintenance) framework to investigate the process and outcomes of implementing CBE with adults living with HIV in the community [31, 34–37]. Our focus in this work is to examine the “E”, effectiveness of the intervention, as a component within the RE-AIM framework [37].

### Participants

We recruited community-dwelling adults living with HIV (18 years and older) in Toronto, Canada who considered themselves medically stable and safe to participate in exercise. We recruited through a combination of HIV community-based organizations and the Toronto YMCA using recruitment posters as well as word of mouth. Interested individuals met with a research coordinator to review eligibility, which included completing the self-administered Physical Activity Readiness Questionnaire (PAR-Q+) [38], and reviewing the information letter and consent form. We asked participants who scored ‘yes’ to one or more items on the PAR-Q+ to contact their health provider to review and confirm their eligibility to participate in the study. Participant recruitment and ongoing enrollment occurred from July 2016 to January 2017. Details on the study design and protocol have been previously published [31].

### Intervention

Phase 1 (Baseline), served as the control phase for establishing baseline trends (change over time) of outcomes. Given the nature of the exercise intervention, participants were not blinded to the intervention.

In Phase 2 (CBE Intervention), participants were provided with a YMCA membership and met weekly with a certified personal training fitness coach at the YMCA (e.g. Canada YMCA Personal Trainer Certification, CAN FIT PRO Personal Training Specialist) for 6 months. We chose a 6-month CBE duration because it aligns with transtheoretical model evidence on the stages of behaviour change whereby the ‘action’ of practicing a new behaviour (exercise) lasts up to 24 weeks followed by the ‘maintenance’ stage during which to solidify a commitment to sustaining the new behavior (self-monitored exercise) [39].

Based on an individual participant’s goals, interests and abilities, YMCA coaches established a personalized intervention that included a combination of aerobic, resistance, balance and flexibility training [40]. Participants were asked to exercise for 90 minutes, 3 times/week for 6 months. *Aerobic exercise* was prescribed 3 days/week at 60–70% peak heart rate for at least 30 minutes with type of activity based on participant choice. *Resistance exercise* involved ~8–10 exercises for major muscle groups, 3 days/week using resistance (weight) equal to 60–70% 1 repetition maximum with 10–12 repetitions each. *Balance training* included activities focused on motor skills (e.g. balance, agility, coordination and gait) for approximately 20–30 minutes, 3 days/week. *Flexibility exercise* included stretching major muscle groups in a static stretch for 10–30 seconds each with 2 repetitions, 3 days/week [41]. Participants had the option to engage in individual exercise (e.g. weight circuit training, endurance treadmill walking / jogging, stationary bike, swimming) and group-based exercise in the form of a class at the YMCA (e.g. circuit training, spinning, yoga, aerobics, dancing) [42]. Participants received in-person weekly supervised coaching at the YMCA to monitor progress and adjust exercise intensity (25 weeks). In addition, participants were invited to attend monthly group-based in-person education sessions related to self-management, HIV and health. Sessions topics included: HIV and exercise, complementary and alternative therapy, healthy eating tips, sleep health, mindfulness and stress reduction, neurocognitive health, HIV and rehabilitation.

In Phase 3 (Follow-up), participants were provided with an extension of their YMCA membership for 8 months and were encouraged to continue with thrice weekly exercise independently with no coaching supervision.

### Data collection

We examined the primary outcome of interest (V̇O_2_peak) and secondary outcomes related to the primary outcome of interest as measured by objective and self-reported physical health assessments. Participants completed a combination of fitness and questionnaire assessments every eight weeks for 8 months to obtain five baseline assessments (T1–T5 across Phase 1), followed by three assessments during the six-month intervention phase (T6–T8 Phase 2), and four assessments in the 8-month follow-up phase (T9–T12 Phase 3), for a total of 12 time points across the 22-month data collection timeframe. Data collection occurred from August 2016 to December 2018. Given the nature of the exercise intervention, assessors were not blinded to the intervention.

### Fitness assessments

Fitness assessments were conducted at the Central Toronto YMCA Performance Centre by certified fitness personnel in accordance with the Canadian Society of Exercise Physiology (CSEP) fitness assessment guidelines [43].

#### Cardiorespiratory fitness

*Peak oxygen consumption (V̇O*_*2*_*peak; ml/kg/min)* was measured at the YMCA on a stationary bike (Monark 817, Monark, Sweden) with metabolic cart for measurement of breath-to-breath gas exchange (CardioCoach 9000, KORR Medical Technologies Inc., USA) using an incremental test protocol. During the exercise test, participants started at a work rate intensity of 50–100 Watts (W) and increased their intensity by 25 Watts per minute while maintaining a target cadence of 50 revolutions per minute. YMCA staff followed the American College of Sports Medicine guidelines assessment procedure and criteria for test termination [41, 44]. Some participants may have had difficulty reaching V̇O_2_max during the assessment due to difficulty tolerating the face mask or episodes of illness affecting their ability to reach maximum effort. Hence, we referred to ‘V̇O_2_peak’ rather than ‘V̇O_2_max’ in our study.

#### Cardiovascular health

Resting Heart Rate was measured as beats per minute prior to the V̇O_2_peak assessment. Resting Blood Pressure (mmHg) was measured in the sitting position, taken at the brachial artery following the American Heart Association recommendations [45], also prior to the V̇O_2_peak assessment.

#### Strength

We measured upper and lower body strength and endurance including grip strength using a digital dynamometer (kilograms of force), lower limb power using the vertical jump test (height in cm), and muscle endurance using the back extension test (maximum seconds able to hold), push-ups (maximum number of repetitions), and partial curl ups (maximum number of repetitions up to 25 maximum) [43].

#### Flexibility

We assessed flexibility of the lower back and hamstring muscles with the sit and reach test and recorded the score to the nearest centimeter of the distance reached by the hand [46]. Strength and flexibility were assessed after V̇O_2_peak assessment.

### Questionnaire assessments

Self-reported questionnaires were completed in-person electronically at the University of Toronto.

#### Physical activity

We electronically administered the Rapid Assessment of Physical Activity (RAPA) questionnaire [47] every 8 weeks using Qualtrics software. This 9-item questionnaire is comprised of two domains that assess engagement in physical activity over the past week. RAPA 1 scores indicate level and intensity of engagement in aerobic physical activity (score range: 1–5 with higher scores indicating greater engagement). RAPA 2 scores are categorial and indicate regular engagement in exercises of strength only (score = 1), flexibility only (2), both (3) or neither (0).

#### Exercise dose

In Phase 2 and 3, participants were asked to complete a weekly exercise questionnaire from home, administered electronically using Qualtrics to document their level of engagement (dose) of exercise. We asked participants to indicate the total number of minutes they engaged in moderate to vigorous physical activity (MVPA) within the past week and total number of minutes engaged in vigorous physical activity within the past week. Questions about dosage were guided by the Canadian Physical Activity Guidelines (CPAGs), which uses number of minutes engaged in moderate to vigorous aerobic physical activity in the past week as a unit of measurement [48, 49].

#### Demographic and health characteristics

Participants completed a demographic and health questionnaire administered electronically at Phase 1 study initiation (T1) with items including but not limited to age, sex, length of time since HIV diagnosis, ethnoracial background, antiretroviral use, and number and type of concurrent health conditions.

We developed the weekly exercise, and demographic and health questionnaires as a team to include items that comprised personal, health and exercise-related characteristics. We piloted the weekly exercise questionnaire and demographic and health questionnaire prior to implementation with members of the team.

### Statistical analysis

#### Primary analyses

We compared measures of cardiorespiratory fitness, strength, flexibility, and physical activity during Phase 1 baseline (0–8 months; 5 time points (T) T1–T5), Phase 2 intervention (8–14 months; 3 time points; T6–T8) and Phase 3 post-intervention (14–22 months; 4 time points T9–T12) using linear mixed effects models.

#### Primary outcome (V̇O_2_peak)

We conducted a preliminary analysis, using only baseline data, to determine how age and sex affect V̇O_2_peak. Previous research has found that V̇O_2_max is 27% lower in females compared with males and declines about 10% per decade [50]. We found that V̇O_2_peak varied by age and sex, but there was no significant age by sex interaction, so this was not included in subsequent models.

To estimate the effect of the intervention on V̇O_2_peak we fit a segmented mixed effect linear regression model. Linear segments were fit for each phase of the study (baseline, intervention and follow-up), with random effects for the baseline intercept and slope. Age and sex were included as model covariates. An intention to treat analysis was used, whereby we analyzed all participants as receiving six months of intervention, as intended. The outcome of interest was the expected change in V̇O2peak over the six month intervention, in excess of what was observed during the baseline phase. This was calculated as 6 × (*β*_*intervention*_ − *β*_*baseline*_), where *β*_*intervention*_ and *β*_*baseline*_ represent the expected monthly change in V̇O_2_peak during the intervention and baseline periods, respectively and the difference represents the change attributable to the intervention. Following the recommendation of Peters et al. [51] missing data were not imputed. Model fit was assessed by examining residual plots and normal Q-Q plots. The complete model is detailed in S1 File. The R statistical programming language [52] was used for all analyses and modelling was performed with the nlme package [53].

#### Secondary outcomes

We used similar approaches to modelling outcomes of cardiovascular health (resting heart rate and blood pressure), strength (grip strength, vertical jump, back extension, push-ups, curl ups), flexibility (sit and reach) and physical activity (RAPA 1 aerobic score). We interpreted p<0.05 in the model to indicate significant trends (slopes) during baseline and intervention phases and to indicate whether there was a significant change in trend (slope) between the intervention (Phase 2) and follow-up (Phase 3). If baseline differences in sex were not significant at p<0.1, and if removing sex improved model fit (assessed with the Bayesian Information Criterion) then we did not adjust for sex in the model. For the categorical RAPA 2 strength and flexibility outcome we used McNemar’s χ2 test to determine if the number of people participating in strength or flexibility physical activity was different immediately prior to (T5) and after the intervention (start of follow-up phase) (T8) and after the follow-up at end of study (T12).

#### Post hoc analyses

We conducted the following additional post hoc exploratory analyses.

#### Per protocol analysis

We conducted a per protocol analysis to investigate the actual impact of intervention (including coaching sessions that were rescheduled post 25 weeks) on the outcomes of interest. For this analysis, we re-defined the baseline phase as any time prior to the first supervised training session and intervention phase as the time between the first and last supervised training session. Measurements after the last training session were not examined for this analysis (no Phase 3).

#### Exercise dose

To investigate the effect of exercise dose on outcomes, we conducted a sub group analysis with participants who recorded engaging in any exercise in their weekly web-based exercise questionnaires. To address issues of missing data across the exercise questionnaires, we decided, based on the relative stability of the exercise minutes within participants, to use the median number of minutes of moderate to vigorous physical activity and number of minutes of vigorous physical activity as proxies for treatment dose. Because it is reasonable to expect that a participant’s fitness will improve more with increasing dose, an interaction term (time in treatment phase x dose) was used to model the change in outcome over the treatment period, thereby allowing the rate of change of fitness outcomes to vary by dose. To determine the expected effect of the intervention on a participant meeting the Canadian Physical Activity Guidelines (CPAGs) [49], the estimated dose effect (per minute of exercise) was multiplied by the recommended amount in the guidelines: at least 150 moderate or vigorous minutes of physical activity in the past week (Model A); and at least 75 vigorous minutes of physical activity in the past week (Model B).

#### Sample size

Given our longitudinal design with 12 time points, to detect a level and trend change in our primary outcome, V̇O_2_peak, assuming an effect size of 1, equal preintervention and postintervention time periods, statistical significance p<0.05, and an autoregression error time series model with lag 1, and autocorrelation estimate 0.3, we would expect a power of 0.80 [54–56]. We aimed to recruit 120 participants, with the goal for 75 participants to complete the study, based on our observed retention rate of approximately 60% in a pilot study. More details on our sample size estimation can be found in our protocol [31].

## Results

**Fig 1:**
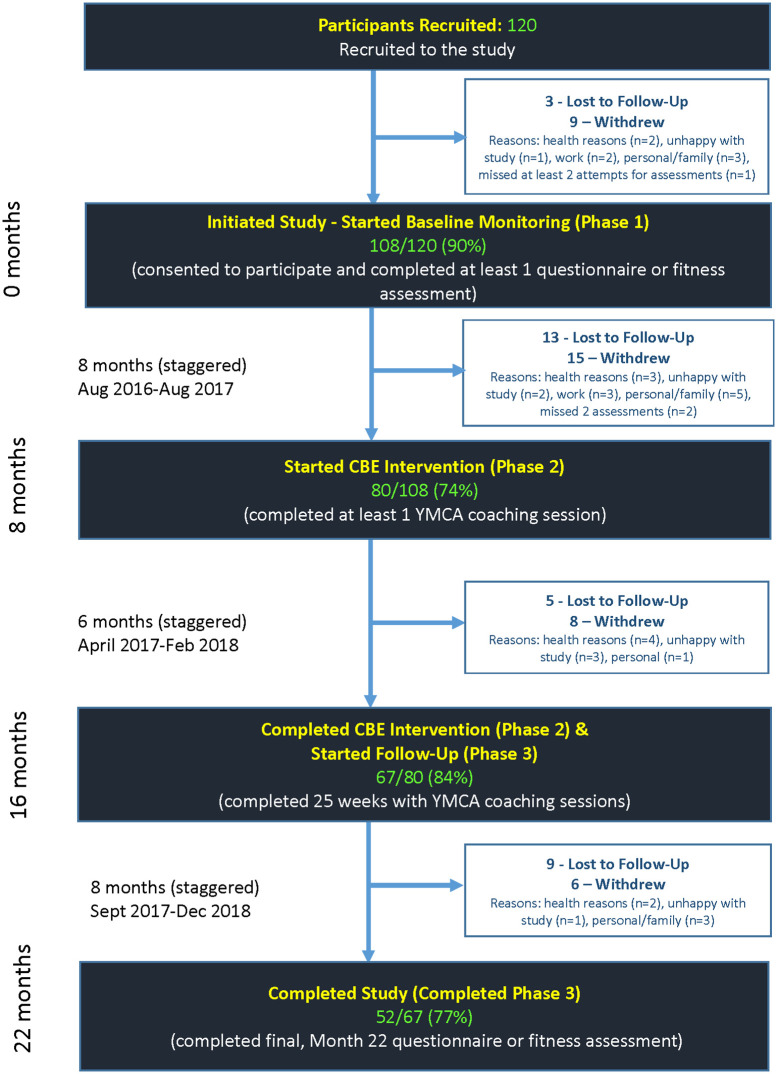
Participant flow chart—Phase 1 (Baseline), Phase 2 (Intervention) and Phase 3 (Follow-up).

Of the 120 individuals recruited to the study, 108 provided consent and initiated the baseline monitoring phase; of these, 80 (74%) started and 67/80 (84%) completed the intervention; and 52/67 (77%) completed the study (Fig 1).

Among the 108 participants at study initiation, most were males (89%), the median age was 51 years (males: 51 years, Interquartile Range (IQR) 45, 59; females: 48 years, IQR: 43, 61), 37% were from racialized communities, and the median number of concurrent health conditions in addition to HIV was 5 (IQR: 2,7). Participants self-reported engaging in a median of 196 minutes (IQR: 120, 306) of moderate or vigorous physical activity in the past week during Phase 2 (intervention) and Phase 3 (follow-up) (Table 1). The median duration between Phase 2 assessments (T5 to T8) was 189 days (27 weeks). Participants who started the intervention attended a median of 18/25 (72%) of the weekly supervised coaching sessions. Thirty of 80 participants who started the intervention, rescheduled at least one of their coaching sessions beyond the 25 week intervention phase due to episodes of illness, travel or work commitments. There were no serious adverse events documented from the intervention in the study. Baseline demographic characteristics of the sample in the study at enrollment, start of intervention, end of intervention, and end of study are detailed in S1 Table.

**Table 1:** Characteristics of participants.

**Characteristics at Study Initiation (Phase 1)**	**Number (%)**
**Median age (IQR)** (n = 91)	51 years (45, 59)
**≥ 50 years**	53 (49%)
**Sex**	
Male	96 (89%)
Female	12 (11%)
**Comorbidities**	
Median number of comorbidities^a^ (IQR)	5 (2,7)
Living with ≥2 comorbidities^a^	89 (82%)
**Most commonly self-reported co-morbidities (>30%) included:**	
Mental health (e.g. depression, anxiety)	52 (48%)
Joint pain (e.g. arthritis)	44 (41%)
Muscle pain	39 (36%)
Bone and joint disorder (osteopenia, osteoporosis, osteoarthritis)	39 (36%)
**Median number of years since HIV diagnosis (25-75** ^**th**^ **percentile)**	17 (8, 27)
**Current antiretroviral (HIV medication) use**	107 (99%)
**Self-reported viral load undetectable** (<50 copies/mL) (n = 94)	90 (83%)
**Self-reported current health status**	
Excellent	8 (7%)
Very good or good	80 (74%)
Fair	18 (17%)
Poor	2 (2%)
**Health status compared to previous year**	
Better now than 1 year ago	50 (46%)
About the same as 1 year ago	42 (39%)
Worse than 1 year ago	16 (15%)
**Employed Full-Time or Part-Time**	34 (31%)
**Smoking History** (n = 104)	
I currently smoke regularly or occasionally	32 (30%)
I am a former smoker	29 (30%)
I have never been a smoker	40 (37%)
**Have Children**	16 (15%)
**Live Alone**	73 (68%)
**Gross average yearly income–CAD** (n = 107)	
≤$20,000	57 (53%)
**Highest level of education** (n = 107)	
Completed high School/secondary school	6 (6%)
Completed trade/technical school	3 (3%)
Completed college	24 (22%)
Completed university	21 (20%)
Post-graduate education	19 (18%)
**Exercise History (at start of exercise intervention)** (n = 83)	
“I currently exercise regularly and have done so for >6 months”	30 (28%)
**Outcomes of Interest at Study Initiation** ^b^	**Mean (SD)**
**V̇O**_**2**_**peak (ml/kg/min)** (n = 100)	
Males (n = 90)	24.24 (±8.04)
Females (n = 10)	16.70 (±4.14)
**Resting Heart Rate (beats per minute)** (n = 102)	76.42 (±16.59)
**Systolic Blood Pressure (mmHg)**	
Males and Females	120.58 (±17.58)
**Diastolic Blood Pressure (mmHg)**	
Males (n = 91)	76.55 (±11.83)
Females (n = 11)	81.27 (±13.71)
**Grip Strength (kg)**	
Male (n = 91)	78.47 (±17.74)
Female (n = 10)	54.73 (±7.46)
**Vertical Jump Test (cm)**	
Male (n = 86)	23.37 (±9.07)
Female (n = 10)	15.55 (±7.31)
**Back Extension (sec)**	
Male (n = 87)	67.06 (±43.05)
Female (n = 10)	58.1 (28.06)
**Push Ups (number completed)**	
Male (n = 90)	10.67 (±9.11)
Female (n = 10)	8.50 (±6.47)
**Curl Ups (number completed up to 25 maximum)**	
Male (n = 88)	15.98 (±10.27)
Female (n = 10)	12.00 (±12.74)
**Flexibility—Sit and Reach Test (cm)** (n = 99)	22.43 (±11.26)
**RAPA 1 –Aerobic physical activity (Range 1–5)**	
Male (n = 94)	4.40 (±0.79)
Female (n = 11)	3.91 (±0.70)
**RAPA 2 –Strength and Flexibility physical activity** (n = 108)	n (%)
0 = None	33 (31%)
1 = Strength Only	11 (10%)
2 = Flexibility Only	35 (32%)
3 = Both Strength and Flexibility	29 (27%)
**Self-Reported Physical Activity in Weekly Questionnaires** ^c^	Median (IQR)
Minutes of Moderate or Vigorous Physical Activity in past week (Phase 2 and 3)	196 (120, 306)

Sample sizes: n = 108 (at enrollment) unless otherwise indicated; some sample sizes may not add up to total sample due to missingness on demographic and health questionnaire items; IQR: interquartile range;

^a^excluding HIV;

^b^reflects first measurement for each participant in the study; Outcomes of interest were reported separately for males and females if they differed and were adjusted for in the model.

^c^n = 45 participants who completed weekly web-based self-reported exercise questionnaires during Phase 2 (intervention) and Phase 3 (follow-up).

### Primary outcome

#### V̇O_2_peak

**Fig 2:**
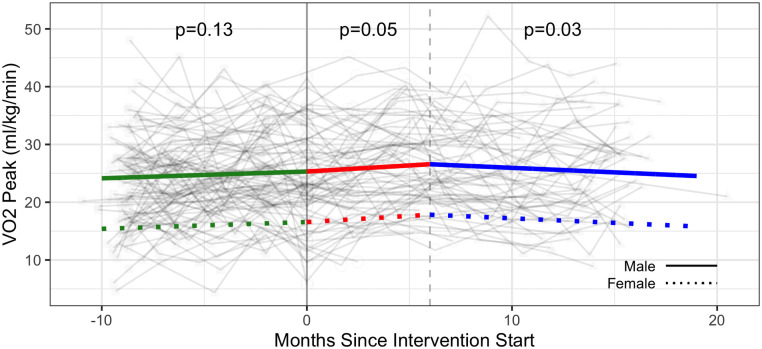
Primary outcome—V̇O_2_peak (ml/kg/min) trajectories over phase 1 (baseline), phase 2 (intervention) and phase 3 (follow-up monitoring); (n = 100 participants). Vertical dotted line indicates end of phase 2 (intention to treat) at 6 months.

One hundred participants completed at least one V̇O_2_peak assessment and were included in the primary (V̇O_2_peak) analysis (90 males, 10 females) with a total of 689 observations across the three phases. Baseline mean (sd) V̇O_2_peak was 24.2 (8.0) ml/kg/min for males (n = 90) and 16.7 (4.1) ml/kg/min for females (n = 10). Results for the linear mixed effect models of V̇O_2_peak over Phase 1 (baseline), Phase 2 (intervention) and Phase 3 (follow-up) are presented in Fig 2 and Table 2.

**Table 2:** Results—Primary outcome—Cardiorespiratory fitness—V̇O_2_peak.

**V̇O**_**2**_ **peak (ml/kg/min)**				
**Estimated Intervention Effect over 6 month Intervention Phase:**				
0.56 ml/kg/min (95% CI: -1.27, 2.39)				
Number of Observations: 689; Sample size: 100 (90 males; 10 females)				
**Parameter**	**Fixed Effects**			**Random Effects (SD)**
	**Estimate**	**95% Confidence Interval**	**p-value**	
Intercept (50yr old male)	25.311	23.900, 26.721	<0.001	5.67
Age Effect	-0.138	-0.248, -0.02	0.014	--
Sex Effect	-8.741	-12.953, -4.529	<0.001	--
Phase 1: Baseline slope (change per month)	0.118	-0.035, 0.270	0.130	0.40
Phase 2: Intervention slope (change per month)	0.211	0.004, 0.418	0.046^b^	0.26
Phase 3: Difference in Follow-up (Phase 3) and Intervention (Phase 2) slope^a^ (change per month)	-0.368	-0.704, -0.032	0.032	<0.01
Residual	--	--	--	4.15

Slopes are change in outcome over one month.

^a^ Estimate refers to the difference in slope between the follow-up and intervention phase; Phase 3 (Follow-Up) slope is the sum of Phase 2 (intervention) slope and Phase 3 (difference in Follow-Up and Intervention slope): (0.211) + (-0.368) = -0.16 ml/kg/min.

^b^Significant trend in Phase 2 (intervention) slope versus 0; *Estimated intervention effect over intervention*: (0.211–0.118)*6 months = 0.56 ml/kg/min; *Interpretation*: Fixed effects indicate the average population level effect while random effects indicate the expected variability across people.

For instance, average V̇O_2_peak value was 25.3 ml/kg/min at the beginning of the intervention with a 95% confidence interval of (23.9, 26.7), which refers to our level of confidence in the population average. The estimated random effect was 5.7 ml/kg/min, which indicates the variability in V̇O_2_peak values in the sample population.

The estimated effect of the CBE intervention on V̇O_2_peak over the six-month intervention (Phase 2) taking the baseline (Phase 1) into account was 0.56 ml/kg/min (95% CI: -1.27, 2.39), with considerable variation of V̇O_2_peak within individuals over time (Table 2 and Fig 2). For the monthly rate of change (slope); during Phase 1, there was a small improvement in V̇O_2_peak (0.12ml/kg/min/month; 95% CI: -0.04, 0.27) followed by an increase in Phase 2 (0.21 ml/kg/min/month; 95% CI: 0.00, 0.42) and a small decline in Phase 3 (-0.16 ml/kg/min/month; 95% CI: -0.35, 0.03 [-0.16 ml/kg/min = 0.21 (Phase 2 slope) + (-0.37) (Phase 3 slope); Table 2]) (Table 2). The Phase 2 slope was not significantly different to the Phase 1 slope (p = 0.19) indicating the change in V̇O_2_peak during the intervention phase was not significantly different to the change during baseline (not shown). Individual trajectory plots of V̇O_2_peak for each participant are detailed in S1 Fig.

### Secondary outcomes

Table 3 summarizes the estimated effect of the CBE intervention (Phase 2) over and above any changes in baseline (Phase 1) for secondary outcomes. Results for secondary outcomes comparing Phase 1 (baseline), Phase 2 (intervention) and Phase 3 (follow-up) are detailed in S2 Table.

**Table 3:** Secondary outcomes—Overall estimated effect of CBE intervention—Intention to treat analysis—Estimated effects of the six-month community-based exercise (CBE) intervention (Phase 2) after taking the eight-month baseline monitoring phase (Phase 1) into account.

Secondary Outcomes	Sample Size (n)	Number of Observations	Overall Effect Estimate (95% CI)
**Cardiorespiratory Fitness**			
Resting heart rate (bpm)	102	718	-0.75 (-3.97, 2.48)
Resting diastolic blood pressure (mmHg)	102	724	-0.68 (-3.78, 2.43)
Resting systolic blood pressure (mmHg)	102	724	-5.18 (-9.66, -0.71)^a^
**Strength and Flexibility**			
Upper extremity–Grip strength (kg)	101	721	-1.41 (-3.62, 0.81)
Lower extremity–Vertical jump test (cm)	96	656	-3.06 (-4.57, -1.54)
Back extension (seconds)	97	667	-6.90 (-16.32, 2.52)
Push ups (number completed)	100	695	2.30 (0.69, 3.91)^a^
Curl ups (number completed)	98	688	2.89 (0.61, 5.17)^a^
Flexibility–sit and reach test (cm)	99	698	1.74 (0.21, 3.28)^a^
**Self-Reported Physical Activity**			
RAPA Aerobic scale	105	805	0.16 (-0.03, 0.35)

^a^ Significant overall estimated treatment effect over the six-month intervention after taking the baseline monitoring into account;

V̇O_2_peak: peak oxygen consumption; RAPA: Rapid Assessment of Physical Activity; RAPA Aerobic (range: 1–5); CI: Confidence Interval.

#### Cardiovascular health

**Fig 3:**
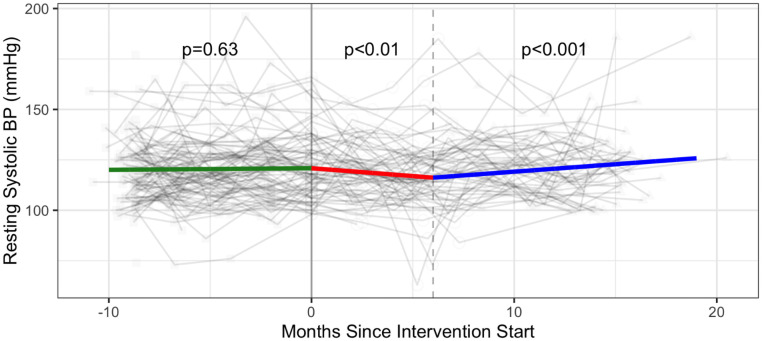
Cardiovascular health—Systolic blood pressure (mmHg) trajectories over phase 1 (baseline), phase 2 (intervention) and phase 3 (follow-up monitoring); (n = 102 participants). Vertical dotted line indicates end of phase 2 (intention to treat) at 6 months; BP: blood pressure.

The change in systolic blood pressure attributable to the intervention over 6 months was a decrease of 5.18 mmHg (95% CI: -9.66, -0.71); the monthly change during Phase 2 was -0.78 mmHg (p<0.01) and during Phase 3 was 0.74 mmHg (p<0.001; comparison with Phase 2) (Fig 3 and S2 Table).

**Fig 4:**
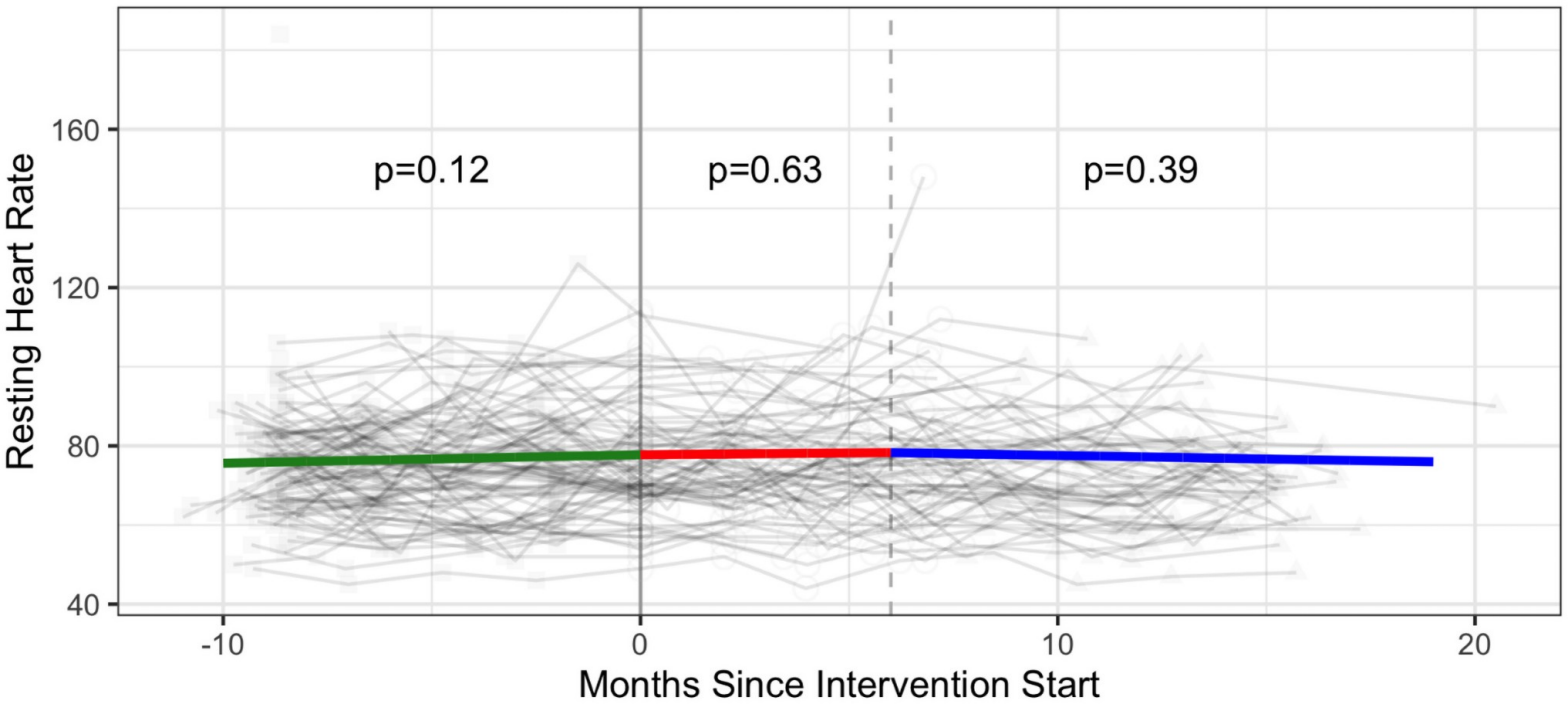
Cardiovascular health—Resting heart rate (beats per minute) trajectories over phase 1 (baseline), phase 2 (intervention) and phase 3 (follow-up monitoring); (n = 102 participants). Vertical dotted line indicates end of phase 2 (intention to treat) at 6 months.

**Fig 5:**
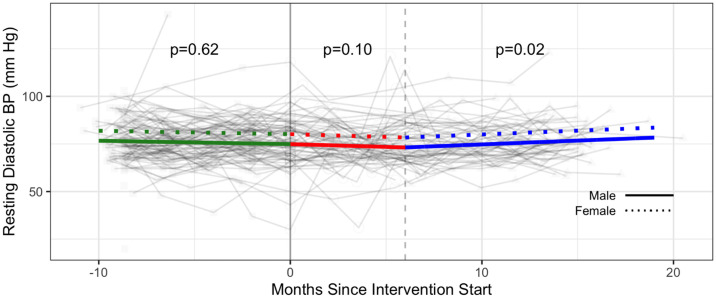
Cardiovascular health—Diastolic blood pressure (mmHg) trajectories over phase 1 (baseline), phase 2 (intervention) and phase 3 (follow-up monitoring); (n = 102 participants). Vertical dotted line indicates end of phase 2 (intention to treat) at 6 months; BP: blood pressure.

Estimated intervention effects were not statistically significant for resting heart rate and diastolic blood pressure. There was no statistically significant monthly change in resting heart rate across all three phases, and a statistically significant monthly increase of 0.40 mmHg in diastolic blood pressure in Phase 3 (p = 0.02 comparison with Phase 2 (Figs 4 and 5 and S2 Table).

#### Strength & flexibility

**Fig 6:**
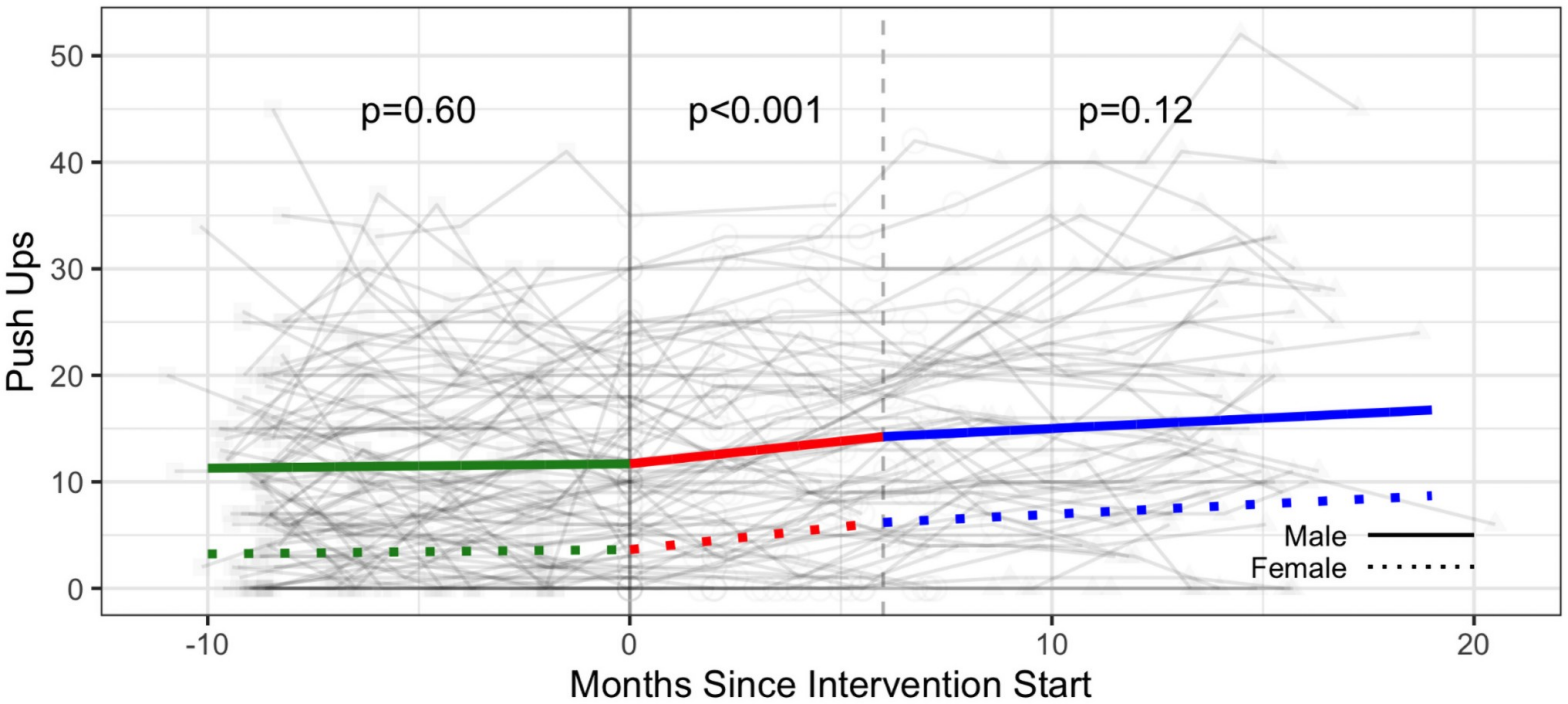
Strength—Push ups (number of repetitions) trajectories over phase 1 (baseline), phase 2 (intervention) and phase 3 (follow-up monitoring); (n = 100 participants). Vertical dotted line indicates end of phase 2 (intention to treat) at 6 months.

**Fig 7:**
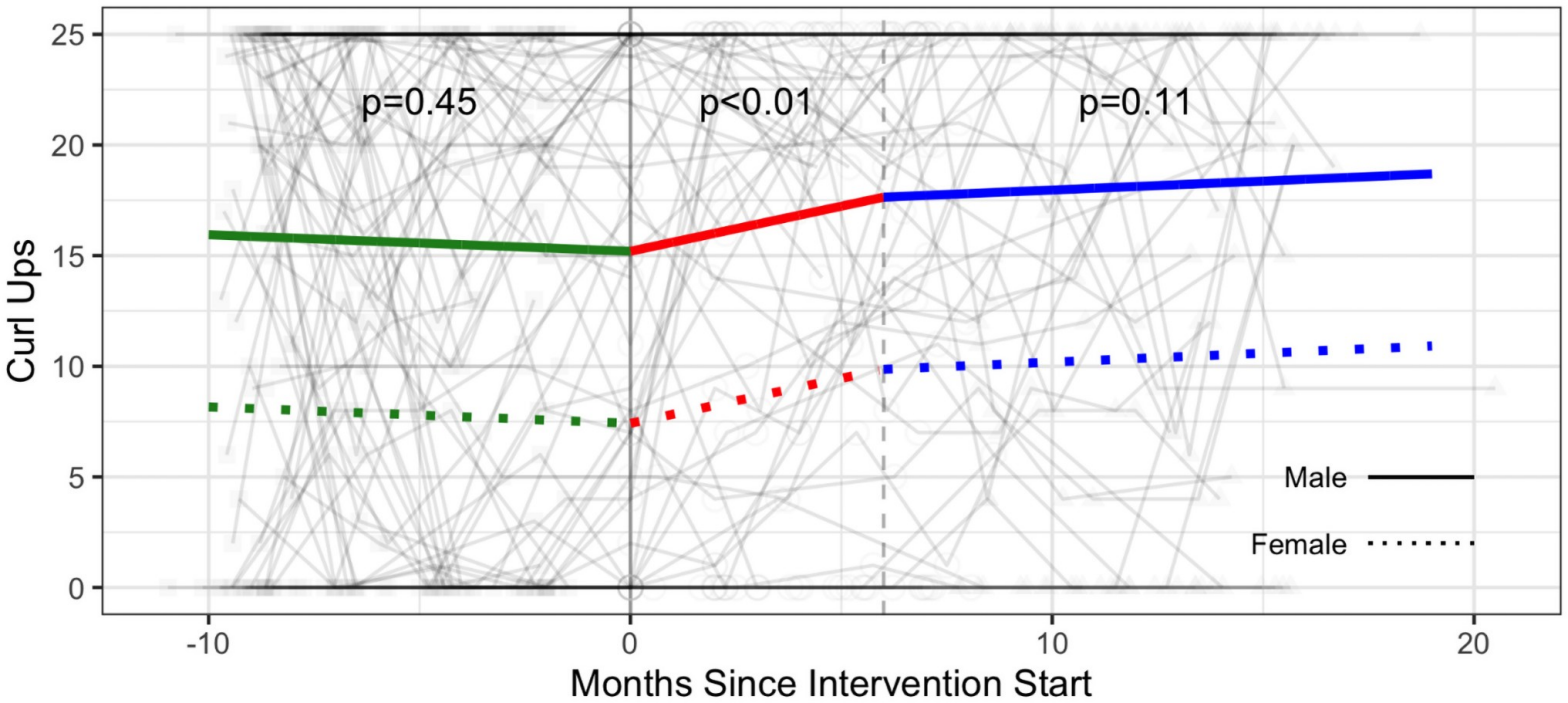
Strength—Curl ups (number of repetitions up to 25 maximum) trajectories over phase 1 (baseline), phase 2 (intervention) and phase 3 (follow-up monitoring); (n = 98 participants). Vertical dotted line indicates end of phase 2 (intention to treat) at 6 months.

**Fig 8:**
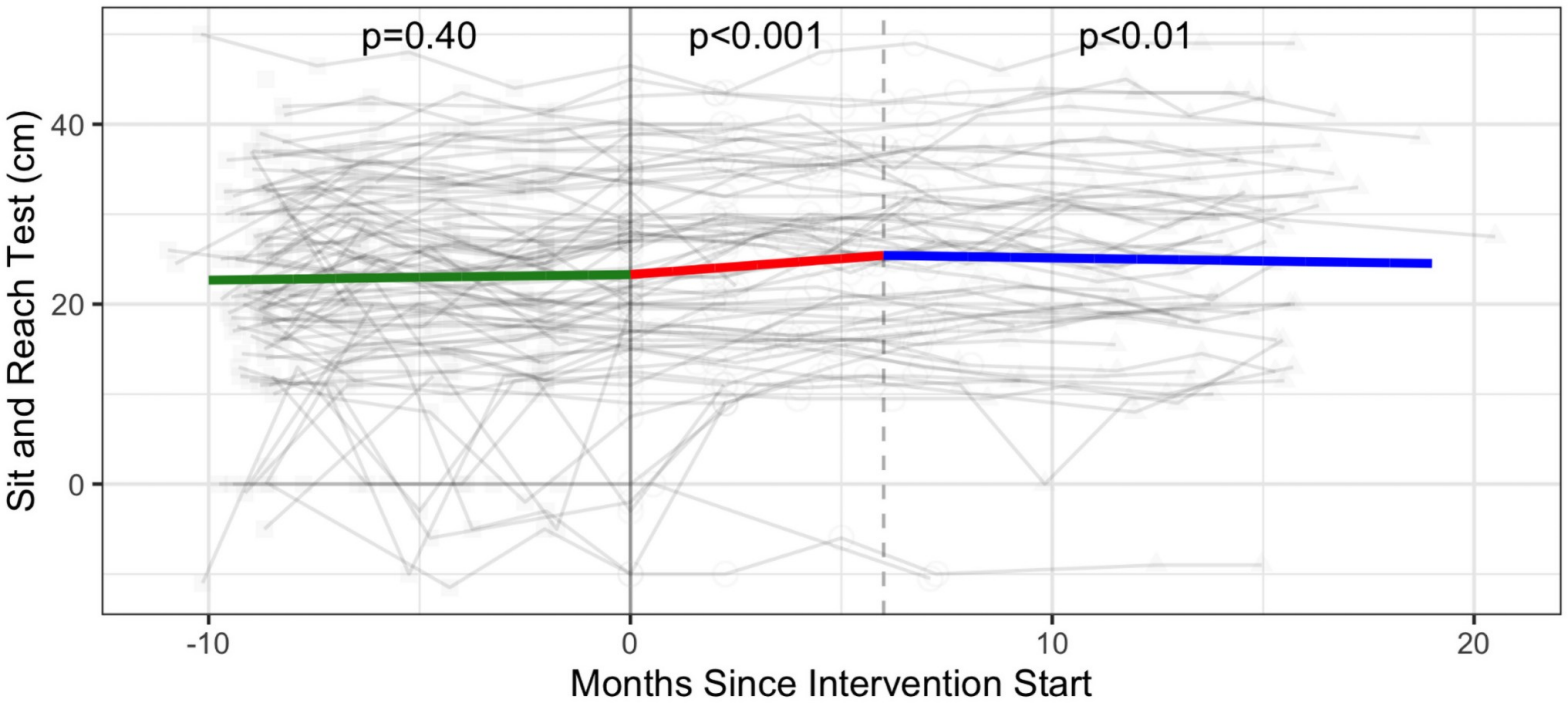
Flexibility—Sit and reach test (cm) trajectories over phase 1 (baseline), phase 2 (intervention) and phase 3 (follow-up monitoring); (n = 99 participants). Vertical dotted line indicates end of phase 2 (intention to treat) at 6 months.

The intervention was associated with a significant increase in the number of push ups (additional 2.30 push ups; 95% CI: 0.69, 3.91), the number of curl ups (+2.89 curl ups; 95% CI: 0.61, 5.17), and the sit and reach test distance (measure of flexibility) (1.74 cm; 95% CI: 0.21, 3.28) (Table 3, Figs 6–8 and S2 Table).

**Fig 9:**
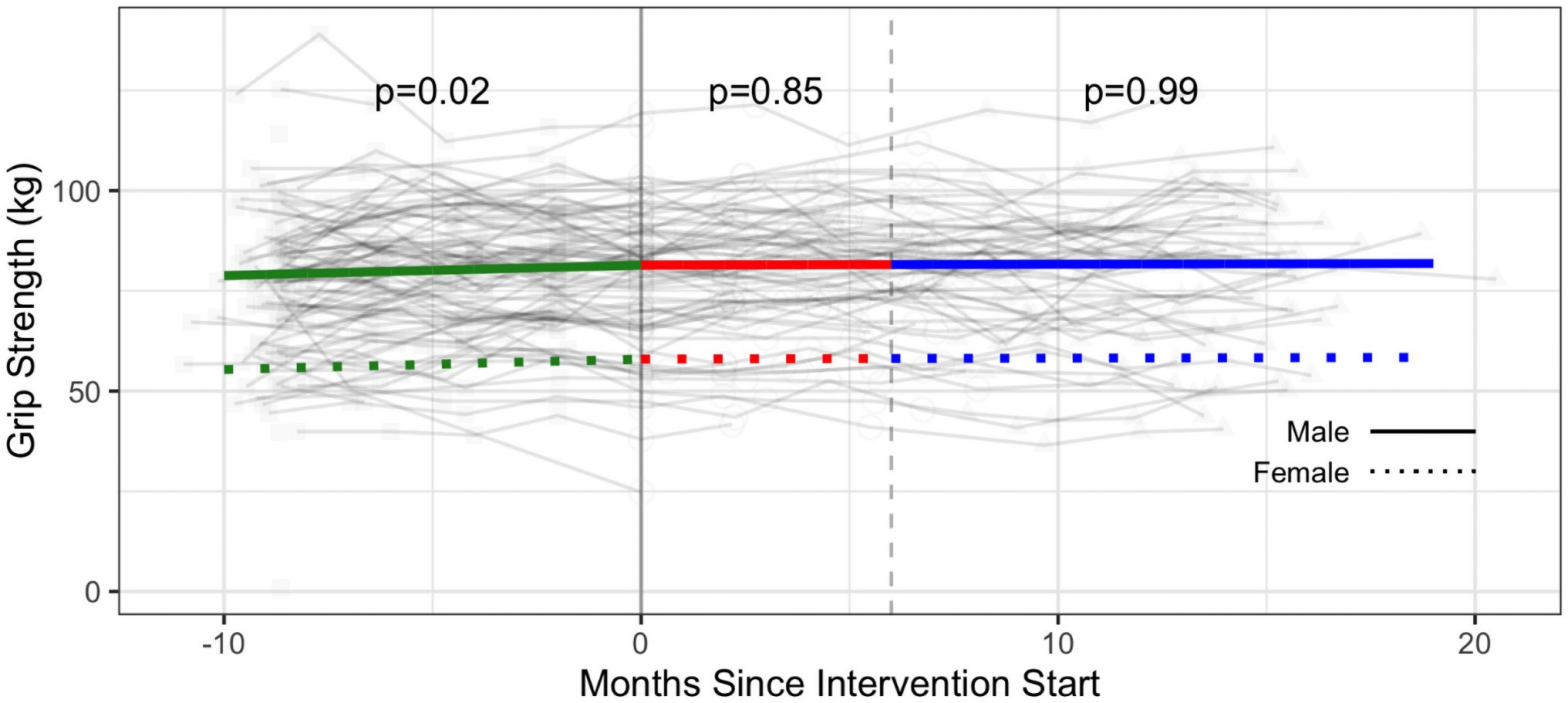
Strength—Upper extremity—Grip strength (kg) trajectories over phase 1 (baseline), phase 2 (intervention) and phase 3 (follow-up monitoring); (n = 101 participants). Vertical dotted line indicates end of phase 2 (intention to treat) at 6 months.

**Fig 10:**
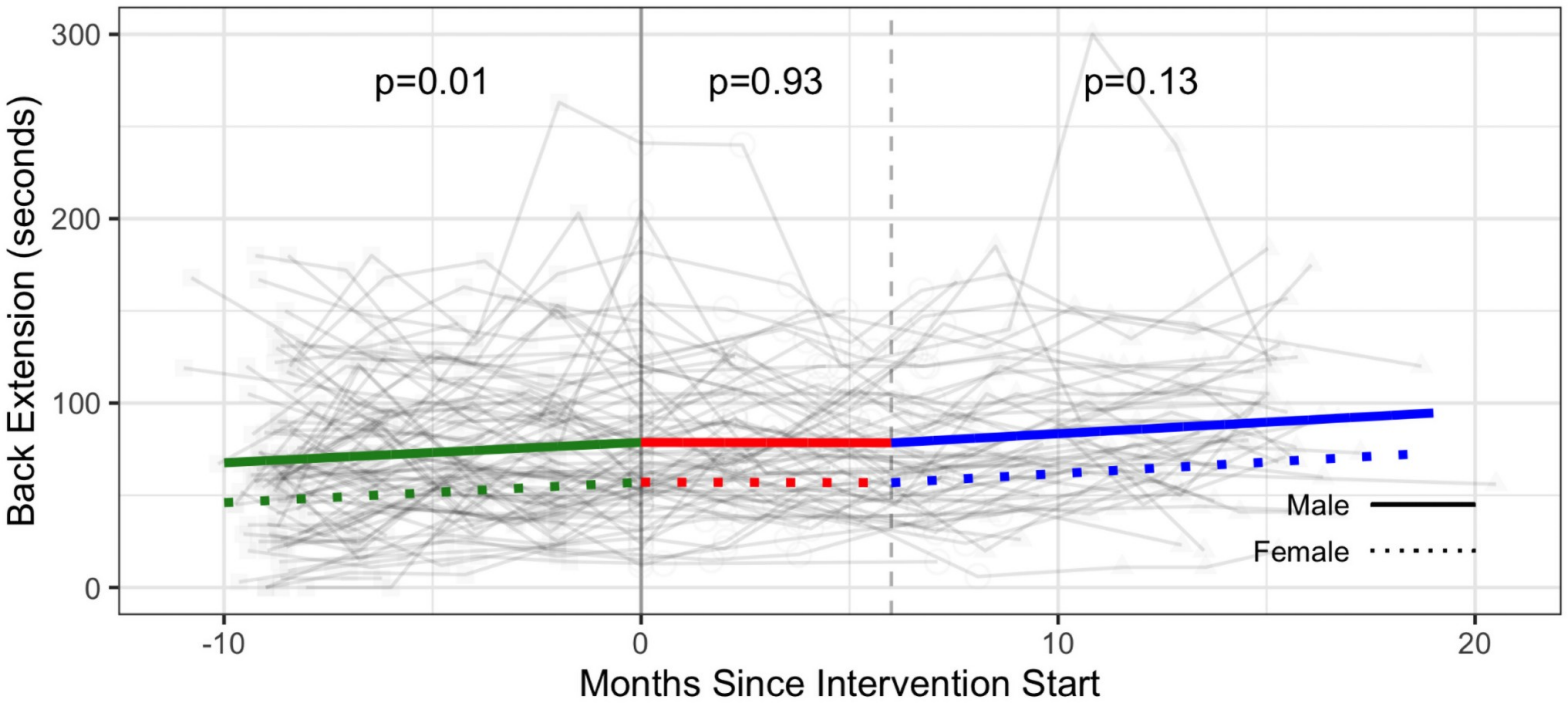
Strength—Back extension (seconds) trajectories over phase 1 (baseline), phase 2 (intervention) and phase 3 (follow-up monitoring); (n = 97 participants). Vertical dotted line indicates end of phase 2 (intention to treat) at 6 months.

**Fig 11:**
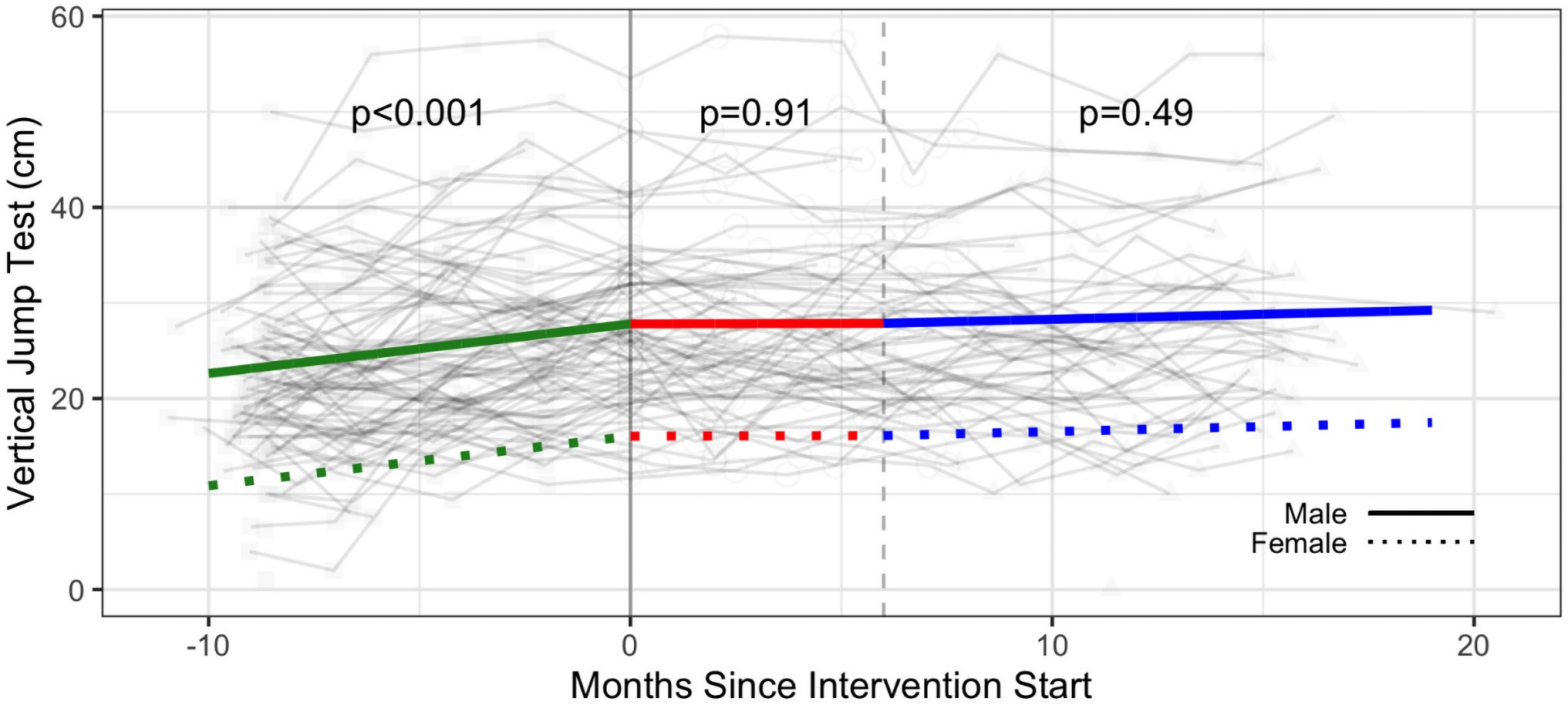
Strength—Lower extremity—Vertical jump test (cm) trajectories over phase 1 (baseline), phase 2 (intervention) and phase 3 (follow-up monitoring); (n = 96 participants). Vertical dotted line indicates end of phase 2 (intention to treat) at 6 months.

Estimated intervention effects were not statistically significant for grip strength and duration of back extension (Table 3, Figs 9–11 and S2 Table).

#### Self-reported physical activity

**Fig 12:**
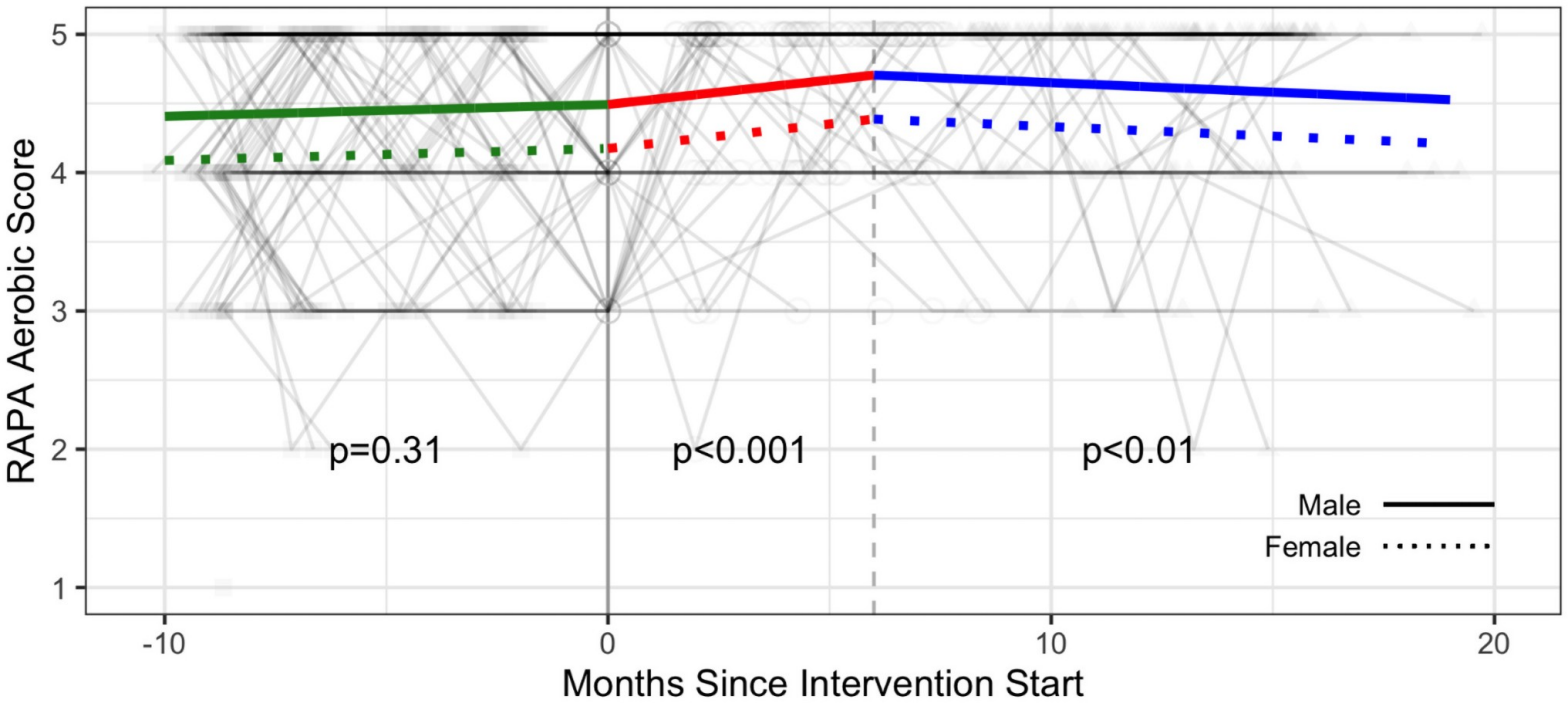
Self-reported physical activity—Rapid Assessment of Physical Activity (RAPA) questionnaire—RAPA 1 (Aerobic) score trajectories over phase 1 (baseline), phase 2 (intervention) and phase 3 (follow-up monitoring); (n = 105 participants). Vertical dotted line indicates end of phase 2 (intention to treat) at 6 months.

There were statistically significant increases in self-reported physical activity over the six-month intervention (Phase 2). For RAPA 1 (aerobic), participants demonstrated a statistically significant monthly increase in scores (0.04 points / month on scale 1–5) in the Phase 2 intervention (p<0.001), followed by a statistically significant decrease in scores in Phase 3 follow-up (-0.01 point / month) compared with Phase 2 (p<0.01) (Fig 12 and S2 Table).

**Fig 13:**
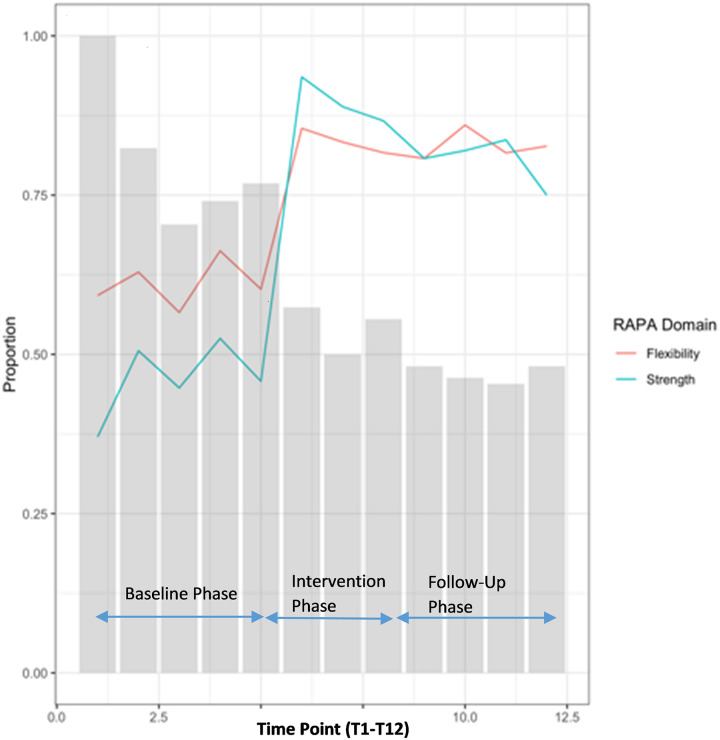
Self-reported physical activity outcome—Rapid Assessment of Physical Activity (RAPA) questionnaire—RAPA 2 (Strength and flexibility) score trajectories over phase 1 (baseline), phase 2 (intervention) and phase 3 (follow-up monitoring); (n = 105 participants).

For RAPA 2 (strength and flexibility) scores, 28 of the 33 (82%) participants not engaging in strength activity at the start of the intervention (T5) were engaged at the end of intervention (T8), while 3 of the 27 (11%) who engaged at T5 had stopped by T8 (McNemar’s χ^2^ = 18.6, p<0.001). For flexibility 15/22 (68%) started flexibility training while 4/38 (11%) stopped (McNemar’s χ^2^ = 5.3, p = 0.02) indicating that more participants engaged in strength and flexibility physical activity after the intervention versus prior to it. Fig 12 illustrates the proportion of participants engaged in strength and flexibility during each phase of the study (Fig 13 and S3 Table).

#### Post hoc analysis

Results for the exploratory post hoc (per protocol and exercise dose adjusted) analyses are detailed in S4–S6 Tables. S4 Table outlines the overall estimated effect of the six-month CBE intervention (Phase 2) over and above the baseline (Phase 1) for each outcome across all analyses (intention to treat and exploratory).

*Per protocol analysis* (S5 Table). Of the 80 participants who started the intervention, 30 (38%) went over the intended 25 weeks, rescheduling their weekly coaching supervision up to 15 weeks into Phase 3 for a total of 40 weeks. Reasons for extending (or rescheduling) weekly coaching sessions included health reasons, travel, work commitments, change in coaching staff, or scheduling communication. When considering all coaching sessions attended by participants (Phase 2 and 3), the estimated intervention effect (taking Phase 1 into account) remained non significant for V̇O_2_peak (1.56mL/kg/min; 95% CI: -0.79, 3.90). (S5 Table details results of the per protocol analyses).

*Exercise dose* (S6 Table). Forty-five (45) participants completed at least one web-based weekly exercise questionnaire resulting in dose data included in the sub-group analysis examining the influence of dose on outcomes. The intervention by dose effect represents the expected increase in outcome for every additional minute spent exercising for every month in the study. Taking exercise dose into account, the primary outcome of V̇O_2_peak, remained non significant when considering exercise dose of ≥150 min/week of MVPA (-0.24 ml/kg/min; 95% CI: -1.48, 1.01) or ≥75 min/week of vigorous physical activity (-0.26 ml/kg/min; 95% CI: -1.68, 1.17) (S6 Table details the results of the exercise dose analysis).

## Discussion

In our study of adults living with HIV who engaged in this six-month CBE intervention, supervised weekly by a fitness coach, we observed no statistically or clinically significant changes in V̇O_2_peak (0.56ml/kg/min; 95% CI: -1.27, 2.39) after taking Phase 1 baseline into account.

Baseline V̇O_2_peak values in our study sample appear to be lower than reported in general populations. The mean baseline V̇O_2_peak among participants in this study (24.1 and 16.7 ml/kg/min in males and females, respectively) were lower than V̇O_2_max reported in the broader population (32-40ml/kg/min for males; 26-32ml/kg/min for females aged 40–59 years) [57]. Cardiorespiratory fitness among adults living with HIV is lower compared with the general population [58, 59] as previously reported by Vancampfort and colleagues who reported V̇O_2_max of 27.4ml/kg/min in a systematic review of 21 studies that pooled data from 1010 adults living with HIV (mean age 41 years; 64% males) [60]. Thus it is important to consider strategies to increase cardiorespiratory fitness among adults living with HIV.

Observed effects of the intervention on V̇O_2_peak in our study were inconclusive. While the point estimate of 0.56 ml/kg/min did not reach statistical significance, the lower and upper limits of the 95% CI (-1.27, 2.39) indicated both a deterioration and clinically important improvement in V̇O_2_peak with CBE. During the baseline phase we observed a small, non significant increase in V̇O_2_peak of 0.12 ml/kg/min per month (95% CI: -0.04, 0.28 ml/kg/min per month) followed by a statistically significant increase during the intervention at 0.21 ml/kg/min per month (95% CI: 0.01, 0.41 ml/kg/min per month), which corresponds to an expected increase of 1.26 ml/kg/min over a six-month intervention (Table 2). While the overall point estimate was <2 ml/kg/min (estimated threshold for important change) the upper limit of the confidence interval was compatible with a potential improvement of 2.46 ml/kg/min [13]. Nevertheless, the estimated effect of 1.26 ml/kg/min was lower than the systematic review literature that reported aerobic and resistance exercise performed at least thrice weekly for at least five weeks resulted in an increase in V̇O_2_max of 2.9 ml/kg/min among exercisers compared with non exercisers living with HIV [12, 13] and a meta-analysis (n = 10 studies) that reported significant improvements in V̇O_2_max of 3.8 ml/kg/min with at least four weeks of intervention among exercisers compared with non-exercisers [11]. These randomized controlled trials often involved highly supervised interventions (thrice weekly), performed in hospital-based rehabilitation settings opposed to our CBE intervention that involved only weekly supervision (Phase 2) followed by entirely independent exercise (Phase 3) in a community-based setting. Our study did not include a comparison group; instead we considered the Phase 1 baseline as a control or monitoring phase of the study from which to estimate an effect. We believe our estimated value of 0.56 ml/kg/min, the additional change during the intervention period, above that observed in the baseline phase, is a conservative estimate of the intervention effect because it provides a measure of the attributable effect of the CBE intervention beyond any effect of enrollment and monitoring in the study. If we interpreted no change in V̇O_2_peak occurring during Phase 1 baseline, the Phase 2 improvement in V̇O_2_peak would be estimated at 1.3 ml/kg/min, a greater improvement (0.211*6 months). While the point estimate remains below the estimated threshold for important change [13], ultimately the study lacks precision to determine either a clear benefit or harm with CBE for V̇O_2_peak. Furthermore, we measured V̇O_2_peak (not V̇O_2_max) which may explain lower values of cardiorespiratory fitness in our study. Similar non significant intervention effects of the point estimate after taking the baseline monitoring phase into account were found with self-reported aerobic physical activity, whereby despite a statistically significant monthly increase in RAPA 1 (aerobic) scores (+0.04 points/month) during the Phase 2 intervention, the overall improvement in aerobic physical activity across the 6 month intervention after taking the baseline phase into was non significant (0.16 points (95% CI: -0.03, 0.35) (S2 Table).

We hypothesize several reasons for our inconclusive findings related to V̇O_2_peak in our study that may include insufficient dose of exercise intensity and duration of intervention achieved by participants within the intervention [61]. The CBE intervention involved a combination of aerobic, resistance, balance and flexibility training, and was not specifically tailored to enhancing aerobic capacity. Oursler and colleagues found a significantly greater increase in V̇O_2_max of 3.6 ml/kg/min among people living with HIV who completed 16 weeks of vigorous intensity aerobic exercise compared with no change in V̇O_2_max among participants who completed 45 minutes of moderate-paced walking [10]. Erlandson and colleagues [62] conducted a thrice weekly 24-week aerobic and resistance exercise intervention comparing older adults living with HIV and without HIV (50–75 years) where after 12 weeks of moderate-intensity of exercise individuals were randomized to 12 additional weeks of moderate or high intensity exercise. Despite similar V̇O_2_max at baseline, older adults with HIV had significantly greater improvements in V̇O_2_max, and physical function compared with older adults without HIV, and significantly greater improvements in strength among those randomized to high-intensity exercise [62]. In our study, we adopted a ‘guided adaptation’ approach whereby the intervention was tailored to the ongoing assessment of the needs and ability of participants by the fitness coach. Participants also were not compensated for their engagement in the CBE intervention as this would have introduced a co-intervention with incentive to exercise. This aligned with our implementation science approach, to evaluate the impact of the intervention in the ‘real world’ setting [34, 63]. There was also considerable variation of V̇O_2_peak within individuals over time which may indicate changes in fluctuations in health status that may occur among adults living with HIV [64] (S1 Fig). Given level of engagement can fluctuate based on level of readiness to exercise [65], along with health fluctuations for people living with HIV [21], it is unlikely all participants exercised at the prescribed intensity and duration.

Beneficial changes with the six-month CBE intervention were found for some secondary measures of cardiovascular health (systolic blood pressure), strength (push ups, curl ups), flexibility (sit and reach) and self-reported physical activity. We found a significant intervention effect with systolic blood pressure. The decreasing rate of systolic BP of ~1 mmHg / month (5 mmHg total) observed in the intervention phase of the study aligns with evidence that exercise can reduce or prevent hypertension among adults [66–68] including people living with HIV [69]. Our results align with systematic review evidence reporting benefits to systolic BP ranging from 1.8 to 10.9 mmHg with endurance and resistive training among adults [70]. Further systematic review evidence indicated a reduction in systolic BP of 10 mmHg can lower the risk of major cardiovascular disease events, coronary heart disease, stroke and heart failure lower risk of mortality from stroke [71]. Hence, results from this study may suggest a trend towards a potential clinically important benefit to cardiovascular health for people living with HIV [72]. Despite the decreasing trend in systolic BP observed in the intervention phase, results were ultimately inconclusive for V̇O_2_peak, highlighting this as an important area for investigation, examining the impact of CBE on cardiovascular health for people living with HIV.

Results demonstrated significant improvements for select strength (push ups and curl ups) and flexibility (sit and reach test) outcomes during the intervention after taking the baseline monitoring phase into account. Benefits to strength were sustained in the follow-up monitoring phase (Phase 3). We hypothesize reasons for improvements to strength and flexibility may be associated with specificity of training whereby push ups, curl ups, and hamstring flexibility assessed in the fitness testing were analogous to exercises in the participants’ CBE intervention. Improvements in the baseline monitoring phase observed for some strength outcomes (grip strength, vertical jump, back extension) may reflect participants initiating independent exercise during the baseline phase or becoming familiarized with the strength assessments over time.

Improvements in some outcomes found during the intervention were not sustained in the follow-up phase, such as self-reported aerobic physical activity (Fig 12 and S2 Table). Similarly, a small decline was observed in V̇O_2_peak in Phase 3 (Table 2). We posit that weekly supervision from the fitness coaches during the Phase 2 intervention may be important for participants to remain engaged in the intervention (and the study). Results of a systematic review that investigated dropout from physical activity interventions suggest that supervised exercise with highly qualified personnel is associated with lower withdrawals from physical activity interventions among people living with HIV [73]. In this study, 84% (67/80) of participants who started the Phase 2 intervention completed it 6 months later, and 77% (52/67) went on to complete the 8 month Phase 3 monitoring phase. Our intervention included a combination of features aimed at reducing barriers to engaging in exercise for people living with HIV such as providing a gym membership, personalized fitness instruction, and monthly self-management educational sessions; we also allowed participants to reschedule their weekly coaching sessions to accommodate for episodes of illness [21, 22]. Nevertheless, most benefits found during the intervention were not sustained in the follow-up monitoring phase, despite continued access to the YMCA membership, highlighting the potential importance of the one-on-one fitness coaching. Personalized coaching is a costly feature of exercise interventions whereby individuals may not be able to sustain out-of-pocket expenses for personalized training over the long term. Future research should explore the role of group-based exercise, peer-support and different self-management approaches as potentially less costly features of CBE interventions to promote the sustained uptake of physical activity over time [74, 75]. Ascertaining who might benefit most from supervised CBE, and identifying strategies to facilitate the sustained benefits of, and engagement in, physical activity after a CBE intervention needs further exploration.

To our knowledge, this is the first examination of cardiorespiratory fitness, cardiovascular health, strength, flexibility and physical activity involving a multi-phased CBE intervention in a ‘real world’ setting with adults living with HIV. Strengths include our prospective longitudinal three-phased study design whereby data were collected at multiple timepoints before and after CBE introduction at T5 to detect whether CBE had an effect significantly greater than the underlying baseline trend [76]. Additional strengths included our implementation science approach offering insights on changes in outcomes that can occur among adults living with HIV exercising in a ‘real world’ community fitness setting, and the sustainability of the intervention-effect over time with an independent self-maintenance phase of exercise. By examining the impact of this six-month supervised CBE intervention we focused on the ‘E-effectiveness’ component of the RE-AIM framework within our broader implementation science approach [42]. In other work, we report on subsequent features of adoption and implementation to further understand the process, uptake and sustainability of implementing CBE with adults living with HIV in the community [77].

### Limitations

We did not achieve our original intended sample size of 75 participants at study completion due to participant withdrawal, and particpants may have missed assessments across the 22-month phase of study. However, we used mixed models including available data for all participants across the three phases. Because of the difficulty in adjusting for multiple comparisons with correlated outcomes we did not adjust our p-values, but have presented confidence intervals for our estimates. We did not adjust for multiple comparisons of the secondary outcomes [78]. As such, results should be considered exploratory. We did not objectively measure exercise intensity of each session, and using weekly self-report recall which limited our ability to measure the intensity of exercise among participants in the study. Finally few females (<15% of the sample) participated in the study. Future work should consider CBE features to address structural, geographical and personal barriers to engaging in exercise [79, 80]. Lastly, this study was conducted in a single urban community fitness centre in Toronto, Canada. Participants who enrolled in this 22-month study may have been more likely to possess a higher level of self-health awareness compared to the broader HIV population. Generalizability of findings to the broader HIV population, other fitness centres, and in other contexts such as low-and middle income countries is unclear.

## Conclusions

Community-dwelling adults living with HIV who engaged in a six-month CBE intervention supervised weekly by a fitness coach demonstrated inconclusive results with respect to V̇O_2_peak, and potential improvements in some outcomes of cardiovascular health, strength, flexibility and self-reported physical activity. CBE is an approach to consider for improving health outcomes with adults living with HIV within a self-management framework. Future research should consider features tailored to promote uptake and sustained engagement in independent exercise among adults living with episodic illness such as HIV.

## Supporting information

S1 Fig Individual V̇O_2_peak trajectory plots across Phase 1 (baseline), Phase 2 (intervention) and Phase 3 (follow-up monitoring). The line indicates participant-level predictions from the model. Trajectories indicate the fitted values across all three study phases. Red dots indicate V̇O_2_peak measurements in Phase 1 (baseline monitoring). Green dots indicate V̇O_2_peak measurements in Phase 2 (intervention). Blue dots indicate V̇O_2_peak measurements in Phase 3 (follow-up monitoring).
(PDF)

S1 File Statistical supplement. (PDF)

S2 File Research ethics board approval letter and original approved protocol (April 2016). (PDF)

S1 Table Characteristics of participants at study initiation in the study sample at enrollment, start of intervention, end of intervention, and end of follow-up (end of study). Characteristics for participants who completed demographic questionnaire at study enrollment (Time 1; Month 0): ^a^ excluding HIV.
(PDF)

S2 Table Results—Secondary outcomes—Primary (Intention to treat) analysis. Slopes are change in outcome over one month. ^a^ Significant trend in Phase 1 (baseline) slope versus 0; ^b^ Significant trend in Phase 2 (intervention) slope versus 0; ^c^ Significant change in Phase 3 (follow-up monitoring) slope versus Phase 2 (intervention) slope. ^d^ Follow-up slope is the difference in slope between the follow-up and intervention phase; RAPA: Rapid Assessment of Physical Activity Questionnaire (higher scores indicate greater physical activity).
(PDF)

S3 Table RAPA 2—Strength and flexibility scores. ^a^One of these participants did not have T8 data so is not included in McNemar’s below; ^b^For strength counts, participants reporting either Strength or Both were combined; ^c^ For flexibility counts, participants reporting either Flexibility or Both were combined; ^d^ Same number of participants moving from not engaging to engaging in flexibility were the same as the number of participants moving from engaging in flexibility to not; RAPA: Rapid Assessment of Physical Activity Questionnaire.
(PDF)

S4 Table Overall estimated effects of the Phase 2—CBE intervention after taking the Phase 1—Baseline monitoring into account for all outcomes and analyses: Primary analysis (intention to treat) and secondary (exploratory, post hoc) analyses (per protocol and exercise dose analysis). ^a^ Significant overall estimated treatment effect over the six-month intervention after taking the baseline monitoring into account; RAPA: Rapid Assessment of Physical Activity; CI: Confidence Interval; V̇O2peak: peak oxygen consumption. *Sample size*: Intention to Treat Analysis: Maximum n = 105 participants; Post Hoc Exploratory Per Protocol Analysis: Maximum n = 80 participants; Post Hoc Exploratory Dosage Analysis: Models A and B: Maximum: 45 participants.
(PDF)

S5 Table Post hoc exploratory analyses—Per protocol—Comparing trends during the baseline monitoring phase (defined as any time prior to the first supervised training session) and the trends while receiving the intervention (defined as the time between the first and last supervised training session). Slopes are change in outcome over one month; Baseline slope (trend) p value <0.05 = significant difference in baseline slope versus 0; Slope while receiving the intervention (trend) p value <0.05 = significant difference in slope during the intervention versus 0; RAPA: Rapid Assessment of Physical Activity; RAPA Aerobic (range: 1–5); CI: Confidence Interval.
(PDF)

S6 Table Post hoc exploratory analyses—Exercise dose interaction of exercise dose and duration of the intervention on all outcomes. Model A: Dose defined as total number of Moderate + Vigorous minutes of activity in the past week; Model B: Dose defined as total number of Vigorous minutes of activity in the past week. Canadian Physical Activity Guidelines (CPAG): Model A: ≥150 minutes moderate to vigorous / week and Model B: ≥75 minutes vigorous activity. Slopes are change in outcome over one month. ^a^Follow-up slope is the difference in slope between the follow-up and intervention phase; Phase 1: Baseline slope p value <0.05: statistically significant difference in Phase 1 (baseline) slope versus 0; Phase 2: Intervention slope p value <0.05: statistically significant difference in Phase 2 (intervention) slope versus 0; Phase 3: Follow-up slope p value <0.05: statistically significant change in Phase 3 (follow-up monitoring) slope versus Phase 2 (intervention) slope.
(PDF)

S1 Checklist TREND checklist. (PDF)

## References

[1] Antiretroviral Therapy Cohort Collaboration. Survival of HIV-positive patients starting antiretroviral therapy between 1996 and 2013: a collaborative analysis of cohort studies. The lancet HIV. 2017;4(8):e349–e56. doi: 10.1016/S2352-3018(17)30066-8 28501495 PMC5555438

[2] Autenrieth CS, Beck EJ, Stelzle D, Mallouris C, Mahy M, Ghys P. Global and regional trends of people living with HIV aged 50 and over: Estimates and projections for 2000–2020. PloS one. 2018;13(11):e0207005. Epub 2018/11/30. doi: 10.1371/journal.pone.0207005 30496302 PMC6264840

[3] Smit M, Brinkman K, Geerlings S, Smit C, Thyagarajan K, Sighem A, et al. Future challenges for clinical care of an ageing population infected with HIV: a modelling study. The Lancet infectious diseases. 2015;15(7):810–8. doi: 10.1016/S1473-3099(15)00056-0 26070969 PMC4528076

[4] Wong C, Gange SJ, Moore RD, Justice AC, Buchacz K, Abraham AG, et al. Multimorbidity Among Persons Living with Human Immunodeficiency Virus in the United States. Clin Infect Dis. 2018;66(8):1230–8. Epub 2017/11/18. doi: 10.1093/cid/cix998 29149237 PMC5889007

[5] Hasse B, Ledergerber B, Furrer H, Battegay M, Hirschel B, Cavassini M, et al. Morbidity and aging in HIV-infected persons: the Swiss HIV cohort study. Clin Infect Dis. 2011;53(11):1130–9. Epub 2011/10/15. doi: 10.1093/cid/cir626 21998280

[6] Kendall CE, Wong J, Taljaard M, Glazier RH, Hogg W, Younger J, et al. A cross-sectional, population-based study measuring comorbidity among people living with HIV in Ontario. BMC public health. 2014;14(1):161. Epub 2014/02/15. doi: 10.1186/1471-2458-14-161 24524286 PMC3933292

[7] Vance DE, Mugavero M, Willig J, Raper JL, Saag MS. Aging With HIV: A Cross-Sectional Study of Comorbidity Prevalence and Clinical Characteristics Across Decades of Life. J Assoc Nurses AIDS Care. 2011;22(1):17–25. doi: 10.1016/j.jana.2010.04.002 20471864

[8] O’Brien KK, Hanna S, Solomon P, Worthington C, Ibanez-Carrasco F, Chan Carusone S, et al. Characterizing the disability experience among adults living with HIV: a structural equation model using the HIV disability questionnaire (HDQ) within the HIV, health and rehabilitation survey. BMC infectious diseases. 2019;19(1):594. Epub 2019/07/10. doi: 10.1186/s12879-019-4203-0 31286891 PMC6615082

[9] Ozemek C, Erlandson KM, Jankowski CM. Physical activity and exercise to improve cardiovascular health for adults living with HIV. Progress in cardiovascular diseases. 2020;63(2):178–83. Epub 2020/02/06. doi: 10.1016/j.pcad.2020.01.005 32014512

[10] Oursler KK, Sorkin JD, Ryan AS, Katzel LI. A pilot randomized aerobic exercise trial in older HIV-infected men: Insights into strategies for successful aging with HIV. PloS one. 2018;13(6):e0198855. Epub 2018/06/13. doi: 10.1371/journal.pone.0198855 29894513 PMC5997336

[11] Ibeneme SC, Omeje C, Myezwa H, Ezeofor SN, Anieto EM, Irem F, et al. Effects of physical exercises on inflammatory biomarkers and cardiopulmonary function in patients living with HIV: a systematic review with meta-analysis. BMC infectious diseases. 2019;19(1):359. Epub 2019/05/01. doi: 10.1186/s12879-019-3960-0 31035959 PMC6489236

[12] O’Brien KK, Tynan AM, Nixon SA, Glazier RH. Effectiveness of Progressive Resistive Exercise (PRE) in the context of HIV: systematic review and meta-analysis using the Cochrane Collaboration protocol. BMC infectious diseases. 2017;17(1):268. doi: 10.1186/s12879-017-2342-8 28403830 PMC5389006

[13] O’Brien KK, Tynan AM, Nixon SA, Glazier RH. Effectiveness of aerobic exercise for adults living with HIV: systematic review and meta-analysis using the Cochrane Collaboration protocol. BMC infectious diseases. 2016;16(1):182. doi: 10.1186/s12879-016-1478-2 27112335 PMC4845358

[14] Gomes-Neto M, Conceicao CS, Oliveira Carvalho V, Brites C. A systematic review of the effects of different types of therapeutic exercise on physiologic and functional measurements in patients with HIV/AIDS. Clinics (Sao Paulo). 2013;68(8):1157–67. Epub 2013/09/17. doi: 10.6061/clinics/2013(08)16 24037014 PMC3752639

[15] Vancampfort D, Mugisha J, De Hert M, Probst M, Firth J, Gorczynski P, et al. Global physical activity levels among people living with HIV: a systematic review and meta-analysis. Disabil Rehabil. 2018;40(4):388–97. Epub 2016/12/09. doi: 10.1080/09638288.2016.1260645 27929355

[16] Pavone RM, Burnett KF, LaPerriere A, Perna FM. Social cognitive and physical health determinants of exercise adherence for HIV-1 seropositive, early symptomatic men and women. Int J Behav Med. 1998;5(3):245–58. Epub 2005/10/28. doi: 10.1207/s15327558ijbm0503_5 16250705

[17] Petroczi A, Hawkins K, Jones G, Naughton DP. HIV Patient Characteristics that Affect Adherence to Exercise Programmes: An Observational Study. The open AIDS journal. 2010;4:148–55. Epub 2010/09/28. doi: 10.2174/1874613601004010148 20871752 PMC2944991

[18] Ciccolo JT, Jowers EM, Bartholomew JB. The benefits of exercise training for quality of life in HIV/AIDS in the post-HAART era. Sports Med. 2004;34(8):487–99. Epub 2004/07/14. doi: 10.2165/00007256-200434080-00001 15248786

[19] Jones G, Hawkins K, Mullin R, Nepusz T, Naughton DP, Sheeran P, et al. Understanding how adherence goals promote adherence behaviours: a repeated measure observational study with HIV seropositive patients. BMC public health. 2012;12:587. Epub 2012/08/03. doi: 10.1186/1471-2458-12-587 22853824 PMC3490813

[20] Ley C, Barrio MR, Leach L. Social-Ecological, Motivational and Volitional Factors for Initiating and Maintaining Physical Activity in the Context of HIV. The open AIDS journal. 2015;9:96–103. Epub 2015/11/21. doi: 10.2174/1874613601509010096 26587078 PMC4645898

[21] Montgomery CA, Henning KJ, Kantarzhi SR, Kideckel TB, Yang CF, O’Brien KK. Experiences participating in a community-based exercise programme from the perspective of people living with HIV: a qualitative study. BMJ Open. 2017;7(4):e015861. Epub 2017/04/06. doi: 10.1136/bmjopen-2017-015861 28377397 PMC5387963

[22] Li A, McCabe T, Silverstein E, Dragan S, Salbach NM, Zobeiry M, et al. Community-Based Exercise in the Context of HIV. Journal of the International Association of Providers of AIDS Care. 2017: doi: 10.1177/2325957416686836 28074681

[23] Gomes Neto M, Conceicao CS, Oliveira Carvalho V, Brites C. Effects of Combined Aerobic and Resistance Exercise on Exercise Capacity, Muscle Strength and Quality of Life in HIV-Infected Patients: A Systematic Review and Meta-Analysis. PloS one. 2015;10(9):e0138066. Epub 2015/09/18. doi: 10.1371/journal.pone.0138066 26378794 PMC4574781

[24] Winward C. Supporting community-based exercise in long-term neurological conditions: experience from the Long-term Individual Fitness Enablement (LIFE) project. Clin Rehabil. 2011;25(7):579–87. Epub 2011/06/24. doi: 10.1177/0269215510392075 21697208

[25] Salbach NM, Howe JA, Brunton K, Salisbury K, Bodiam L. Partnering to Increase Access to Community Exercise Programs for People With Stroke, Acquired Brain Injury and Multiple Sclerosis. Journal of physical activity & health. 2013. Epub 2013/05/17. 10.1123/jpah.2012-0183 23676952

[26] Stuart M, Benvenuti F, Macko R, Taviani A, Segenni L, Mayer F, et al. Community-based adaptive physical activity program for chronic stroke: feasibility, safety, and efficacy of the Empoli model. Neurorehabilitation and neural repair. 2009;23(7):726–34. Epub 2009/03/26. doi: 10.1177/1545968309332734 19318465 PMC3024240

[27] Lee LL, Arthur A, Avis M. Using self-efficacy theory to develop interventions that help older people overcome psychological barriers to physical activity: a discussion paper. Int J Nurs Stud. 2008;45(11):1690–9. Epub 2008/05/27. doi: 10.1016/j.ijnurstu.2008.02.012 18501359

[28] Salbach NM, Howe JA, Baldry D, Merali S, Munce SEP. Considerations for expanding community exercise programs incorporating a healthcare-recreation partnership for people with balance and mobility limitations: a mixed methods evaluation. BMC research notes. 2018;11(1):214. Epub 2018/04/04. doi: 10.1186/s13104-018-3313-x 29609662 PMC5879753

[29] Merali S, Cameron JI, Barclay R, Salbach NM. Experiences of people with stroke and multiple sclerosis and caregivers of a community exercise programme involving a healthcare-recreation partnership. Disabil Rehabil. 2020;42(9):1220–6. Epub 2019/01/23. doi: 10.1080/09638288.2018.1519042 30668173

[30] Davis AM, Kennedy D, Wong R, Robarts S, Skou ST, McGlasson R, et al. Cross-cultural adaptation and implementation of Good Life with osteoarthritis in Denmark (GLA:D): group education and exercise for hip and knee osteoarthritis is feasible in Canada. Osteoarthritis Cartilage. 2018;26(2):211–9. Epub 2017/11/18. doi: 10.1016/j.joca.2017.11.005 29146385

[31] O’Brien KK, Bayoumi AM, Solomon P, Tang A, Murzin K, Chan Carusone S, et al. Evaluating a community-based exercise intervention with adults living with HIV: protocol for an interrupted time series study. BMJ Open. 2016;6(10):e013618. Epub 2016/11/01. doi: 10.1136/bmjopen-2016-013618 27798038 PMC5073553

[32] Biglan A, Ary D, Wagenaar AC. The value of interrupted time-series experiments for community intervention research. Prevention science: the official journal of the Society for Prevention Research. 2000;1(1):31–49. Epub 2001/08/18. doi: 10.1023/a:1010024016308 11507793 PMC4553062

[33] Kokkinos P, Myers J. Exercise and physical activity: clinical outcomes and applications. Circulation. 2010;122(16):1637–48. Epub 2010/10/20. doi: 10.1161/CIRCULATIONAHA.110.948349 20956238

[34] Glasgow RE, Eckstein ET, Elzarrad MK. Implementation science perspectives and opportunities for HIV/AIDS research: integrating science, practice, and policy. J Acquir Immune Defic Syndr. 2013;63 Suppl 1:S26–31. Epub 2013/05/17. 23673882 10.1097/QAI.0b013e3182920286

[35] Gaglio B, Shoup JA, Glasgow RE. The RE-AIM framework: a systematic review of use over time. Am J Public Health. 2013;103(6):e38–46. Epub 2013/04/20. doi: 10.2105/AJPH.2013.301299 23597377 PMC3698732

[36] Glasgow RE, Nelson CC, Strycker LA, King DK. Using RE-AIM metrics to evaluate diabetes self-management support interventions. American journal of preventive medicine. 2006;30(1):67–73. Epub 2006/01/18. doi: 10.1016/j.amepre.2005.08.037 16414426

[37] Glasgow RE, Klesges LM, Dzewaltowski DA, Estabrooks PA, Vogt TM. Evaluating the impact of health promotion programs: using the RE-AIM framework to form summary measures for decision making involving complex issues. Health education research. 2006;21(5):688–94. Epub 2006/09/02. doi: 10.1093/her/cyl081 16945984

[38] Warburton DER, Jamnik VK, Bredin SSD, Gledhill N, on behalf of the PAR-Q+ Collaboration. The Physical Activity Readiness Questionnaire for Everyone (PAR-Q+) and Electronic Physical Activity Readiness Medical Examination (ePARmed-X+). Health & Fitness Journal of Canada. 2011;4(2):3–23.

[39] Prochaska JO, Velicer WF. The transtheoretical model of health behavior change. American journal of health promotion: AJHP. 1997;12(1):38–48. Epub 1997/08/05. doi: 10.4278/0890-1171-12.1.38 10170434

[40] Elzarrad MK, Eckstein ET, Glasgow RE. Applying chronic illness care, implementation science, and self-management support to HIV. American journal of preventive medicine. 2013;44(1 Suppl 2):S99–107. Epub 2013/01/04. doi: 10.1016/j.amepre.2012.09.046 23253770

[41] Garber CE, Blissmer B, Deschenes MR, Franklin BA, Lamonte MJ, Lee IM, et al. American College of Sports Medicine position stand. Quantity and quality of exercise for developing and maintaining cardiorespiratory, musculoskeletal, and neuromotor fitness in apparently healthy adults: guidance for prescribing exercise. Med Sci Sports Exerc. 2011;43(7):1334–59. Epub 2011/06/23. 21694556 10.1249/MSS.0b013e318213fefb

[42] Glasgow RE, McKay HG, Piette JD, Reynolds KD. The RE-AIM framework for evaluating interventions: what can it tell us about approaches to chronic illness management? Patient Educ Couns. 2001;44(2):119–27. Epub 2001/08/02. doi: 10.1016/s0738-3991(00)00186-5 11479052

[43] Canadian Society for Exercise Physiology. The CSEP Physical Activity Training for Health^®^ Resource Manual. https://store.csep.ca/pages/csep-path-manual2019.

[44] American College of Sports Medicine. ACSM’s guidelines for exercise testing and prescription. Philadelphia: Lippincott Wilams &Wilkins; 2013. 10.1249/JSR.0b013e31829a68cf 23851406

[45] Pickering TG, Hall JE, Appel LJ, Falkner BE, Graves J, Hill MN, et al. Recommendations for blood pressure measurement in humans and experimental animals: part 1: blood pressure measurement in humans: a statement for professionals from the Subcommittee of Professional and Public Education of the American Heart Association Council on High Blood Pressure Research. Circulation. 2005;111(5):697–716. Epub 2005/02/09. doi: 10.1161/01.CIR.0000154900.76284.F6 15699287

[46] Wells KF, Dillon EK. The sit and reach. A test of back and leg flexibility. Research Quarterly. 1952;23:115–8.

[47] Topolski TD, LoGerfo J, Patrick DL, Williams B, Walwick J, Patrick MB. The Rapid Assessment of Physical Activity (RAPA) among older adults. Prev Chronic Dis. 2006;3(4):A118. 16978493 PMC1779282

[48] Ross R, Tremblay M. Introduction to the Canadian 24-Hour Movement Guidelines for Adults aged 18–64 years and Adults aged 65 years or older: an integration of physical activity, sedentary behaviour, and sleep. Applied physiology, nutrition, and metabolism = Physiologie appliquee, nutrition et metabolisme. 2020;45(10 (Suppl. 2)):v–xi. Epub 2020/10/16. doi: 10.1139/apnm-2020-0843 33054330

[49] Ross R, Chaput JP, Giangregorio LM, Janssen I, Saunders TJ, Kho ME, et al. Canadian 24-Hour Movement Guidelines for Adults aged 18–64 years and Adults aged 65 years or older: an integration of physical activity, sedentary behaviour, and sleep. Applied physiology, nutrition, and metabolism = Physiologie appliquee, nutrition et metabolisme. 2020;45(10 (Suppl. 2)):S57–S102. Epub 2020/10/16. 10.1139/apnm-2020-0467 33054332

[50] Kaminsky LA, Arena R, Myers J. Reference Standards for Cardiorespiratory Fitness Measured With Cardiopulmonary Exercise Testing: Data From the Fitness Registry and the Importance of Exercise National Database. Mayo Clinic proceedings Mayo Clinic. 2015;90(11):1515–23. Epub 2015/10/13. 10.1016/j.mayocp.2015.07.026 PMC4919021 26455884

[51] Peters SA, Bots ML, den Ruijter HM, Palmer MK, Grobbee DE, Crouse JR 3rd, et al. Multiple imputation of missing repeated outcome measurements did not add to linear mixed-effects models. J Clin Epidemiol. 2012;65(6):686–95. Epub 2012/03/31. doi: 10.1016/j.jclinepi.2011.11.012 22459429

[52] R Core Team. R A Language and Environment for Statistical Computing [Internet] Vienna, Austria: R Foundation for Statistical Computing; 2019.

[53] Pinheiro J, Bates D. R-core. nlme: Linear and Nonlinear Mixed Effects Models2019. https://CRAN.R-project.org/package=nlme.

[54] Zhang F, Wagner AK, Ross-Degnan D. Simulation-based power calculation for designing interrupted time series analyses of health policy interventions. J Clin Epidemiol. 2011;64(11):1252–61. Epub 2011/06/07. doi: 10.1016/j.jclinepi.2011.02.007 21640554

[55] McLeod AI, Vingilis ER. Power computations in time series analyses for traffic safety interventions. Accident; analysis and prevention. 2008;40(3):1244–8. Epub 2008/05/08. doi: 10.1016/j.aap.2007.10.007 18460394 PMC2447167

[56] Wagner AK, Soumerai SB, Zhang F, Ross-Degnan D. Segmented regression analysis of interrupted time series studies in medication use research. Journal of clinical pharmacy and therapeutics. 2002;27(4):299–309. Epub 2002/08/14. doi: 10.1046/j.1365-2710.2002.00430.x 12174032

[57] Rapp D, Scharhag J, Wagenpfeil S, Scholl J. Reference values for peak oxygen uptake: cross-sectional analysis of cycle ergometry-based cardiopulmonary exercise tests of 10 090 adult German volunteers from the Prevention First Registry. BMJ Open. 2018;8(3):e018697. Epub 2018/03/07. doi: 10.1136/bmjopen-2017-018697 29506981 PMC5855221

[58] Chisati EM, Vasseljen O. Aerobic endurance in HIV-positive young adults and HIV-negative controls in Malawi. Malawi medical journal: the journal of Medical Association of Malawi. 2015;27(1):5–9. Epub 2015/07/03. doi: 10.4314/mmj.v27i1.2 26137190 PMC4478397

[59] Oursler KK, Sorkin JD, Smith BA, Katzel LI. Reduced aerobic capacity and physical functioning in older HIV-infected men. AIDS Res Hum Retroviruses. 2006;22(11):1113–21. Epub 2006/12/07. doi: 10.1089/aid.2006.22.1113 17147498

[60] Vancampfort D, Mugisha J, Rosenbaum S, Firth J, De Hert M, Probst M, et al. Cardiorespiratory fitness levels and moderators in people with HIV: A systematic review and meta-analysis. Preventive medicine. 2016;93:106–14. Epub 2016/11/05. doi: 10.1016/j.ypmed.2016.10.001 27713101

[61] Hoogeboom TJ, Kousemaker MC, van Meeteren NL, Howe T, Bo K, Tugwell P, et al. i-CONTENT tool for assessing therapeutic quality of exercise programs employed in randomised clinical trials. British journal of sports medicine. 2020. Epub 2020/11/05. doi: 10.1136/bjsports-2019-101630 33144350 PMC8479742

[62] Erlandson KM, MaWhinney S, Wilson M, Gross L, McCandless SA, Campbell TB, et al. Physical function improvements with moderate or high-intensity exercise among older adults with or without HIV infection. Aids. 2018;32(16):2317–26. Epub 2018/08/23. doi: 10.1097/QAD.0000000000001984 30134299 PMC6170687

[63] Damschroder LJ, Aron DC, Keith RE, Kirsh SR, Alexander JA, Lowery JC. Fostering implementation of health services research findings into practice: a consolidated framework for advancing implementation science. Implement Sci. 2009;4:50. Epub 2009/08/12. doi: 10.1186/1748-5908-4-50 19664226 PMC2736161

[64] Solomon P, O’Brien KK, Nixon S, Letts L, Baxter L, Gervais N. Trajectories of Episodic Disability in People Aging with HIV: A Longitudinal Qualitative Study. Journal of the International Association of Providers of AIDS Care. 2018;17: doi: 10.1177/2325958218759210 29464973 PMC6748469

[65] Simonik A, Vader K, Ellis D, Kesbian D, Leung P, Jachyra P, et al. Are you ready? Exploring readiness to engage in exercise among people living with HIV and multimorbidity in Toronto, Canada: a qualitative study. BMJ Open. 2016;6(3):e010029. doi: 10.1136/bmjopen-2015-010029 26956163 PMC4785327

[66] Huai P, Xun H, Reilly KH, Wang Y, Ma W, Xi B. Physical activity and risk of hypertension: a meta-analysis of prospective cohort studies. Hypertension. 2013;62(6):1021–6. Epub 2013/10/02. doi: 10.1161/HYPERTENSIONAHA.113.01965 24082054

[67] Ghadieh AS, Saab B. Evidence for exercise training in the management of hypertension in adults. Canadian family physician Medecin de famille canadien. 2015;61(3):233–9. Epub 2015/05/01. 25927108 PMC4369613

[68] Dimeo F, Pagonas N, Seibert F, Arndt R, Zidek W, Westhoff TH. Aerobic exercise reduces blood pressure in resistant hypertension. Hypertension. 2012;60(3):653–8. Epub 2012/07/18. doi: 10.1161/HYPERTENSIONAHA.112.197780 22802220

[69] Cade WT, Reeds DN, Mondy KE, Overton ET, Grassino J, Tucker S, et al. Yoga lifestyle intervention reduces blood pressure in HIV-infected adults with cardiovascular disease risk factors. HIV Med. 2010;11(6):379–88. Epub 2010/01/12. doi: 10.1111/j.1468-1293.2009.00801.x 20059570 PMC2889007

[70] Cornelissen VA, Smart NA. Exercise training for blood pressure: a systematic review and meta-analysis. J Am Heart Assoc. 2013;2(1):e004473. Epub 2013/03/26. doi: 10.1161/JAHA.112.004473 23525435 PMC3603230

[71] Lewington S, Clarke R, Qizilbash N, Peto R, Collins R, Prospective Studies C. Age-specific relevance of usual blood pressure to vascular mortality: a meta-analysis of individual data for one million adults in 61 prospective studies. Lancet. 2002;360(9349):1903–13. Epub 2002/12/21. doi: 10.1016/s0140-6736(02)11911-8 12493255

[72] Ettehad D, Emdin CA, Kiran A, Anderson SG, Callender T, Emberson J, et al. Blood pressure lowering for prevention of cardiovascular disease and death: a systematic review and meta-analysis. Lancet. 2016;387(10022):957–67. Epub 2016/01/03. doi: 10.1016/S0140-6736(15)01225-8 26724178

[73] Vancampfort D, Mugisha J, Richards J, De Hert M, Lazzarotto AR, Schuch FB, et al. Dropout from physical activity interventions in people living with HIV: a systematic review and meta-analysis. AIDS Care. 2017;29(5):636–43. Epub 2016/11/01. doi: 10.1080/09540121.2016.1248347 27794625

[74] Boucher LM, Liddy C, Mihan A, Kendall C. Peer-led Self-management Interventions and Adherence to Antiretroviral Therapy Among People Living with HIV: A Systematic Review. AIDS Behav. 2019. Epub 2019/10/11. 10.1007/s10461-019-02690-7 31598801

[75] Boucher LM, O’Brien KK, Baxter LN, Fitzgerald ML, Liddy CE, Kendall CE. Healthy aging with HIV: The role of self-management support. Patient Educ Couns. 2019;102(8):1565–9. Epub 2019/03/05. doi: 10.1016/j.pec.2019.02.019 30827568

[76] Ramsay CR, Matowe L, Grilli R, Grimshaw JM, Thomas RE. Interrupted time series designs in health technology assessment: lessons from two systematic reviews of behavior change strategies. Int J Technol Assess Health Care. 2003;19(4):613–23. Epub 2004/04/21. doi: 10.1017/s0266462303000576 15095767

[77] Solomon P, Carusone SC, Davis AM, Aubry R, O’Brien KK. Experiences of People Living With HIV in Community Based Exercise: A Qualitative Longitudinal Study. Journal of the International Association of Providers of AIDS Care. 2021;20: Epub 2021/02/23. doi: 10.1177/2325958221995344 33611978 PMC7903824

[78] Li G, Taljaard M, Van den Heuvel ER, Levine MA, Cook DJ, Wells GA, et al. An introduction to multiplicity issues in clinical trials: the what, why, when and how. International journal of epidemiology. 2017;46(2):746–55. Epub 2016/12/28. doi: 10.1093/ije/dyw320 28025257

[79] Webel AR, Barkley J, Longenecker CT, Mittelsteadt A, Gripshover B, Salata RA. A cross-sectional description of age and gender differences in exercise patterns in adults living with HIV. J Assoc Nurses AIDS Care. 2015;26(2):176–86. Epub 2014/09/25. doi: 10.1016/j.jana.2014.06.004 25249267 PMC4284137

[80] Vancampfort D, Mugisha J, Richards J, De Hert M, Probst M, Stubbs B. Physical activity correlates in people living with HIV/AIDS: a systematic review of 45 studies. Disabil Rehabil. 2018;40(14):1618–29. Epub 2017/03/23. doi: 10.1080/09638288.2017.1306587 28325087

