# Supplementary material for: Examining the impact of a community-based exercise intervention on cardiorespiratory fitness, cardiovascular health, strength, flexibility and physical activity among adults living with HIV: A three-phased intervention study
Source: PLoS One. 2021 Sep 24;16(9):e0257639. doi: 10.1371/journal.pone.0257639 (PMC8462727; doi:10.1371/journal.pone.0257639)
Supplement: S1 Fig — The line indicates participant-level predictions from the model. Trajectories indicate the fitted values across all three study phases. Red dots indicate V̇O2peak measurements in Phase 1 (baseline monitoring). Green dots indicate V̇O2peak measurements in Phase 2 (intervention). Blue dots indicate V̇O2peak measurements in Phase 3 (follow-up monitoring). (PDF) [file pone.0257639.s001.pdf]

Individual VO2 Peak Trajectories

ID 001

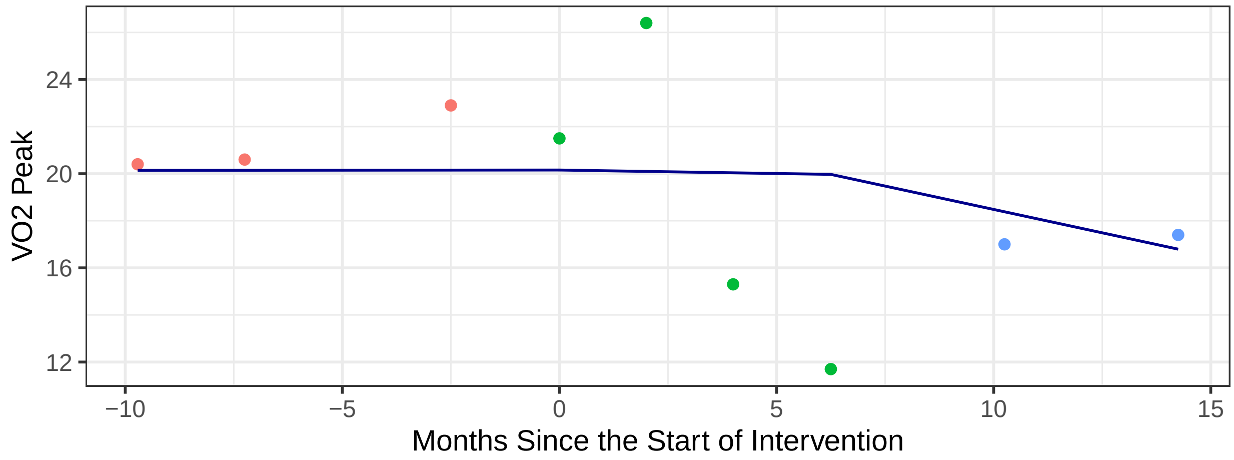

ID 002

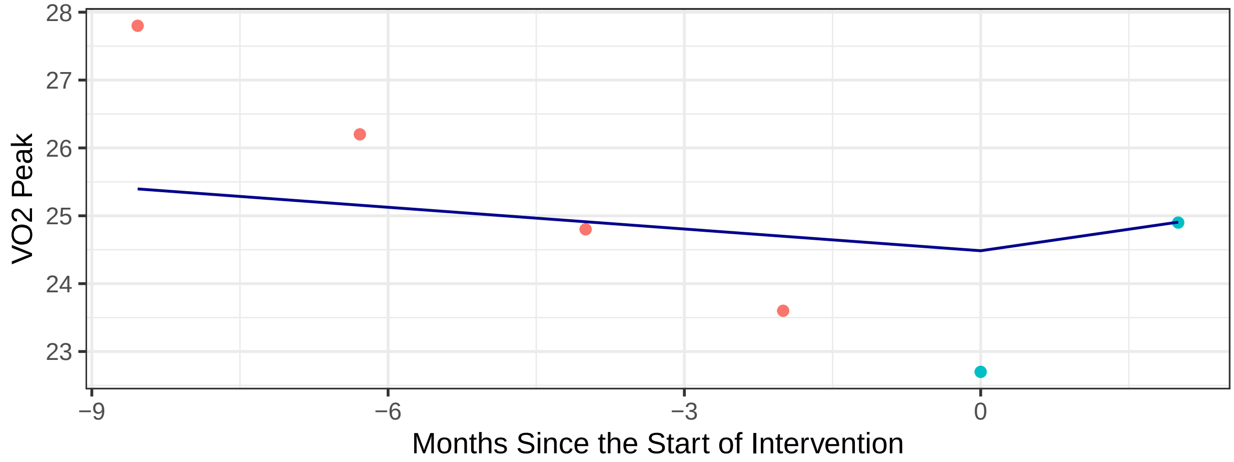

ID 004

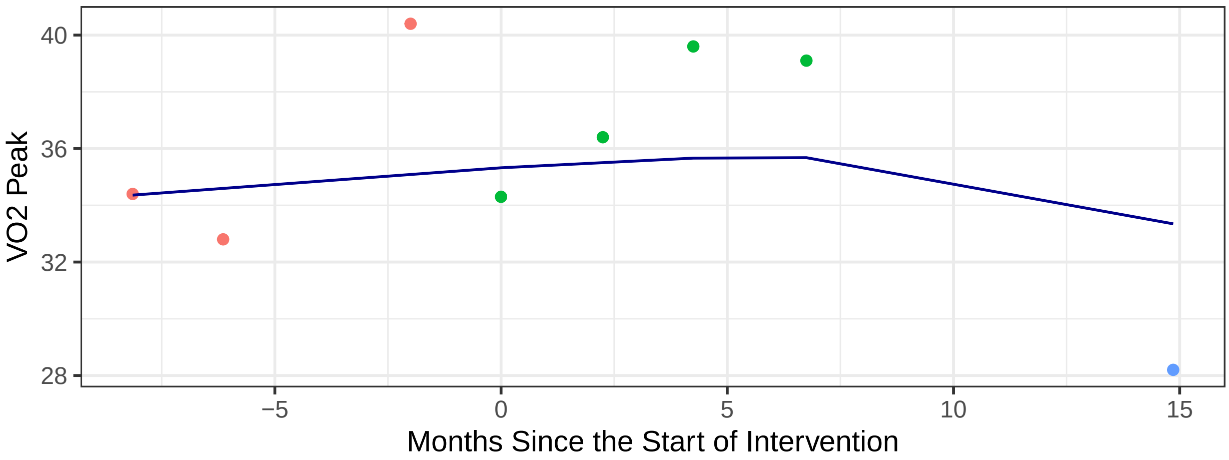

ID 006

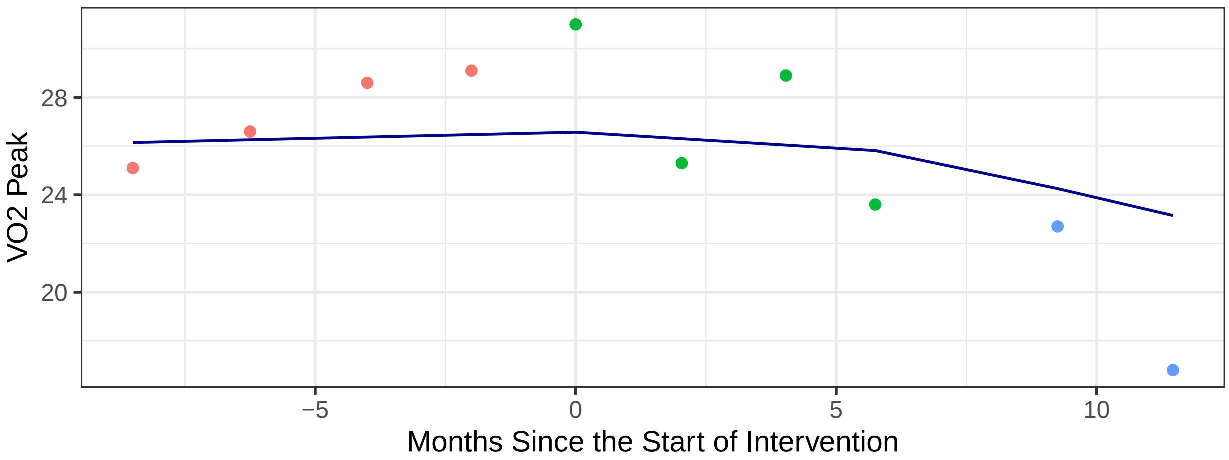

ID 007

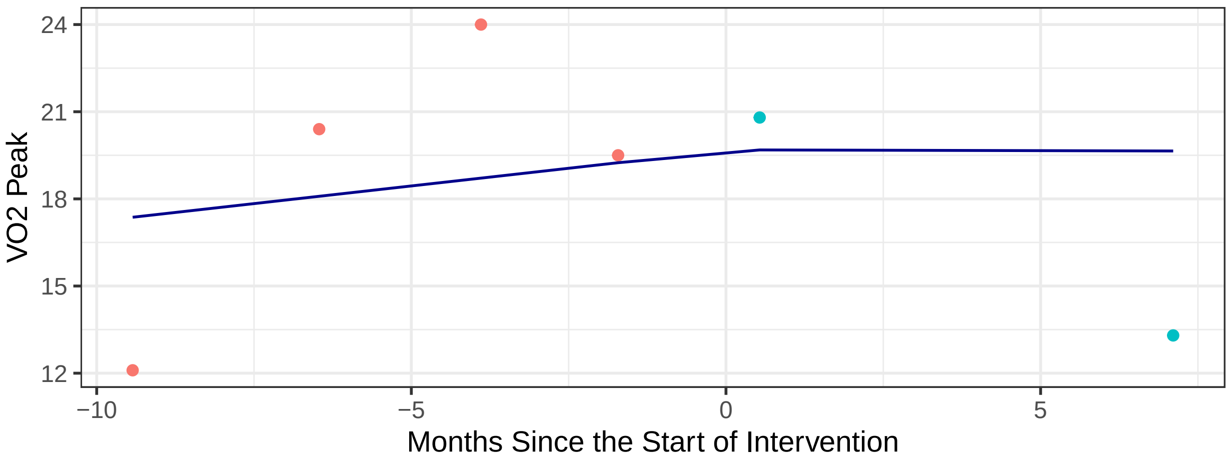

ID 008

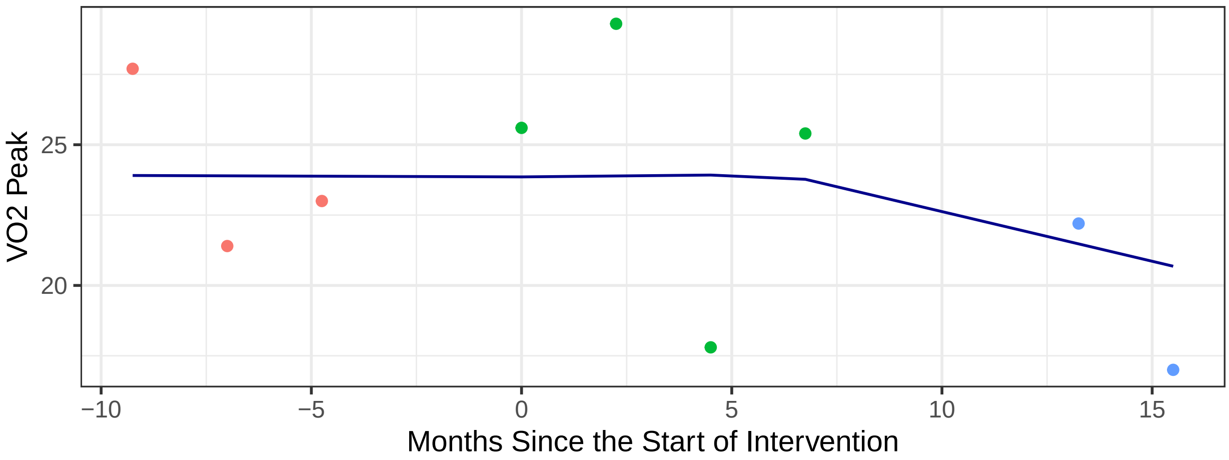

ID 010

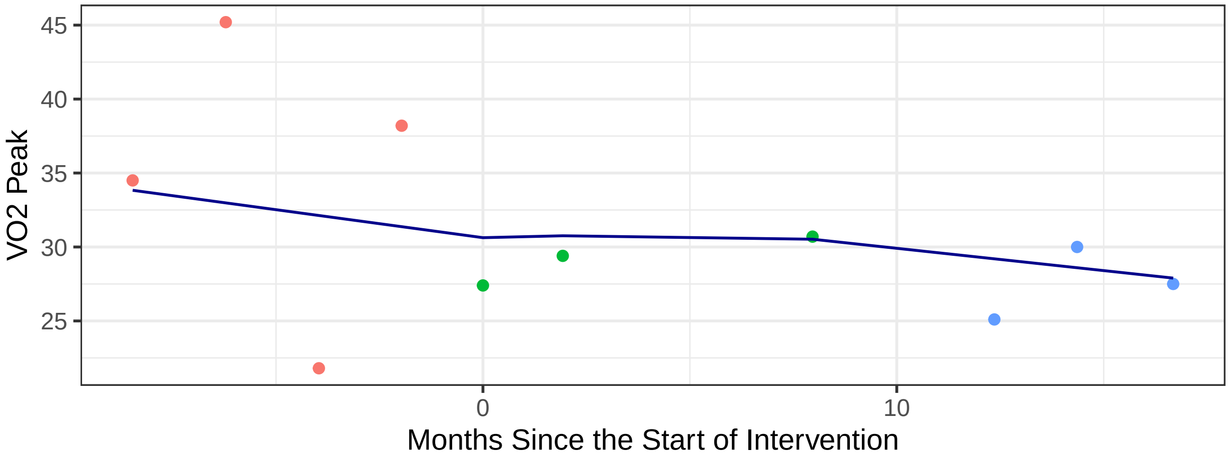

ID 011

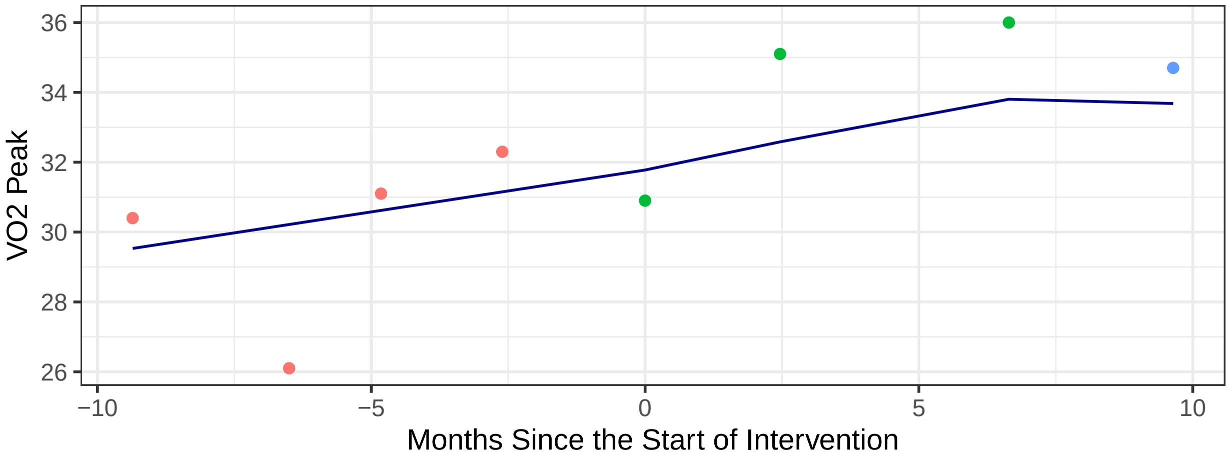

ID 012

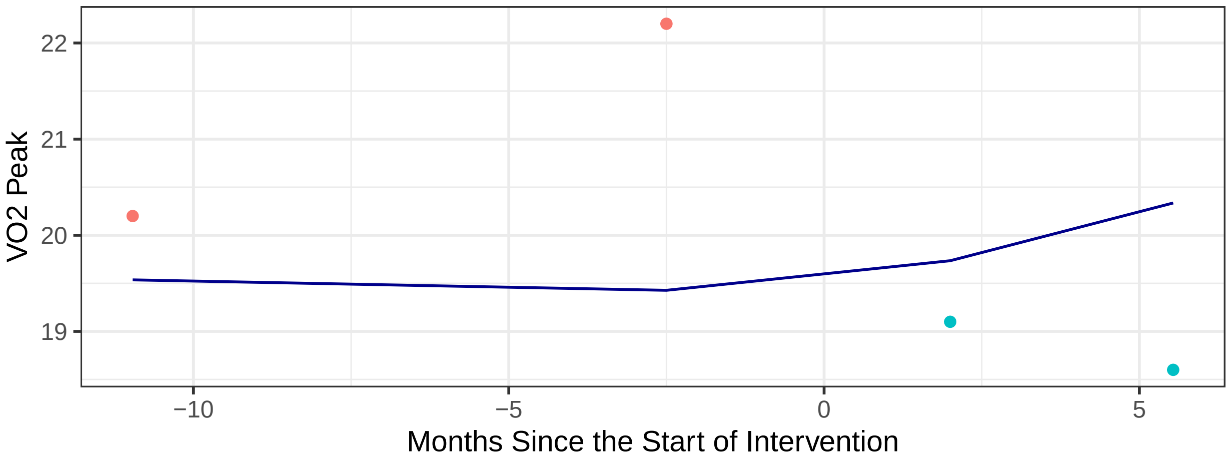

ID 013

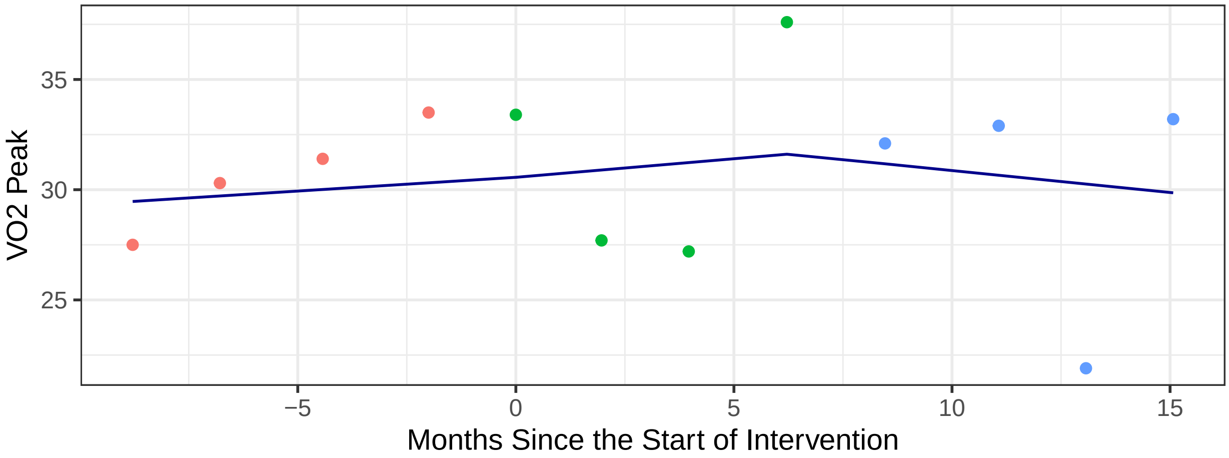

ID 014

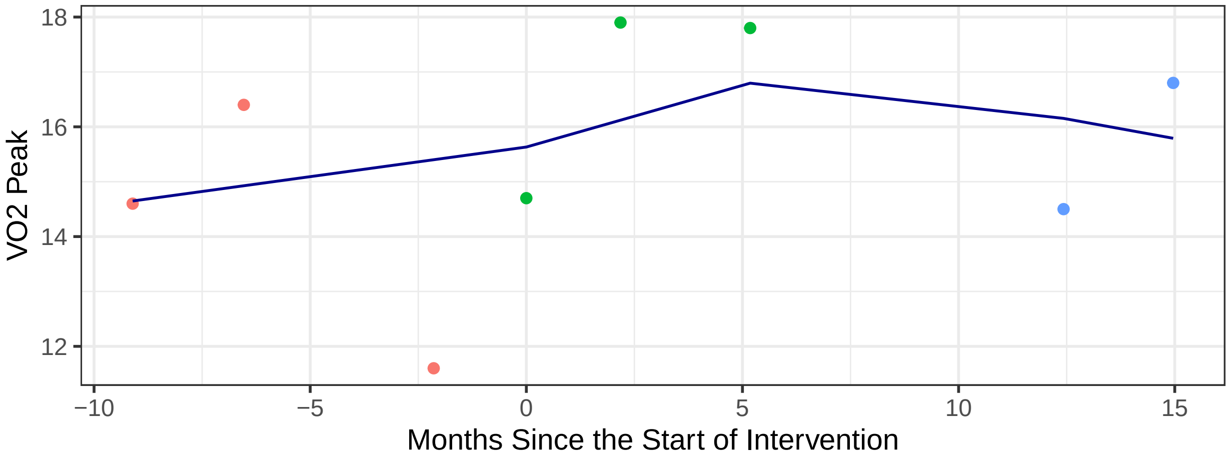

ID 015

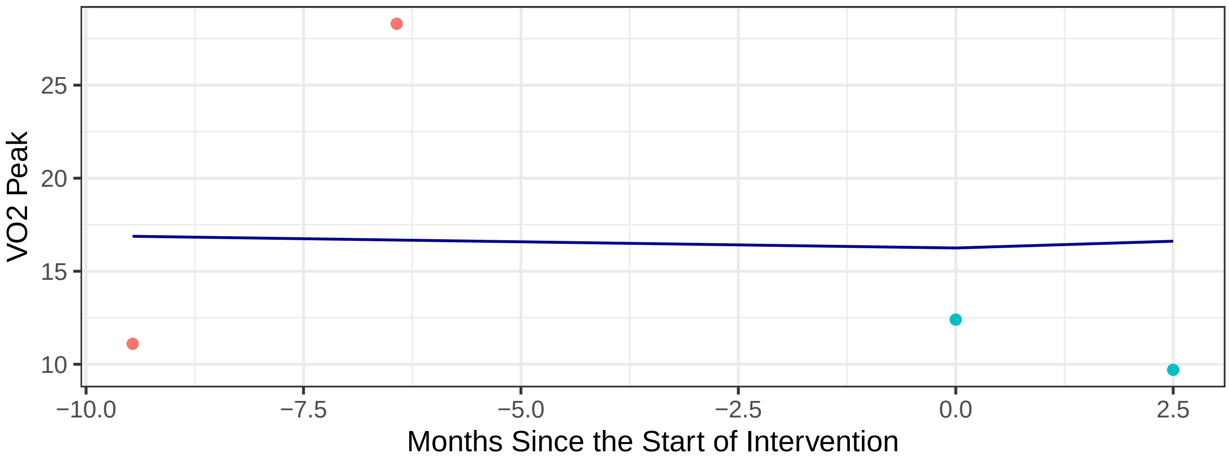

ID 016

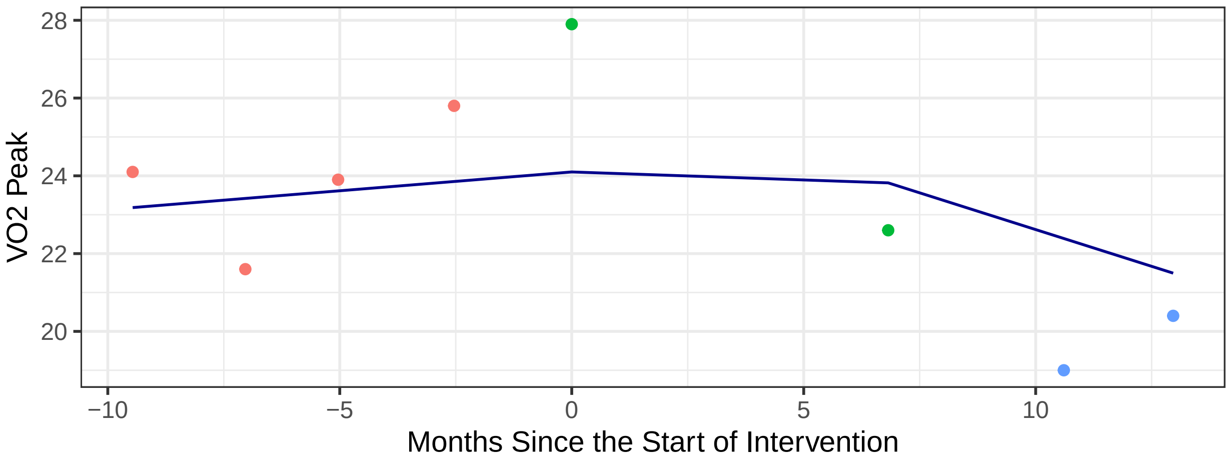

ID 017

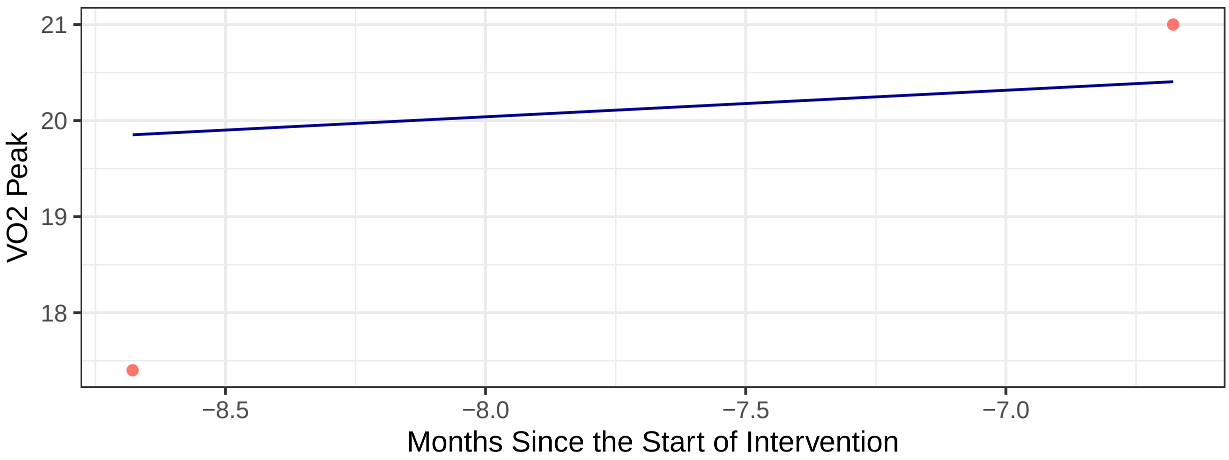

ID 018

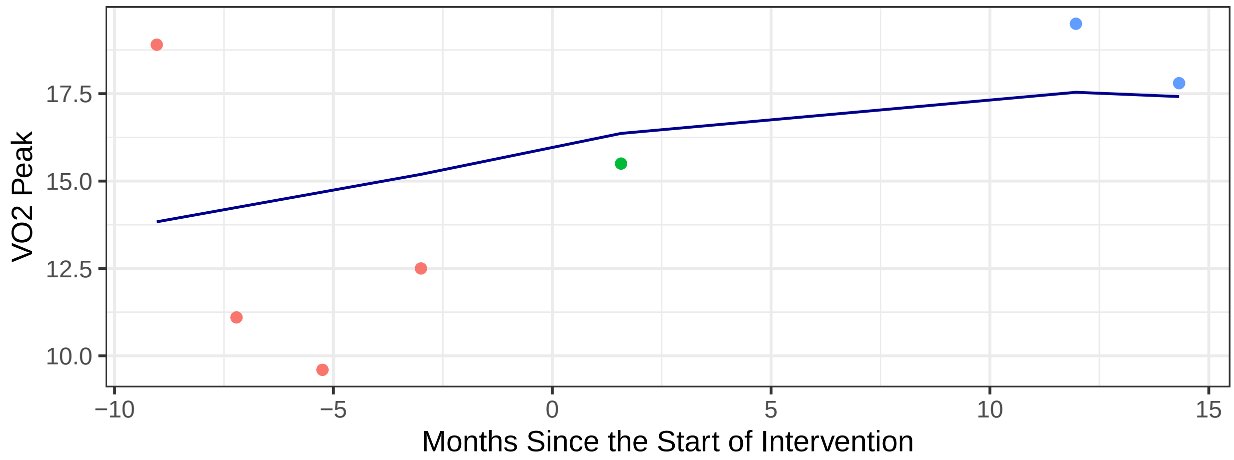

ID 019

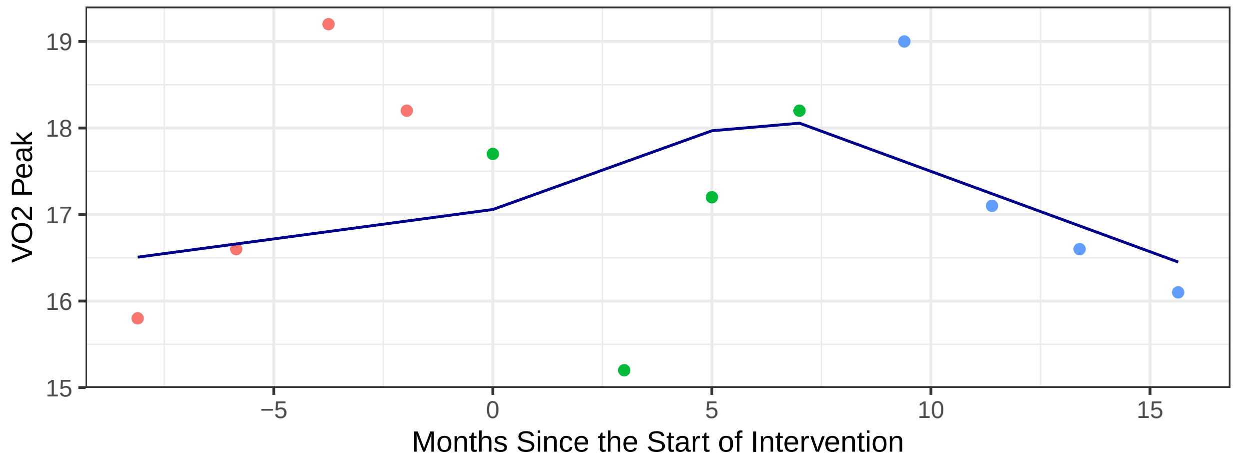

ID 021

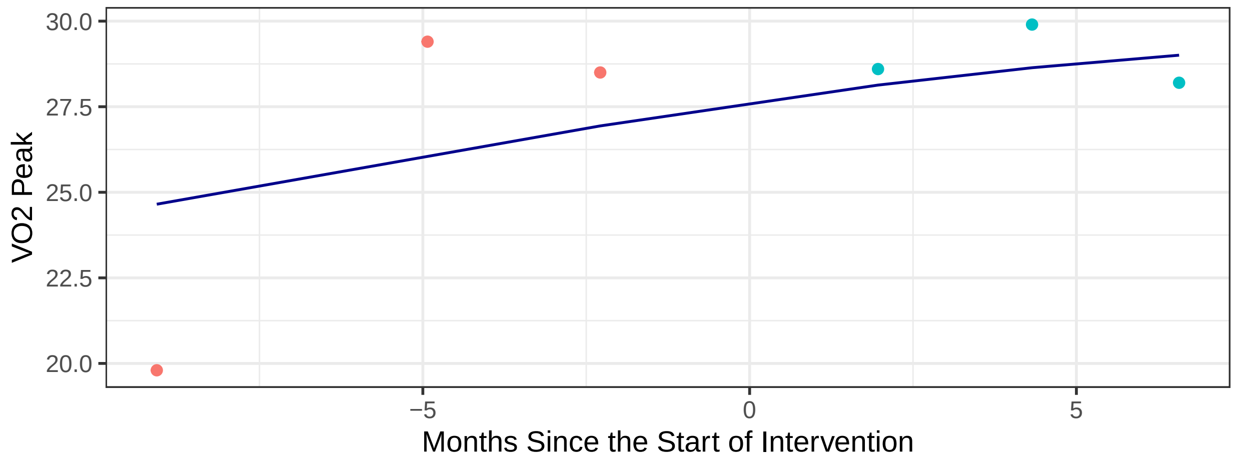

ID 022

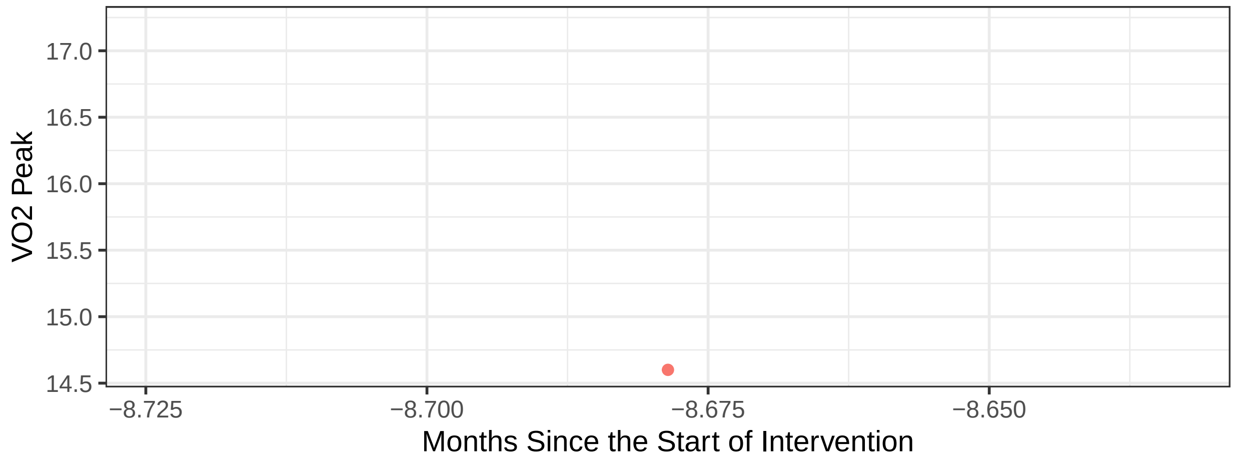

ID 023

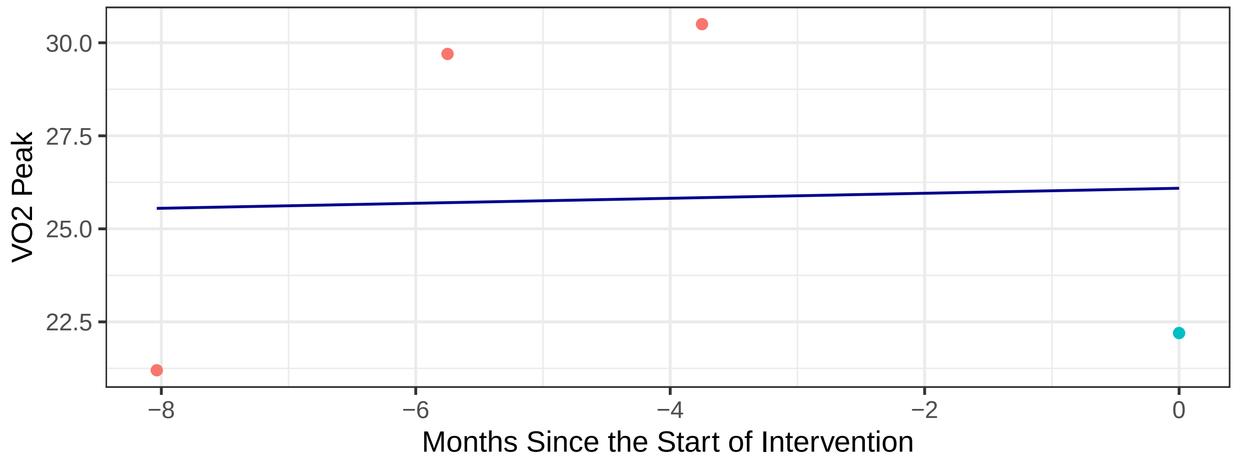

ID 024

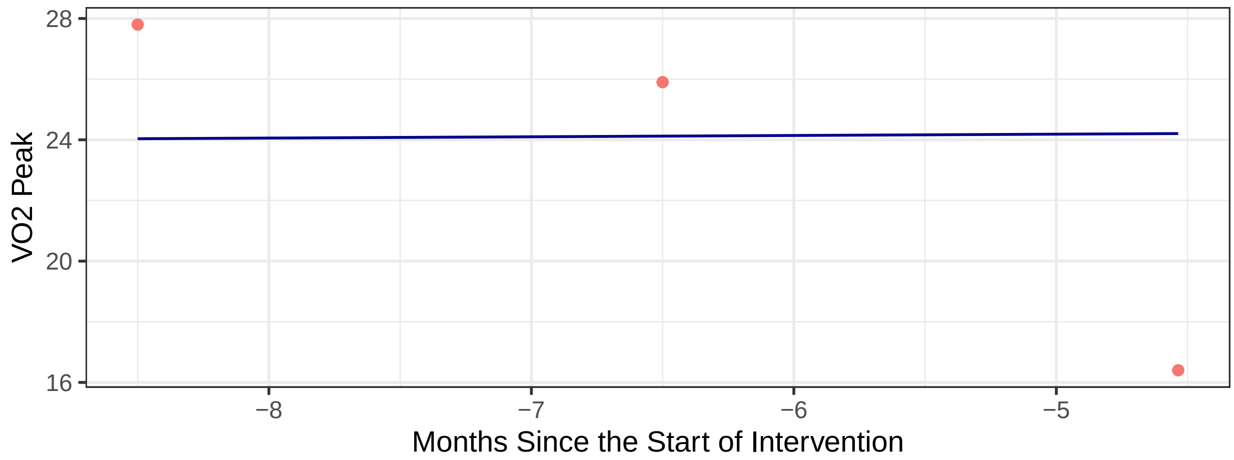

ID 025

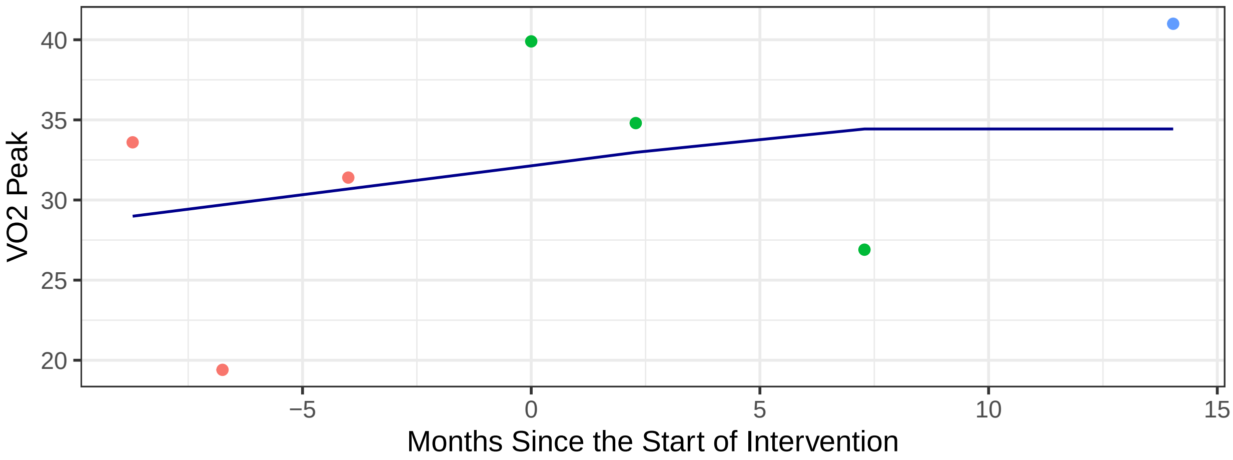

ID 026

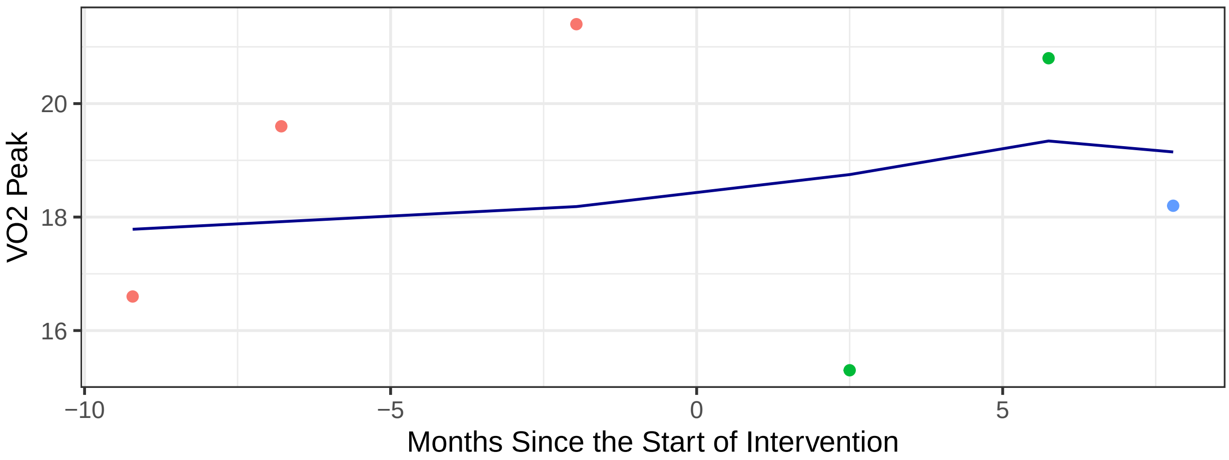

ID 027

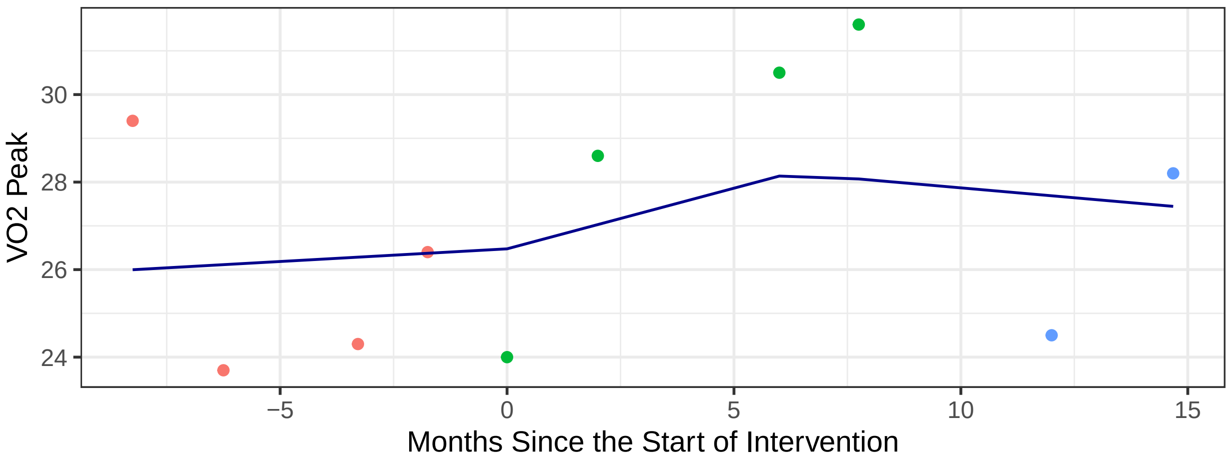

ID 028

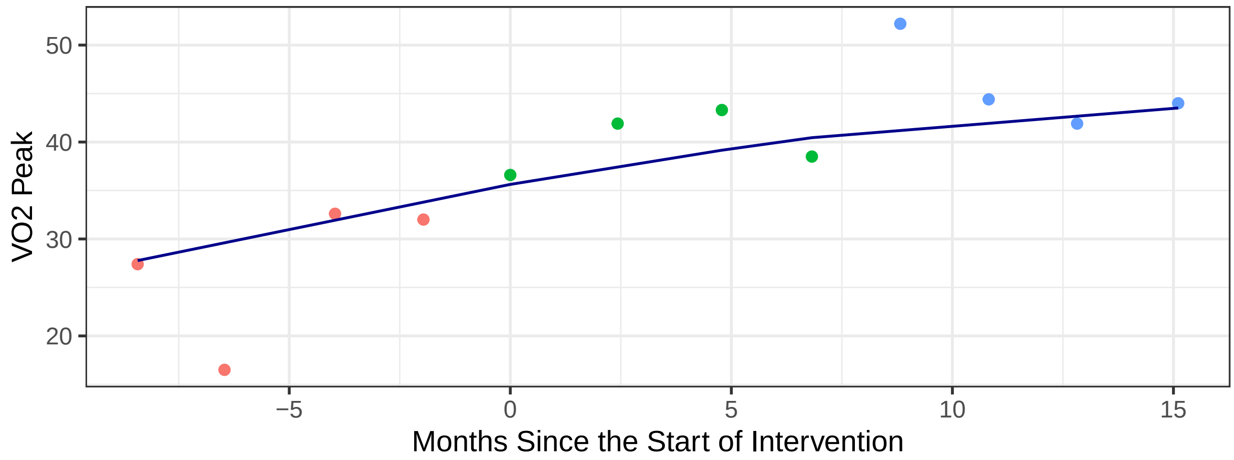

ID 029

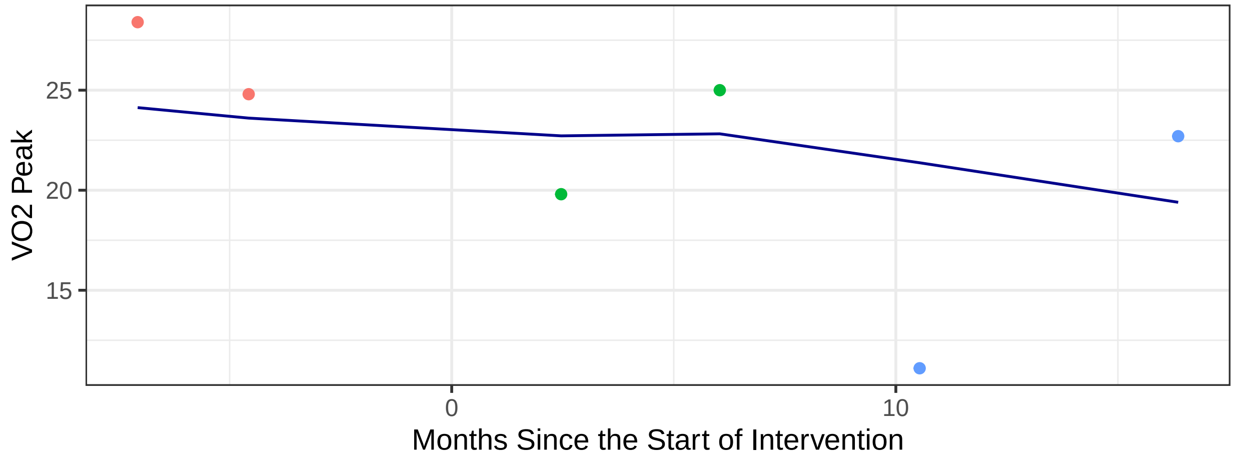

ID 030

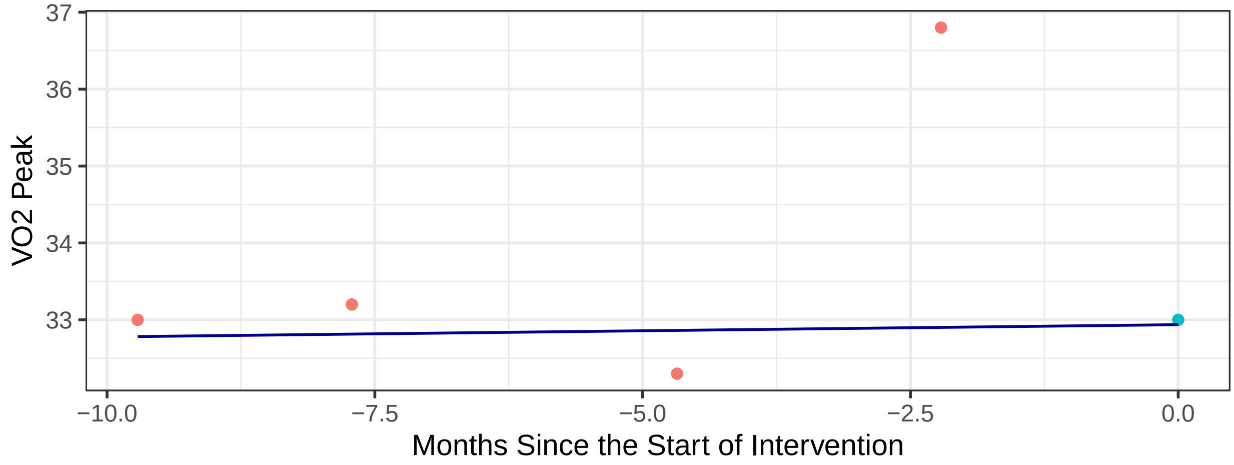

ID 031

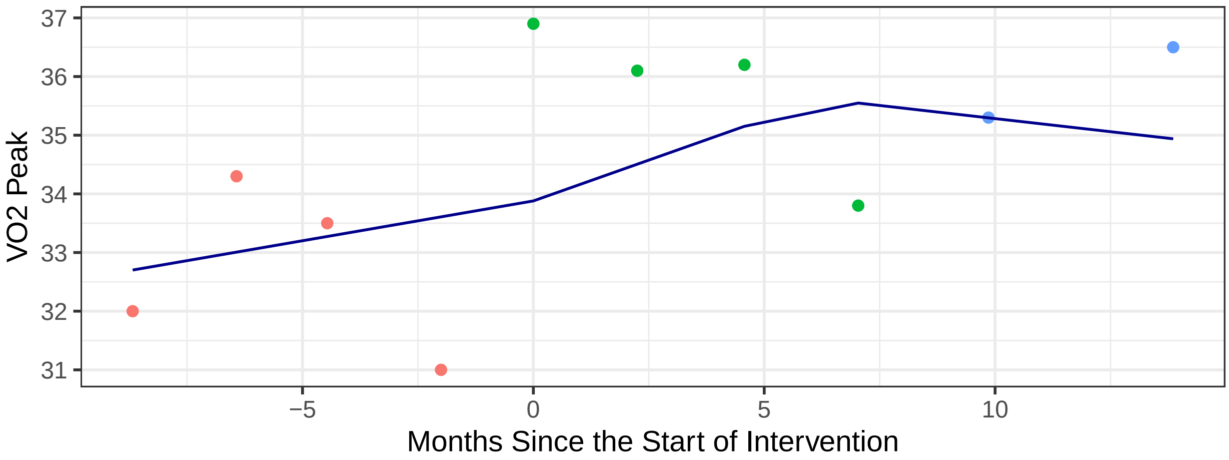

ID 033

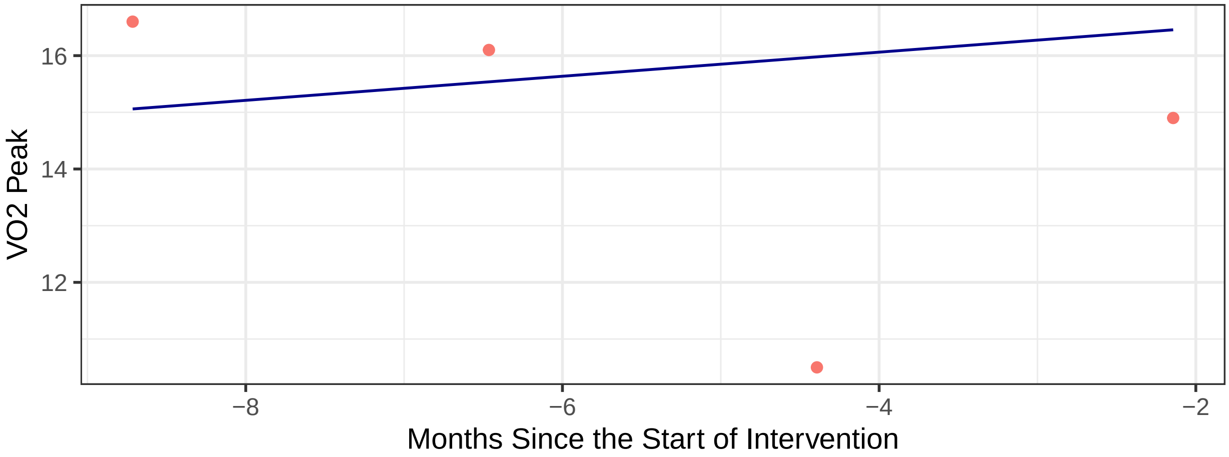

ID 034

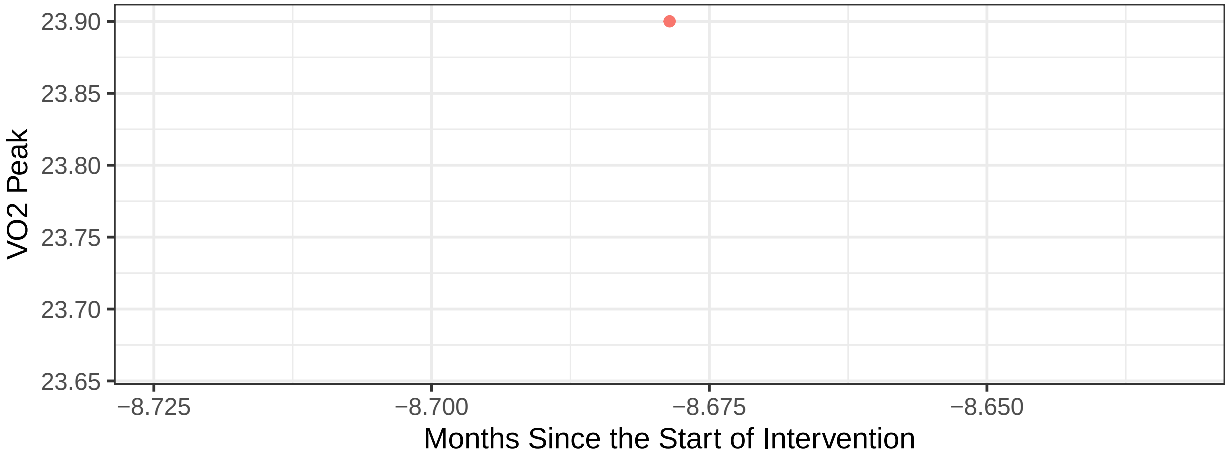

ID 035

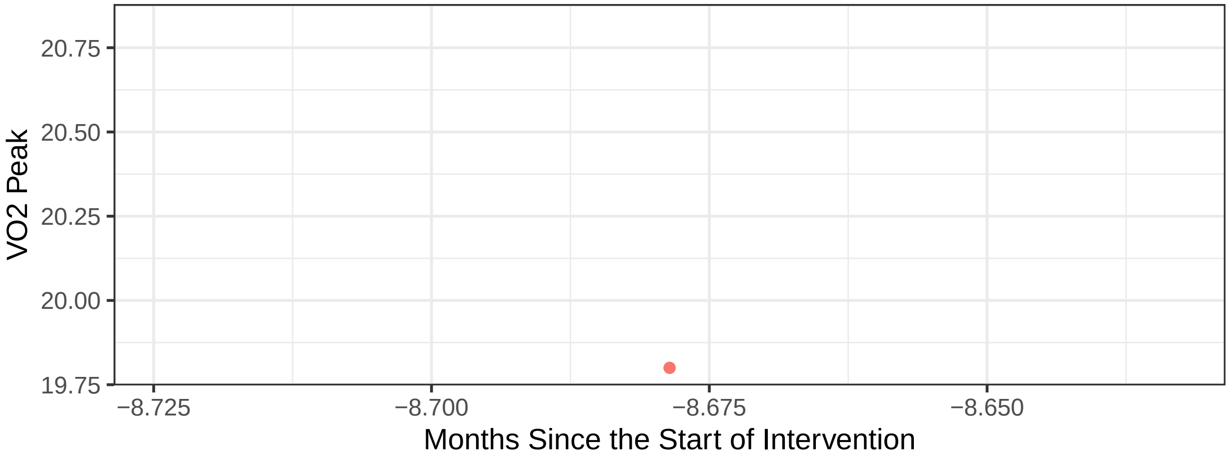

ID 036

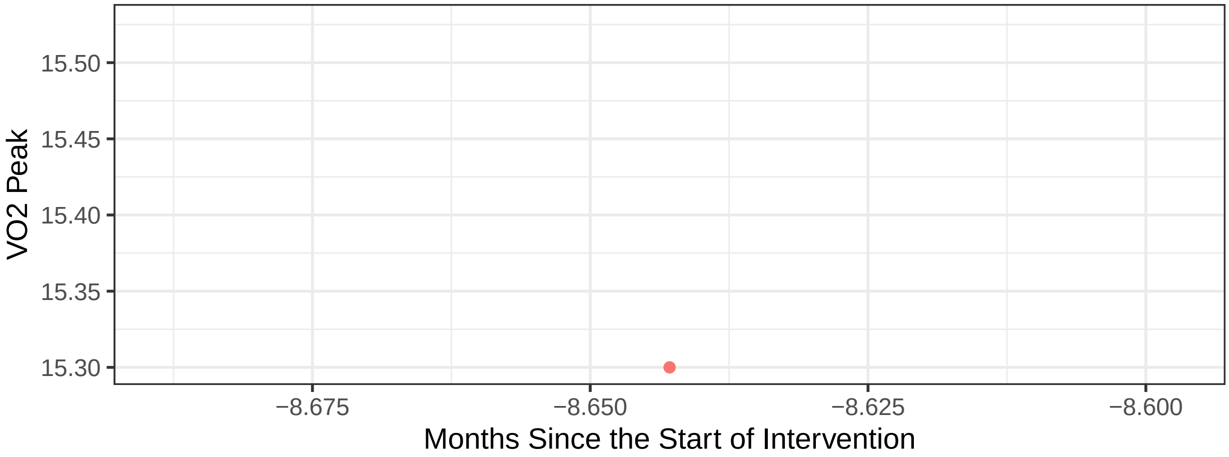

ID 037

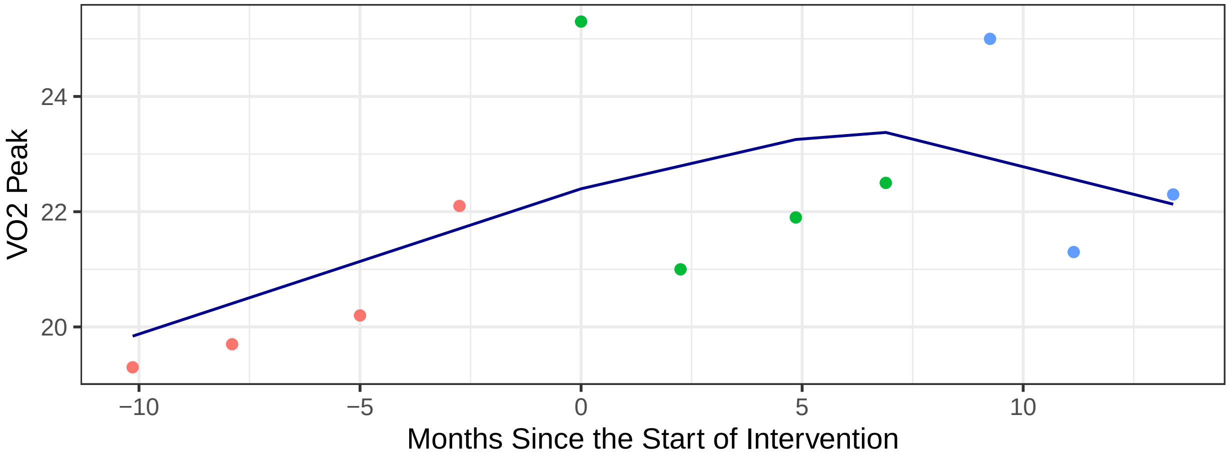

ID 038

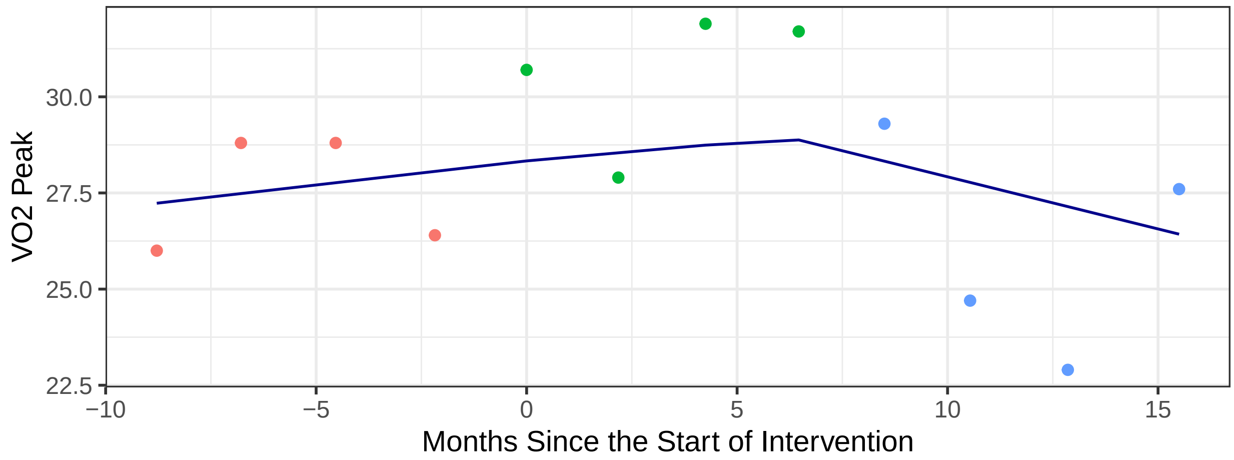

ID 039

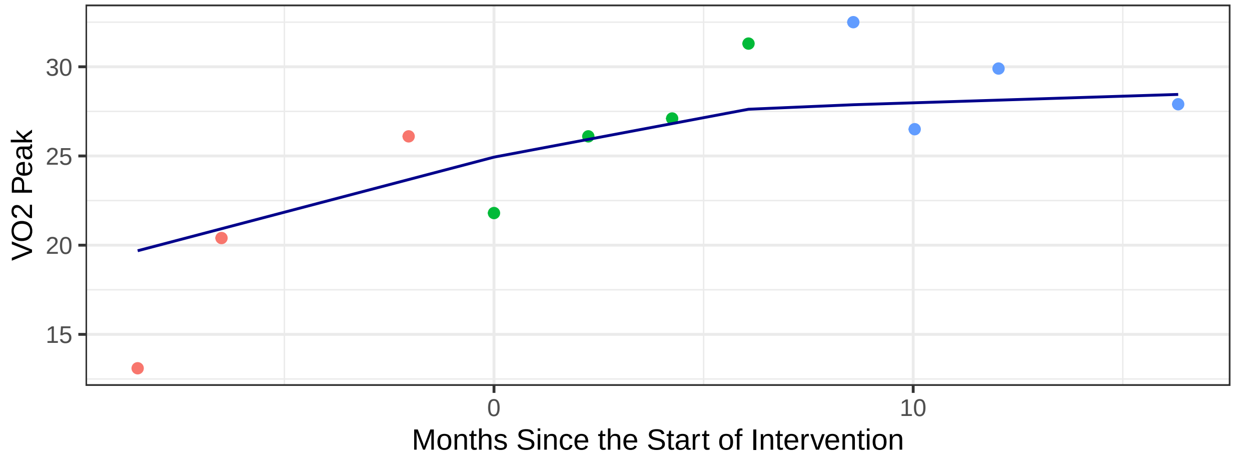

ID 040

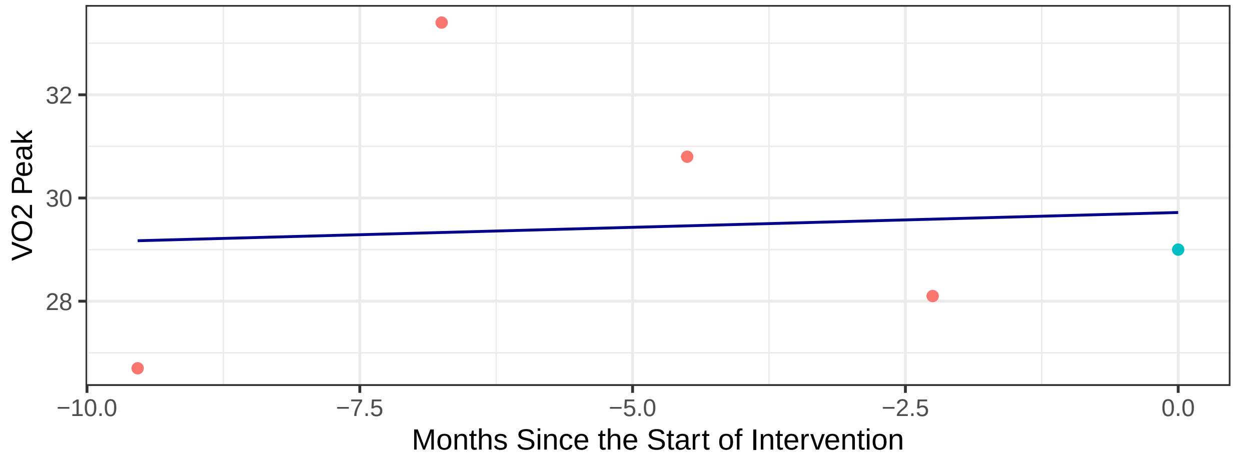

ID 041

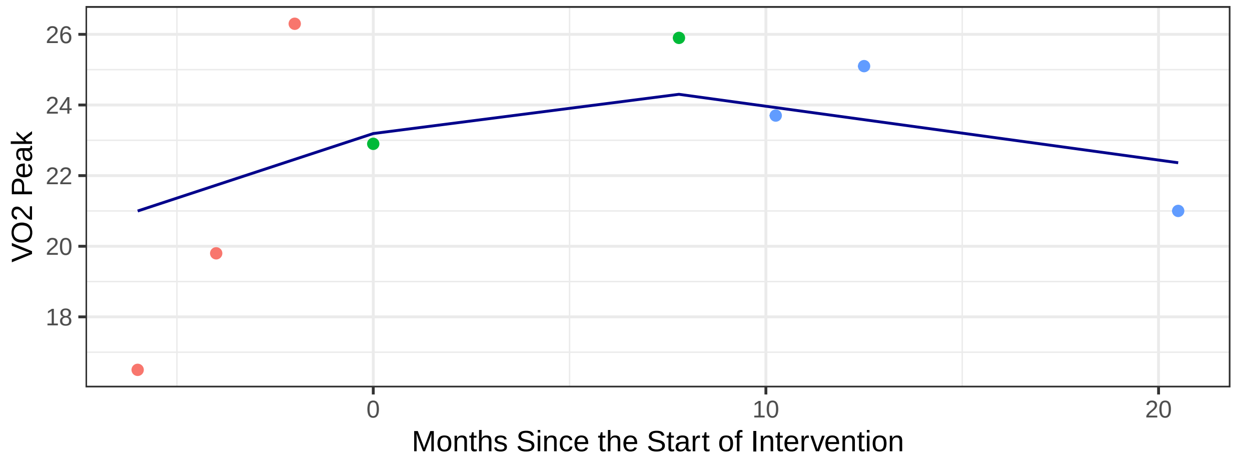

ID 042

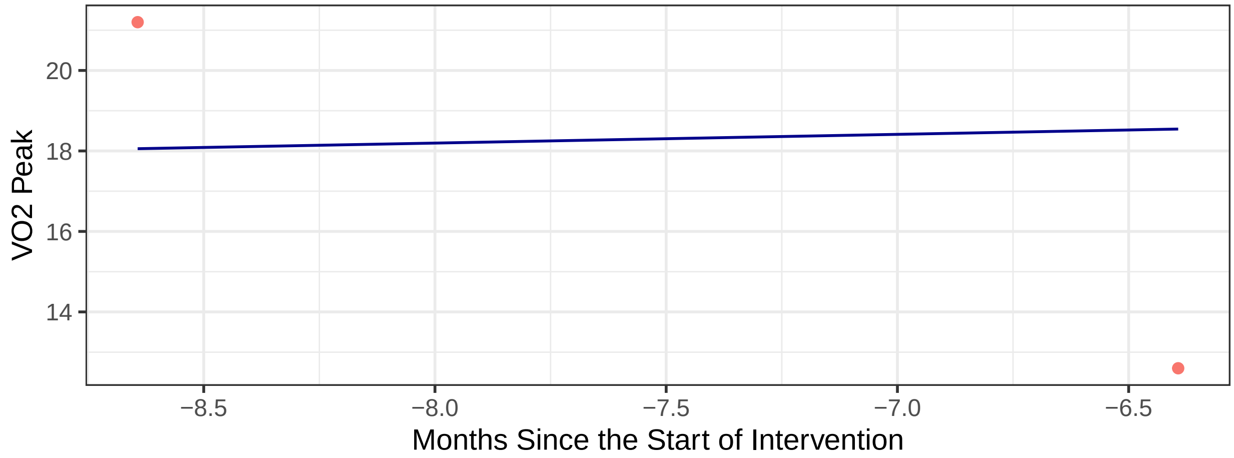

ID 043

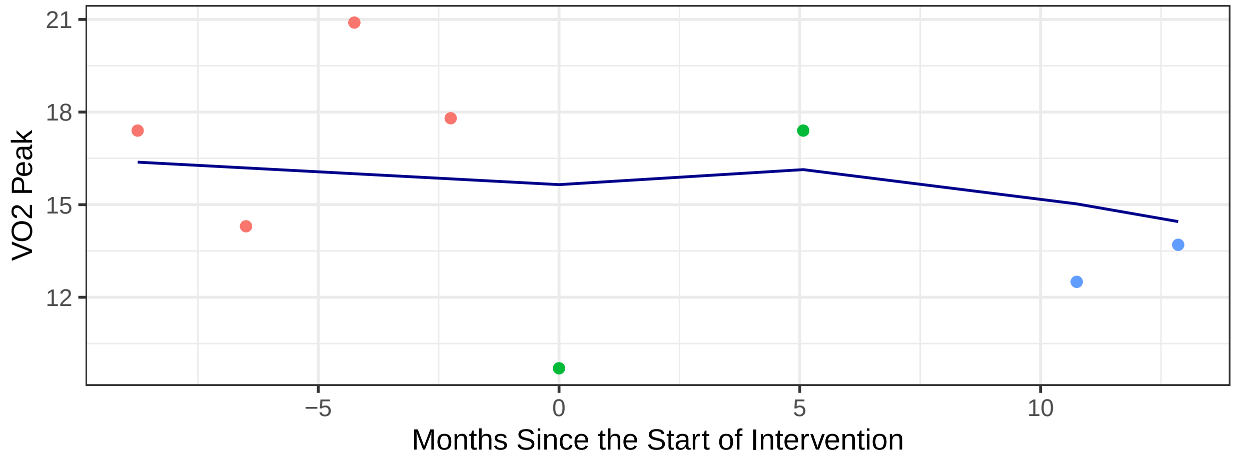

ID 044

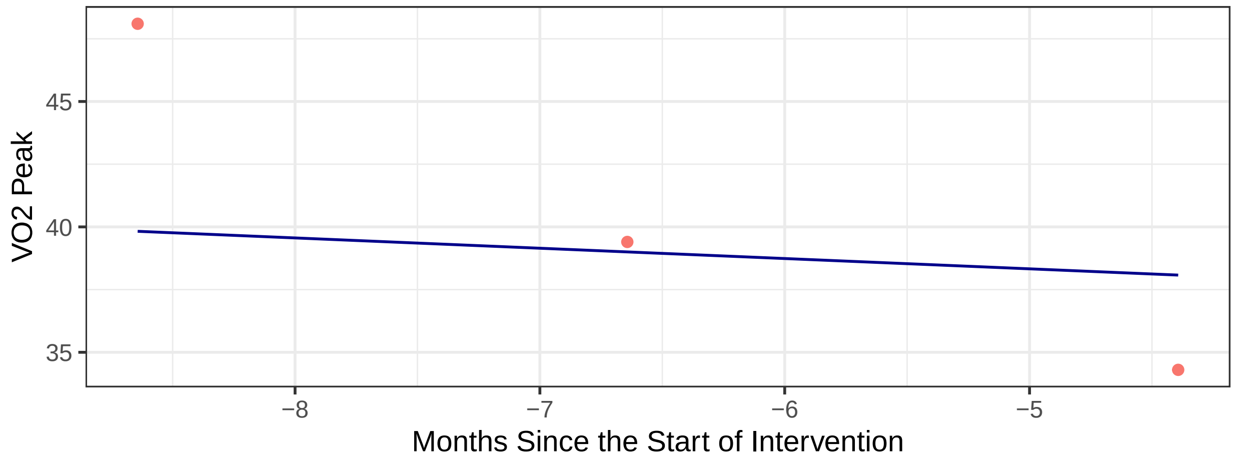

ID 046

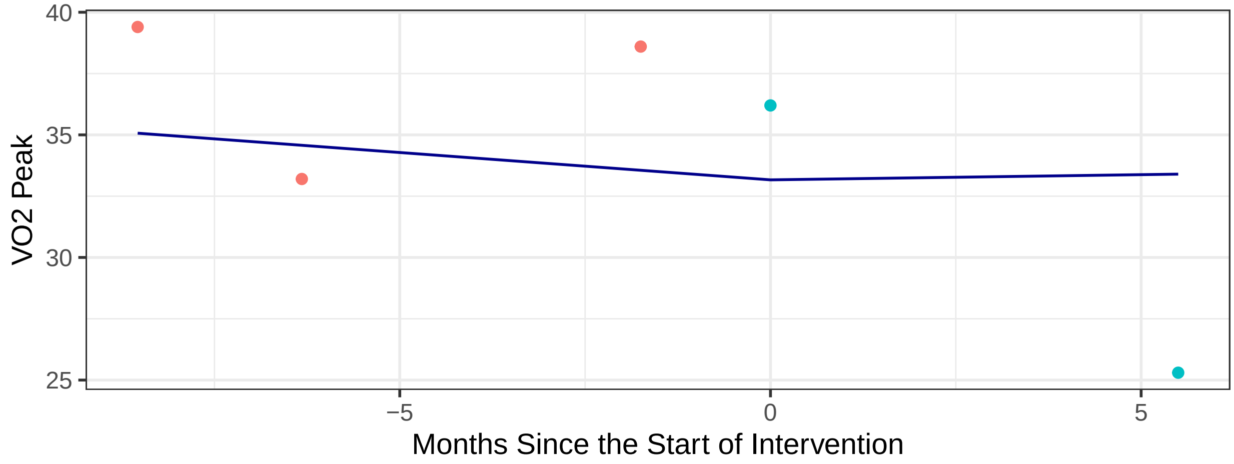

ID 047

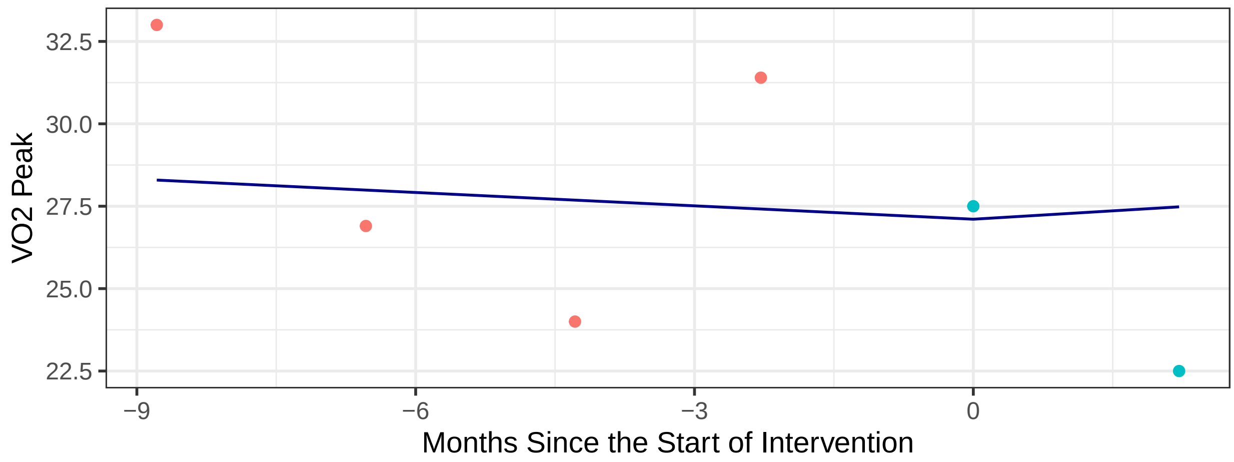

ID 048

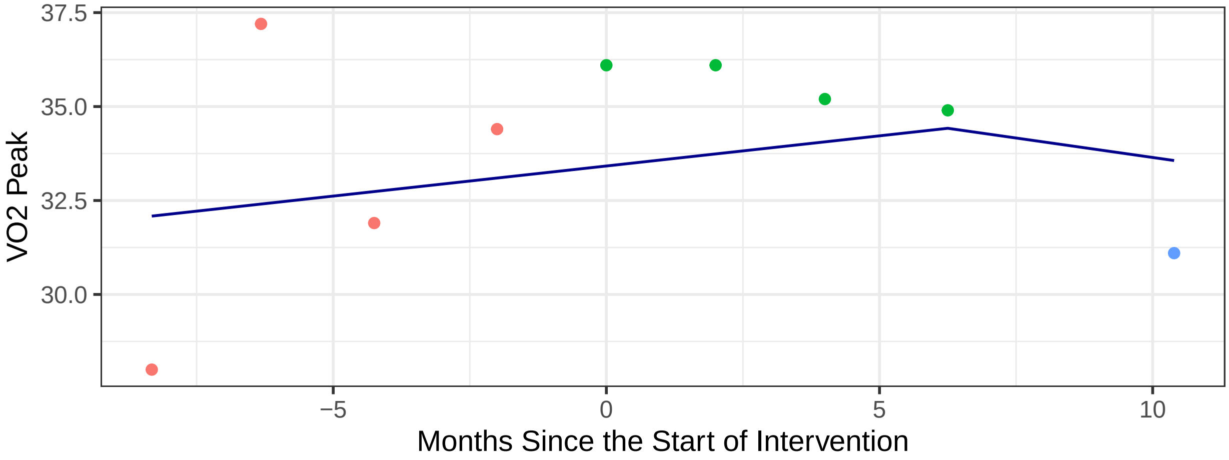

ID 049

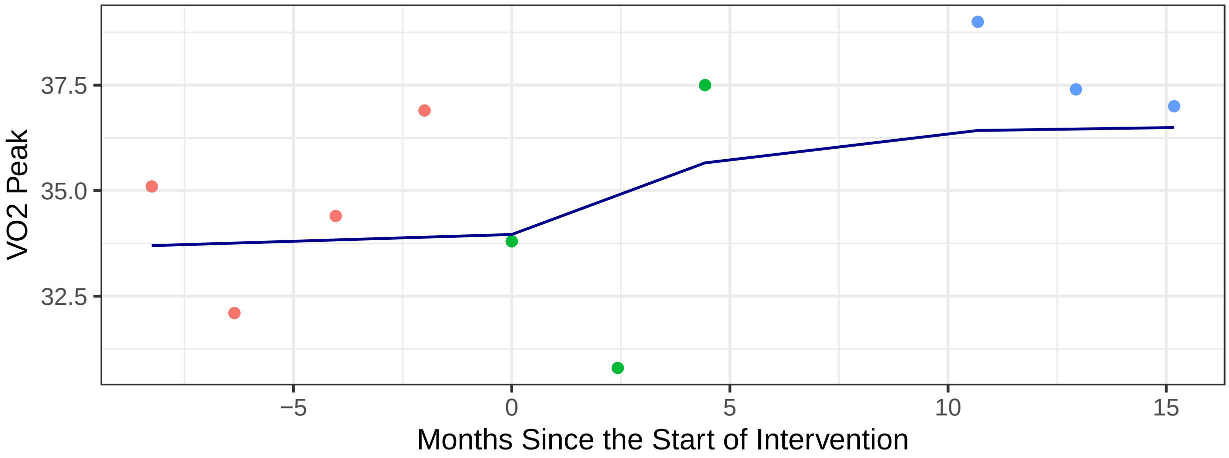

ID 050

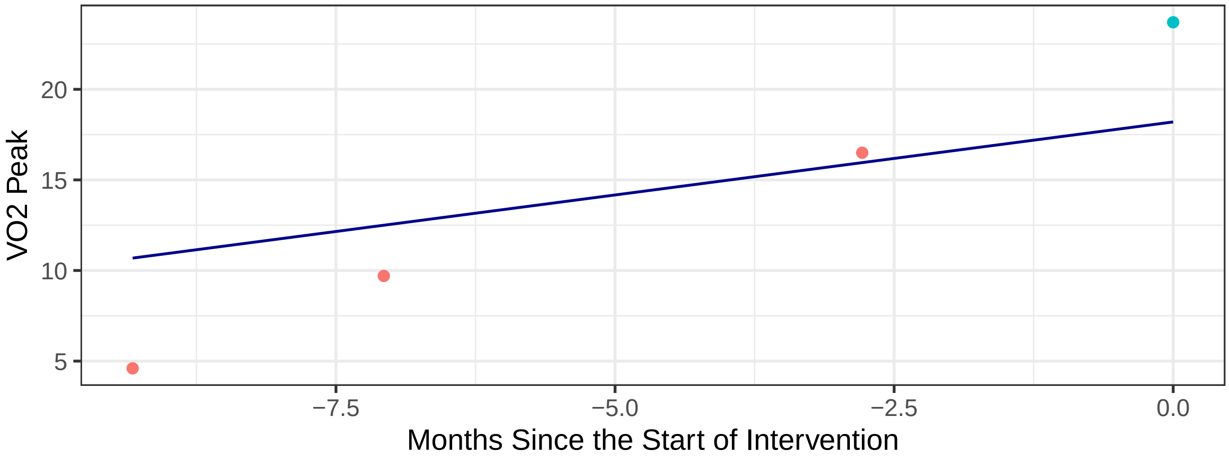

ID 052

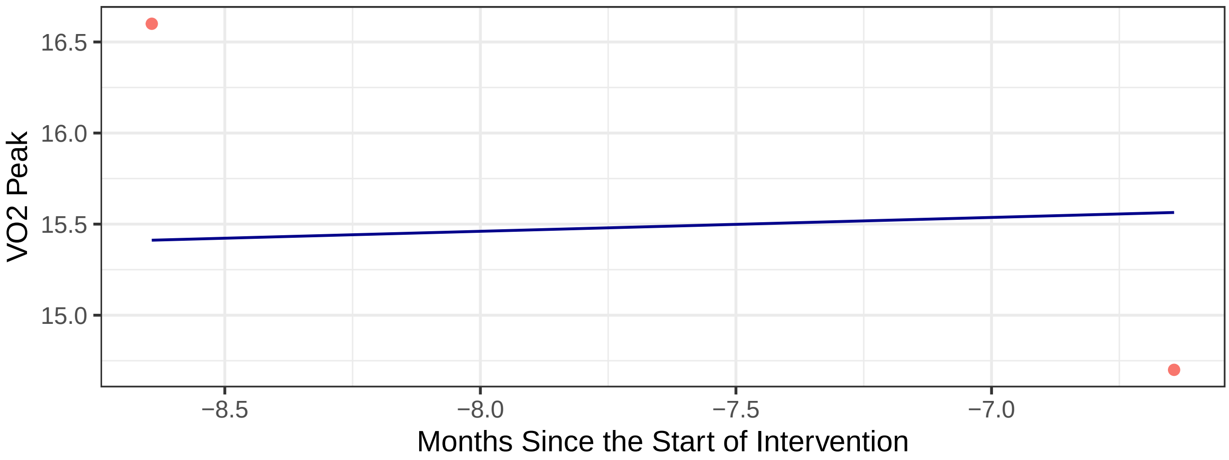

ID 053

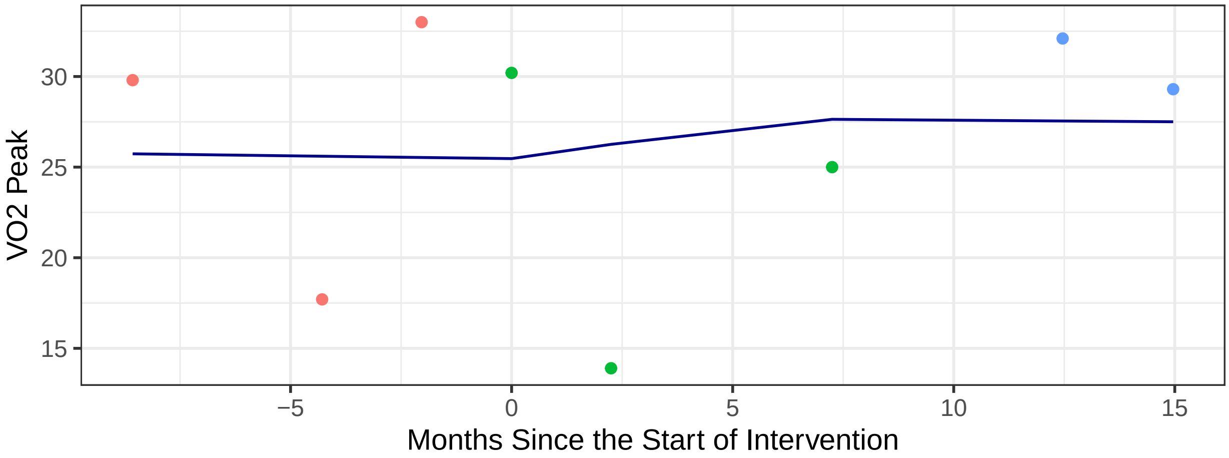

ID 054

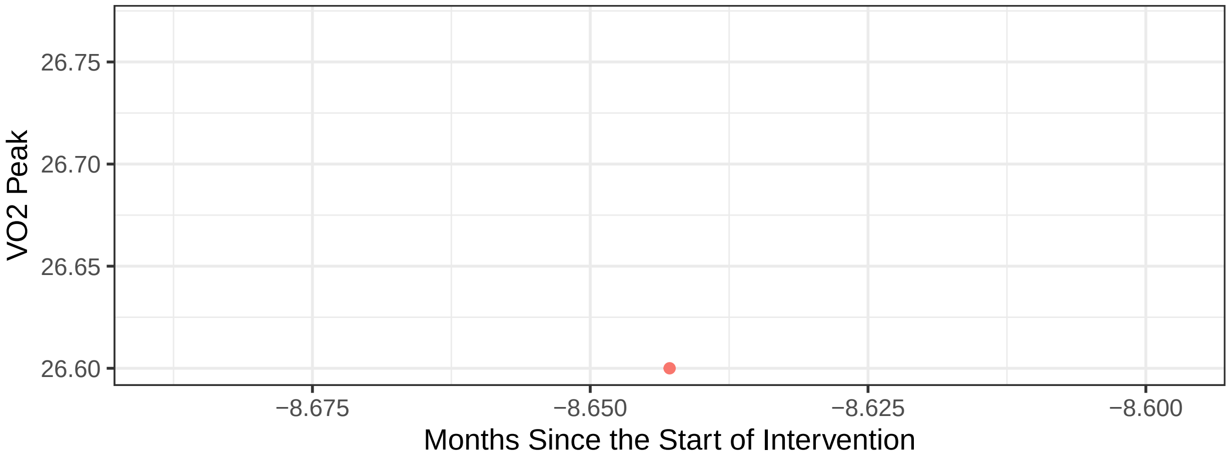

ID 055

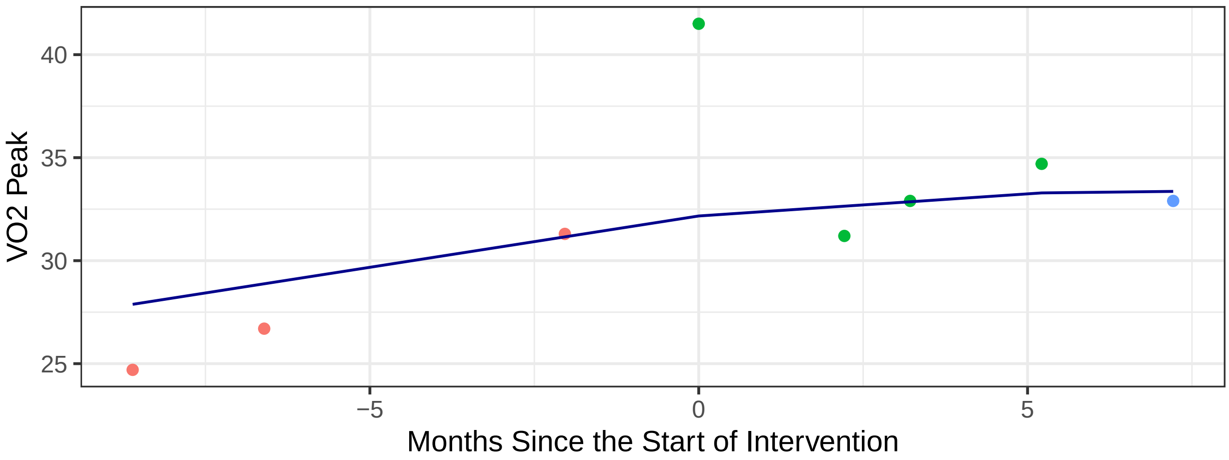

ID 056

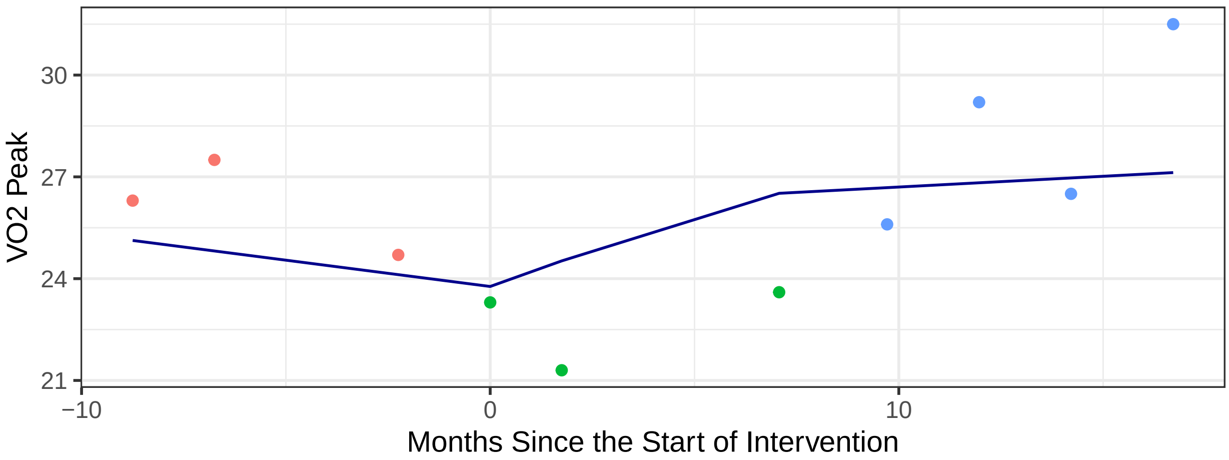

ID 057

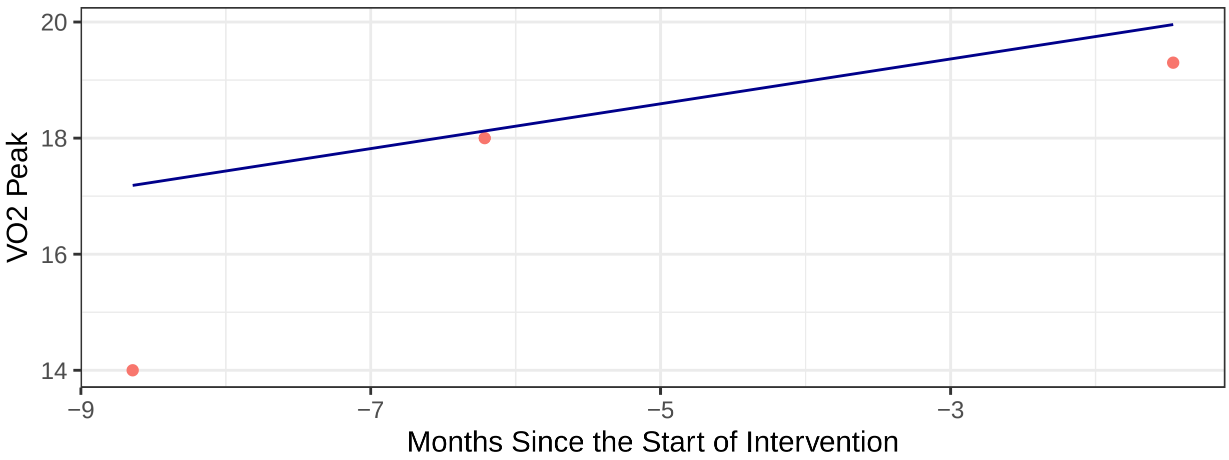

ID 059

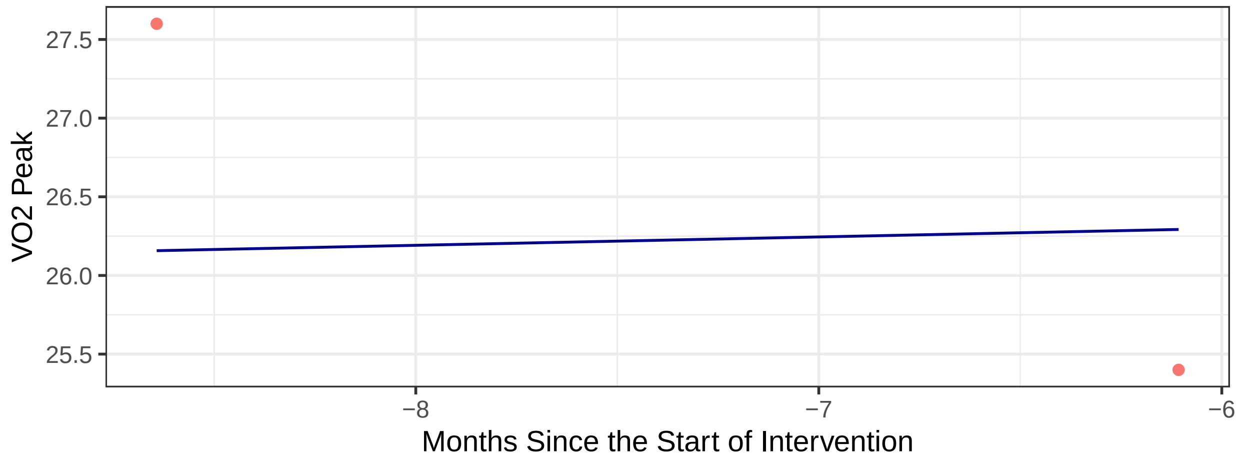

ID 061

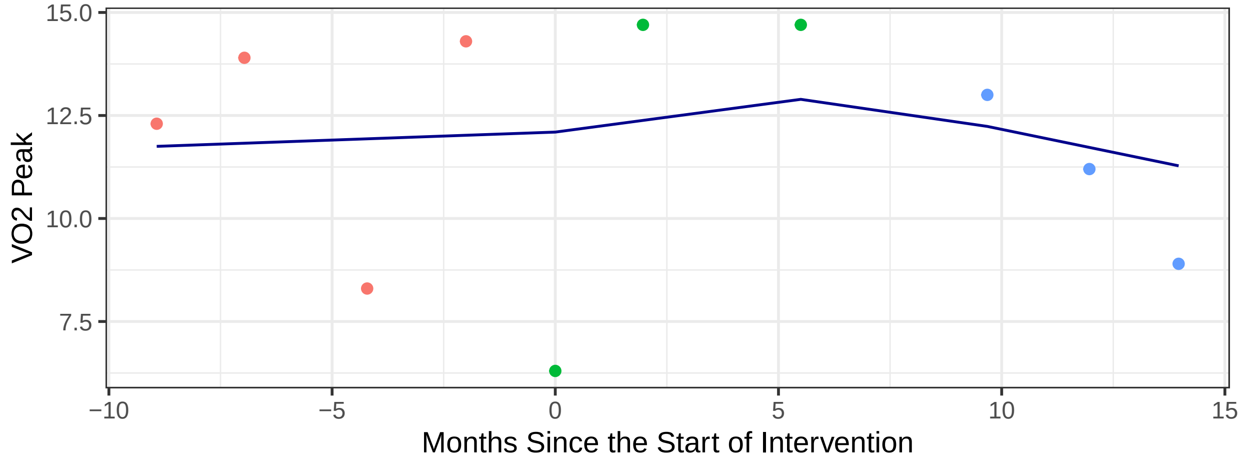

ID 063

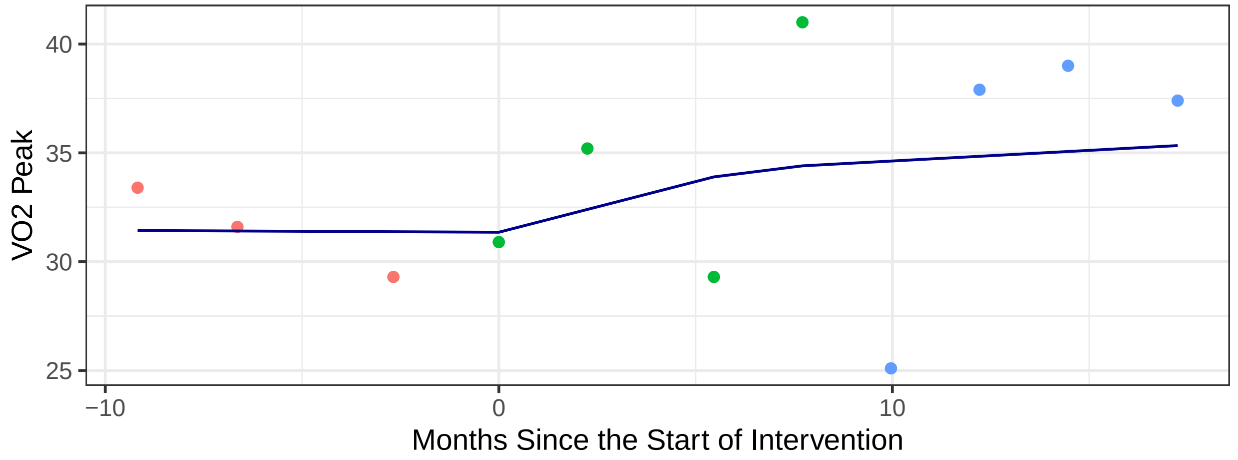

ID 064

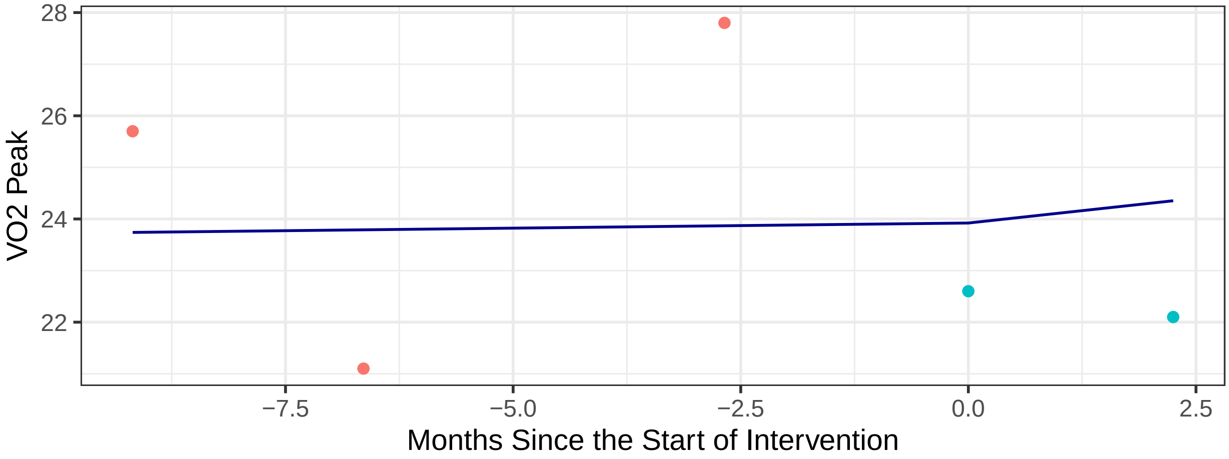

ID 066

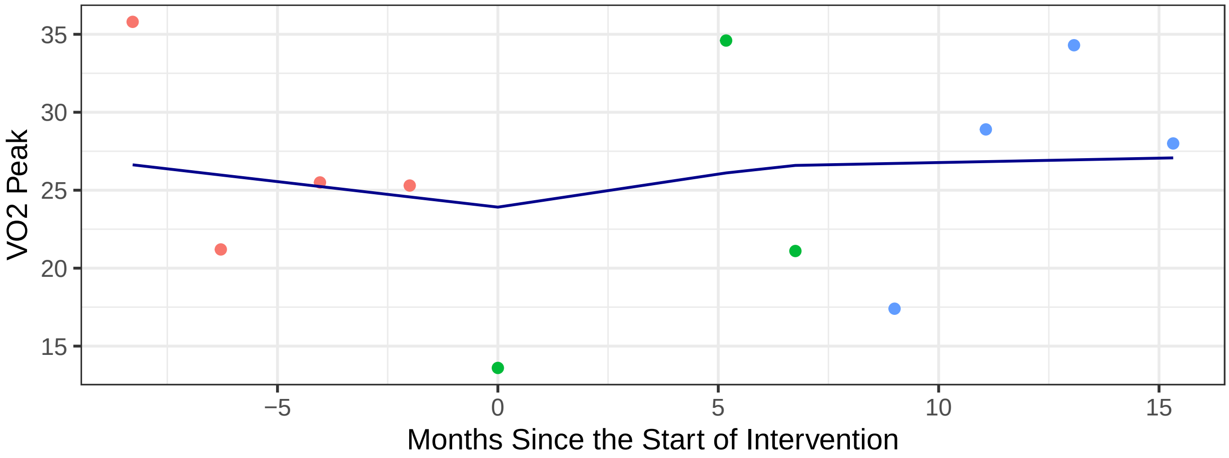

ID 067

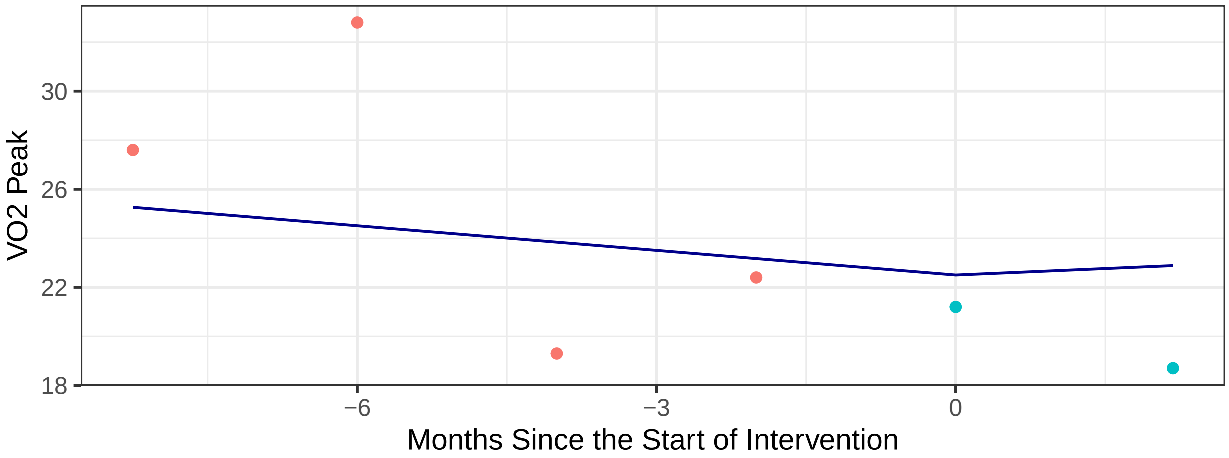

ID 068

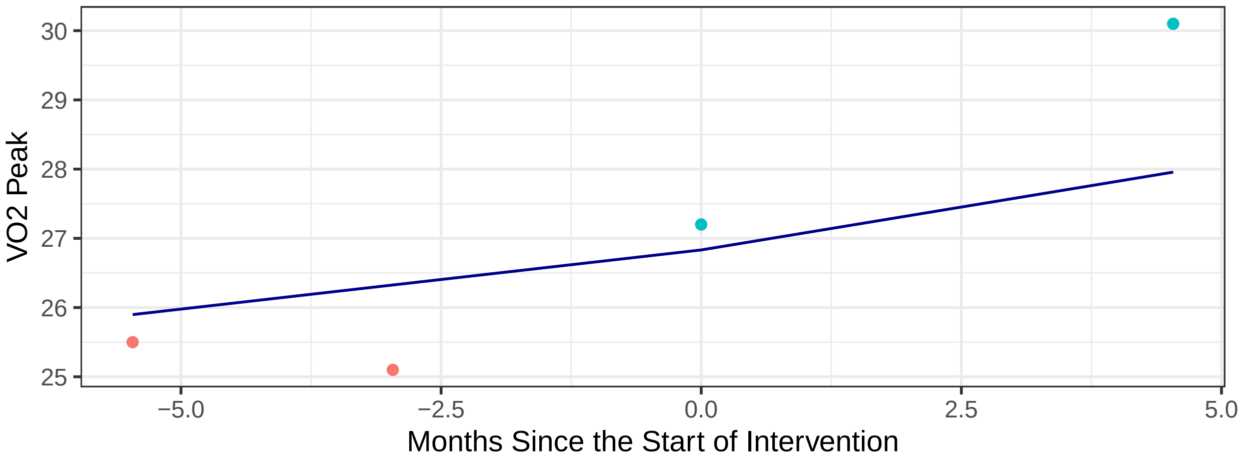

ID 071

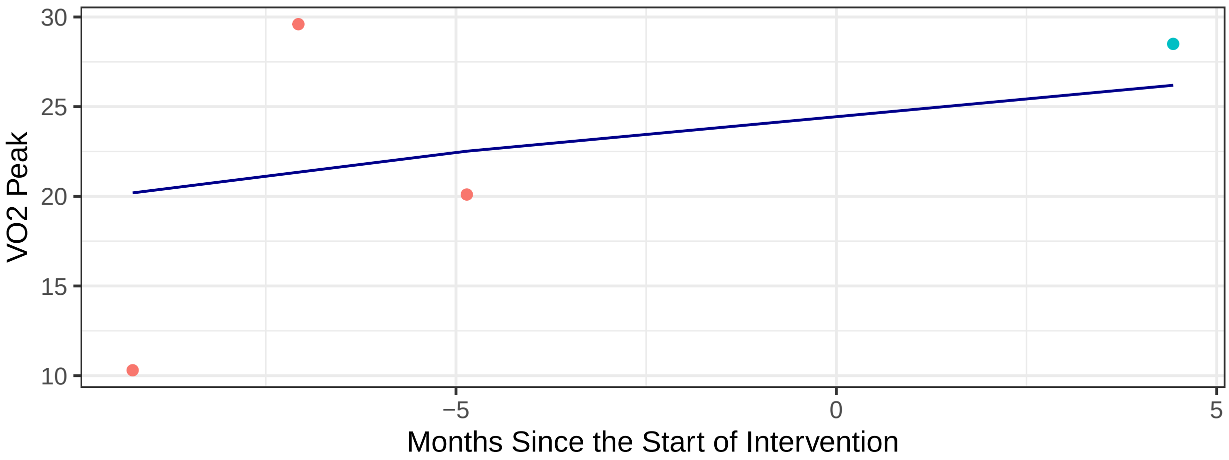

ID 072

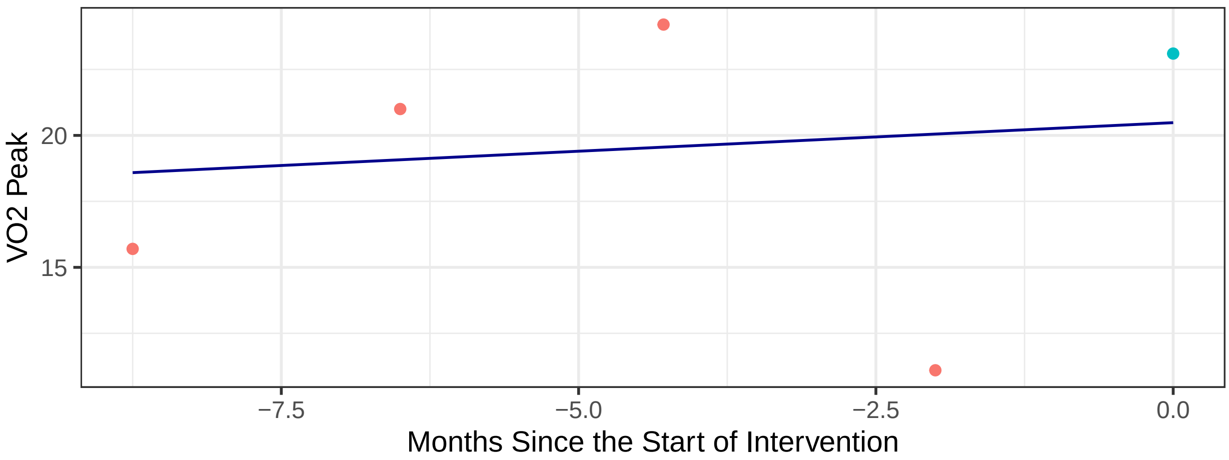

ID 074

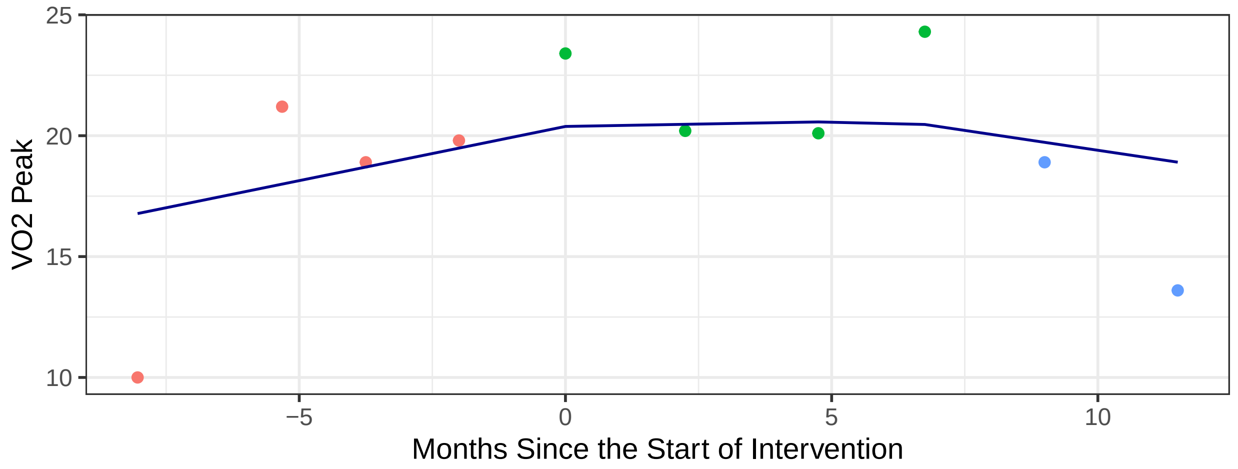

ID 076

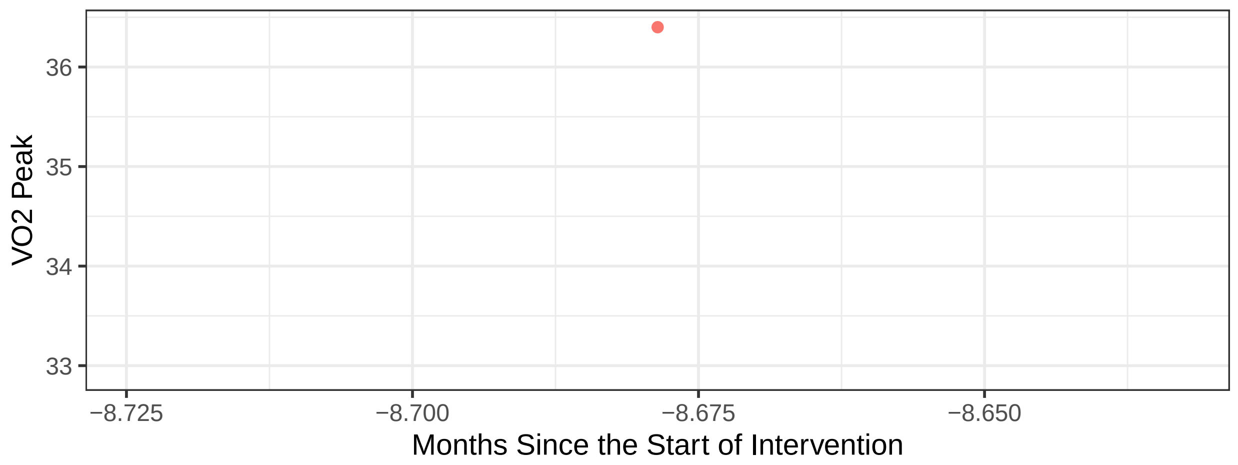

ID 077

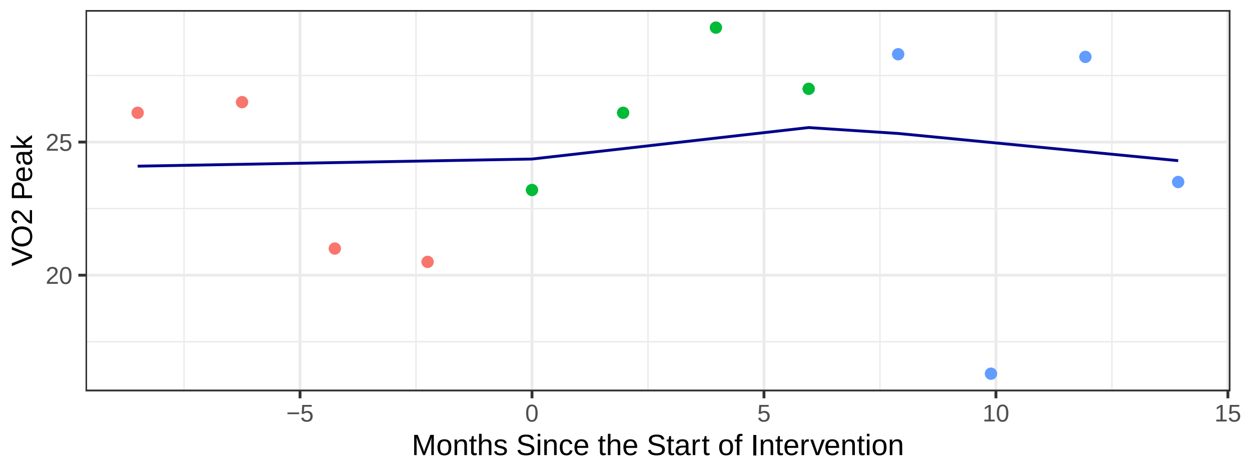

ID 078

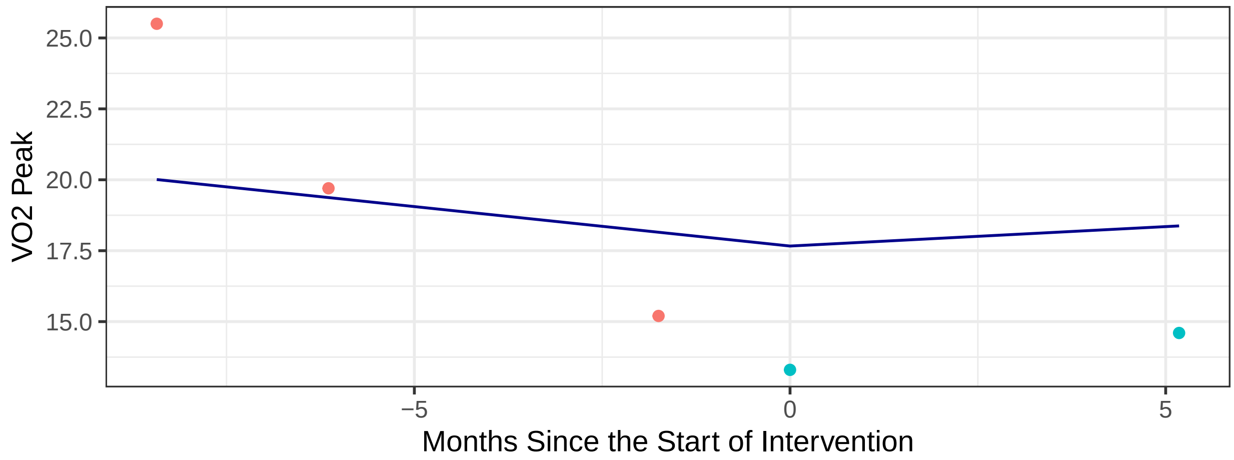

ID 079

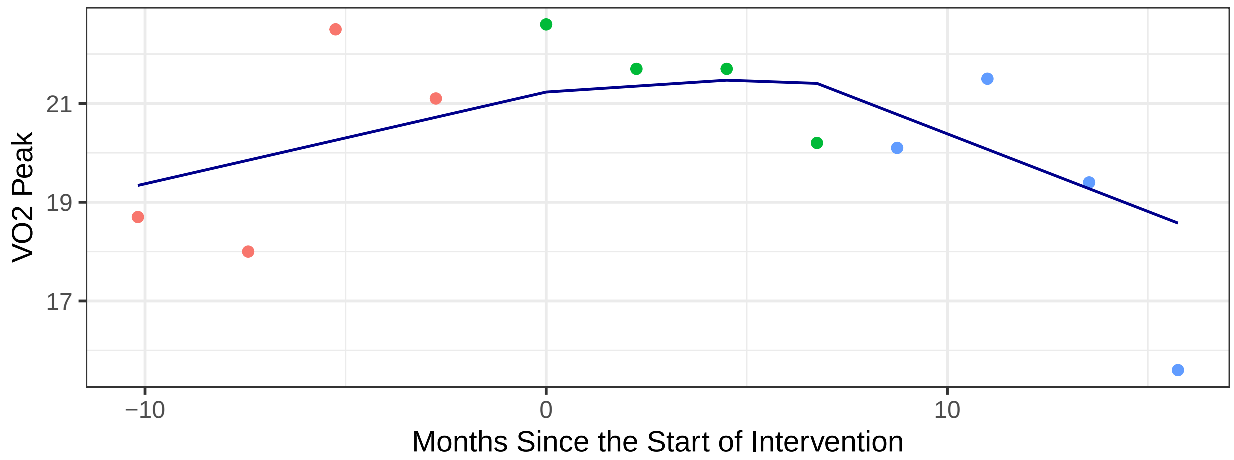

ID 080

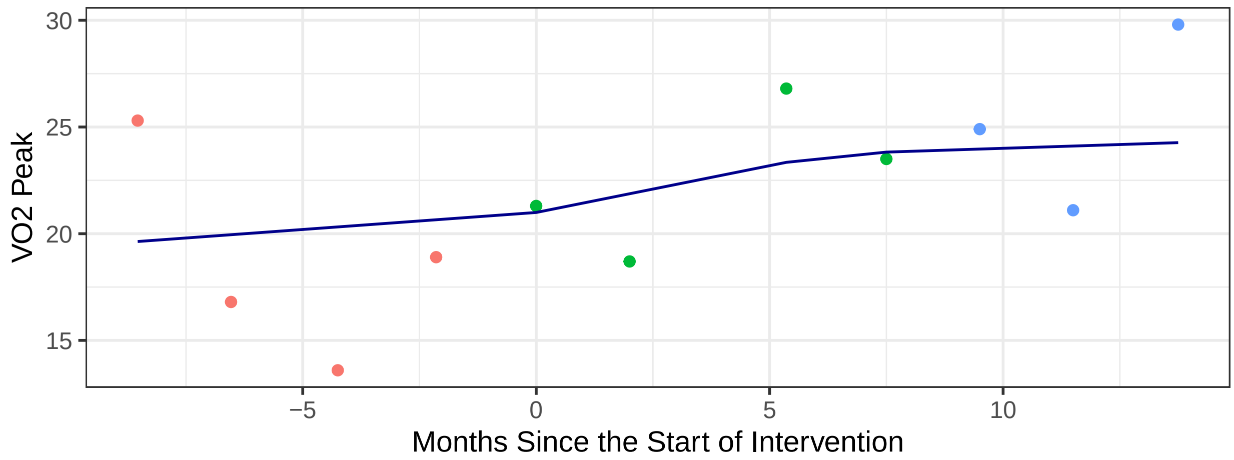

ID 082

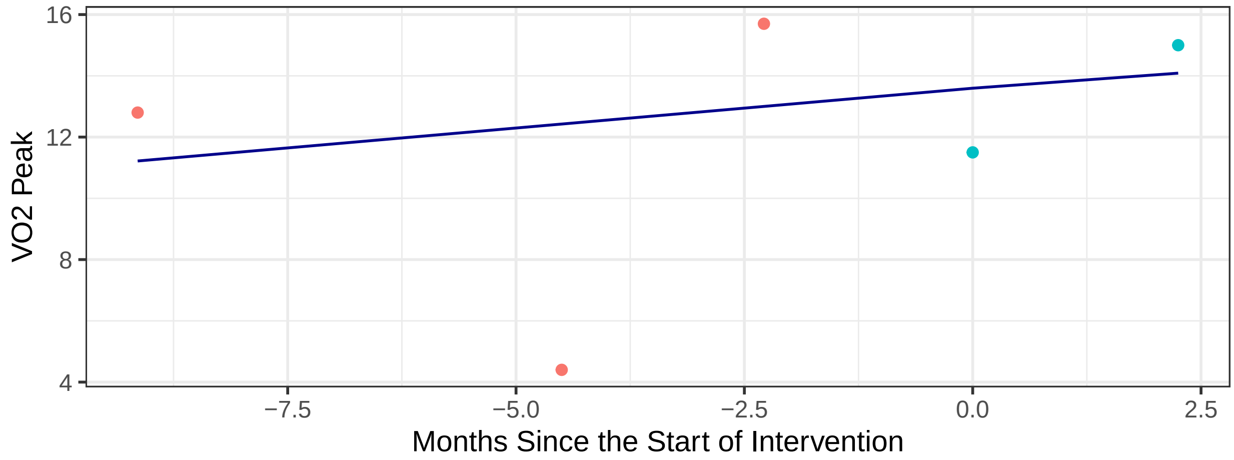

ID 083

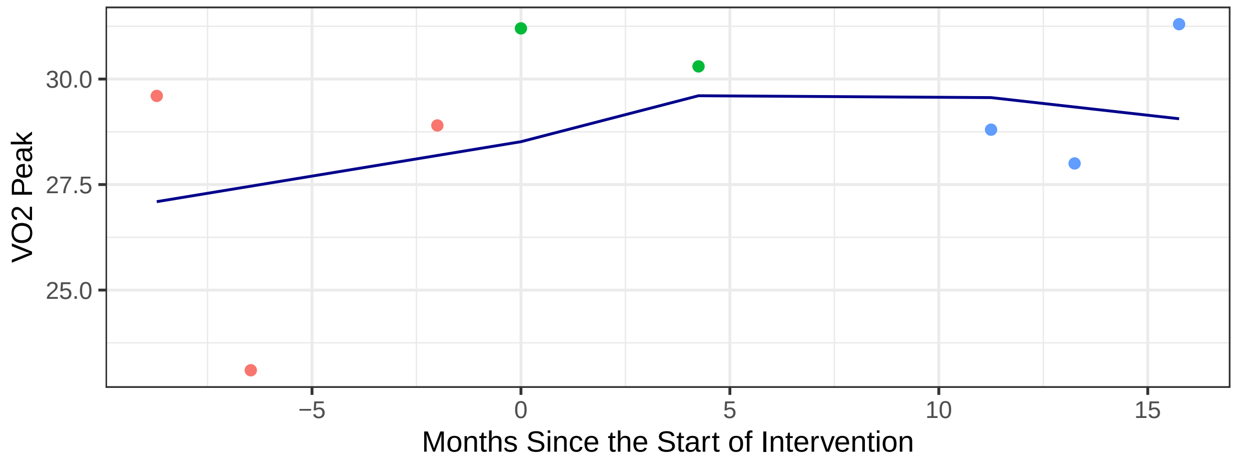

ID 085

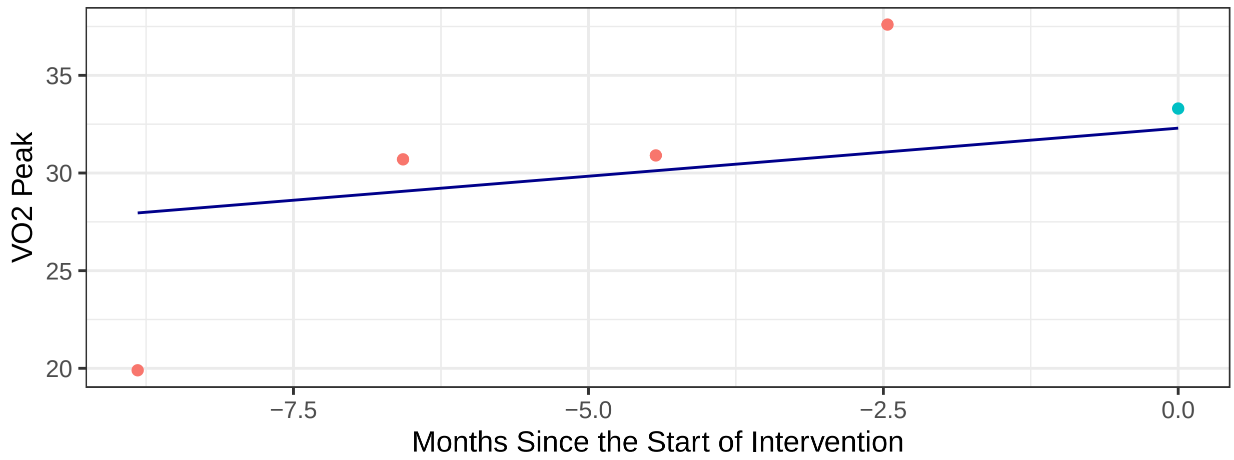

ID 086

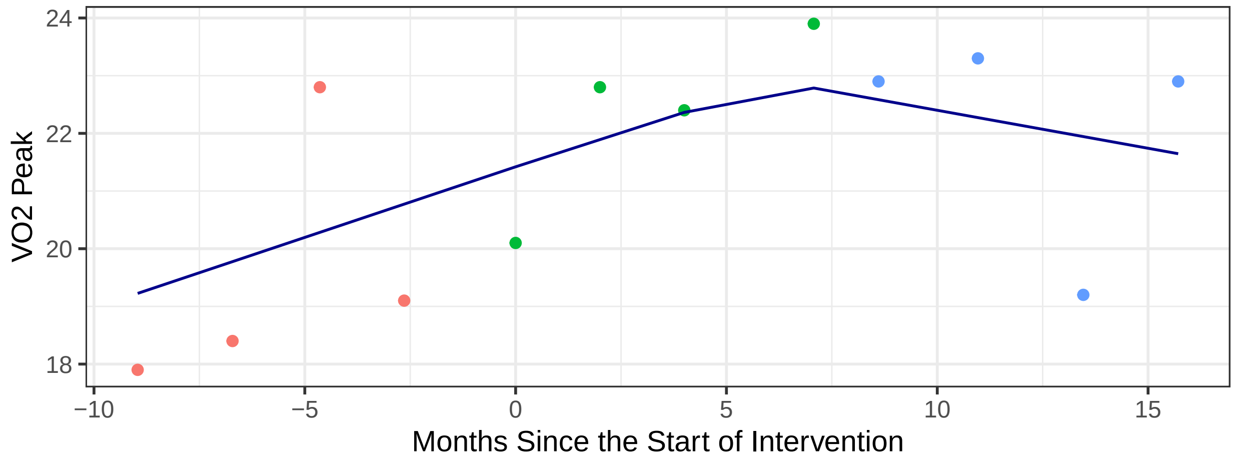

ID 088

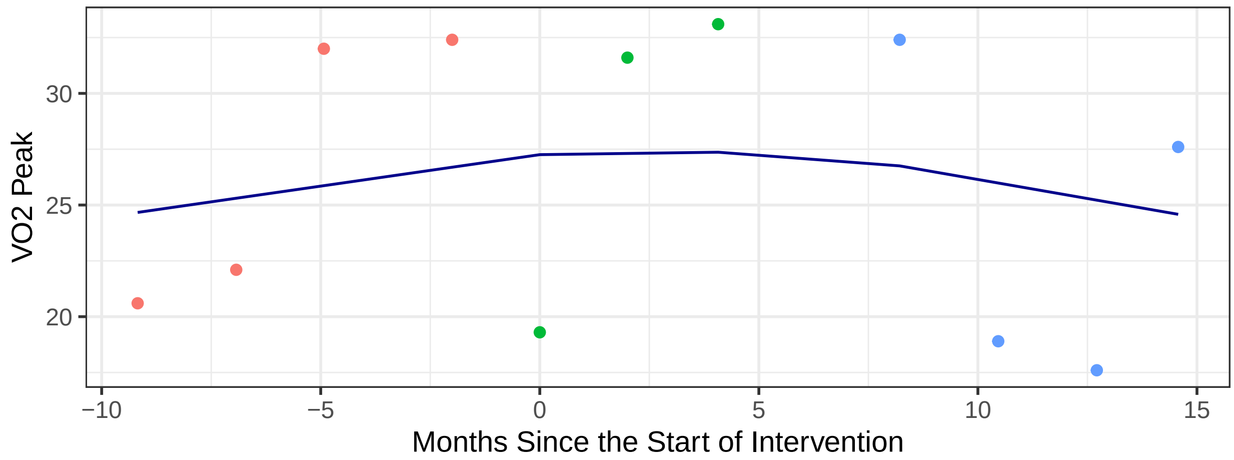

ID 089

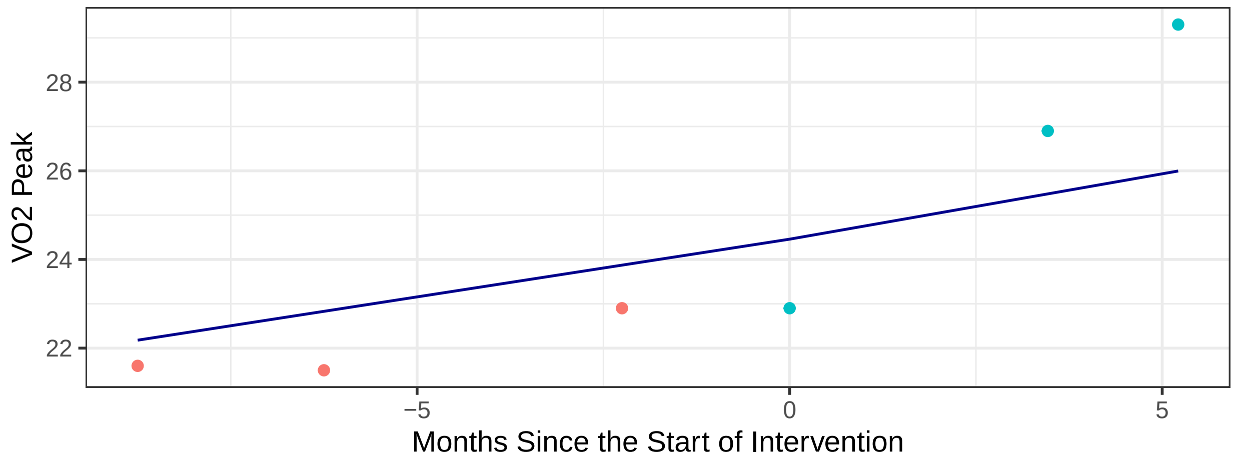

ID 091

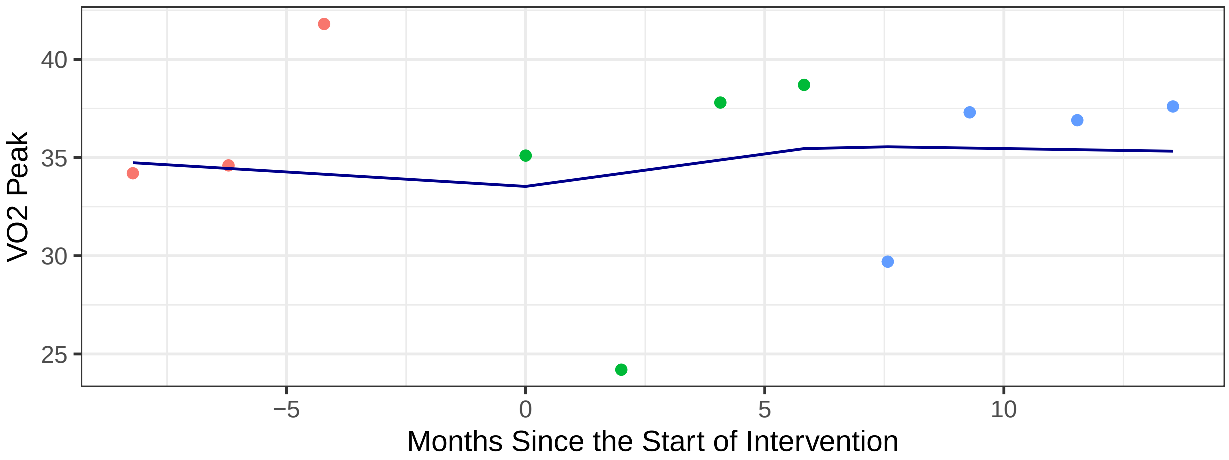

ID 092

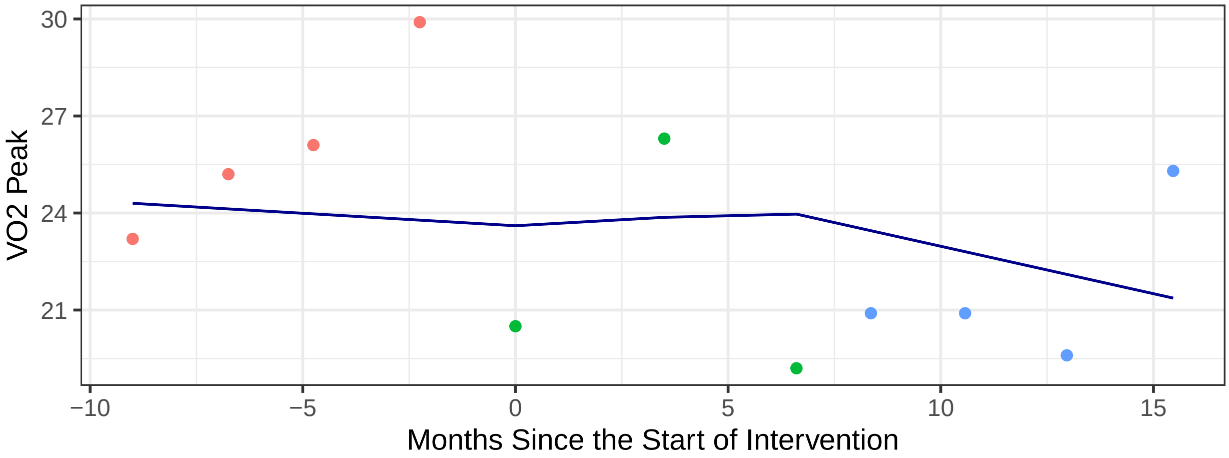

ID 093

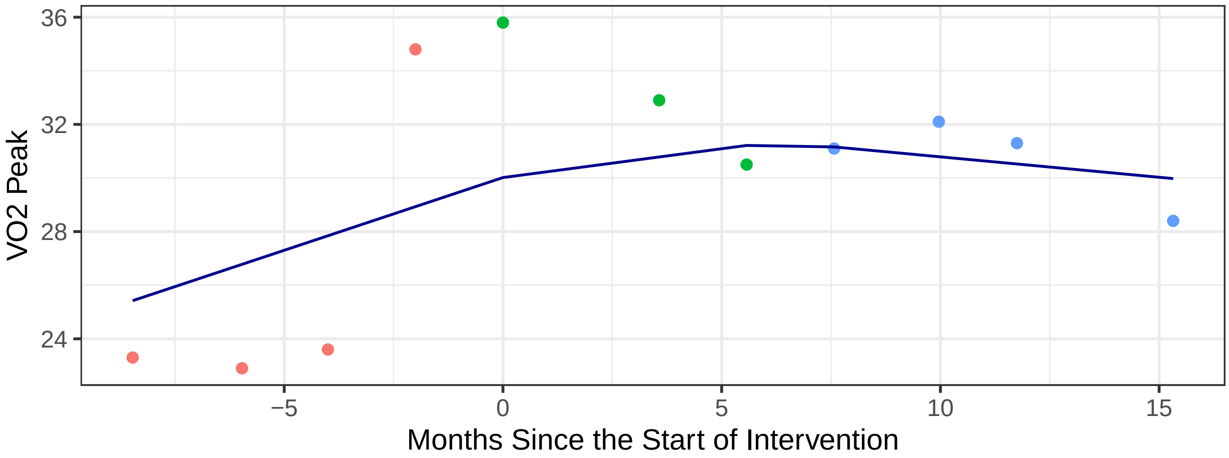

ID 094

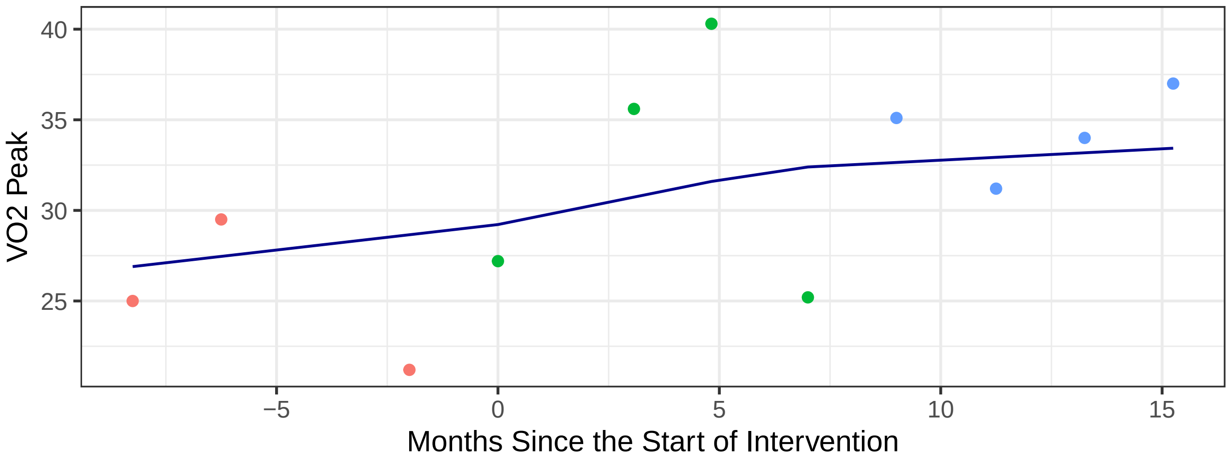

ID 095

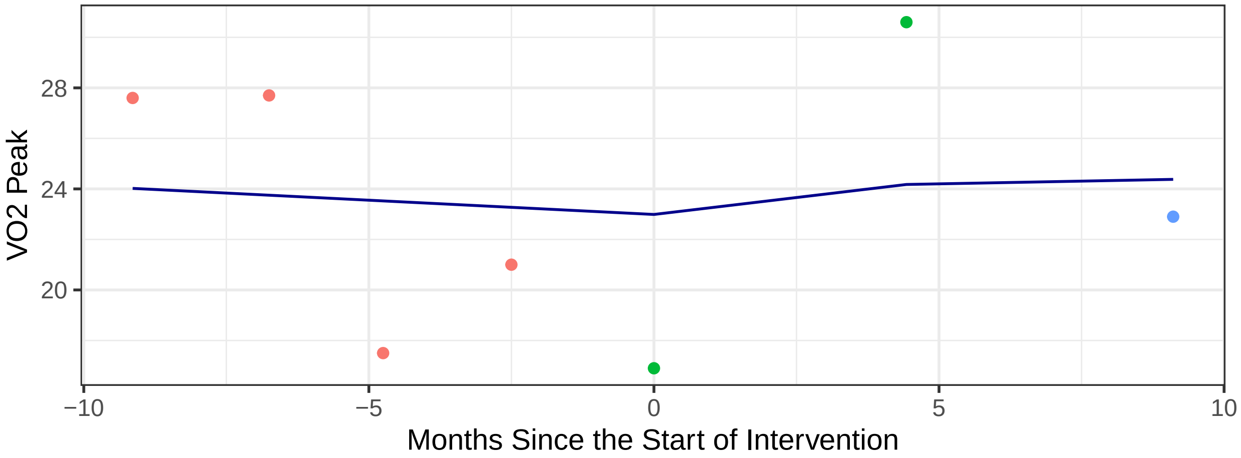

ID 096

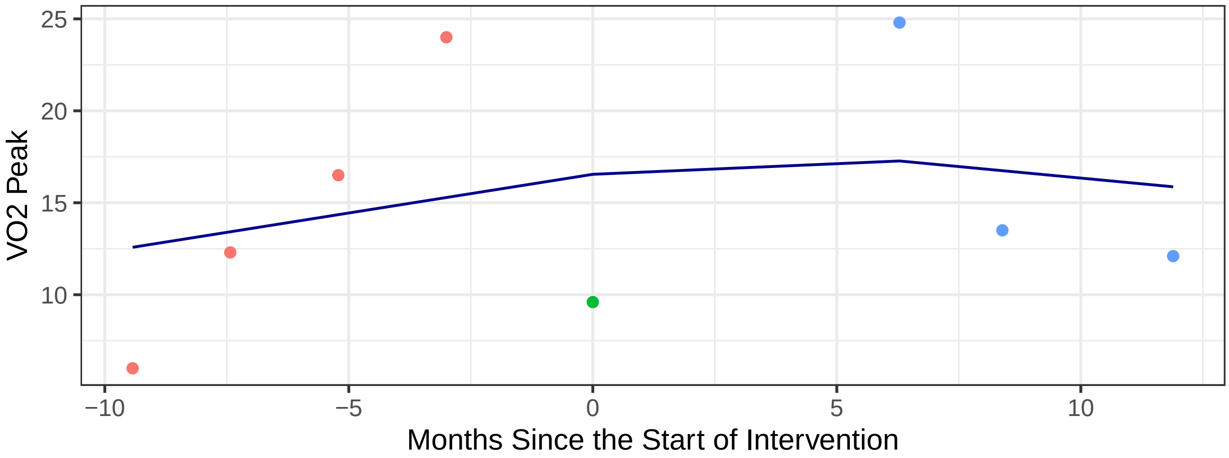

ID 097

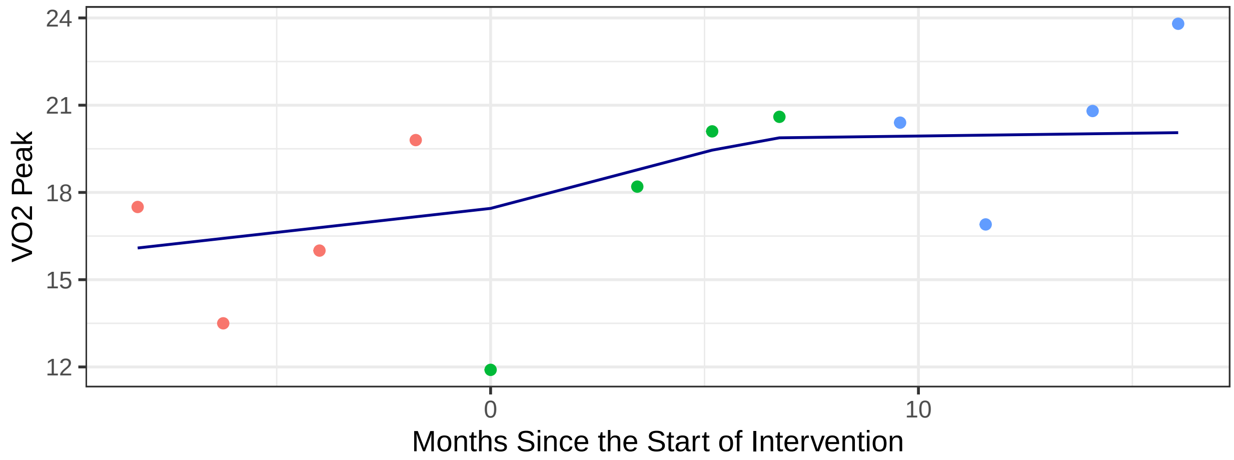

ID 098

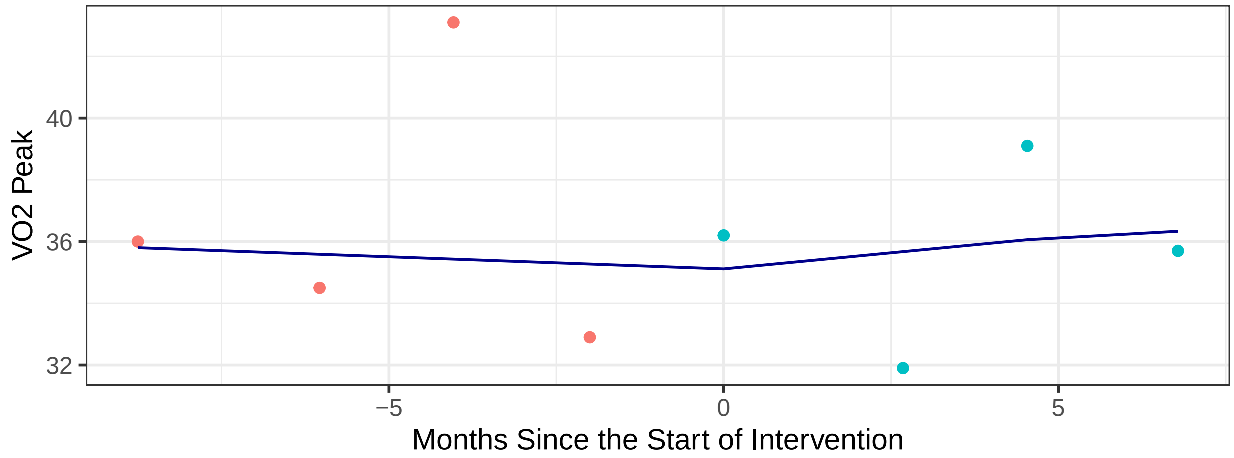

ID 099

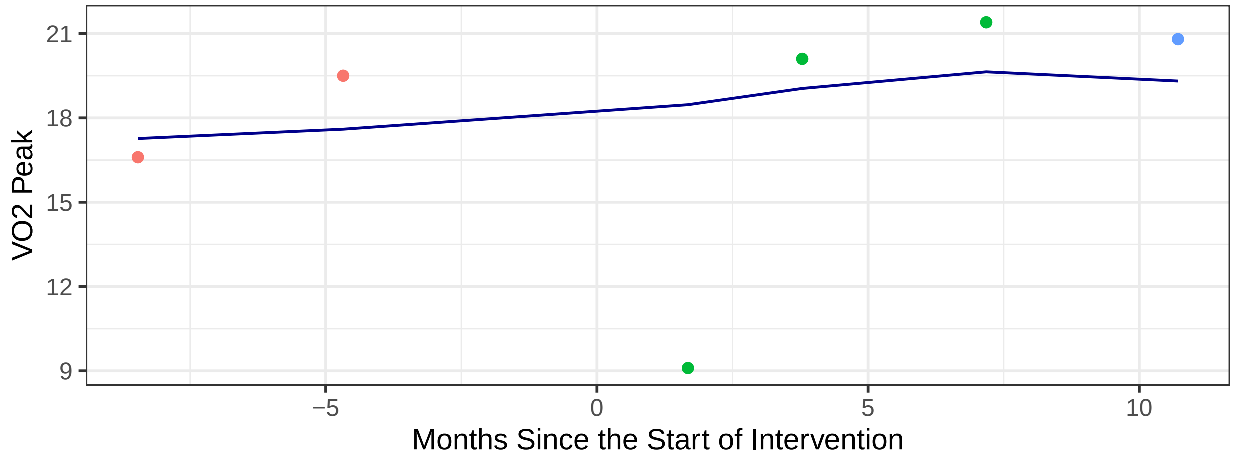

ID 100

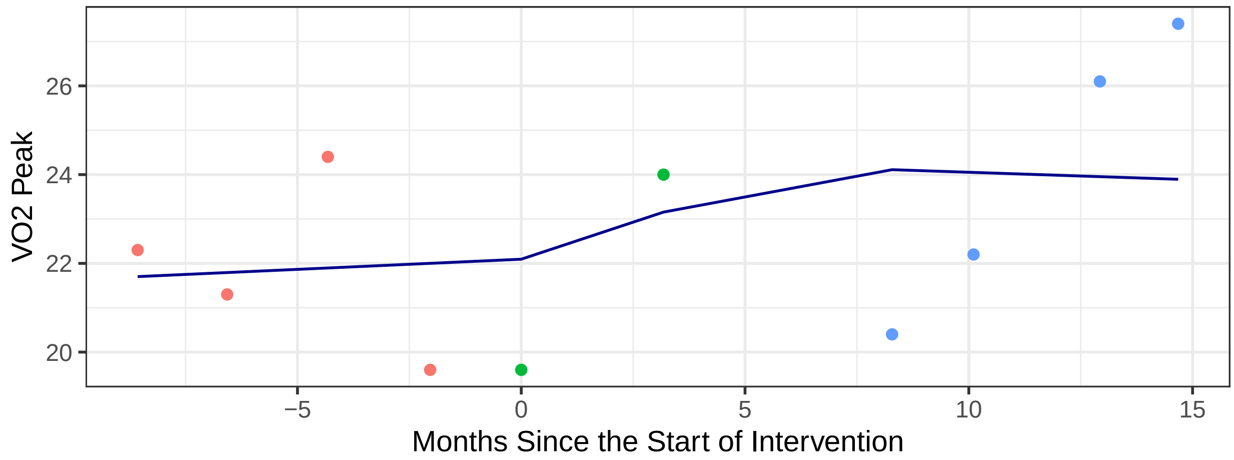

ID 101

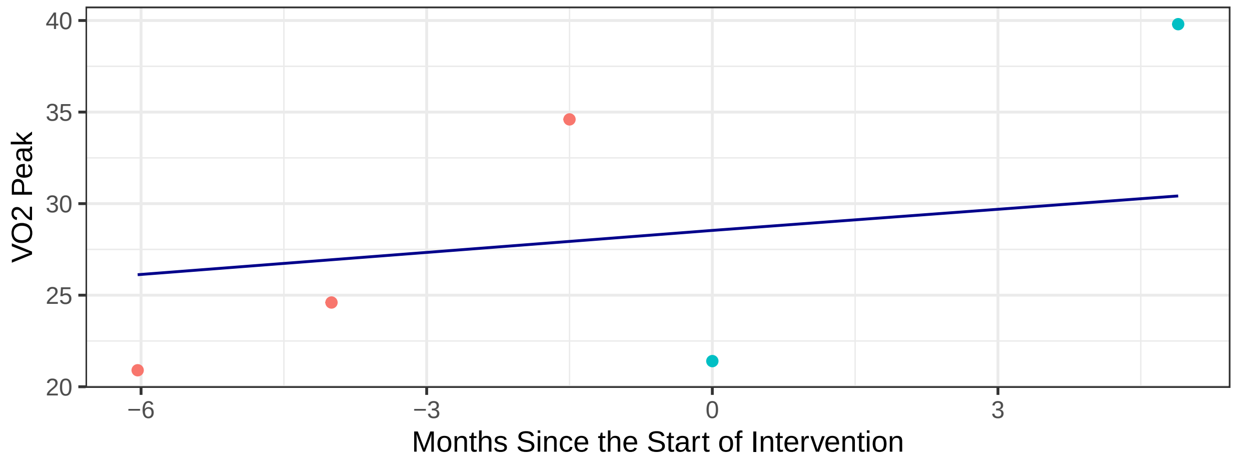

ID 102

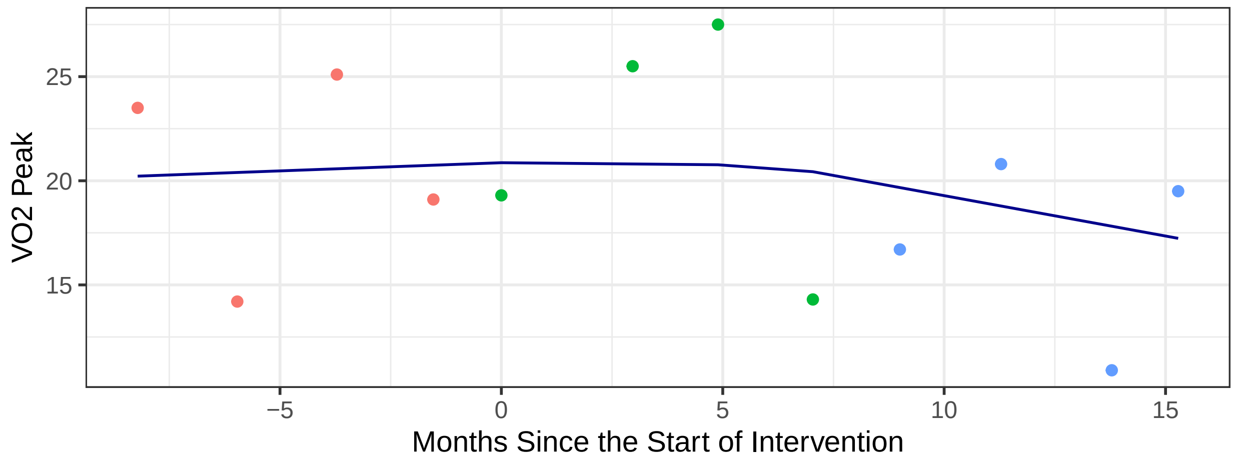

ID 104

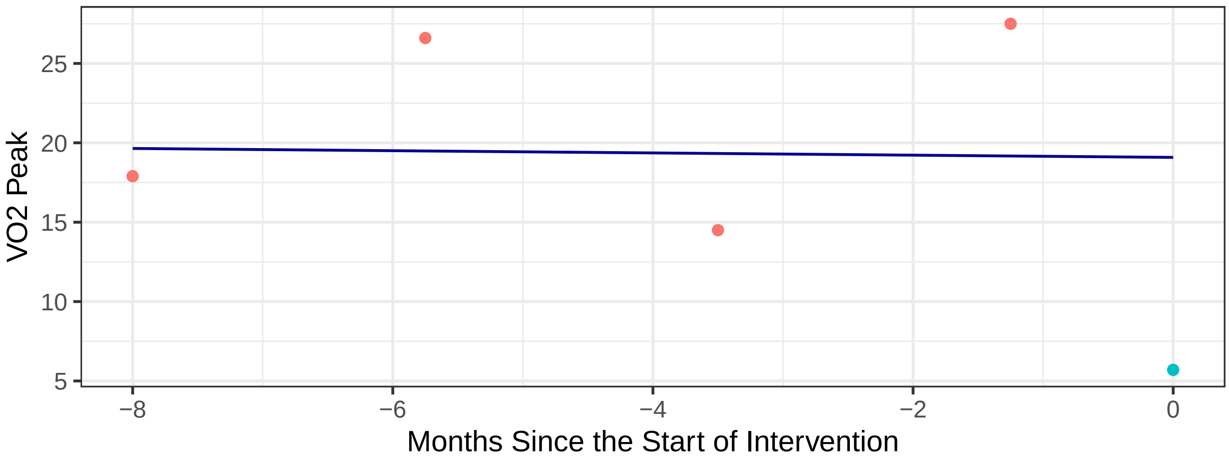

ID 105

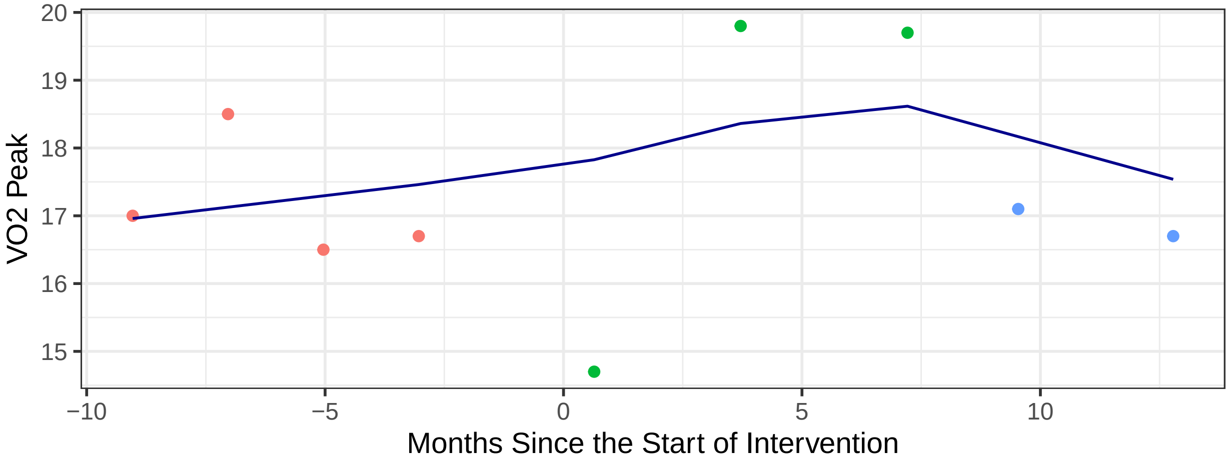

ID 107

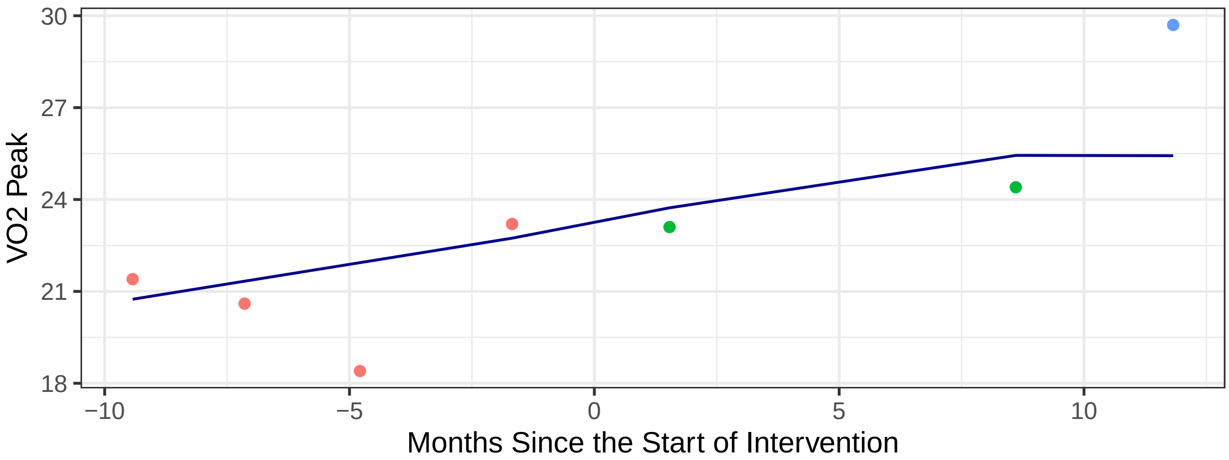

ID 108

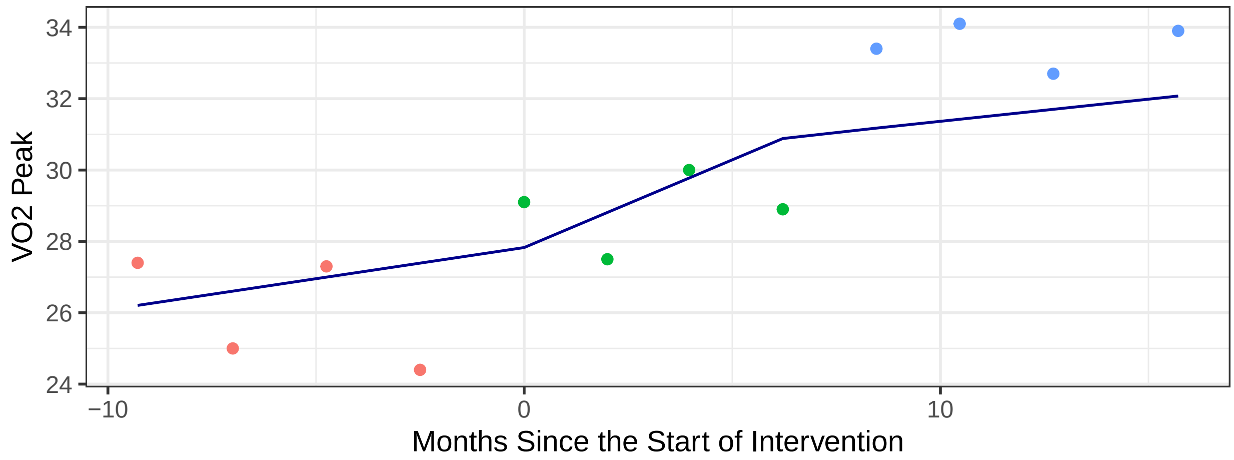

ID 109

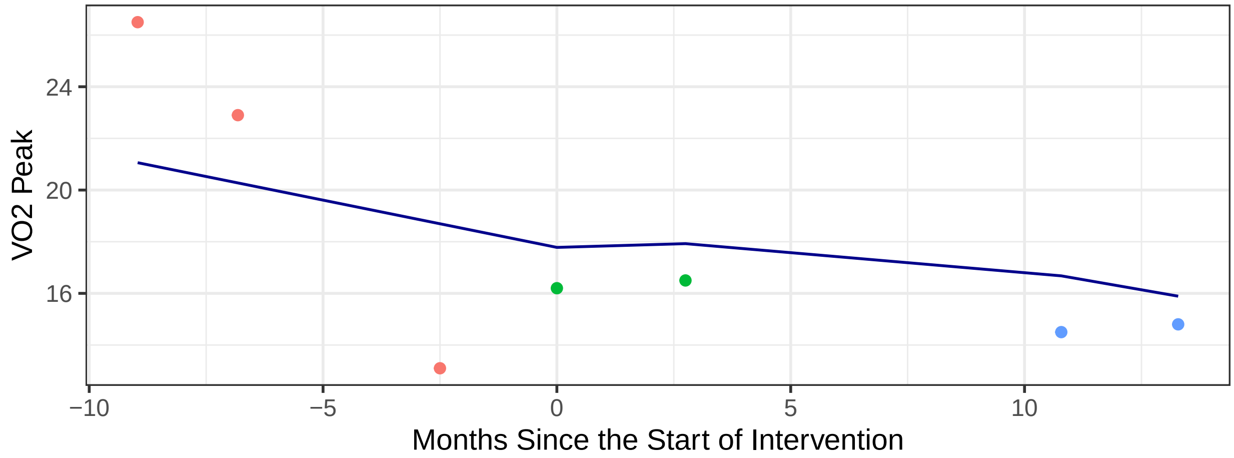

ID 110

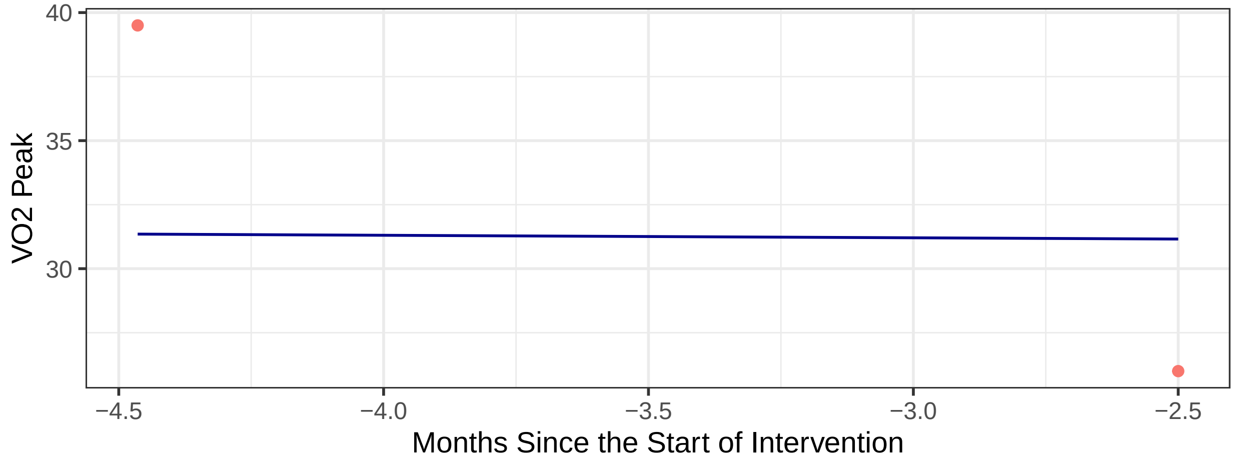

ID 111

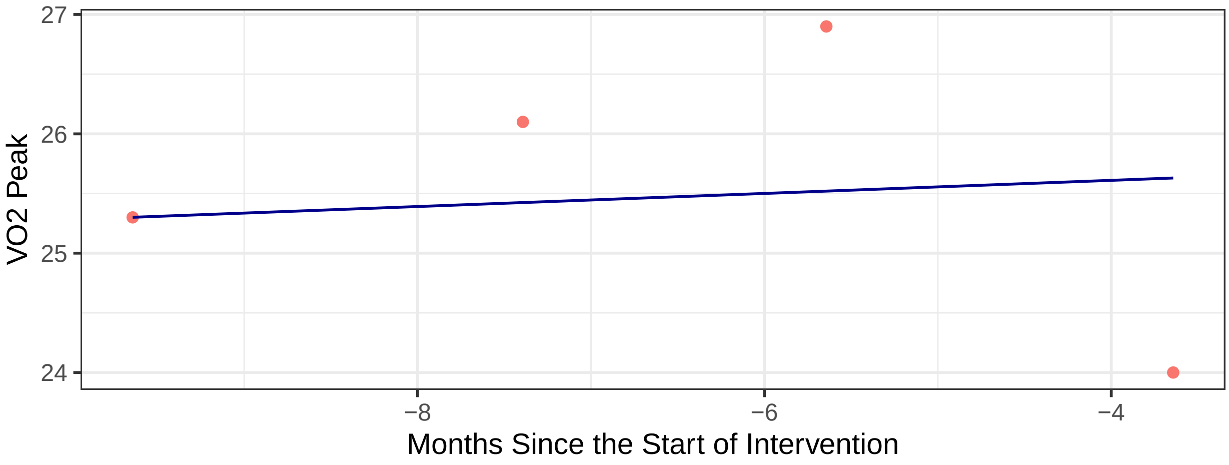

ID 112

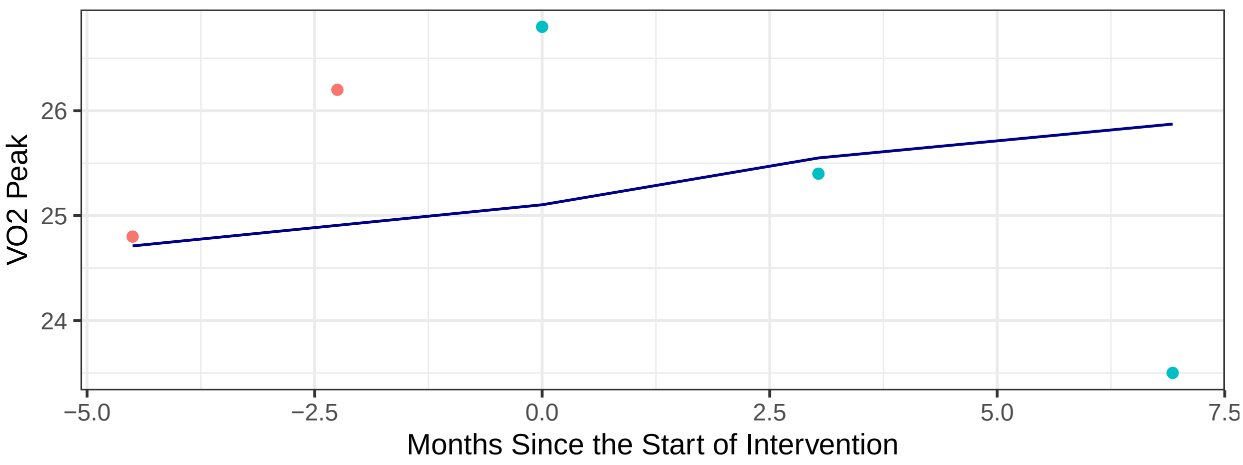

ID 113

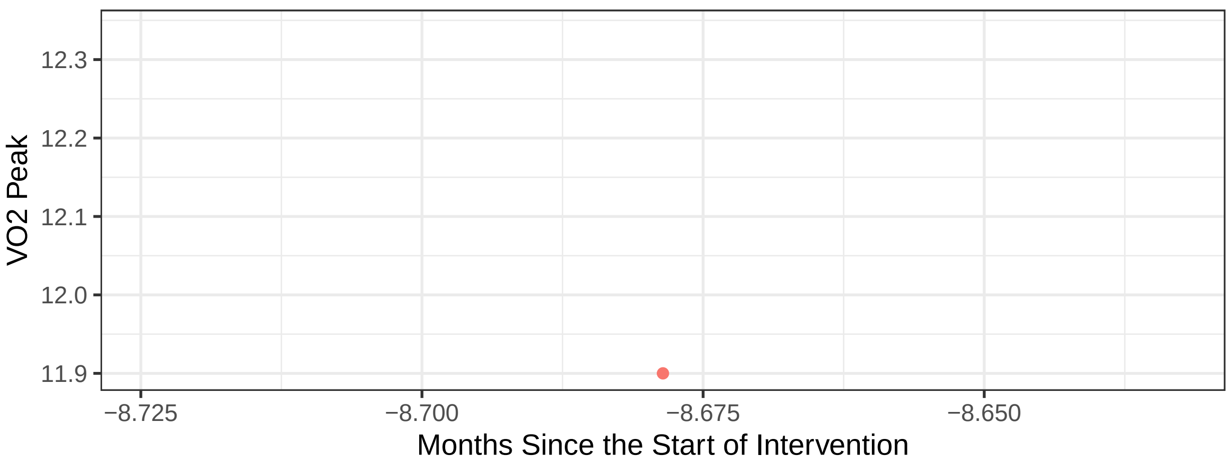

ID 114

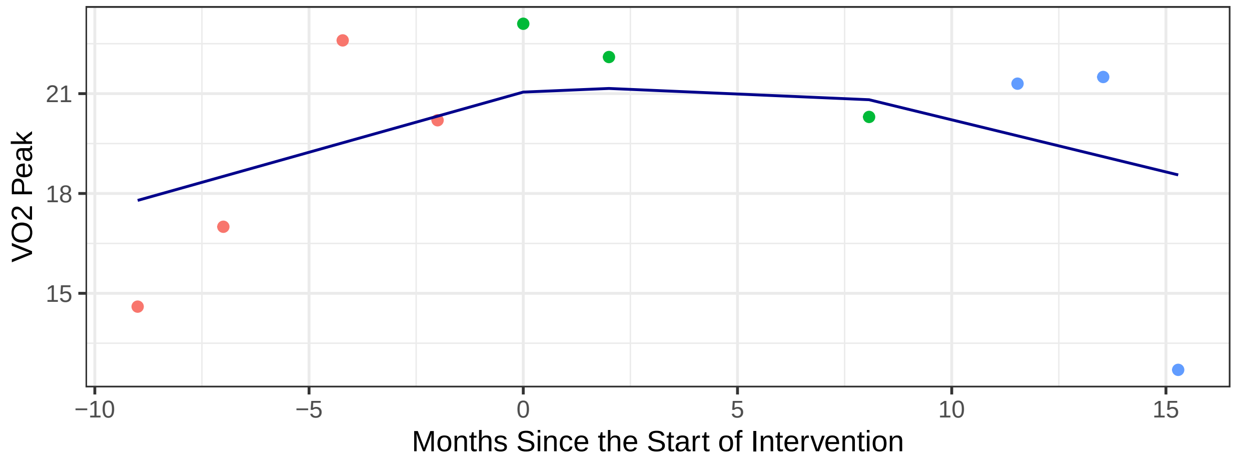

ID 115

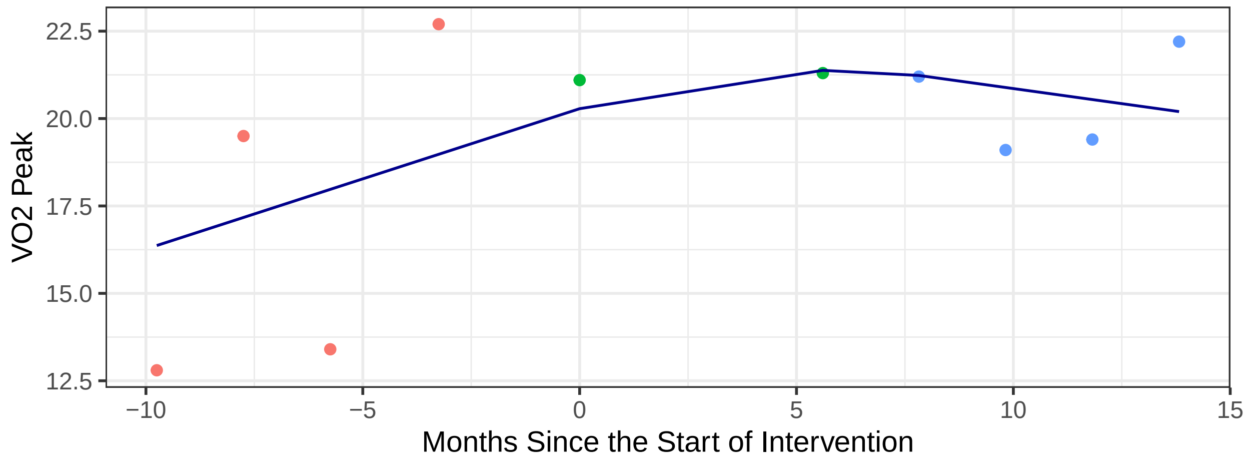

ID 116

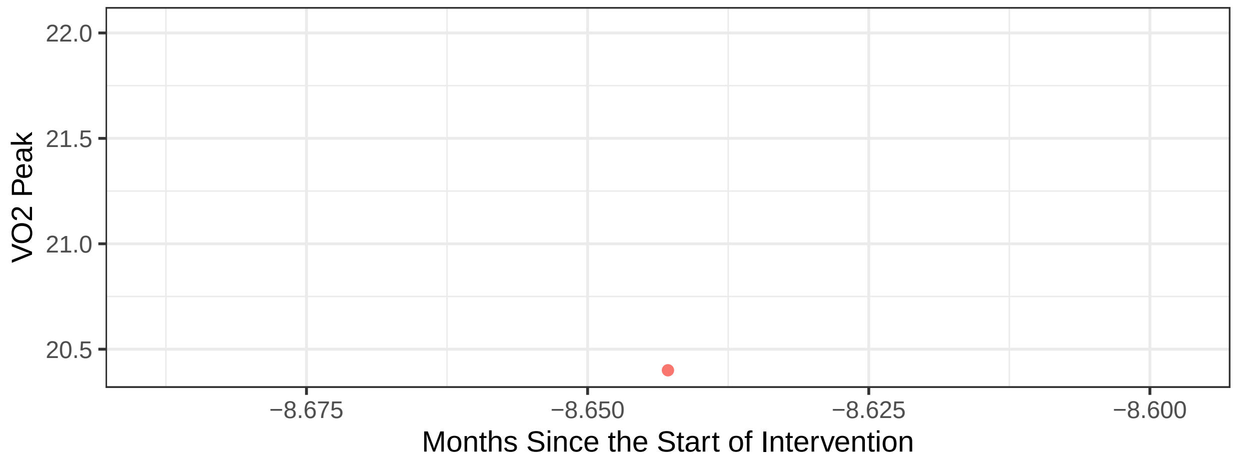

ID 117

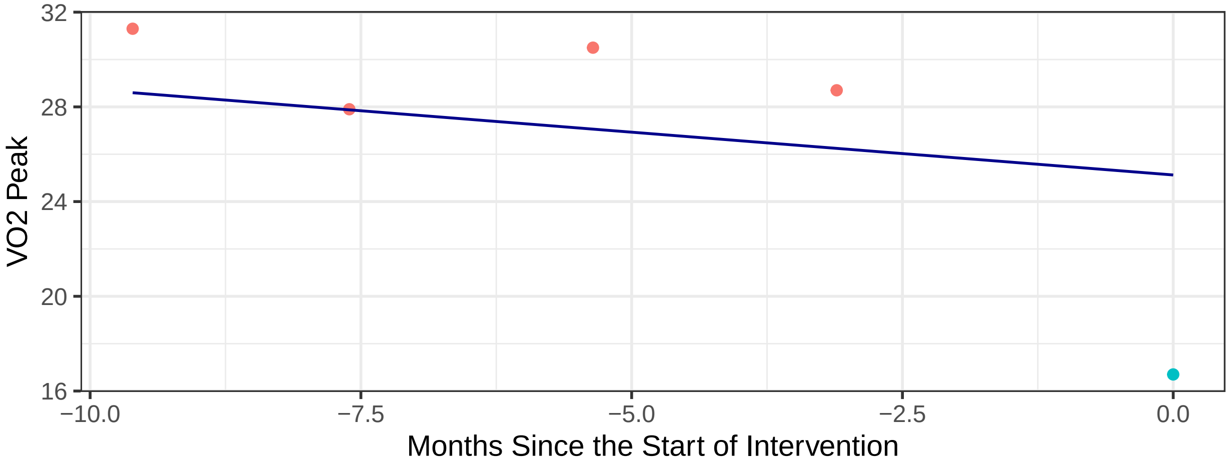

ID 118

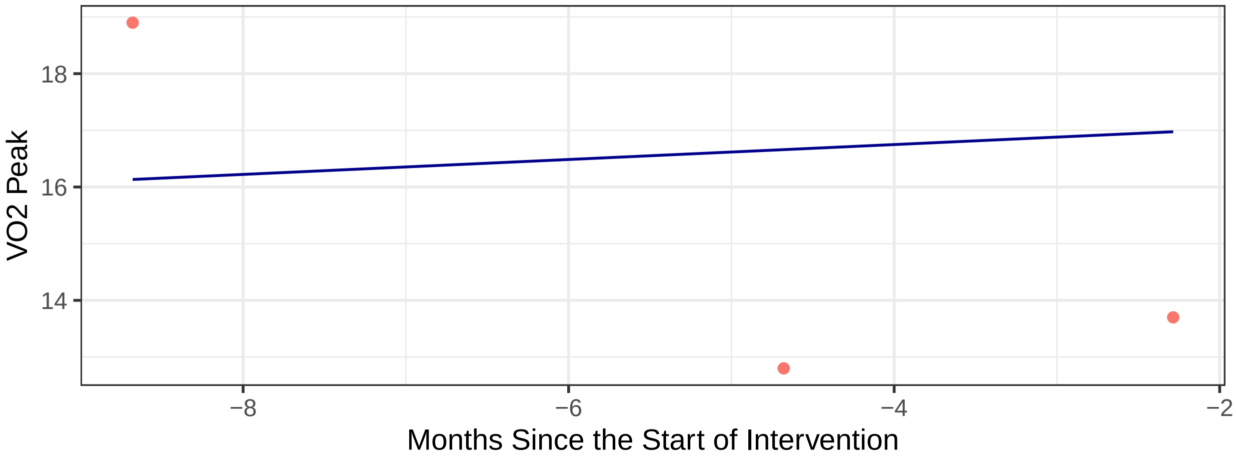

ID 119

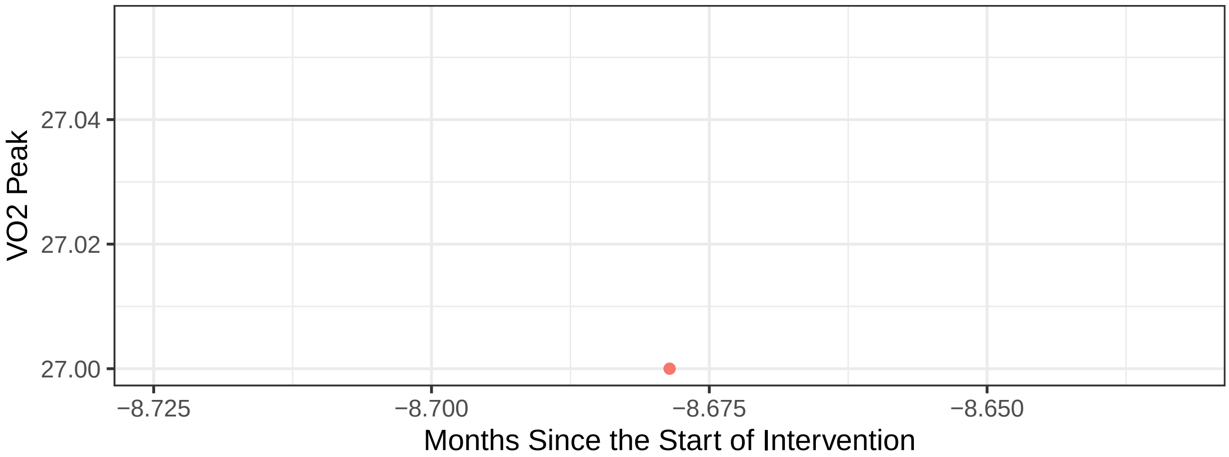

ID 120

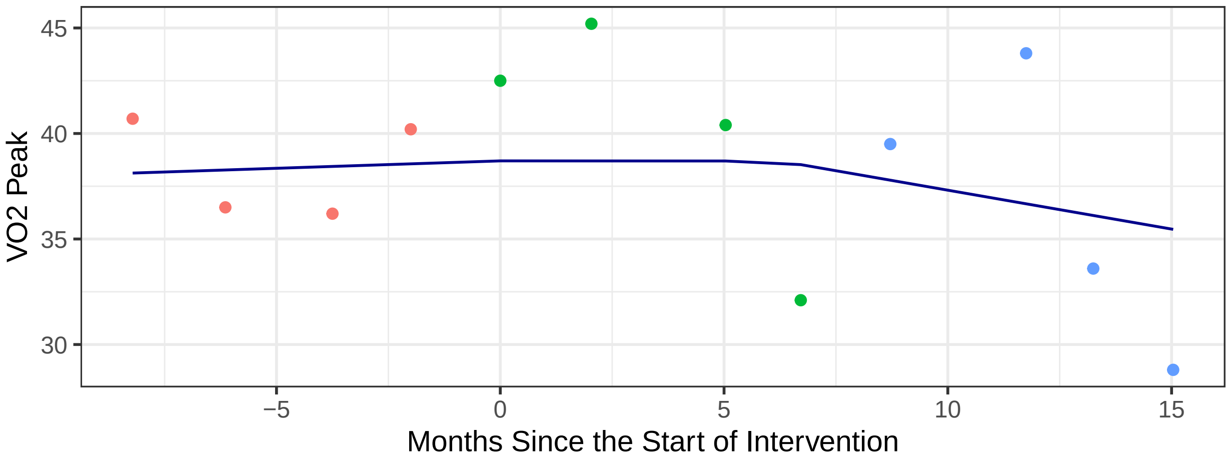

45
40
35
30
VO2 Peak
−5
0
5
10
15
Months Since the Start of Intervention

ID 121

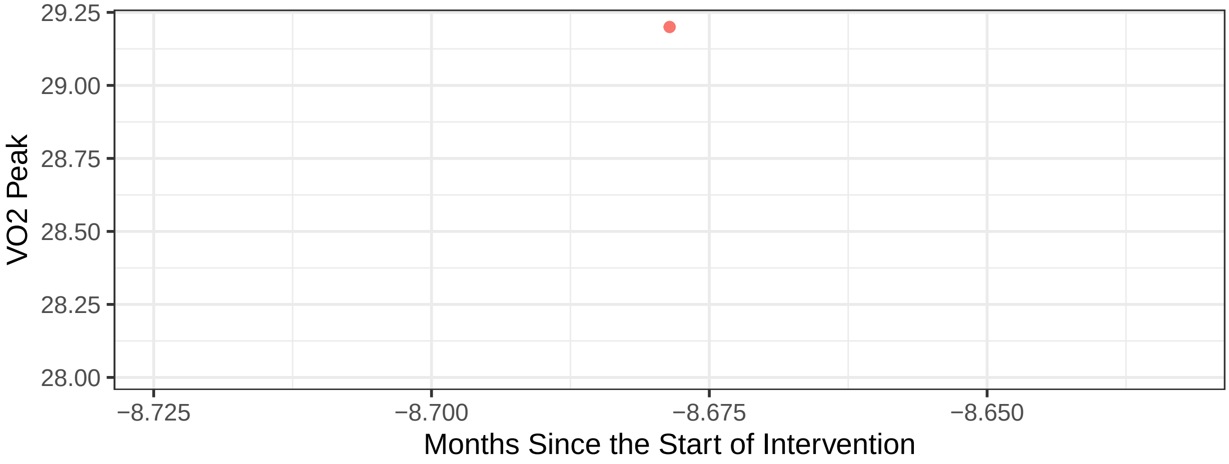

29.25
29.00
28.75
28.50
28.25
28.00
VO2 Peak
−8.725
−8.700
−8.675
−8.650
Months Since the Start of Intervention
