# Supplementary material for: Examining the impact of a community-based exercise intervention on cardiorespiratory fitness, cardiovascular health, strength, flexibility and physical activity among adults living with HIV: A three-phased intervention study
Source: PLoS One. 2021 Sep 24;16(9):e0257639. doi: 10.1371/journal.pone.0257639 (PMC8462727; doi:10.1371/journal.pone.0257639)
Supplement: S1 File — (PDF) [file pone.0257639.s002.pdf]

Supplemental File 1 - Statistical Supplement

Primary Analysis: Segmented Regression Model

The following model was fit to evaluate the effect of the intervention on V̇O2peak. The quantity of interest is $D(\beta_2 - \beta_1)$, where D is equal to six, the intended length of the intervention in months.

Primary Model

$$y_{ij} = \beta_0 + \beta_1 x_{1i} + \beta_2 x_{2i} + \beta_3 x_{3i} + \beta_4(Age - 50) + \beta_5 Woman + b_{0i} + b_{1i} + \epsilon_{ij}$$

Where:

$$b_i = \begin{vmatrix} b_{0i} \\ b_{1i} \end{vmatrix} \sim \mathcal{N}\left(0, \begin{vmatrix} \sigma_{0i} \\ \sigma_{1i} \end{vmatrix}\right), \epsilon_{ij} \sim \mathcal{N}(0, \sigma^2)$$

- b_i is a vector of random person-level effects (for intercept and Phase 1 slope)
- ϵ_{ij} are the residual errors, see note below
- β_0 is the average V̇O2peak at the beginning of Phase 2 for a 50-year old male
- β_1 is the average change in V̇O2peak (slope) during Phase 1 (baseline)
- β_2 is the average change in V̇O2peak (slope) during Phase 2 (intervention)
- β_3 is the difference in change in V̇O2peak during Phase 3 (follow-up) compared with Phase 2
- β_4 is the difference in V̇O2peak, per year of age, relative to a person who is 50.
- β_5 is the difference in V̇O2peak for females.
- x_{1i} is number of months (28 days) prior to the start of the intervention for person i
- x_{2i} is number of months (28 days) since the start of the intervention for person i
- x_{3i} is number of months (28 days) since the end of the protocol-defined intervention period (25 weeks)

$$x_{3i} = \begin{cases} x_{2i} - 175, & \text{if } x_{2i} > 175 \\ 0, & \text{otherwise} \end{cases}$$

The estimated intervention effect is calculated as $I = 6(\beta_2 - \beta_1)$, corresponding to the expected change in V̇O2peak due to the six month intervention. The variation in I (s_I^2) is given by:

$$s_I^2 = 36(s_{\beta_1}^2 + s_{\beta_2}^2 - 2r_{\beta_1\beta_2}s_{\beta_1}s_{\beta_2})$$

where: $s_{\beta_1} = 0.0808480$, $s_{\beta_2} = 0.1030961$ and $r_{\beta_1\beta_2} = -0.448$

An auto correlated error model was tested, but the auto correlation was small ($\rho = 0.06$), and did not improve model fit as assessed with a likelihood ratio test, so normally distributed residuals were assumed.

Post Hoc Exploratory Analyses

Exploratory Model Incorporating Dose

$$y_{ij} = \beta_0 + \beta_1 x_{1i} + \beta_2 x_{2i} d_i + \beta_3 x_{3i} + \beta_4 (Age - 50) + \beta_5 Woman + b_{0i} + b_{1i} + b_{2i} + \epsilon_{ij}$$

Terms are as for the previous model, with the following additions: d_i indicates the participant's median number of minutes of moderate to vigorous activity and b_{2i} is the random person effect associated with change during the intervention phase.

The expected intervention effect for someone meeting the Canadian Physical Activity Guidelines (CPAGs) of 150 min/week was then calculated as I = 6 × 150 $(\beta_2 - \beta_1)$ with associated variation:

$$s_I^2 = 36(150^2\, s_{\beta_1}^2 + s_{\beta_2}^2 - 300 r_{\beta_1 \beta_2} s_{\beta_1} s_{\beta_2})$$
