# Supplementary material for: Examining the impact of a community-based exercise intervention on cardiorespiratory fitness, cardiovascular health, strength, flexibility and physical activity among adults living with HIV: A three-phased intervention study
Source: PLoS One. 2021 Sep 24;16(9):e0257639. doi: 10.1371/journal.pone.0257639 (PMC8462727; doi:10.1371/journal.pone.0257639)
Supplement: S1 Table — Characteristics for participants who completed demographic questionnaire at study enrollment (Time 1; Month 0): a excluding HIV. (PDF) [file pone.0257639.s004.pdf]

Impact of a community-based exercise intervention among adults living with HIV
Supplemental File 2 –Characteristics of Participants at Study Initiation in the study sample at enrollment, start of intervention, end of intervention, and end of follow-up (end of study).

Supplemental File 2 – Characteristics of Participants at Study Initiation and Study Milestones

	**Study Milestones**			
**Characteristics at Study Initiation (Phase 1)**	**Initiated Study (n=108)**	**Initiated Intervention (n=80)**	**Completed Intervention (n=67)**	**Completed Follow-up (n=52)**
**Median age (IQR)**<br>**≥ 50 years**	51 years (45, 59)<br>53 (49%)	51 (45, 60)<br>42 (53%)	53 (46, 60)<br>37 (55%)	55 (45, 61)<br>28 (54%)
**Male**<br>**Female**	96 (89%)<br>12 (11%)	73 (91%)<br>7 (9%)	60 (90%)<br>7 (10%)	47 (90%)<br>5 (10%)
**Median number of comorbidities[a] (IQR)**	5 (2,7)	5 (2,8)	5 (2, 9)	5 (2,9)
**Living with ≥2 comorbidities[a]**	89 (82%)	67 (84%)	58 (87%)	46 (89%)
**Most commonly self-reported co-morbidities (>30%) included:**<br>Mental health (e.g. depression, anxiety)<br>Joint pain (e.g. arthritis)<br>Muscle pain<br>Bone and joint disorder (osteopenia, osteoporosis, osteoarthritis)	 52 (48%)<br>44 (41%)<br>39 (36%)<br>39 (36%)	 43 (54%)<br>34 (43%)<br>28 (35%)<br>27 (34%)	 35 (52%)<br>29 (43%)<br>25 (37%)<br>21 (31%)	 21 (40%)<br>23 (44%)<br>21 (40%)<br>16 (31%)
**Median number of years since HIV diagnosis (25-75th percentile)**	17 (8, 27)	18 (8, 27)	21 (9, 28)	21 (9, 29)
**Current antiretroviral (HIV medication) use**	107 (99%)	80 (100%)	67 (100%)	52 (100%)
**Self-reported viral load undetectable** (<50 copies/mL)	90 (83%)	69 (86%)	57 (85%)	45 (87%)
**Self-reported current health status**<br>Excellent<br>Very good or good<br>Fair<br>Poor	 8 (7%)<br>80 (74%)<br>18 (17%)<br>2 (2%)	 4 (6%)<br>61 (91%)<br>15 (22%)<br>0 (0%)	 2 (3%)<br>52 (78%)<br>13 (19%)<br>0 (0%)	 2 (4%)<br>43 (83%)<br>7 (33%)<br>0 (0%)
**Health status compared to previous year**<br>Better now than 1 year ago<br>About the same as 1 year ago<br>Worse than 1 year ago	 50 (46%)<br>42 (39%)<br>16 (15%)	 37 (55%)<br>32 (48%)<br>11 (16%)	 30 (45%)<br>27 (40%)<br>10 (15%)	 23 (44%)<br>24 (46%)<br>5 (10%)
**Employed Full-Time or Part-Time**	34 (31%)	28 (35%)	21 (31%)	19 (37%)

Date Last Revised: August 16, 2021

Impact of a community-based exercise intervention among adults living with HIV

Supplemental File 2 –Characteristics of Participants at Study Initiation in the study sample at enrollment, start of intervention, end of intervention, and end of follow-up (end of study).

	Study Milestones			
**Characteristics at Study Initiation (Phase 1)**	**Initiated Study (n=108)**	**Initiated Intervention (n=80)**	**Completed Intervention (n=67)**	**Completed Follow-up (n=52)**
**Smoking History**				
I currently smoke regularly or occasionally	32 (30%)	21 (31%)	17 (46%)	12 (23%)
I am a former smoker	29 (30%)	27 (40%)	25 (37%)	20 (38%)
I have never been a smoker	40 (37%)	29 (43%)	22 (33%)	17 (33%)
**Have Children**	16 (15%)	10 (13%)	9 (13%)	8 (15%)
**Live Alone**	73 (68%)	51 (64%)	43 (64%)	33 (64%)
**Gross average yearly income – CAD**				
≤$20,000	57 (53%)	38 (57%)	41 (61%)	25 (48%)
**Highest level of education**				
Completed high School/secondary school	6 (6%)	2 (3%)	1 (2%)	1 (2%)
Completed trade/technical school	3 (3%)	3 (4%)	3 (5%)	3 (6%)
Completed college	24 (22%)	19 (24%)	14 (21%)	9 (17%)
Completed university	21 (20%)	16 (20%)	13 (19%)	11 (21%)
Post-graduate education	19 (18%)	16 (20%)	15 (22%)	14 (27%)

LEGEND: Characteristics for participants who completed demographic questionnaire at study enrollment (Time 1; Month 0): [a] excluding HIV.
