# Supplementary material for: Examining the impact of a community-based exercise intervention on cardiorespiratory fitness, cardiovascular health, strength, flexibility and physical activity among adults living with HIV: A three-phased intervention study
Source: PLoS One. 2021 Sep 24;16(9):e0257639. doi: 10.1371/journal.pone.0257639 (PMC8462727; doi:10.1371/journal.pone.0257639)
Supplement: S2 Table — Slopes are change in outcome over one month. a Significant trend in Phase 1 (baseline) slope versus 0; b Significant trend in Phase 2 (intervention) slope versus 0; c Significant change in Phase 3 (follow-up monitoring) slope versus Phase 2 (intervention) slope. d Follow-up slope is the difference in slope between the follow-up and intervention phase; RAPA: Rapid Assessment of Physical Activity Questionnaire (higher scores indicate greater physical activity). (PDF) [file pone.0257639.s005.pdf]

Supplemental File 4 – Results – Secondary Outcomes – Primary (Intention to Treat) Analysis

Cardiorespiratory Outcomes

Resting Heart Rate (beats per minute)

Estimated Intervention Effect over 6 month Intervention Phase:
-0.75 beats per minute (95% CI: -3.97, 2.48)
Number of Observations: 718; Sample size: 102 (91 males; 11 females)

Parameter	Fixed Effects			Random Effects (SD)
	Estimate	95% Confidence Interval	p-value	
Intercept (50yr old male)	77.773	74.857, 80.688	<0.001	13.03
Age Effect	-0.492	-0.733, -0.251	<0.001	--
Phase 1: Baseline slope (change / month)	0.213	-0.054, 0.481	0.118	0.61
Phase 2: Intervention slope (change / month)	0.088	-0.267, 0.444	0.625	<0.01
Phase 3: Difference in Follow-up (Phase 3) and Intervention (Phase 2) slope[d] (change / month)	-0.267	-0.877, 0.344	0.391	0.39
Residual	--	--	--	7.76

Diastolic Blood Pressure (mmHg)

Estimated Intervention Effect over 6 month Intervention Phase:
-0.68 mmHg (95% CI: -3.78, 2.43)
Number of Observations: 724; Sample size: 102 (91 males; 11 females))

Parameter	Fixed Effects			Random Effects (SD)
	Estimate	95% Confidence Interval	p-value	
Intercept (50yr old male)	74.899	72.535, 77.264	<0.001	9.36
Age Effect	0.075	-0.103, 0.254	0.402	--
Sex Effect	5.239	-1.391, 11.869	0.120	--
Phase 1: Baseline slope (change / month)	-0.178	-0.423, 0.068	0.155	0.41
Phase 2: Intervention slope (change / month)	-0.291	-0.638, 0.056	0.100	0.11
Phase 3: Difference in Follow-up (Phase 3) and Intervention (Phase 2) slope[d] (change / month)	0.689	0.111, 1.267	0.020[c]	<0.01
Residual	--	--	--	7.65

Outcome	Parameter	Fixed Effects			Random Effects (SD)
**Systolic Blood Pressure (mmHg)**	**Estimated Intervention Effect over 6 month Intervention Phase:** -5.18 mmHg (95% CI: -9.66, -0.71) Number of Observations: 724; Sample size: 102 (91 males; 11 females)				
	**Parameter**	**Estimate**	**95% Confidence Interval**	**p-value**	
	Intercept (50yr old male)	120.860	117.708, 124.012	<0.001	12.64
	Age Effect	0.275	0.035, 0.515	0.025	--
	Phase 1: Baseline slope (change / month)	0.083	-0.251, 0.417	0.625	<0.01
	Phase 2: Intervention slope (change / month)	-0.781	-1.290, -0.271	0.003[b]	0.35
	Phase 3: Difference in Follow-up (Phase 3) and Intervention (Phase 2) slope[d] (change / month)	1.520	0.674, 2.366	<0.001[c]	0.16
	Residual	--	--	--	11.16

Strength and Flexibility Outcomes

Outcome	Parameter	Fixed Effects			Random Effects (SD)
**Upper Extremity: Grip Strength (kg)**	**Estimated Intervention Effect over 6 month Intervention Phase**: -1.41 kg (95% CI: -3.62, 0.81) Number of Observations: 721; Sample size: 101 (91 males; 10 females)				
	**Parameter**	**Estimate**	**95% Confidence Interval**	**p-value**	
	Intercept (50yr old male)	81.406	(78.015, 84.797)	<0.001	15.78
	Age Effect	-0.412	(-0.697, -0.127)	0.005	--
	Sex Effect	-23.430	(-34.268, -12.591)	<0.001	--
	Phase 1: Baseline slope (change / month)	0.258	(0.046, 0.470)	0.017[a]	0.74
	Phase 2: Intervention slope (change / month)	0.024	(-0.217, 0.264)	0.848	0.43
	Phase 3: Difference in Follow-up (Phase 3) and Intervention (Phase 2) slope[d] (change / month)	-0.002	(-0.369, 0.366)	0.993	0.00
	Residual	--	--	--	4.66

Supplemental File 4 – Results – Secondary Outcomes – Primary (Intention to Treat) Analysis

**Vertical Jump Test (cm)**	**Estimated Intervention Effect over 6 month Intervention Phase**:<br>-3.06 cm (95% CI: -4.57, -1.54)<br>Number of Observations: 656; Sample size: 96 (86 males; 10 females)				
	**Parameter**	**Fixed Effects**			**Random Effects (SD)**
		**Estimate**	**95% Confidence Interval**	**p-value**	
	Intercept (50yr old male)	27.804	(26.228, 29.380)	<0.001	6.75
	Age Effect	-0.441	(-0.569, -0.312)	<0.001	--
	Sex Effect	-11.750	(-16.565, -6.936)	<0.001	--
	Phase 1: Baseline slope (change / month)	0.519	(0.390, 0.648)	<0.001[a]	0.34
	Phase 2: Intervention slope (change / month)	0.010	(-0.158, 0.178)	0.907	0.18
	Phase 3: Difference in Follow-up (Phase 3) and Intervention (Phase 2) slope[d] (change / month)	0.095	(-0.178, 0.368)	0.494	0.00
	Residual	--	--	--	3.31
**Back Extension (seconds)**	**Estimated Intervention Effect over 6 month Intervention Phase**:<br>-6.90 seconds (95% CI: -16.32, 2.52)<br>Number of Observations: 667; Sample size: 97 (87 males; 10 females)				
	**Parameter**	**Fixed Effects**			**Random Effects (SD)**
		**Estimate**	**95% Confidence Interval**	**p-value**	
	Intercept (50yr old male)	78.721	(70.389, 87.054)	<0.001	34.27
	Age Effect	-0.115	(-0.777, 0.548)	0.732	--
	Sex Effect	-21.634	(-46.427, 3.160)	0.086	--
	Phase 1: Baseline slope (change / month)	1.106	(0.301, 1.911)	0.007[a]	2.14
	Phase 2: Intervention slope (change / month)	-0.044	(-1.090, 1.001)	0.934	1.25
	Phase 3: Difference in Follow-up (Phase 3) and Intervention (Phase 2) slope[d] (change / month)	1.288	(-0.388, 2.965)	0.132	0.00
	Residual	--	--	--	20.81

Supplemental File 4 – Results – Secondary Outcomes – Primary (Intention to Treat) Analysis

**Push Ups (number of additional pushups)**	**Estimated Intervention Effect over 6 month Intervention Phase:**<br>2.30 additional pushups (95% CI: 0.69, 3.91)<br>Number of Observations: 695; Sample size: 100 (90 males; 10 females)				
	**Parameter**	**Fixed Effects**			**Random Effects (SD)**
		**Estimate**	**95% Confidence Interval**	**p-value**	
	Intercept (50yr old male)	11.693	(9.908, 13.477)	<0.001	7.93
	Age Effect	-0.156	(-0.303, -0.009)	0.037	--
	Sex Effect	-8.052	(-13.666, -2.438)	0.005	--
	Phase 1: Baseline slope (change / month)	0.041	(-0.112, 0.194)	0.601	0.53
	Phase 2: Intervention slope (change / month)	0.424	(0.250, 0.599)	<0.001[b]	0.30
	Phase 3: Difference in Follow-up (Phase 3) and Intervention (Phase 2) slope[d] (change / month)	-0.232	(-0.524, 0.060)	0.119	0.40
	Residual	--	--	--	3.33
**Curl ups (number of additional pushups)**	**Estimated Intervention Effect over 6 month Intervention Phase**:<br>2.89 additional curl ups (95% CI: 0.61, 5.17)<br>Number of Observations: 688; Sample size: 98 (88 males; 10 females)				
	**Parameter**	**Fixed Effects**			**Random Effects (SD)**
		**Estimate**	**95% Confidence Interval**	**p-value**	
	Intercept (50yr old male)	15.195	(13.198, 17.193)	0.000	8.30
	Age Effect	-0.199	(-0.359, -0.040)	0.015	--
	Sex Effect	-7.776	(-13.804, -1.748)	0.012	--
	Phase 1: Baseline slope (change / month)	-0.075	(-0.270, 0.121)	0.454	0.55
	Phase 2: Intervention slope (change / month)	0.407	(0.150, 0.664)	0.002[b]	0.38
	Phase 3: Difference in Follow-up (Phase 3) and Intervention (Phase 2) slope[d] (change / month)	-0.326	(-0.731, 0.078)	0.114	0.00
	Residual	--	--	--	5.06

Flexibility

**Sit and Reach Test (cm)**	**Estimated Intervention Effect over 6 month Intervention Phase**:<br>1.74 cm (95% CI: 0.21, 3.28)<br>Number of Observations: 698; Sample size: 99 (89 males; 10 females)				
	**Parameter**	**Fixed Effects**			**Random Effects (SD)**
		**Estimate**	**95% Confidence Interval**	**p-value**	
	Intercept (50yr old male)	23.297	(21.143, 25.451)	<0.001	10.34
	Age Effect	-0.110	(-0.298, 0.077)	0.246	--
	Phase 1: Baseline slope (change / month)	0.063	(-0.083, 0.208)	0.398	0.48
	Phase 2: Intervention slope (change / month)	0.353	(0.188, 0.518)	<0.001[b]	0.19
	Phase 3: Difference in Follow-up (Phase 3) and Intervention (Phase 2) slope[d] (change / month)	-0.422	(-0.685, -0.158)	0.002[c]	0.00
	Residual	--	--	--	3.33

Physical Activity Outcomes

**RAPA Aerobic Scale (Range: 1-5)**	**Estimated Intervention Effect over 6 month Intervention Phase**:<br>0.16 (95% CI: -0.03, 0.35)<br>Number of Observations: 805; Sample size: 105 (94 males; 11 females)				
	**Parameter**	**Fixed Effects**			**Random Effects (SD)**
		**Estimate**	**95% Confidence Interval**	**p-value**	
	Intercept (50yr old male)	4.492	4.361, 4.624	<0.001	0.51
	Age Effect	0.001	0.009, 0.011)	0.803	--
	Sex Effect	-0.318	-0.699, 0.06	0.100	--
	Phase 1: Baseline slope (change / month)	0.009	-0.008, 0.025	0.312	0.05
	Phase 2: Intervention slope (change / month)	0.035	0.015, 0.056	0.001[b]	0.01
	Phase 3: Difference in Follow-up (Phase 3) and Intervention (Phase 2) slope[d] (change / month)	-0.049	-0.083, -0.016	0.004[c]	0.03
	Residual	--	--	--	0.47

LEGEND: Slopes are change in outcome over one month. [a] Significant trend in Phase 1 (baseline) slope versus 0; [b] Significant trend in Phase 2 (intervention) slope versus 0; [c] Significant change in Phase 3 (follow-up monitoring) slope versus Phase 2 (intervention) slope. [d] Estimate refers to the difference in slope between in the follow-up and intervention phase; Phase 3 (Follow-Up) slope is the sum of Phase 2 (intervention) slope

and Phase 3 (difference in Follow-Up and Intervention slope). RAPA: Rapid Assessment of Physical Activity Questionnaire (higher scores indicate greater physical activity)
