# Supplementary material for: Examining the impact of a community-based exercise intervention on cardiorespiratory fitness, cardiovascular health, strength, flexibility and physical activity among adults living with HIV: A three-phased intervention study
Source: PLoS One. 2021 Sep 24;16(9):e0257639. doi: 10.1371/journal.pone.0257639 (PMC8462727; doi:10.1371/journal.pone.0257639)
Supplement: S3 Table — aOne of these participants did not have T8 data so is not included in McNemar’s below; bFor strength counts, participants reporting either Strength or Both were combined; c For flexibility counts, participants reporting either Flexibility or Both were combined; d Same number of participants moving from not engaging to engaging in flexibility were the same as the number of participants moving from engaging in flexibility to not; RAPA: Rapid Assessment of Physical Activity Questionnaire. (PDF) [file pone.0257639.s006.pdf]

Supplemental File 5 – RAPA 2 - Strength and Flexibility Scores

RAPA 2 - Strength and Flexibility Score	Immediately Prior to Intervention (Time Point 5) n (%)	Immediately After Intervention (Time Point 8) n (%)	End of Follow-Up Phase / End of Study (Time Point 12) n (%)
No activity	26 (31)	6 (10)	7 (13)
Strength only	7 (8)	5 (8)	2 (4)
Flexibility only	19(23)	2 (3)	6 (12)
Both strength + flexibility	31 (37)	47 (78)	37 (71)
**Total**	83	60	52[a]
Participants Reporting Physical Activity at Both Time Points (T5 and T8) (n=60)			
**Strength[b]**	**End of Intervention**		
Start of Intervention	No Strength	Strength	
No Strength	5	28	
Strength	3	24	
McNemar's ChiSq = 18.6 on 1 df, p < 0.001			
**Flexibility[c]**	**End of Intervention**		
Start of Intervention	No Flexibility	Flexibility	
No Flexibility	7	15	
Flexibility	4	34	
McNemar's ChiSq = 5.3 on 1 df, p = 0.02			
Participants Reporting Physical Activity at Both Time Points (T8 and T12) (n=51)			
**Strength[b]**	**End of Study**		
End of Intervention	No Strength	Strength	
No Strength	1	5	
Strength	12	33	
McNemar's ChiSq = 2.1 on 1 df, p = 0.14			
**Flexibility[c]**	**End of Study**		
End of Intervention	No Flexibility	Flexibility	
No Flexibility	3	6	
Flexibility	6	36	
McNemar's ChiSq = 0 on 1 df, p = 1[d]			

LEGEND: [a] One of these participants did not have T8 data so is not included in McNemar's below.
[b] For strength counts, participants reporting either Strength or Both were combined.
[c] For flexibility counts, participants reporting either Flexibility or Both were combined;
[d] Same number of participants moving from not engaging to engaging in flexibility were the same as the number of participants moving from engaging in flexibility to not.
RAPA: Rapid Assessment of Physical Activity Questionnaire

Date Last Revised: August 16, 2021
