# Supplementary material for: Examining the impact of a community-based exercise intervention on cardiorespiratory fitness, cardiovascular health, strength, flexibility and physical activity among adults living with HIV: A three-phased intervention study
Source: PLoS One. 2021 Sep 24;16(9):e0257639. doi: 10.1371/journal.pone.0257639 (PMC8462727; doi:10.1371/journal.pone.0257639)
Supplement: S4 Table — a Significant overall estimated treatment effect over the six-month intervention after taking the baseline monitoring into account; RAPA: Rapid Assessment of Physical Activity; CI: Confidence Interval; V̇O2peak: peak oxygen consumption. Sample size: Intention to Treat Analysis: Maximum n = 105 participants; Post Hoc Exploratory Per Protocol Analysis: Maximum n = 80 participants; Post Hoc Exploratory Dosage Analysis: Models A and B: Maximum: 45 participants. (PDF) [file pone.0257639.s007.pdf]

Supplemental File 6 – Overall Estimated Effects of the Phase 2 - CBE Intervention after taking the Phase 1 - Baseline Monitoring into account for all outcomes and analyses: Primary Analysis (intention to treat) and Secondary (exploratory, post hoc) Analyses (per protocol and exercise dose analysis)

**Outcome**	**Overall Effect – Intention to Treat Primary Analysis** Estimate (95% CI)	**Post Hoc Analysis Per Protocol Effect** Estimate (95% CI)	**Post Hoc Analysis Model A:** Estimated Effect with Exercise Dose for ≥150 minutes of Moderate + Vigorous Physical Activity in Past week Estimate (95% CI)	**Post Hoc Analysis Model B:** Estimated effect for Exercise Dose of ≥75 minutes of Vigorous Physical Activity in the Past week Estimate (95% CI)
**Cardiorespiratory Fitness**				
$\dot{V}O_2$peak (ml/kg/min)	0.56 (-1.27, 2.39)	1.56 (-0.79, 3.90)	-0.24 (-1.48, 1.01)	-0.26 (-1.68, 1.17)
Resting heart rate (bpm)	-0.75 (-3.97, 2.48)	-2.20 (-6.22, 1.81)	-1.85 (-3.99, 0.28)	-1.97 (-4.00, 0.06)
Resting diastolic blood pressure (mmHg)	-0.68 (-3.78, 2.43)	-0.30 (-4.27, 3.67)	0.37 (-1.52, 2.26)	0.60 (-1.28, 2.47)
Resting systolic blood pressure (mmHg)	-5.18 (-9.66, -0.71)[a]	-5.52 (-10.80, -0.24)[a]	-1.43 (-4.17, 1.31)	-1.25 (-4.04, 1.54)
**Strength and Flexibility**				
Upper extremity - Grip strength (kg)	-1.41 (-3.62, 0.81)	-0.60 (-3.05, 1.86)	-0.45 (-2.11, 1.21)	-0.26 (-2.02, 1.51)
Lower extremity – Vertical jump test (cm)	-3.06 (-4.57, -1.54)	-2.60 (-4.47, -0.73)	-2.79 (-3.93, -1.64)	-2.40 (-3.55, -1.25)
Back extension (seconds)	-6.90 (-16.32, 2.52)	-7.26 (-18.04, 3.52)	-4.35 (-11.46, 2.76)	-5.69 (-12.62, 1.23)
Push ups (number completed)	2.30 (0.69, 3.91)[a]	1.92 (-0.13, 3.98)	-0.05 (-1.71, 1.61)	0.25 (-1.39, 1.89)
Curl ups (number completed)	2.89 (0.61, 5.17)[a]	2.69 (0.01, 5.37)[a]	1.04 (-1.03, 3.12)	0.84 (-1.18, 2.86)
Flexibility – sit and reach test (cm)	1.74 (0.21, 3.28)[a]	2.53 (0.60, 4.47)[a]	0.11 (-1.10, 1.31)	-0.11 (-1.28, 1.06)
**Self-Reported Physical Activity**				
RAPA Aerobic (scale 1-5)	0.16 (-0.03, 0.35)	0.23 (-0.01, 0.46)	-0.04 (-0.17, 0.08)	-0.08 (-0.20, 0.04)

LEGEND: [a] Significant overall estimated treatment effect over the six-month intervention after taking the baseline monitoring into account;
RAPA: Rapid Assessment of Physical Activity; CI: Confidence Interval; $\dot{V}O_2$peak: peak oxygen consumption
Sample size: Intention to Treat Analysis: Maximum n=105 participants; Post Hoc Exploratory Per Protocol Analysis: Maximum n=80 participants; Post Hoc Exploratory Dosage Analysis: Models A and B: Maximum: 45 participants.
