# Supplementary material for: Examining the impact of a community-based exercise intervention on cardiorespiratory fitness, cardiovascular health, strength, flexibility and physical activity among adults living with HIV: A three-phased intervention study
Source: PLoS One. 2021 Sep 24;16(9):e0257639. doi: 10.1371/journal.pone.0257639 (PMC8462727; doi:10.1371/journal.pone.0257639)
Supplement: S5 Table — Slopes are change in outcome over one month; Baseline slope (trend) p value <0.05 = significant difference in baseline slope versus 0; Slope while receiving the intervention (trend) p value <0.05 = significant difference in slope during the intervention versus 0; RAPA: Rapid Assessment of Physical Activity; RAPA Aerobic (range: 1–5); CI: Confidence Interval. (PDF) [file pone.0257639.s008.pdf]

Supplemental File 7 – Post Hoc Exploratory Analyses – Per Protocol - Comparing trends during the baseline monitoring phase (defined as any time prior to the first supervised training session) and the trends while receiving the intervention (defined as the time between the first and last supervised training session)**.**

Cardiorespiratory Fitness Outcomes

Outcome	Parameter	Fixed Effects			Random Effects (SD)
		Estimate	95% Confidence Interval	p-value	
**V̇O$_2$peak (ml/kg/min)**	**Estimated Intervention Effect over six months:** 1.56 ml/kg/min (95% CI: -0.79, 3.90) Number of Observations: 474; Sample size: 79 (72 males; 7 females)				
	Intercept (50yr old male)	25.630	(24.144, 27.115)	0.000	5.45
	Age Effect	-0.163	(-0.281, -0.046)	0.007	--
	Sex Effect	-8.832	(-13.501, -4.164)	0.000	--
	Baseline trend (change / month)	0.101	(-0.040, 0.243)	0.160	0.23
	Trend while receiving the intervention (change / month)	0.361	(0.051, 0.670)	0.022	0.58
	Residual	--	--	--	4.26
**Resting Heart Rate (beats per minute)**	**Estimated Intervention Effect over six months:** -2.20 beats per minute (95% CI: -6.22, 1.81) Number of Observations: 488; Sample size: 80 (73 males; 7 females)				
	Intercept (50yr old male)	77.052	(74.511, 79.593)	0.000	9.85
	Age Effect	-0.431	(-0.638, -0.223)	0.000	--
	Baseline trend (change / month)	0.285	(0.046, 0.525)	0.020	0.31
	Trend while receiving the intervention (change / month)	-0.082	(-0.611, 0.447)	0.761	0.98
	Residual	--	--	--	7.57

Supplemental File 7 – Post Hoc Exploratory Analyses – Per Protocol - Comparing trends during the baseline monitoring phase (defined as any time prior to the first supervised training session) and the trends while receiving the intervention (defined as the time between the first and last supervised training session)**.**

**Diastolic Blood Pressure (mmHg)**	**Estimated Intervention Effect over six months:** -0.30 mmHg (95% CI: -4.27, 3.67)<br>Number of Observations: 491; Sample size: 80 (73 males; 7 females)				
	**Parameter**	**Fixed Effects**			**Random Effects (SD)**
		**Estimate**	**95% Confidence Interval**	**p-value**	
	Intercept (50yr old male)	75.345	(73.023, 77.668)	0.000	8.27
	Age Effect	0.055	(-0.124, 0.234)	0.540	--
	Sex Effect	3.165	(-3.952, 10.282)	0.379	--
	Baseline trend (change / month)	-0.202	(-0.428, 0.024)	0.079	0.00
	Trend while receiving the intervention (change / month)	-0.253	(-0.781, 0.276)	0.348	1.03
	Residual	--	--	--	7.47
**Systolic Blood Pressure (mmHg)**	**Estimated Intervention Effect over six months:** -5.52 mmHg (95% CI: -10.80, -0.24)<br>Number of Observations: 491; Sample size: 80 (73 males; 7 females)				
	**Parameter**	**Fixed Effects**			**Random Effects (SD)**
		**Estimate**	**95% Confidence Interval**	**p-value**	
	Intercept (50yr old male)	121.325	(118.100, 124.550)	0.000	12.06
	Age Effect	0.320	(0.064, 0.576)	0.015	--
	Baseline trend (change / month)	0.058	(-0.261, 0.376)	0.722	0.00
	Trend while receiving the intervention (change / month)	-0.862	(-1.543, -0.181)	0.013	0.97
	Residual	--	--	--	10.55

Supplemental File 7 – Post Hoc Exploratory Analyses – Per Protocol - Comparing trends during the baseline monitoring phase (defined as any time prior to the first supervised training session) and the trends while receiving the intervention (defined as the time between the first and last supervised training session)**.**

Strength and Flexibility Outcomes

Outcome	Parameter	Fixed Effects: Estimate	Fixed Effects: 95% Confidence Interval	Fixed Effects: p-value	Random Effects (SD)
**Upper Extremity - Grip Strength (kg)**	**Estimated Intervention Effect over six months:** -0.60 kg (95% CI: -3.05, 1.86)<br>Number of Observations: 493; Sample size: 80 (73 males; 7 females)				
	Intercept (50yr old male)	82.425	(79.235, 85.615)	0.000	13.29
	Age Effect	-0.481	(-0.754, -0.208)	0.001	--
	Sex Effect	-24.289	(-35.101, -13.478)	0.000	--
	Baseline trend (change / month)	0.281	(0.093, 0.470)	0.004	0.55
	Trend while receiving the intervention (change / month)	0.182	(-0.116, 0.480)	0.232	0.28
	Residual	--	--	--	4.74
**Lower Extremity - Vertical Jump Test (cm)**	**Estimated Intervention Effect over six months:** -2.60 cm (95% CI: -4.47, -0.73)<br>Number of Observations: 448; Sample size: 76 (70 males; 6 females)				
	Intercept (50yr old male)	28.766	(27.231, 30.300)	0.000	5.92
	Age Effect	-0.534	(-0.664, -0.403)	0.000	--
	Sex Effect	-12.687	(-18.052, -7.321)	0.000	--
	Baseline trend (change / month)	0.472	(0.347, 0.596)	0.000	0.28
	Trend while receiving the intervention (change / month)	0.038	(-0.201, 0.277)	0.753	0.33
	Residual	--	--	--	3.36

Supplemental File 7 – Post Hoc Exploratory Analyses – Per Protocol - Comparing trends during the baseline monitoring phase (defined as any time prior to the first supervised training session) and the trends while receiving the intervention (defined as the time between the first and last supervised training session)**.**

<table>
<tr><td rowspan="9">Back Extension (seconds)</td><td colspan="5">Estimated Intervention Effect over six months: -7.26 sec (95% CI: -18.04, 3.52)
Number of Observations: 458; Sample size: 77 (70 males; 7 females)</td></tr>
<tr><td rowspan="2">Parameter</td><td colspan="3">Fixed Effects</td><td rowspan="2">Random Effects (SD)</td></tr>
<tr><td>Estimate</td><td>95% Confidence Interval</td><td>p-value</td></tr>
<tr><td>Intercept (50yr old male)</td><td>80.930</td><td>(71.887, 89.973)</td><td>0.000</td><td>34.42</td></tr>
<tr><td>Age Effect</td><td>-0.155</td><td>(-0.898, 0.588)</td><td>0.678</td><td>--</td></tr>
<tr><td>Sex Effect</td><td>-16.614</td><td>(-45.369, 12.140)</td><td>0.253</td><td>--</td></tr>
<tr><td>Baseline trend (change / month)</td><td>1.043</td><td>(0.284, 1.802)</td><td>0.007</td><td>1.55</td></tr>
<tr><td>Trend while receiving the intervention (change / month)</td><td>-0.167</td><td>(-1.485, 1.151)</td><td>0.804</td><td>0.00</td></tr>
<tr><td>Residual</td><td>--</td><td>--</td><td>--</td><td>21.23</td></tr>
<tr><td rowspan="9">Push Ups (number completed)</td><td colspan="5">Estimated Intervention Effect over six months: 1.92 additional push ups in 1 minute (95% CI: -0.13, 3.98)
Number of Observations: 471; Sample size: 79 (72 males; 7 females)</td></tr>
<tr><td rowspan="2">Parameter</td><td colspan="3">Fixed Effects</td><td rowspan="2">Random Effects (SD)</td></tr>
<tr><td>Estimate</td><td>95% Confidence Interval</td><td>p-value</td></tr>
<tr><td>Intercept (50yr old male)</td><td>12.679</td><td>(10.697, 14.662)</td><td>0.000</td><td>8.02</td></tr>
<tr><td>Age Effect</td><td>-0.217</td><td>(-0.385, -0.049)</td><td>0.012</td><td>--</td></tr>
<tr><td>Sex Effect</td><td>-8.910</td><td>(-15.528, -2.292)</td><td>0.009</td><td>--</td></tr>
<tr><td>Baseline trend (change / month)</td><td>0.054</td><td>(-0.097, 0.204)</td><td>0.483</td><td>0.46</td></tr>
<tr><td>Trend while receiving the intervention (change / month)</td><td>0.374</td><td>(0.110, 0.639)</td><td>0.006</td><td>0.54</td></tr>
<tr><td>Residual</td><td>--</td><td>--</td><td>--</td><td>3.46</td></tr>
</table>

Supplemental File 7 – Post Hoc Exploratory Analyses – Per Protocol - Comparing trends during the baseline monitoring phase (defined as any time prior to the first supervised training session) and the trends while receiving the intervention (defined as the time between the first and last supervised training session)**.**

**Curl Ups (number completed)**	**Estimated Intervention Effect over six months:** 2.69 additional curl ups in 1 minute (95% CI: 0.01, 5.37)<br>Number of Observations: 469; Sample size: 78 (71 males; 7 females)				
	**Parameter**	**Fixed Effects**			**Random Effects (SD)**
		**Estimate**	**95% Confidence Interval**	**p-value**	
	Intercept (50yr old male)	15.636	(13.542, 17.730)	0.000	7.91
	Age Effect	-0.297	(-0.468, -0.125)	0.001	--
	Sex Effect	-11.589	(-18.265, -4.914)	0.001	--
	Baseline trend (change / month)	-0.054	(-0.257, 0.149)	0.602	0.54
	Trend while receiving the intervention (change / month)	0.395	(0.071, 0.718)	0.017	0.00
	Residual	--	--	--	5.27
**Flexibility – Sit and Reach Test (cm)**	**Estimated Intervention Effect over six months:** 2.53 cm (95% CI: 0.60, 4.47)<br>Number of Observations: 477; Sample size: 79 (72 males; 7 females)				
	**Parameter**	**Fixed Effects**			**Random Effects (SD)**
		**Estimate**	**95% Confidence Interval**	**p-value**	
	Intercept (50yr old male)	23.276	(20.865, 25.688)	0.000	10.43
	Age Effect	-0.116	(-0.328, 0.096)	0.280	--
	Baseline trend (change / month)	0.044	(-0.097, 0.186)	0.536	0.35
	Trend while receiving the intervention (change / month)	0.466	(0.231, 0.702)	0.000	0.00
	Residual	--	--	--	3.85

Supplemental File 7 – Post Hoc Exploratory Analyses – Per Protocol - Comparing trends during the baseline monitoring phase (defined as any time prior to the first supervised training session) and the trends while receiving the intervention (defined as the time between the first and last supervised training session)**.**

Self-Reported Physical Activity

	**Estimated Intervention Effect over six months:** 0.23 points (95% CI: -0.01, 0.46)<br>Number of Observations: 486; Sample size: 73 (73 males; 7 females)				
	**Parameter**	**Fixed Effects**			**Random Effects (SD)**
		**Estimate**	**95% Confidence Interval**	**p-value**	
**RAPA Aerobic (points)**	Intercept (50yr old male)	4.569	(4.439, 4.700)	0.000	0.43
	Age Effect	0.003	(-0.006, 0.013)	0.500	--
	Sex Effect	-0.300	(-0.689, 0.090)	0.130	--
	Baseline slope (change / month)	0.008	(-0.009, 0.025)	0.365	0.04
	Slope while receiving the intervention (change / month)	0.046	(0.017, 0.074)	0.002	0.00
	Residual	--	--	--	0.48

LEGEND: Slopes are change in outcome over one month; Baseline slope (trend) p value <0.05 = significant difference in baseline slope versus 0; Slope while receiving the intervention (trend) p value <0.05 = significant difference in slope during the intervention versus 0;
RAPA: Rapid Assessment of Physical Activity; RAPA Aerobic (range: 1-5); CI: Confidence Interval
