# Supplementary material for: Examining the impact of a community-based exercise intervention on cardiorespiratory fitness, cardiovascular health, strength, flexibility and physical activity among adults living with HIV: A three-phased intervention study
Source: PLoS One. 2021 Sep 24;16(9):e0257639. doi: 10.1371/journal.pone.0257639 (PMC8462727; doi:10.1371/journal.pone.0257639)
Supplement: S6 Table — Model A: Dose defined as total number of Moderate + Vigorous minutes of activity in the past week; Model B: Dose defined as total number of Vigorous minutes of activity in the past week. Canadian Physical Activity Guidelines (CPAG): Model A: ≥150 minutes moderate to vigorous / week and Model B: ≥75 minutes vigorous activity. Slopes are change in outcome over one month. aFollow-up slope is the difference in slope between the follow-up and intervention phase; Phase 1: Baseline slope p value <0.05: statistically significant difference in Phase 1 (baseline) slope versus 0; Phase 2: Intervention slope p value <0.05: statistically significant difference in Phase 2 (intervention) slope versus 0; Phase 3: Follow-up slope p value <0.05: statistically significant change in Phase 3 (follow-up monitoring) slope versus Phase 2 (intervention) slope. (PDF) [file pone.0257639.s009.pdf]

**Supplemental File 8 – Post Hoc Exploratory Analyses – Exercise Dose**

Interaction of exercise dose and duration of the intervention on all outcomes; **Model A**: Dose defined as total number of Moderate + Vigorous minutes of activity in the past week; **Model B**: Dose defined as total number of Vigorous minutes of activity in the past week.

Canadian Physical Activity Guidelines (CPAG): **Model A:** ≥150 minutes moderate to vigorous / week and **Model B**: ≥75 minutes vigorous activity.

## Cardiorespiratory Fitness Outcomes

**Model A - Estimated Intervention Effect over 6 month Intervention Phase with dosage of ≥150 min/week of moderate to vigorous activity:** -0.24 ml/kg/min (95% CI: -1.48, 1.01); Number of Observations: 425; Sample size: 45 (41 males; 4 females)

| Outcome | Parameter | Fixed Effects | | | Random Effects (SD) |
|---|---|---|---|---|---|
| | | Estimate | 95% Confidence Interval | p-value | |
| **Model A**: $\dot{V}O_2$peak (ml/kg/min) | Intercept (50yr old male) | 27.054 | (25.040, 29.067) | 0.000 | 5.64 |
| | Age Effect | -0.171 | (-0.347, 0.005) | 0.056 | -- |
| | Sex Effect | -11.676 | (-18.286, -5.065) | 0.001 | -- |
| | Phase 1: Baseline slope (change / month) | 0.159 | (-0.010, 0.328) | 0.065 | 0.28 |
| | Phase 2: Intervention slope (change / month) | 0.001 | (0.000, 0.001) | 0.006 | 0.00 |
| | Phase 3: Difference in Follow-up (Phase 3) and Intervention (Phase 2) slope[a] (change / month) | -0.410 | (-0.670, -0.149) | 0.002 | 0.29 |
| | Residual | -- | -- | -- | 4.13 |

**Model B - Estimated Intervention Effect over 6 month Intervention Phase with dosage of ≥75 min/week of vigorous activity:**
-0.26 ml/kg/min (95% CI: -1.68, 1.17)
Number of Observations: 425; Sample size: 45 (41 males; 4 females)

| Outcome | Parameter | Fixed Effects | | | Random Effects (SD) |
|---|---|---|---|---|---|
| | | Estimate | 95% Confidence Interval | p-value | |
| **Model B**: $\dot{V}O_2$peak (ml/kg/min) | Intercept (50yr old male) | 27.168 | (25.133, 29.203) | 0.000 | 5.73 |
| | Age Effect | -0.149 | (-0.328, 0.029) | 0.099 | -- |
| | Sex Effect | -11.693 | (-18.435, -4.950) | 0.001 | -- |
| | Phase 1: Baseline slope (change / month) | 0.187 | (0.008, 0.367) | 0.041 | 0.36 |
| | Phase 2: Intervention slope (change / month) | 0.002 | (0.000, 0.004) | 0.048 | 0.00 |
| | Phase 3: Difference in Follow-up (Phase 3) and Intervention (Phase 2) slope[a] (change / month) | -0.281 | (-0.508, -0.055) | 0.015 | 0.22 |
| | Residual | -- | -- | -- | 4.11 |

**Supplemental File 8 – Post Hoc Exploratory Analyses – Exercise Dose**

Interaction of exercise dose and duration of the intervention on all outcomes; **Model A**: Dose defined as total number of Moderate + Vigorous minutes of activity in the past week; **Model B**: Dose defined as total number of Vigorous minutes of activity in the past week.

Canadian Physical Activity Guidelines (CPAG): **Model A:** ≥150 minutes moderate to vigorous / week and **Model B**: ≥75 minutes vigorous activity.

| | **Model A - Estimated Intervention Effect over 6 month Intervention Phase with dosage of ≥150 min/week of moderate to vigorous activity:** -1.85 beats per minute (95% CI: -3.99, 0.28)<br>Number of Observations: 443; Sample size: 45 (41 males; 4 females) | | | | |
|---|---|---|---|---|---|
| **Model A: Resting Heart Rate (beats per minute)** | **Parameter** | **Fixed Effects** | | | **Random Effects (SD)** |
| | | **Estimate** | **95% Confidence Interval** | **p-value** | |
| | Intercept (50yr old male) | 76.070 | (72.685, 79.455) | 0.000 | 10.25 |
| | Age Effect | -0.485 | (-0.787, -0.183) | 0.002 | -- |
| | Phase 1: Baseline slope (change / month) | 0.282 | (-0.030, 0.595) | 0.077 | 0.55 |
| | Phase 2: Intervention slope (change / month) | 0.000 | (-0.001, 0.001) | 0.637 | 0.00 |
| | Phase 3: Difference in Follow-up (Phase 3) and Intervention (Phase 2) slope[a] (change / month) | -0.003 | (-0.411, 0.405) | 0.989 | 0.34 |
| | Residual | -- | -- | -- | 7.79 |

| | **Model B - Estimated Intervention Effect over 6 month Intervention Phase with dosage of ≥75 min/week of vigorous activity:**<br>-1.97 beats per minutes (95% CI: -4.00, 0.06)<br>Number of Observations: 443; Sample size: 45 (41 males; 4 females) | | | | |
|---|---|---|---|---|---|
| **Model B: Resting Heart Rate (beats per minute)** | **Parameter** | **Fixed Effects** | | | **Random Effects (SD)** |
| | | **Estimate** | **95% Confidence Interval** | **p-value** | |
| | Intercept (50yr old male) | 76.077 | (72.718, 79.437) | 0.000 | 10.22 |
| | Age Effect | -0.485 | (-0.786, -0.184) | 0.002 | -- |
| | Phase 1: Baseline slope (change / month) | 0.284 | (-0.024, 0.591) | 0.071 | 0.56 |
| | Phase 2: Intervention slope (change / month) | -0.001 | (-0.002, 0.001) | 0.409 | 0.00 |
| | Phase 3: Difference in Follow-up (Phase 3) and Intervention (Phase 2) slope[a] (change / month) | 0.002 | (-0.339, 0.343) | 0.990 | 0.30 |
| | Residual | -- | -- | -- | 7.79 |

**Supplemental File 8 – Post Hoc Exploratory Analyses – Exercise Dose**
Interaction of exercise dose and duration of the intervention on all outcomes; **Model A**: Dose defined as total number of Moderate + Vigorous minutes of activity in the past week; **Model B**: Dose defined as total number of Vigorous minutes of activity in the past week.
Canadian Physical Activity Guidelines (CPAG): **Model A:** ≥150 minutes moderate to vigorous / week and **Model B**: ≥75 minutes vigorous activity.

| | **Model A - Estimated Intervention Effect over 6 month Intervention Phase with dosage of ≥150 min/week of moderate to vigorous activity:** 0.37 mmHg (95% CI: -1.52, 2.26)<br>Number of Observations: 448; Sample size: 45 (41 males; 4 females) | | | | |
|---|---|---|---|---|---|
| | **Parameter** | **Fixed Effects** | | | **Random Effects (SD)** |
| | | **Estimate** | **95% Confidence Interval** | **p-value** | |
| **Model A: Diastolic Blood Pressure (mmHg)** | Intercept (50yr old male) | 74.638 | (71.318, 77.959) | 0.000 | 9.26 |
| | Age Effect | 0.101 | (-0.188, 0.389) | 0.484 | -- |
| | Sex Effect | 5.030 | (-5.768, 15.828) | 0.353 | -- |
| | Phase 1: Baseline slope (change / month) | -0.192 | (-0.469, 0.084) | 0.172 | 0.33 |
| | Phase 2: Intervention slope (change / month) | -0.001 | (-0.002, 0.000) | 0.009 | 0.00 |
| | Phase 3: Difference in Follow-up (Phase 3) and Intervention (Phase 2) slope[a] (change / month) | 0.577 | (0.214, 0.940) | 0.002 | 0.00 |
| | Residual | -- | -- | -- | 7.64 |

| | **Model B - Estimated Intervention Effect over 6 month Intervention Phase with dosage of ≥75 min/week of vigorous activity:** 0.60 mmHg (95% CI: -1.28, 2.47)<br>Number of Observations: 448; Sample size: 45 (41 males; 4 females) | | | | |
|---|---|---|---|---|---|
| | **Parameter** | **Fixed Effects** | | | **Random Effects (SD)** |
| | | **Estimate** | **95% Confidence Interval** | **p-value** | |
| **Model B: Diastolic Blood Pressure (mmHg)** | Intercept (50yr old male) | 74.456 | (71.193, 77.718) | 0.000 | 9.12 |
| | Age Effect | 0.085 | (-0.199, 0.370) | 0.548 | -- |
| | Sex Effect | 5.186 | (-5.476, 15.848) | 0.332 | -- |
| | Phase 1: Baseline slope (change / month) | -0.228 | (-0.511, 0.056) | 0.115 | 0.43 |
| | Phase 2: Intervention slope (change / month) | -0.002 | (-0.003, 0.000) | 0.016 | 0.00 |
| | Phase 3: Difference in Follow-up (Phase 3) and Intervention (Phase 2) slope[a] (change / month) | 0.447 | (0.141, 0.753) | 0.004 | 0.00 |
| | Residual | -- | -- | -- | 7.61 |

**Supplemental File 8 – Post Hoc Exploratory Analyses – Exercise Dose**
Interaction of exercise dose and duration of the intervention on all outcomes; **Model A**: Dose defined as total number of Moderate + Vigorous minutes of activity in the past week; **Model B**: Dose defined as total number of Vigorous minutes of activity in the past week.
Canadian Physical Activity Guidelines (CPAG): **Model A:** ≥150 minutes moderate to vigorous / week and **Model B**: ≥75 minutes vigorous activity.

| | **Model A - Estimated Intervention Effect over 6 month Intervention Phase with dosage of ≥150 min/week of moderate to vigorous activity:** -1.43 mmHg (95% CI: -4.17, 1.31)<br>Number of Observations: 448; Sample size: 45 (41 males; 4 females) | | | | |
|---|---|---|---|---|---|
| | **Parameter** | **Fixed Effects** | | | **Random Effects (SD)** |
| | | **Estimate** | **95% Confidence Interval** | **p-value** | |
| **Model A: Systolic Blood Pressure (mmHg)** | Intercept (50yr old male) | 120.762 | (116.093, 125.432) | 0.000 | 14.03 |
| | Age Effect | 0.395 | (-0.018, 0.808) | 0.060 | -- |
| | Phase 1: Baseline slope (change / month) | 0.017 | (-0.372, 0.405) | 0.932 | 0.00 |
| | Phase 2: Intervention slope (change / month) | -0.001 | (-0.003, 0.000) | 0.006 | 0.00 |
| | Phase 3: Difference in Follow-up (Phase 3) and Intervention (Phase 2) slope[a] (change / month) | 0.807 | (0.215, 1.400) | 0.008 | 0.59 |
| | Residual | -- | -- | -- | 11.41 |

| | **Model B - Estimated Intervention Effect over 6 month Intervention Phase with dosage of ≥75 min/week of vigorous activity:** -1.25 mmHg (95%CI: -4.04, 1.54)<br>Number of Observations: 448; Sample size: 45 (41 males; 4 females) | | | | |
|---|---|---|---|---|---|
| | **Parameter** | **Fixed Effects** | | | **Random Effects (SD)** |
| | | **Estimate** | **95% Confidence Interval** | **p-value** | |
| **Model B: Systolic Blood Pressure (mmHg)** | Intercept (50yr old male) | 120.462 | (115.818, 125.105) | 0.000 | 13.99 |
| | Age Effect | 0.376 | (-0.035, 0.788) | 0.072 | -- |
| | Phase 1: Baseline slope (change / month) | -0.039 | (-0.422, 0.344) | 0.841 | 0.00 |
| | Phase 2: Intervention slope (change / month) | -0.003 | (-0.006, 0.000) | 0.035 | 0.00 |
| | Phase 3: Difference in Follow-up (Phase 3) and Intervention (Phase 2) slope[a] (change / month) | 0.601 | (0.070, 1.133) | 0.027 | 0.55 |
| | Residual | -- | -- | -- | 11.42 |

**Supplemental File 8 – Post Hoc Exploratory Analyses – Exercise Dose**

Interaction of exercise dose and duration of the intervention on all outcomes; **Model A**: Dose defined as total number of Moderate + Vigorous minutes of activity in the past week; **Model B**: Dose defined as total number of Vigorous minutes of activity in the past week.

Canadian Physical Activity Guidelines (CPAG): **Model A:** ≥150 minutes moderate to vigorous / week and **Model B**: ≥75 minutes vigorous activity.

## Strength and Flexibility Outcomes

| Outcome | Parameter | Fixed Effects | | | Random Effects (SD) |
|---|---|---|---|---|---|
| | **Model A - Estimated Intervention Effect over 6 month Intervention Phase with dosage of ≥150 min/week of moderate to vigorous activity:** -0.45 kg (95% CI: -2.11, 1.21); Number of Observations: 446; Sample size: 45 (41 males; 4 females) | | | | |
| | **Parameter** | **Estimate** | **95% Confidence Interval** | **p-value** | **Random Effects (SD)** |
| **Model A: Upper Extremity - Grip Strength (kg)** | Intercept (50yr old male) | 84.129 | (80.134, 88.124) | 0.000 | 11.72 |
| | Age Effect | -0.602 | (-0.958, -0.246) | 0.001 | -- |
| | Sex Effect | -35.203 | (-48.489, -21.917) | 0.000 | -- |
| | Phase 1: Baseline slope (change / month) | 0.134 | (-0.105, 0.373) | 0.270 | 0.56 |
| | Phase 2: Intervention slope (change / month) | 0.000 | (0.000, 0.001) | 0.202 | 0.00 |
| | Phase 3: Difference in Follow-up (Phase 3) and Intervention (Phase 2) slope[a] (change / month) | -0.156 | (-0.516, 0.203) | 0.393 | 0.65 |
| | Residual | -- | -- | -- | 4.90 |
| | **Model B - Estimated Intervention Effect over 6 month Intervention Phase with dosage of ≥75 min/week of vigorous activity:** -0.26 kg (95% CI: -2.02, 1.51) Number of Observations: 446; Sample size: 45 (41 males; 4 females) | | | | |
| | **Parameter** | **Estimate** | **95% Confidence Interval** | **p-value** | **Random Effects (SD)** |
| **Model B: Upper Extremity - Grip Strength (kg)** | Intercept (50yr old male) | 84.096 | (80.111, 88.082) | 0.000 | 11.70 |
| | Age Effect | -0.597 | (-0.952, -0.241) | 0.002 | -- |
| | Sex Effect | -35.038 | (-48.317, -21.758) | 0.000 | -- |
| | Phase 1: Baseline slope (change / month) | 0.134 | (-0.101, 0.369) | 0.263 | 0.55 |
| | Phase 2: Intervention slope (change / month) | 0.001 | (-0.001, 0.003) | 0.259 | 0.00 |
| | Phase 3: Difference in Follow-up (Phase 3) and Intervention (Phase 2) slope[a] (change / month) | -0.125 | (-0.440, 0.189) | 0.435 | 0.54 |
| | Residual | -- | -- | -- | 4.89 |

**Supplemental File 8 – Post Hoc Exploratory Analyses – Exercise Dose**

Interaction of exercise dose and duration of the intervention on all outcomes; **Model A**: Dose defined as total number of Moderate + Vigorous minutes of activity in the past week; **Model B**: Dose defined as total number of Vigorous minutes of activity in the past week.

Canadian Physical Activity Guidelines (CPAG): **Model A:** ≥150 minutes moderate to vigorous / week and **Model B**: ≥75 minutes vigorous activity.

| | **Model A - Estimated Intervention Effect over 6 month Intervention Phase with dosage of ≥150 min/week of moderate to vigorous activity:** -2.79 cm (95% CI: -3.93, -1.64)<br>Number of Observations: 410; Sample size: 43 (39 males; 4 females) | | | | |
|---|---|---|---|---|---|
| | **Parameter** | **Fixed Effects** | | | **Random Effects (SD)** |
| | | **Estimate** | **95% Confidence Interval** | **p-value** | |
| **Model A: Lower Extremity - Vertical Jump Test (cm)** | Intercept (50yr old male) | 29.076 | (27.166, 30.985) | 0.000 | 5.39 |
| | Age Effect | -0.570 | (-0.739, -0.402) | 0.000 | -- |
| | Sex Effect | -14.582 | (-20.797, -8.367) | 0.000 | -- |
| | Phase 1: Baseline slope (change / month) | 0.490 | (0.328, 0.653) | 0.000 | 0.36 |
| | Phase 2: Intervention slope (change / month) | 0.000 | (0.000, 0.001) | 0.455 | 0.00 |
| | Phase 3: Difference in Follow-up (Phase 3) and Intervention (Phase 2) slope[a] (change / month) | 0.139 | (-0.059, 0.337) | 0.167 | 0.13 |
| | Residual | -- | -- | -- | 3.30 |

| | **Model B - Estimated Intervention Effect over 6 month Intervention Phase with dosage of ≥75 min/week of vigorous activity:** -2.40 cm (95% CI: -3.55, -1.25)<br>Number of Observations: 410; Sample size: 43 (39 males; 4 females) | | | | |
|---|---|---|---|---|---|
| | **Parameter** | **Fixed Effects** | | | **Random Effects (SD)** |
| | | **Estimate** | **95% Confidence Interval** | **p-value** | |
| **Model B: Lower Extremity - Vertical Jump Test (cm)** | Intercept (50yr old male) | 29.000 | (27.101, 30.899) | 0.000 | 5.38 |
| | Age Effect | -0.571 | (-0.739, -0.403) | 0.000 | -- |
| | Sex Effect | -14.532 | (-20.743, -8.321) | 0.000 | |
| | Phase 1: Baseline slope (change / month) | 0.479 | (0.318, 0.639) | 0.000 | 0.37 |
| | Phase 2: Intervention slope (change / month) | 0.001 | (0.000, 0.002) | 0.088 | 0.00 |
| | Phase 3: Difference in Follow-up (Phase 3) and Intervention (Phase 2) slope[a] (change / month) | 0.100 | (-0.064, 0.264) | 0.230 | 0.08 |
| | Residual | -- | -- | -- | 3.29 |

**Supplemental File 8 – Post Hoc Exploratory Analyses – Exercise Dose**
Interaction of exercise dose and duration of the intervention on all outcomes; **Model A**: Dose defined as total number of Moderate + Vigorous minutes of activity in the past week; **Model B**: Dose defined as total number of Vigorous minutes of activity in the past week.
Canadian Physical Activity Guidelines (CPAG): **Model A:** ≥150 minutes moderate to vigorous / week and **Model B**: ≥75 minutes vigorous activity.

**Supplemental File 8 – Post Hoc Exploratory Analyses – Exercise Dose**

Interaction of exercise dose and duration of the intervention on all outcomes; **Model A**: Dose defined as total number of Moderate + Vigorous minutes of activity in the past week; **Model B**: Dose defined as total number of Vigorous minutes of activity in the past week.
Canadian Physical Activity Guidelines (CPAG): **Model A:** ≥150 minutes moderate to vigorous / week and **Model B**: ≥75 minutes vigorous activity.

| | **Model A - Estimated Intervention Effect over 6 month Intervention Phase with dosage of ≥150 min/week of moderate to vigorous activity:** -4.35 sec (95% CI: -11.46, 2.76)<br>Number of Observations: 414; Sample size: 43 (39 males; 4 females) | | | | |
|---|---|---|---|---|---|
| | **Parameter** | **Fixed Effects** | | | **Random Effects (SD)** |
| | | **Estimate** | **95% Confidence Interval** | **p-value** | |
| **Model A: Back Extension (seconds)** | Intercept (50yr old male) | 80.134 | (67.228, 93.039) | 0.000 | 36.15 |
| | Age Effect | 0.207 | (-0.920, 1.334) | 0.712 | -- |
| | Sex Effect | -18.076 | (-59.823, 23.671) | 0.387 | -- |
| | Phase 1: Baseline slope (change / month) | 1.071 | (0.065, 2.077) | 0.037 | 1.94 |
| | Phase 2: Intervention slope (change / month) | 0.002 | (0.000, 0.005) | 0.103 | 0.00 |
| | Phase 3: Follow-up slope[a] (change / month) | 0.598 | (-0.723, 1.919) | 0.374 | 1.05 |
| | Residual | -- | -- | -- | 22.59 |

| | **Model B - Estimated Intervention Effect over 6 month Intervention Phase with dosage of ≥75 min/week of vigorous activity:**<br>-5.69 sec (95% CI: -12.62, 1.23)<br>Number of Observations: 414; Sample size: 43 (39 males; 4 females) | | | | |
|---|---|---|---|---|---|
| | **Parameter** | **Fixed Effects** | | | **Random Effects (SD)** |
| | | **Estimate** | **95% Confidence Interval** | **p-value** | |
| **Model B: Back Extension (seconds)** | Intercept (50yr old male) | 80.892 | (68.138, 93.647) | 0.000 | 35.81 |
| | Age Effect | 0.235 | (-0.881, 1.351) | 0.673 | -- |
| | Sex Effect | -18.497 | (-59.878, 22.885) | 0.372 | -- |
| | Phase 1: Baseline slope (change / month) | 1.193 | (0.186, 2.201) | 0.020 | 2.01 |
| | Phase 2: Intervention slope (change / month) | 0.003 | (-0.003, 0.009) | 0.294 | 0.00 |
| | Phase 3: Follow-up slope[a] (change / month) | 1.089 | (-0.041, 2.218) | 0.059 | 1.26 |
| | Residual | -- | -- | -- | 22.69 |

**Supplemental File 8 – Post Hoc Exploratory Analyses – Exercise Dose**
Interaction of exercise dose and duration of the intervention on all outcomes; **Model A**: Dose defined as total number of Moderate + Vigorous minutes of activity in the past week; **Model B**: Dose defined as total number of Vigorous minutes of activity in the past week.
Canadian Physical Activity Guidelines (CPAG): **Model A:** ≥150 minutes moderate to vigorous / week and **Model B**: ≥75 minutes vigorous activity.

| | | | | | |
|---|---|---|---|---|---|
| | **Model A - Estimated Intervention Effect over 6 month Intervention Phase with dosage of ≥150 min/week of moderate to vigorous activity:** -0.05 additional push ups (95% CI: -1.71, 1.61)<br>Number of Observations: 436; Sample size: 45 (41 males; 4 females) | | | | |
| | **Parameter** | **Fixed Effects** | | | **Random Effects (SD)** |
| | | **Estimate** | **95% Confidence Interval** | **p-value** | |
| **Model A: Push Ups (number completed)** | Intercept (50yr old male) | 14.824 | (11.789, 17.860) | 0.000 | 8.87 |
| | Age Effect | -0.236 | (-0.506, 0.034) | 0.085 | -- |
| | Sex Effect | -11.675 | (-21.759, -1.591) | 0.024 | -- |
| | Phase 1: Baseline slope (change / month) | 0.139 | (-0.094, 0.372) | 0.242 | 0.65 |
| | Phase 2: Intervention slope (change / month) | 0.001 | (0.000, 0.002) | 0.016 | 0.00 |
| | Phase 3: Follow-up slope[a] (change / month) | 0.121 | (-0.166, 0.409) | 0.407 | 0.47 |
| | Residual | -- | -- | -- | 3.60 |
| | **Model B - Estimated Intervention Effect over 6 month Intervention Phase with dosage of ≥75 min/week of vigorous activity:**<br>0.25 additional push ups (95% CI: -1.39, 1.89)<br>Number of Observations: 436; Sample size: 45 (41 males; 4 females) | | | | |
| | **Parameter** | **Fixed Effects** | | | **Random Effects (SD)** |
| | | **Estimate** | **95% Confidence Interval** | **p-value** | |
| **Model B: Push Ups (number completed)** | Intercept (50yr old male) | 14.866 | (11.835, 17.897) | 0.000 | 8.89 |
| | Age Effect | -0.236 | (-0.507, 0.034) | 0.085 | -- |
| | Sex Effect | -11.726 | (-21.828, -1.623) | 0.024 | -- |
| | Phase 1: Baseline slope (change / month) | 0.145 | (-0.085, 0.375) | 0.216 | 0.65 |
| | Phase 2: Intervention slope (change / month) | 0.002 | (0.001, 0.004) | 0.004 | 0.00 |
| | Phase 3: Follow-up slope[a] (change / month) | 0.143 | (-0.109, 0.394) | 0.266 | 0.45 |
| | Residual | -- | -- | -- | 3.64 |

**Supplemental File 8 – Post Hoc Exploratory Analyses – Exercise Dose**

Interaction of exercise dose and duration of the intervention on all outcomes; **Model A**: Dose defined as total number of Moderate + Vigorous minutes of activity in the past week; **Model B**: Dose defined as total number of Vigorous minutes of activity in the past week.

Canadian Physical Activity Guidelines (CPAG): **Model A:** ≥150 minutes moderate to vigorous / week and **Model B**: ≥75 minutes vigorous activity.

| | **Model A - Estimated Intervention Effect over 6 month Intervention Phase with dosage of ≥150 min/week of moderate to vigorous activity:** 1.04 additional curl ups (95% CI: -1.03, 3.12)<br>Number of Observations: 420; Sample size: 43 (39 males; 4 females) | | | | |
|---|---|---|---|---|---|
| | **Parameter** | **Fixed Effects** | | | **Random Effects (SD)** |
| | | **Estimate** | **95% Confidence Interval** | **p-value** | |
| **Model A: Curl Ups (number completed)** | Intercept (50yr old male) | 15.873 | (12.953, 18.792) | 0.000 | 8.17 |
| | Age Effect | -0.354 | (-0.611, -0.096) | 0.008 | -- |
| | Sex Effect | -13.743 | (-23.261, -4.226) | 0.006 | -- |
| | Phase 1: Baseline slope (change / month) | 0.004 | (-0.274, 0.282) | 0.977 | 0.66 |
| | Phase 2: Intervention slope (change / month) | 0.001 | (0.000, 0.002) | 0.021 | 0.00 |
| | Phase 3: Follow-up slope[a] (change / month) | -0.038 | (-0.380, 0.304) | 0.827 | 0.24 |
| | Residual | -- | -- | -- | 5.30 |
| | **Model B - Estimated Intervention Effect over 6 month Intervention Phase with dosage of ≥75 min/week of vigorous activity:**<br>0.84 additional curl ups (95% CI: -1.18, 2.86)<br>Number of Observations: 420; Sample size: 43 (39 males; 4 females) | | | | |
| | **Parameter** | **Fixed Effects** | | | **Random Effects (SD)** |
| | | **Estimate** | **95% Confidence Interval** | **p-value** | |
| **Model B: Curl Ups (number copmleted)** | Intercept (50yr old male) | 16.045 | (13.195, 18.894) | 0.000 | 8.00 |
| | Age Effect | -0.338 | (-0.590, -0.086) | 0.010 | -- |
| | Sex Effect | -13.691 | (-23.014, -4.367) | 0.005 | -- |
| | Phase 1: Baseline slope (change / month) | 0.037 | (-0.234, 0.308) | 0.790 | 0.63 |
| | Phase 2: Intervention slope (change / month) | 0.002 | (0.000, 0.005) | 0.047 | 0.00 |
| | Phase 3: Follow-up slope[a] (change / month) | 0.102 | (-0.210, 0.414) | 0.521 | 0.41 |
| | Residual | -- | -- | -- | 5.40 |

**Supplemental File 8 – Post Hoc Exploratory Analyses – Exercise Dose**

Interaction of exercise dose and duration of the intervention on all outcomes; **Model A**: Dose defined as total number of Moderate + Vigorous minutes of activity in the past week; **Model B**: Dose defined as total number of Vigorous minutes of activity in the past week.

Canadian Physical Activity Guidelines (CPAG): **Model A:** ≥150 minutes moderate to vigorous / week and **Model B**: ≥75 minutes vigorous activity.

| | **Model A - Estimated Intervention Effect over 6 month Intervention Phase with dosage of ≥150 min/week of moderate to vigorous activity:**<br>0.11 cm (95% CI: -1.10, 1.31)<br>Number of Observations: 427; Sample size: 44 (40 males; 4 females) | | | | |
|---|---|---|---|---|---|
| **Model A: Flexibility – Sit and Reach Test (cm)** | **Parameter** | **Fixed Effects** | | | **Random Effects (SD)** |
| | | **Estimate** | **95% Confidence Interval** | **p-value** | |
| | Intercept (50yr old male) | 25.060 | (21.956, 28.164) | 0.000 | 9.89 |
| | Age Effect | -0.161 | (-0.445, 0.123) | 0.258 | -- |
| | Phase 1: Baseline slope (change / month) | 0.080 | (-0.091, 0.251) | 0.356 | 0.45 |
| | Phase 2: Intervention slope (change / month) | 0.001 | (0.000, 0.001) | 0.011 | 0.00 |
| | Phase 3: Follow-up slope[a] (change / month) | -0.110 | (-0.288, 0.068) | 0.225 | 0.00 |
| | Residual | -- | -- | -- | 2.96 |

| | **Model B - Estimated Intervention Effect over 6 month Intervention Phase with dosage of ≥75 min/week of vigorous activity:**<br>-0.11 cm (95% CI: -1.28, 1.06)<br>Number of Observations: 427; Sample size: 44 (40 males; 4 females) | | | | |
|---|---|---|---|---|---|
| **Model B: Flexibility – Sit and Reach Test (cm)** | **Parameter** | **Fixed Effects** | | | **Random Effects (SD)** |
| | | **Estimate** | **95% Confidence Interval** | **p-value** | |
| | Intercept (50yr old male) | 25.177 | (22.061, 28.293) | 0.000 | 9.94 |
| | Age Effect | -0.154 | (-0.440, 0.131) | 0.281 | -- |
| | Phase 1: Baseline slope (change / month) | 0.102 | (-0.073, 0.276) | 0.253 | 0.47 |
| | Phase 2: Intervention slope (change / month) | 0.001 | (0.000, 0.002) | 0.019 | 0.00 |
| | Phase 3: Follow-up slope[a] (change / month) | -0.016 | (-0.169, 0.137) | 0.836 | 0.16 |
| | Residual | -- | -- | -- | 3.01 |

**Supplemental File 8 – Post Hoc Exploratory Analyses – Exercise Dose**
Interaction of exercise dose and duration of the intervention on all outcomes; **Model A**: Dose defined as total number of Moderate + Vigorous minutes of activity in the past week; **Model B**: Dose defined as total number of Vigorous minutes of activity in the past week.
Canadian Physical Activity Guidelines (CPAG): **Model A:** ≥150 minutes moderate to vigorous / week and **Model B**: ≥75 minutes vigorous activity.

## Self-Reported Physical Activity

**Model A - Estimated Intervention Effect over 6 month Intervention Phase with dosage of ≥150 min/week of moderate to vigorous activity:** -0.04 points (95% CI: -0.17, 0.08)
Number of Observations: 487; Sample size: 45 (41 males; 4 females)

| | Parameter | Fixed Effects | | | Random Effects (SD) |
|---|---|---|---|---|---|
| | | Estimate | 95% Confidence Interval | p-value | |
| **Model A: RAPA Aerobic (points)** | Intercept (50yr old male) | 4.703 | (4.562, 4.844) | 0.000 | 0.37 |
| | Age Effect | 0.002 | (-0.010, 0.014) | 0.734 | -- |
| | Sex Effect | -0.203 | (-0.653, 0.246) | 0.367 | -- |
| | Phase 1: Baseline slope (change / month) | 0.015 | (-0.004, 0.034) | 0.113 | 0.04 |
| | Phase 2: Intervention slope (change / month) | 0.000 | (0.000, 0.000) | 0.020 | 0.00 |
| | Phase 3: Follow-up slope[a] (change / month) | -0.013 | (-0.037, 0.011) | 0.281 | 0.04 |
| | Residual | -- | -- | -- | 0.40 |

**Model B - Estimated Intervention Effect over 6 month Intervention Phase with dosage of ≥75 min/week of vigorous activity:** -0.08 points (95% CI: -0.20, 0.04)
Number of Observations: 487; Sample size: 45 (41 males; 4 females)

| | Parameter | Fixed Effects | | | Random Effects (SD) |
|---|---|---|---|---|---|
| | | Estimate | 95% Confidence Interval | p-value | |
| **Model B: RAPA Aerobic (points)** | Intercept (50yr old male) | 4.724 | (4.582, 4.866) | 0.000 | 0.38 |
| | Age Effect | 0.003 | (-0.010, 0.015) | 0.663 | -- |
| | Sex Effect | -0.215 | (-0.674, 0.243) | 0.349 | -- |
| | Phase 1: Baseline slope (change / month) | 0.018 | (0.000, 0.037) | 0.046 | 0.04 |
| | Phase 2: Intervention slope (change / month) | 0.000 | (0.000, 0.000) | 0.161 | 0.00 |
| | Phase 3: Follow-up slope[a] (change / month) | -0.002 | (-0.024, 0.019) | 0.837 | 0.04 |
| | Residual | -- | -- | -- | 0.40 |

**Supplemental File 8 – Post Hoc Exploratory Analyses – Exercise Dose**
Interaction of exercise dose and duration of the intervention on all outcomes; **Model A**: Dose defined as total number of Moderate + Vigorous minutes of activity in the past week; **Model B**: Dose defined as total number of Vigorous minutes of activity in the past week.
Canadian Physical Activity Guidelines (CPAG): **Model A:** ≥150 minutes moderate to vigorous / week and **Model B**: ≥75 minutes vigorous activity.

**LEGEND**: Slopes are change in outcome over one month. [a] Follow-up slope is the difference in slope between the follow-up and intervention phase; Phase 1: Baseline slope p value <0.05: statistically significant difference in Phase 1 (baseline) slope versus 0;
Phase 2: Intervention slope p value <0.05: statistically significant difference in Phase 2 (intervention) slope versus 0;
Phase 3: Follow-up slope p value <0.05: statistically significant change in Phase 3 (follow-up monitoring) slope versus Phase 2 (intervention) slope.
