# Supplementary material for: Examining the impact of a community-based exercise intervention on cardiorespiratory fitness, cardiovascular health, strength, flexibility and physical activity among adults living with HIV: A three-phased intervention study
Source: PLoS One. 2021 Sep 24;16(9):e0257639. doi: 10.1371/journal.pone.0257639 (PMC8462727; doi:10.1371/journal.pone.0257639)
Supplement: S2 File — (PDF) [file pone.0257639.s003.pdf]

PROTOCOL REFERENCE # 32910

May 1, 2016

Dr. Kelly O'Brien
DEPT OF PHYSICAL THERAPY
FACULTY OF MEDICINE

Dear Dr. O'Brien,

Re: Your research protocol entitled, "Translating exercise into the HIV community: Evaluating a community-based exercise intervention to improve the health of adults living with HIV"

**ETHICS APPROVAL**	**Original Approval Date: April 29, 2016**<br>**Expiry Date: April 28, 2017**<br>**Continuing Review Level: 3**

We are writing to advise you that the HIV Research Ethics Board (REB) has granted approval to the above-named research protocol, for a period of **one year**. Ongoing research under this protocol must be renewed prior to the expiry date.

Any changes to the approved protocol or consent materials must be reviewed and approved through the amendment process prior to its implementation. Any adverse or unanticipated events in the research should be reported to the Office of Research Ethics as soon as possible.

Please ensure that you submit an Annual Renewal Form or a Study Completion Report 15 to 30 days prior to the expiry date of your current ethics approval. Note that annual renewals for studies cannot be accepted more than 30 days prior to the date of expiry.

If your research is funded by a third party, please contact the assigned Research Funding Officer in Research Services to ensure that your funds are released.

Best wishes for the successful completion of your research.

Yours sincerely,

Raj Maharaj
REB Co-Chair

Darrell Tan, M.D., Ph.D.
REB Co-Chair

Research Oversight and Compliance Office - Human Research Ethics Program
McMurrich Building, 12 Queen's Park Crescent West, 2nd Floor, Toronto, ON M5S 1S8 Canada
Tel: +1 416 946-3273 ● Fax: +1 416 946-5763 ● ethics.review@utoronto.ca ● http://www.research.utoronto.ca/for-researchers-administrators/ethics/

Office Use Only<br>Protocol Number:

Office of the Vice-President, Research and Innovation

Office of Research Ethics

ETHICS REVIEW PROTOCOL SUBMISSION FORM – FACULTY

Original Study Protocol: Approved April 29, 2016

SECTION A – GENERAL INFORMATION

1. TITLE OF RESEARCH PROJECT

Translating Exercise into the HIV Community: Evaluating A Community-Based Exercise Intervention to Improve the Health of Adults Living with HIV

2. INVESTIGATOR INFORMATION

Principal Investigator:

Title (e.g., Dr., Ms., etc.): Dr.	Name: Kelly O'Brien
Department (or organization if not affiliated with U of T): Department of Physical Therapy	
Mailing address: 500 University Ave, Room 160, Toronto, ON, Canada, M5G 1V7	
Phone: 416-978-0565	Institutional e-mail: kelly.obrien@utoronto.ca

Co-Investigators:

Are co-investigators involved? Yes ☒ No ☐

Title: Dr.	Name: Patty Solomon
Department (or organization if not affiliated with U of T): McMaster University, School of Rehabilitation Science	
Mailing address: Institute for Applied Health Sciences, McMaster University, 1400 Main Street West, Room 403, Hamilton, ON, Canada, L8S 1C7	
Phone: 905-525-9140 ext. 27820	Institutional e-mail: solomon@mcmaster.ca

Title: Dr.	Name: Ahmed Bayoumi
Department (or organization if not affiliated with U of T): St. Michael's Hospital	
Mailing address: Keenan Research Centre, St. Michael's Hospital, 30 Bond Street, Toronto, ON, Canada, M5B 1W8	
Phone: 416-864-5728	Institutional e-mail: ahmed.bayoumi@utoronto.ca

Title: Dr.	Name: Ada Tang
Department (or organization if not affiliated with U of T): McMaster University	
Mailing address: 1400 Main Street West, Room 437, Hamilton, Ontario, L8S 1C7	
Phone: 905-525-9140 ext. 27818	Institutional e-mail: atang@mcmaster.ca

Title: Dr.	Name: Aileen Davis
Department (or organization if not affiliated with U of T): Division of Health Care and Outcomes, Krembil Research Institute, University Health Network (UHN)	
Mailing address: MP11-322, 399 Bathurst Street, Toronto, Ontario M5T 2S8	
Phone: 416-603-5543	Institutional e-mail: advais@uhnresearch.ca

Title: Dr.	Name: Soo Chan Carusone
Department (or organization if not affiliated with U of T): Casey House	
Mailing address: 119 Isabella Street, Toronto, ON M4Y 1P2	
Phone: 416-362-7600	Institutional e-mail: schancarusone@CaseyHouse.on.ca

Title: Dr.	Name: Sean Rourke
Department (or organization if not affiliated with U of T): Centre for Research on Inner City Health, St. Michael's Hospital	
Mailing address: 30 Bond Street, Toronto, Ontario, M5B 1W8	
Phone:	Institutional e-mail: rourkes@smh.ca

Principal Knowledge User:

Title: VP General Manager	Name: Mehdi Zobeiry
Department (or organization if not affiliated with U of T): Toronto YMCA	
Mailing address: 20 Grosvenor St, Toronto, ON, Canada, M4Y 2V5	
Phone: 416-975-9168 x41400	Institutional e-mail: mehdi.zobeiry@ymcagta.org

3. UNIVERSITY OF TORONTO RESEARCH ETHICS BOARD:

Health Sciences ☐ Social Sciences, Humanities and Education ☐ HIV/AIDS ☒

To determine which Research Ethics Board (REB) your protocol should be submitted, please consult: http://www.research.utoronto.ca/for-researchers-administrators/ethics/human/boards-committees/..

4. LOCATION(S) WHERE THE RESEARCH WILL BE CONDUCTED:

If the research is to be conducted at a site requiring administrative approval/consent (e.g., in a school), please include all administrative consent letters. It is the responsibility of the researcher to determine what other means of approval are required, and to obtain approval prior to starting the project.

University of Toronto ☒
Hospital ☒ specify site(s) St. Michael's Hospital (some data analysis will occur here)
School board or community agency ☒ specify site(s) Community-based organizations such as: Toronto PWA Foundation, Casey House, AIDS Committee of Toronto, and the Canadian Working Group on HIV and Rehabilitation (locations where recruitment may occur) and Central Toronto YMCA (location where recruitment may occur and location of the intervention).
Community within the GTA ☐ specify site(s)
International ☐ specify site(s)
Other ☐ specify site(s)

The University of Toronto has an agreement with the Toronto Academic Health Sciences Network (TAHSN) hospitals regarding ethics review of hospital-based research where the University plays a

peripheral role. Based on this agreement, certain hospital-based research may not require ethics review at the University of Toronto. If your research is based at a TAHSN hospital please consult the following document to determine whether or not your research requires review at the University of Toronto. http://www.research.utoronto.ca/for-researchers-administrators/ethics/human/at-a-glance/where-to-apply-tahsn-institutions/

5. OTHER RESEARCH ETHICS BOARD APPROVAL(S)

(a) Does the research involve another institution or site? Yes ☒ No ☐
(b) Has any other REB approved this project? Yes ☐ No ☒

If **Yes**, please provide a copy of the approval letter upon submission of this application.

If **No**, will any other REB be asked for approval?

Yes ☒ McMaster University, St. Michael's Hospital No ☐

Please note that REB approvals from other sites must be submitted to the ORE at U of T

6. FUNDING OF THIS PROJECT

(a)

Funding Status	Source and Type	Details
Funded ☒	Agency: CIHR Operating Grant	Fund #: 498753
	Agency:	Fund # :4 (6 digits)
Applied for funding ☐	Agency:	Submission date:
	Agency:	Submission date:
Unfunded ☐<br>If unfunded, please explain why no funding is needed:		

(b) If one protocol is to cover more than one grant, please include all fund numbers:

7. CONTRACTS

Is this research to be carried out as a contract? Yes ☐ No ☒

If yes, is there a University of Toronto funding or non-funded agreement associated with the research?

Yes ☐ No ☐

If **Yes,** please append a copy of the agreement with of this application.

Is there any aspect of the contract that could put any member of the research team in a potential conflict of interest? Yes ☐ No ☒

If yes, please elaborate under #10.

8. PROJECT START AND END DATES

Estimated start date for the component of this project that involves human participants or data: March 2016
Estimated completion date of involvement of human participants or data for this project: December 2018

9. SCHOLARLY REVIEW:

(Please note: for submissions to the **HIV REB** from community investigators, scientific review is a pre-requisite for ethics review. If your study is unfunded, please contact the OHTN to arrange a scientific review prior to completing your ethics submission.)

(a) Please check one:

I. ☒ The research has undergone scholarly review by thesis committee, departmental review committee, peer review committee or some other equivalent (Specify review type – e.g., departmental research committee, supervisor, CIHR, SSHRC, OHTN, etc.): CIHR Scholarly review (CIHR Review Committee). This work builds on a pilot study recently completed at the YMCA (pilot protocol was reviewed / approved by UofT REB (Protocol #31044), McMaster REB, and St. Michael's Hospital REB.

II. ☐ The research will undergo scholarly review prior to funding (Specify review committee – e.g., departmental research committee, SSHRC, CIHR peer-review committee, etc.):

III. ☐ The research will not undergo scholarly review (Please note that all research greater than minimal risk requires scholarly review)

(b) If box I or II above was checked, please specify if:

☐ The review was/will be specific to this protocol

☒ The review was/will be part of a larger grant – This study is the third phase of a larger intervention study with the aim to develop, pilot, and fully implement and evaluate the effect of a community-based exercise program for adults living with HIV in Toronto (Protocol # 31044). Previous pilot work that relates to this submission include:

- The first design phase of the study (identifying factors to consider in the development of an HIV-CBE program) was completed by a group of MScPT students under the supervision of Kelly O'Brien at the University of Toronto (REB Protocol Reference #: 29545).
- The second phase of this study (piloting the community-based exercise program at the Central Toronto YMCA) was recently completed and we are currently piloting an improved version of the exercise log (REB Protocol Reference # 31044).
- A second qualitative study with the aim to evaluate the experience of engaging in the CBE program from the perspective of adults living with HIV is currently being conducted by a group of MScPT students under the supervision of Kelly O'Brien (REB Protocol Reference #32275)

10. CONFLICTS OF INTEREST

(a) Will the researcher(s), members of the research team, and/or their partners or immediate family members:

(i) Receive any personal benefits (e.g., financial benefit such as remuneration, intellectual property rights, rights of employment, consultancies, board membership, share ownership, stock options, etc.) as a result of or in connection with this study? Yes ☐ No ☒

(ii) If **Yes**, please describe the benefits below. (Do not include conference and travel expense coverage, or other benefits which are considered standard for the conduct of research.)

The YMCA may indirectly benefit by broadening their membership base from participants who decide to join the YMCA in the baseline monitoring phase or sustain their membership after participation in the study. They may also benefit financially from room booking fees for the educational sessions, qualitative interviews and questionnaire assessments.

These financial benefits are offset with the in-kind contributions from the YMCA for this study which include: waiving the initial YMCA membership fee for participants ($96.05 per participant; total contribution for 120 participants ~$11,526), and providing a 25% discounted rate for the CardioCoach VO2 Max and Body Metric Body Composition assessments (estimated total of~$72,817 in-kind contribution by the YMCA for 120 participants at 12 assessment each).

Participants in this study will receive a membership with the YMCA. As per regular practice, the YMCA collects personal and emergency contact information from all of its members (including name, birthday, allergies or health concerns, gender, address, phone number, email address, emergency contact name and

phone number). This will be collected and stored at the Central Toronto YMCA as part of their general membership process, but is not part of the research study. The YMCA does not provide the information of their members to others. However they do communicate with members regarding YMCA promotions, or newsletters. All communication with YMCA members is within the new Canadian Anti-Spam Legislation (CASL).

(b) Describe any restrictions regarding access to or disclosure of information (during or at the end of the study) that have been placed on the investigator(s). These restrictions include controls placed by the sponsor, funding body, advisory or steering committee.

N/A

(c) Where relevant, please explain any pre-existing relationship between the researcher(s) and the researched (e.g., instructor-student; manager-employee; clinician-patient; minister-congregant). Please pay special attention to relationships in which there may be a power differential – actual or perceived..

Participants may include persons whom Kelly O'Brien, Ahmed Bayoumi, Patty Solomon, Aileen Davis, Ada Tang or Sean Rourke have worked with as a colleague in a community-based research or educational capacity. The Knowledge Users in the study are General Manager (Mehdi Zobeiry) and Manager of Programming (Katie Lowe) at the Central YMCA in Toronto who also may know participants through the community. Study investigators or the research coordinator, who have no relationship with the potential participant, will obtain consent. Interested participants will be invited to contact the study co-investigators or research coordinator who has no relationship with the potential participants, who will discuss the study in detail and if applicable, will obtain consent.

(d) Please describe the decision-making processes for collaborative research studies. If Terms of Reference exist, attach them. Collaborative research studies include those where a number of sites (e.g. other universities, non-TAHSN hospitals, etc.) are involved, as well as those that involve community agencies.

This research involves an academic-community partnership between the University of Toronto, McMaster University, St. Michael's Hospital, University Health Network, Central Toronto YMCA, Casey House, CWGHR and Toronto PWA Foundation. This research is also informed by an HIV Community Advisory Committee that Kelly O'Brien (principal investigator) works with part of a larger program of research (HIV Disability Questionnaire). Toronto PWA, the Canadian Working Group on HIV and Rehabilitation (CWGHR) and Casey House are represented on the Community Advisory Committee as collaborators in this work. The decision making process will be shared among the members of the research team who will inform and guide all aspects of this research.

SECTION B – SUMMARY OF THE PROPOSED RESEARCH

11. RATIONALE

Describe the purpose and scholarly rationale for the proposed project. State the hypotheses/research questions to be examined. The rationale for doing the study must be clear. Please include references in this section.

Research Aim and Objectives
The primary aim of this research is to evaluate a community-based exercise (CBE) intervention for adults living with HIV within the community with the goal of reducing disability and enhancing health (cardiopulmonary, strength, weight and body composition, and neurocognitive outcomes) and contextual factor outcomes (social support, stigma, mastery, coping) for adults living with HIV.

The secondary aim of this research is to utilize data collected during the course of the exercise intervention to further assess the measurement properties of the HIV Disability Questionnaire (HDQ); specifically the interpretability (cross-sectional meaning of HDQ scores and minimal clinically important difference); and responsiveness of the HDQ (ability to detect change in disability when it occurs) with adults living with HIV.

Specific objectives are to:
1) To determine the *extent* to which adults with HIV participate in a CBE intervention;
2) To assess the *effect* of a CBE intervention on health and disability outcomes [physical (cardiopulmonary fitness, strength, weight, body composition and anthropometrics, flexibility), mental-emotional, cognitive health, daily function, social inclusion] for adults living with HIV;
3) To assess *engagement* in CBE for adults with HIV over time (adherence, level of physical activity);
4) To evaluate the *process* (strengths and challenges; feasibility; accessibility, long term sustainability) of implementing a CBE intervention within the community from the perspective of recreation providers (fitness instructors; managers) and adults living with HIV.
5) To assess change (episodic nature) of disability as measured by the HDQ among adults living with HIV who participate in the exercise intervention to inform the assessment of interpretability (objective 5a) and responsiveness (objective 5b) of the HDQ.

Rationale
In developed countries, people living with HIV (PHAs) are living longer and now aging with the health-related consequences of HIV, potential adverse effects of treatment and multi-morbidity (1-7). These health-related consequences are known as *disability*, and include symptoms and impairments, difficulties with day-to-day activities, challenges to social inclusion, and uncertainty or anxiety about future health (8).

Rehabilitation, such as physical therapy and occupational therapy, can limit disability experienced by HIV and comorbidities such as fatigue, pain, cognitive impairments, body composition changes, and employment challenges, and has the potential to improve health and quality of life for PHAs. However, rehabilitation in the context of HIV is still emerging. Few rehabilitation professionals adequately serve PHAs (9). With little access to formal rehabilitation services through the health system, PHAs are likely left to employ self-management strategies to deal with their daily health challenges (10). **Exercise** is one intervention that can address disability and improve or sustain the health of PHAs (11). Systematic reviews suggest exercise is safe and may lead to benefits in cardiopulmonary fitness, strength, weight and body composition, and psychological status (12-15). However, this evidence focuses on highly supervised interventions by physical therapists and exercise physiologists that can be costly and unrealistic for PHAs to sustain as part of their everyday lifestyle. So despite the benefits, few PHAs engage in regular physical activity (12, 13, 16-18). It is essential to consider exercise interventions that are accessible and practical for PHAs to sustain over the long term.

Community Based Exercise (CBE) is an ideal approach for enhancing the health of PHAs within a self-management framework. CBE involves a group of individuals exercising under the supervision of a health or fitness instructor with the goal of promoting regular exercise in the community (19-21). CBE can foster social interaction, support and encouragement to exercise (19-21), and promote emotional, cognitive and behavioural self-management strategies to help independently manage health challenges associated with chronic conditions (20, 22). *However, the impact of CBE when translated into the 'real-world' HIV community setting, and its sustainability over the long term are unknown.*

In an earlier phase of work, we conducted a qualitative study to identify the key factors to consider in the development and implementation of a CBE program for people living with HIV. (REB Protocol Reference #: 29545). This work directly informed the development and refinement of the CBE intervention in this study. We also conducted a pilot study to test the community-based exercise intervention, recruitment strategy and data collection methods to inform the refinement of the protocol for this study (REB Protocol Reference # 31044). A second qualitative study with the aim to evaluate the experiences engaging in the pilot CBE

program from the perspective of adults living with HIV is currently being conducted by a group of MScPT students under the supervision of Kelly O'Brien (REB Protocol Reference #32275).

Hence, this protocol reflects the continuation of this research whereby the primary aim is to evaluate a community-based exercise intervention to reduce disability experienced by adults living with HIV.

In order to determine the effectiveness of rehabilitation interventions, we need to have established tools that comprehensively describe disability and can measure change in disability over time (23). The HIV Disability Questionnaire (HDQ) is a newly developed self-administered instrument with the purpose of describing the presence, severity and episodic nature of disability experienced by people living with HIV (24). Developed during Kelly O'Brien's postdoctoral research and derived from the Episodic Disability Framework, the HDQ measures disability across six domains: physical symptoms and impairments, mental-emotional health symptoms, cognitive symptoms, difficulties with day-to-day activities, challenges to social inclusion and uncertainty about future health (24). The HDQ possesses sensibility (face and content validity, ease of use) from the perspective of PHAs and HIV clinicians and we subsequently demonstrated HDQ validity and reliability with over 360 PHAs in Ontario and 96 PHAs in Ireland (25-26). Hence, the HDQ is an ideal tool in which to assess the effect of exercise interventions on disability for people with HIV. *However, in order to establish the HDQ as an evaluative tool; to document the episodic nature of disability and determine the effectiveness of different treatment interventions, we need to know what the HDQ scores mean (interpretability) and determine whether the HDQ can detect changes in disability when it occurs (responsiveness). Exercise is an ideal intervention in which to assess these measurement properties of the HDQ.*

Hence, the secondary aim of this research is to utilize data collected during the course of the exercise intervention to assess the interpretability (cross-sectional meaning of HDQ scores and minimal clinically important difference); and responsiveness of the HIV Disability Questionnaire (HDQ) (ability to detect change in disability when it occurs) with adults living with HIV.

Outcomes will yield a comprehensive community-based rehabilitation intervention for adults living with HIV, data on the impact of community-based exercise for PHAs and the interpretability and responsiveness of the HDQ, while building a research team of community and academic partners. Results will inform the uptake of this CBE intervention within the community.

Conceptual Foundation

We will use the *RE-AIM Framework* as the conceptual foundation to evaluate the CBE intervention. The *RE-AIM Framework* is derived from the field of *Implementation Science* that emphasizes the importance of considering multiple aspects of an intervention beyond clinical efficacy (27). The *Framework* includes criteria to evaluate the impact and translation of an intervention at both individual and organizational-levels in order to promote uptake, transferability and ultimately enhance the public health impact of health promotion interventions (28). Components of the *Framework* include: **Reach –**the number, proportion, and representativeness of individuals who participate in a given intervention; **Effectiveness** or efficacy – the impact of the intervention; **Adoption** – the number, proportion and representativeness of organizations and staff who are willing to initiate the intervention as a program; **Implementation** – the process (consistency, quality, time and cost) of the delivery of the intervention; and **Maintenance** – the extent individuals can sustain benefits of the intervention over time, and to which organizations adopt the intervention as a part of usual practice. In this study, we will assess the **Reach** *(objective 1- extent PHAs participate in CBE)* and **Maintenance** *(objective 3- engagement in CBE over time)* using quantitative methods. **Effectiveness** (*objective 2)* will be assessed both quantitatively (assessing change in health and disability outcomes); and qualitatively (exploring perceived benefits of the intervention) from the PHA perspective. **Adoption** and **Implementation,** which concern organizational aspects of the evaluation, will be assessed using qualitative interviews with recreation provider and PHA participants to determine the strengths and challenges of implementing the CBE intervention; and the feasibility, accessibility and long-term sustainability of CBE in the community *(objective 4)*.

References

1. Rusch M, Nixon S, Schilder A, Braitstein P, Chan K, Hogg RS. Impairments, activity limitations and participation restrictions: prevalence and associations among persons living with HIV/AIDS in British Columbia. *Health Qual Life Outcomes.* 2004;2:46.
2. Rusch M, Nixon S, Schilder A, Braitstein P, Chan K, Hogg RS. Prevalence of activity limitation among persons living with HIV/AIDS in British Columbia. *Can J Public Health.* Nov-Dec 2004;95(6):437-440.
3. Gaidhane AM, Zahiruddin QS, Waghmare L, Zodpey S, Goyal RC, Johrapurkar SR. Assessing self-care component of activities and participation domain of the international classification of functioning, disability and health (ICF) among people living with HIV/AIDS. *AIDS Care.* Oct 2008;20(9):1098-1104.
4. Palella FJ, Jr., Baker RK, Moorman AC, et al. Mortality in the highly active antiretroviral therapy era: changing causes of death and disease in the HIV outpatient study. *J Acquir Immune Defic Syndr.* Sep 2006;43(1):27-34.
5. Willard S, Holzemer WL, Wantland DJ, et al. Does "asymptomatic" mean without symptoms for those living with HIV infection? *AIDS Care.* Mar 2009;21(3):322-328.
6. Guaraldi G, Orlando G, Zona S, et al. Premature age-related comorbidities among HIV-infected persons compared with the general population. *Clin Infect Dis.* Dec 2011;53(11):1120-1126.
7. Rodriguez-Penney AT, Iudicello JE, Riggs PK, et al. Co-morbidities in persons infected with HIV: increased burden with older age and negative effects on health-related quality of life. *AIDS Patient Care STDS.* Jan 2013;27(1):5-16.
8. O'Brien KK, Bayoumi AM, Strike C, Young NL, Davis AM. Exploring disability from the perspective of adults living with HIV/AIDS: development of a conceptual framework. *Health Qual Life Outcomes.* 2008;6:76.
9. Worthington C, Myers T, O'Brien K, Nixon S, Cockerill R, Bereket T. Rehabilitation professionals and human immunodeficiency virus care: results of a national Canadian survey. *Arch Phys Med Rehabil.* Jan 2008;89(1):105-113.
10. Swendeman D, Ingram BL, Rotheram-Borus MJ. Common elements in self-management of HIV and other chronic illnesses: an integrative framework. *AIDS Care.* Oct 2009;21(10):1321-1334.
11. Botros D, Somarriba G, Neri D, Miller TL. Interventions to Address Chronic Disease and HIV: Strategies to Promote Exercise and Nutrition Among HIV-Infected Individuals. *Curr HIV/AIDS Rep.* Dec 2012;9(4):351-363.
12. O'Brien K, Nixon S, Tynan AM, Glazier R. Aerobic exercise interventions for adults living with HIV/AIDS. *Cochrane Database Syst Rev.* 2010;8:CD001796.
13. O'Brien K, Tynan AM, Nixon S, Glazier RH. Effects of progressive resistive exercise in adults living with HIV/AIDS: systematic review and meta-analysis of randomized trials. *AIDS Care.* Jul 2008;20(6):631-653.
14. Gomes-Neto M, Conceicao CS, Oliveira Carvalho V, Brites C. A systematic review of the effects of different types of therapeutic exercise on physiologic and functional measurements in patients with HIV/AIDS. *Clinics (Sao Paulo).* 2013;68(8):1157-1167.
15. Gomes Neto M, Ogalha C, Andrade AM, Brites C. A systematic review of effects of concurrent strength and endurance training on the health-related quality of life and cardiopulmonary status in patients with HIV/AIDS. *BioMed research international.* 2013;2013:319524.
16. Clingerman E. Physical activity, social support, and health-related quality of life among persons with HIV disease. *Journal of community health nursing.* Fall 2004;21(3):179-197.
17. Clingerman EM. Participation in physical activity by persons living with HIV disease. *J Assoc Nurses AIDS Care.* Sep-Oct 2003;14(5):59-70.
18. Schuelter-Trevisol F, Wolff FH, Alencastro PR, et al. Physical activity: do patients infected with HIV practice? How much? A systematic review. *Curr HIV Res.* Sep 2012;10(6):487-497.
19. Winward C. Supporting community-based exercise in long-term neurological conditions: experience from the Long-term Individual Fitness Enablement (LIFE) project. *Clin Rehabil.* Jul 2011;25(7):579-587.
20. Salbach NM, Howe JA, Brunton K, Salisbury K, Bodiam L. Partnering to Increase Access to Community Exercise Programs for People With Stroke, Acquired Brain Injury and Multiple Sclerosis. *Journal of physical activity & health.* May 13 2013.

21. Stuart M, Benvenuti F, Macko R, et al. Community-based adaptive physical activity program for chronic stroke: feasibility, safety, and efficacy of the Empoli model. *Neurorehabilitation and neural repair.* Sep 2009;23(7):726-734.
22. Lee LL, Arthur A, Avis M. Using self-efficacy theory to develop interventions that help older people overcome psychological barriers to physical activity: a discussion paper. *Int J Nurs Stud.* Nov 2008;45(11):1690-1699.
23. O'Brien KK, Bayoumi AM, Strike C, Young NL, King K, Davis AM. How do existing HIV-specific instruments measure up? Evaluating the ability of instruments to describe disability experienced by adults living with HIV. Health Qual Life Outcomes. 2010;8:88. Epub 2010/08/21.
24. O'Brien KK, Alexander, R., King, K., Murray, J., Tebeje, M., Bayoumi, A.M., Bereket, T., Swinton, M., Norman, G., Solomon, P. Developing a new HIV disability questionnaire: a community-integrated approach. . Physiotherapy Canada. 2010;62(Supplement 1):34-5
25. O'Brien KK, Bayoumi AM, Stratford P, Solomon P. Establishing the Domain Structure of the HIV Disability Questionnaire: An Exploratory Factor Analysis. Submitted to: Quality of Life Research (September 2012)
26. O'Brien KK, Solomon P, Bergin C, O'Dea S, Stratford P, Iku N, Bayoumi AM**.** Reliability and Validity of the HIV Disability Questionnaire (HDQ) with Adults Living with HIV in Canada and Ireland. P222. Annual Canadian Conference on HIV/AIDS (CAHR). Canadian Journal of Infectious Diseases and Medical Microbiology. Spring 2014. 25(Suppl A);pp 107A
27. Glasgow RE, Eckstein ET, Elzarrad MK. Implementation science perspectives and opportunities for HIV/AIDS research: integrating science, practice, and policy. *J Acquir Immune Defic Syndr.* Jun 1 2013;63 Suppl 1:S26-31.
28. Glasgow RE, Vogt TM, Boles SM. Evaluating the public health impact of health promotion interventions: the RE-AIM framework. *Am J Public Health.* Sep 1999;89(9):1322-1327.

12. METHODS

(a) Please describe all formal and informal procedures to be used. Describe the data to be collected, where and how they will be obtained and how they will be analyzed.

STUDY DESIGN

We will conduct a prospective longitudinal study using quantitative and qualitative methods to evaluate a CBE exercise intervention at the Central Toronto YMCA with adults living with HIV in the community while using anchor and distribution-based methods to assess the measurement properties of the HDQ. *Interpretability* is the degree to which an individual can assign meaning to a score (or a change in score) on an instrument (29,30). *Responsiveness* is the ability of an instrument to assess and quantify clinically important changes within or between individuals over time (31). Responsiveness may be considered an aspect of validity – the ability of an instrument to detect change over time in the construct to be measured' (30). In situations in which no "gold standard" exists, such as disability, longitudinal construct validity may be used to assess responsiveness by testing a priori hypotheses about the predicted relationship between change scores of measures that relate to components of disability and change scores of the HDQ. The strength of longitudinal validity depends on the extent to which the hypotheses being tested are verified or refuted (32).

METHODS:

We will use an interrupted time series (ITS) design in combination with qualitative interviews to assess outcomes at baseline (pre-testing phase), during (intervention phase) and after the CBE intervention (post-testing phase) to evaluate the short- and long-term effect of CBE.

For objectives #1-4 we will implement a CBE intervention with adults living with HIV and assess health status and administer disability and health status measures bimonthly to evaluate the impact of the intervention on health and contextual factor outcomes for PHAs. We will also conduct qualitative interviews with a subset of participants and recreation providers prior to, during and after the intervention. For objective #5, at 0, 4, 8, 10, 12, 14, 18 and 22 months we will administer the HDQ in conjunction with two Global Rating Change (GRC)

scales and a Disability Rating Scale to assess interpretability (objective 5a) and in conjunction with the MOS-HIV and EQ5D questionnaires in order to test a series of a priori hypotheses to assess responsiveness of the HDQ (objective 5b).

Community collaborations fostered throughout earlier phases of this research will continue in this study. People living with HIV (PHAs), representatives from ASOs (Toronto PWA Foundation), a specialty hospital (Casey House), the Canadian Working Group on HIV and Rehabilitation (CWGHR), Ontario Ministry of Health and Long-Term Care, and community-health centres (YMCA) will comprise the Community Advisory Committee who have guided and will continue to advise on all aspects of the research.

CBE Intervention

The *HIV Community-Based Exercise (CBE) Study* is a 22-month study consisting of an 8-month baseline phase, 6-month exercise intervention at the Central Toronto YMCA and an 8-month post-intervention phase. The intervention will be conducted in waves of ~30 participants. The intervention is derived from high level research evidence on the effectiveness of exercise with PHAs, recommendations from the American College of Sports Medicine (ACSM), the preceding qualitative study exploring factors to consider in a CBE intervention for people with HIV, the preceding pilot study assessing the intervention, recruitment strategy and data collection outcomes, the current qualitative study evaluating the experience of engaging in the CBE program from the perspective of adults living with HIV and the existing HIV community based exercise programs in the UK.

Baseline (0-8 months): Participants will be monitored bimonthly during the baseline (pre-intervention phase) for 32 weeks. This will identify the pattern of pretest outcomes and will serve as the 'control' phase in which to compare the level and trend of outcomes during and post-intervention in the ITS.

Intervention - Exercise and Self-Management Education (8-14 months): The *HIV Community-Based Exercise (CBE) intervention* is a 6-month exercise program at the Central Toronto YMCA. The intervention is derived from high quality research evidence on the effectiveness of exercise with PHAs, recommendations from the American College of Sports Medicine (ACSM), qualitative consultation with community (PHAs and fitness instructors) (33) existing HIV CBE programs in the UK, and collectively adapted for the Canadian context. The intervention will include: *framing and explanation* of the exercise through educational sessions; participants will have the opportunity to *choose* activities of interest to enhance engagement and adherence to exercise; *problem-solving support* where participants will have access to staff to assist with goal setting and overcoming barriers to exercise; and *communication* among participants, fitness instructors and study staff to ensure participants receive *feedback* on their progression and goal attainment (36).

Prior to the intervention, fitness instructors will take part in the CWGHR inter-professional online learning module on HIV and an education session on HIV, rehabilitation and exercise for PHAs (37). Participants will individually meet the fitness instructor to assess their goals and establish an individualized exercise program that will include a combination of aerobic, resistive, neuromotor and flexibility training.

Aerobic exercise will include physical activity 3 days/week (frequency) at 60-70% heart rate maximum (intensity) for at least 30 minutes (time) with a variety of types of activity based on participant choice (type). *Resistance exercise* will include resistance training for each major muscle group ~8-10 exercises (type) 3 days/week (frequency) at 60-70% 1 repetition maximum (intensity) with 10-12 repetitions each (time). *Flexibility exercise* will include stretching major muscle-tendon groups in a static stretch for 10-30 seconds each with 2 repetitions; *Neuromotor exercise* will include a range of motor skills (e.g. balance, agility, coordination and gait) for approximately 20-30 minutes (34). To achieve these exercise elements, participants will engage in a combination of individual based exercise (e.g. weight circuit training, endurance treadmill walking / jogging, stationary bike, swimming) and group-based exercise in the form of a class (e.g. circuit training, spinning, yoga, aerobics). The combination of group and individualized exercise will ensure the intervention will be tailored to the individual while promoting benefits of efficacy and social support that come

from group activity. Note, in this study we adopt a 'guided adaptation' approach whereby the intervention may be adapted based on the ongoing assessment of the needs and ability of participants by the fitness instructor.

Participants will attend *exercise sessions* for ~1.5 hour, 3 times per week for 24 weeks. Sessions will be supervised weekly by a fitness instructor who will monitor progress of participants and progress exercise intensity. We will ask Fitness Instructors to fill out the Coach Log (Appendix B) following each weekly exercise session. Participants will also attend monthly *educational sessions* co-led by a health or fitness professional and/or person living with HIV focused on topics related to self-management and healthy lifestyle living with HIV. Topics of the 6 sessions may include: stress and relaxation, pain management, nutrition, fatigue management, continuing physical activity, smoking cessation, goal setting, confidence and self-management, community services and support, living with HIV, rehabilitation in the context of HIV, sexuality and body image, and wrap up and overall Q&A.

Participants will be asked to complete an online exercise log (Appendix C) at the end of each week to document the frequency, intensity, time and type of activity during the intervention. We will provide participants with a paper-based exercise log (Appendix D) that they can use to document their daily activity to assist with filling out the weekly online log. We will administer the online exercise log using Fluid Surveys Software.

Post-Intervention - Self-Monitored Exercise (14-22 months): Participants will be encouraged to continue to engage in unsupervised exercise 3 times per week. As per usual practice at the YMCA, a fitness instructor will be available to monitor participants monthly. Participants will be asked to complete the weekly online exercise log throughout to document the frequency, intensity, time and type of activity during both exercise phases (8-22 months) to assess long-term engagement in exercise. Participants also will be provided with a wireless activity tracker (Fitbit®) to self-monitor steps, distance and calories burned during both exercise phases (8-22 months) (38). Participants will be asked to download their Fitbit activity data (steps, distance, and calories) each week throughout (8-22 months) to assist with filling in their exercise logs. Investigators will directly export participant Fitbit data monthly to assist in capturing physical activity of participants in the intervention and post-intervention phases.

Research Procedure

Participants will be followed over the course of the intervention and assessments administered at twelve time points: baseline (0, 2, 4, 6 months), intervention (8, 10, 12, 14 months) and post-intervention (16, 18, 20, 22 months). Please see Appendix E for an overview of the research procedure, Appendix F for a timeline of the intervention and Appendix G for a timeline of the entire study. Participants will attend 2 outcome assessment sessions, one at the YMCA (objective health assessment) and a second session with the research coordinator for the self-reported disability and contextual factor assessment (at the YMCA or Toronto PWA Foundation). Assessments measuring interpretability (Global Rating Change Scales and Disability Rating Scale) will be administered during the baseline phase (0, 4, months), intervention phase (8, 10, 12 months) and post-intervention phase (14, 18, 22 months). Neurocognitive assessments will be administered at 8 months, 14 months and 22 months. All other assessments will be administered bimonthly.

We will evaluate the CBE intervention with PHAs in the community using the *RE-AIM Framework.*

Objective #1 - Reach

To determine the extent PHAs participate in the CBE intervention: We will measure the number of individuals who a) are eligible but do not consent, b) consent but do not initiate the CBE intervention, c) initiate but do not complete in the CBE intervention, d) complete the CBE intervention but not self-monitored exercise and e) complete the CBE intervention and self-monitored exercise. We will specifically determine the number of participants who withdraw, the week they withdraw, and reasons for withdrawal.

Objective 2 (Effectiveness)
To assess the effect of the CBE: We will use a combination of an interrupted time series (ITS) analysis and qualitative interviews to assess the impact of the CBE intervention. Outcomes of interest are guided by the disability and contextual factor components in the *Episodic Disability Framework* (Appendix H). Interrupted time series analyses assess the effect of an intervention by comparing a pattern of pretest responses (baseline), with a pattern of post-test responses (post-intervention). An effect is demonstrated when there is a change in the level and/or slope (or trend) of the interruption or post-treatment responses as compared with the pretest responses. We will assess disability and health outcomes bimonthly at baseline (0-8 months), during (8-14 months) and post-intervention (14-22 months) for a total of 12 time points (Appendix F). In the demographic questionnaire, we will ask participants whether they experienced any life events or illnesses since the last assessment that may impact their ability to engage in exercise. We will also conduct a series of face-to-face interviews with a sub-sample of 15 PHA participants at initiation (8 months), midway (11 months) and completion (14 months) of the 6-month CBE intervention.

Part 1 – YMCA Objective Health Assessment

Fitness staff at the YMCA will conduct the following objective assessments bimonthly (at 0, 2, 4, 6, 8, 10, 12, 14, 16, 18, 20 and 22 months).

Physical Health Symptoms and Impairments: The *Central Toronto YMCA Performance Centre* provides comprehensive physical fitness assessment services that include: a) *cardiopulmonary fitness* (maximum oxygen consumption (VO2max), resting and maximal heart rate, resting and maximal blood pressure); b) *strength* for upper and lower body (grip strength, vertical jump, back extension, push ups, partial curl ups); c) *weight, body composition and anthropometrics* (weight, body mass index, body fat percent, lean body mass, waist and hip circumference, fat free mass); and d) *flexibility* (lower back and hamstrings). Fitness staff at the YMCA with certified fitness training experience will conduct the objective assessment. See Appendix I for an overview of the procedure for assessing these outcomes.

If a participant does not wish to or is unable to do the VO2 max test, Coaches will conduct either the Treadmill Walking Test (Appendix II) or the One Mile Walk Test (Appendix JJ) as an alternative.

The above assessment will take approximately 1.5 hours.

Part 2A - Self-Reported Questionnaire Assessment

The Research Coordinator will meet with participants at the YMCA, Toronto PWA Foundation or University of Toronto (depending on the preference of the participant) to administer the below questionnaires. Please see Appendix J for an overview of the Disability, Quality of Life, Health, Intrinsic Contextual Factors and Extrinsic Contextual Factors questionnaires.

Disability:
1) The **HIV Disability Questionnaire (HDQ)** (Appendix J): We will administer the HDQ, a 69 item self-administered instrument we developed to describe the presence, severity and episodic nature of disability experienced by PHAs across six domains: physical, cognitive and mental-emotional health symptoms and impairments, difficulties with day-to-day activities, challenges to social inclusion and uncertainty (39-40).

2) Given we are assessing the properties of the HDQ, we will administer a generic questionnaire, the **EQ5D** (42) (Appendix J) to evaluate the impact of the CBE intervention. The EQ5D is a health status questionnaire comprised of five domains (mobility, self-care, usual activities, pain, anxiety / depression). The EQ5D also is widely used and has demonstrated responsiveness with PHAs (44-45). This measure will also be used to assess the responsiveness of the HDQ.

Quality of Life:
3) **Medical Outcomes Study HIV** (**MOS-HIV) Questionnaire** (Appendix J): We will administer the MOS-HIV to measure quality of life. The MOS-HIV consists of 35 questions that assess the ten dimensions of health: general health perceptions, pain, physical functioning, role functioning, social functioning, mental health, energy/fatigue, cognitive function, health distress and quality of life. This questionnaire has been used extensively and possesses reliability and validity with PHAs (44).

Mental Health:
4) The **Patient health Questionnaire (PHQ-9)** (Appendix J) is a 9 item measure of depression severity. Items are rated using a Likert-type scale from 0-3, with a total score range of 0-27 (46). Higher scores indicate a greater severity of depression. The PHQ-9 has been assessed for construct validity and test-retest reliability among PHAs (47-48).

Cognitive Health:
5) The **Medical Outcomes Study (MOS) Cognitive Functioning Scale** (Appendix J) includes 4 items with a 6-point ordinal scale that measures challenges across reasoning and problem solving, memory, attention, and concentration and thinking (49). The MOS Cognitive Function Scale scores range from 0-100 with higher scores representing better cognitive function. The scale has been validated for use with PHAs (50).

Intrinsic Contextual Factors:
Living strategies will be measured using the following two instruments, both of which possess reliability and validity and have been used with PHAs (53).

7) The **Brief COPE** (Appendix J) is a 28-item instrument, comprised of 14 scales with two items each (54). The scales are as follows: Active Coping, Planning, Positive Reframing, Acceptance, Humour, Religion, Using Emotional Support, Using Instrumental Support, Self-Distraction, Denial, Venting, Substance Use, Behavioural Disengagement, Self-Blame, The Brief COPE possesses internal reliability, internal validity and good construct validity for undergraduate students (55).

8) The **Pearlin Mastery Scale** (Appendix J) is a self-administered questionnaire which consists of 7 items that assess sense of personal control over important life forces or outcomes (56). The items have been developed to reflect whether individuals feel they have control over the decisions they make, what happens to them, and whether they can deal with their problems appropriately and effectively. Each item in the scale is measured using a Likert scale with 4 response categories, and summary scores are generated indicating level of mastery (limited, moderate, great) (57). This scale has demonstrated construct validity and good reliability for caregivers of PHAs, individuals with Alzheimer’s disease and individuals with Dementia (58-60).

Extrinsic Contextual Factors:

Stigma and social support are extrinsic factors that may influence the health and disability of PHAs (61). Their inclusion is integral to obtain a comprehensive understanding of how CBE may affect perceived stigma and social support and subsequently the health of PHAs.

9) We will administer the **HIV Stigma Scale** (Appendix J) which possesses construct validity and reliability when used with PHAs (62-63). This scale captures the complexity and multi-dimensionality of HIV-related stigma. The HIV Stigma Scale is a self-administered questionnaire comprised of 40 questions (62). The four subscales include: personalized stigma, disclosure, negative self-image and concern with public attitudes (62). This scale possesses good validity and reliability (64-65). Each item is measured using a Likert-type scale with 5 response categories ranging from “strongly disagree” to “strongly agree.”

10) We will administer the **Social Support Scale (MOS-SSS)** (Appendix J) which possesses construct validity and reliability when used with PHAs (51). This scale is a self-administered questionnaire that was designed to measure five different dimensions of social support among patients with chronic illness. (63).

These dimensions are: emotional/informational support, tangible support, positive social interaction and affectionate support. The scale consists of 20 items that are measured using a scale with 5 response categories ranging “none of the time” to “all of the time.” Higher overall scores are indicative of higher levels of social support.

Personal Attributes (Demographic and Disease Characteristics):

11) The **demographic and disease questionnaire** (Appendix K1) will capture characteristics including but not limited to age, gender, length of time since HIV diagnosis, ethnocultural background, antiretroviral use and adherence, smoking history, and number and type of comorbidities. We will re-administer items from this questionnaire bimonthly (comorbidities, medication adherence, smoking history) and also ask whether there are any events that may influence participants’ ability to exercise since the last assessment (Appendix K2). Consideration of these attributes is integral to better understanding how they may influence the impact of, and engagement in, exercise.

The above self-reported questionnaire and cognitive screening assessment will take approximately 1.5 hours to complete.

Part 2B – Neurocognitive Assessment

Please see Appendix L for the administration instructions. The above will be administered by the Research Coordinator who has been trained in the below assessments by Dr. Rourke’s staff at three time points: initiation of the intervention (8 months), immediately post intervention (14 months), and at follow-up (22 months).

13) We will measure spatial working memory by using the **WMS-III Spatial Span Test** (66) which is a measure of working memory for visual-spatial information. In this test, the participant is confronted with a white board on which ten blue blocks are affixed. The examiner taps the blocks in a pre-arranged sequence, and the participant is asked to repeat the sequence by tapping the same blocks in the same order (spatial span forward) or the reverse order (spatial span backwards). The score is the sum of correctly repeated sequences (both forward and backward).

14) The **WAIS-R Digit Symbol Test** (67) is a measure of complex psychomotor speed. The participant is presented with a key pairing numbers from one to nine with nine different symbols. The participant’s task is to write the corresponding symbols below the numbers as quickly as possible. The score is the number of symbols correctly written within the 90 second time limit.

15) **The Hopkins Verbal Learning** Test (HVLT) (68) assesses verbal learning and working memory (immediate recall, delayed recall, and delayed recognition). In this test, the examiner reads a list of 12 words, which consist of three sets of four semantically categorized groups. Each time the examiner reads the list, the participant is asked recall as many of the words as possible, and the examiner records the responses. This reading and recall of the same word list is repeated for a total of three trials. Twenty to 25 minutes after the third learning trial, the examiner asks the participant to recall the word list again, recording the responses. The free recall is followed by a 24-word recognition list containing all twelve target words, plus six semantically related foils and six unrelated ones (69). Then, the total score for the three learning trials; delayed recall total; recognition; and discriminability are calculated. These scores are then converted to age-based T scores based on a general English-speaking population (n=1 179, age=16-92).

16) **The Grooved Pegboard** (70) assesses fine motor dexterity. The pegboard consists of a small board containing a 5x5 grid of slotted holes, angled in different directions, and a set of identical pegs. Each peg has a ridge along one side, requiring it to be rotated into position for correct insertion. The task, filling all 25 holes, is timed by the examiner. Both hands are usually tested, beginning with the dominant hand. The test’s

complexity makes it a sensitive instrument for measuring speed of processing, and for detecting slowing due to medication, disease progression (including HIV-disease progression) and brain injury (69). The score is the time to completion.

17) We will measure speed of processing and executive function by using **The Trail Making Test**. This is a test of visual conceptual and visuomotor tracking (69). Participants draw lines connecting consecutively numbered and/or lettered circles as quickly as possible. In Trails A participants are instructed to draw lines connecting numbered circles, in order, as quickly as possible, without lifting the pen from the paper. In Trails B, participants are required to draw lines connecting numbered and lettered circles, alternating between the two, in order, without lifting the pen from the paper. The examiner observes each exercise and points out any errors, instructing the participant to return to the last correct circle before continuing. The score for each test is time to completion. T-scores are calculated based on time in seconds.

18) **The Letter-Number Sequencing Test** is a measure of attention and complex working memory. The participant is presented verbally with sets of “scrambled” letters and number, and is tasked with repeating the sequence back by listing first the numbers in ascending order, then the letters in alphabetical order (66). Each sequence is scored as correct (1) or incorrect (0). The total score is the total number of correct sequences obtained.

19) We will administer **The Patient’s Assessment of Own Functioning** (PAOF) (71) to assess subjective cognitive symptoms. The PAOF is a multidimensional measure that assesses perceptions of memory, language/communication, orientation, use of hands, sensorimotor ability, and higher cognitive abilities/executive function. Participants typically read the 34-item questionnaire, and circle multiple-choice-style responses on their own, after receiving detailed instructions from the examiner, but the survey can be administered orally, as a structured interview. Each test item has five possible responses ranging from almost never to almost always, and responses correspond to a score of 0-5. Higher PAOF scores indicate poorer perceived cognitive function.

The above neurocognitive assessments will take approximately 30 minutes to complete.

Breaks and refreshments (water / juice) will be provided to participants as needed throughout the above assessments. We will document any reflections from the participants and investigative team about feasibility of the outcome assessment throughout the study in a Research Team CBE Study Log.

Part 3 – Face-to-Face Interviews

We will conduct a series of face-to-face interviews with a sub-sample of 15 PHA participants at initiation (8 months), midway (11 months) and completion (14 months) of the 6-month CBE intervention. Using a semi-structured interview guide (Appendix M), we will explore their a) current experience with exercise, b) anticipated benefits of exercise (*initiation*), and c) perceived impact of CBE on their health and disability over time (*midway and post*). In addition, we will specifically explore the influence of extrinsic factors (social support, stigma) and intrinsic factors (personal attributes, coping strategies), in the *Episodic Disability Framework,* on the impact and level of engagement in exercise. All interviews will be audio recorded and later transcribed verbatim.

Intake and Phase Completion/Withdrawal Questionnaires

20) At month 0 we will administer a **CBE Study Intake Questionnaire** which will ask participants for their contact information, how they found out about the study and previous exercise experience (new exerciser vs. experienced exerciser) (Appendix N).

At completion of Phase One and the beginning of Phase Two (month 8), we will administer the **Goal Attainment Scale** (Appendix O) (72), the **Phase One Completion/Withdrawal Questionnaire** (Appendix P1) and we will re-administer the **CBE Study Intake Questionnaire** (Appendix N)**.**

At completion of Phase Two and the beginning of Phase 3 (month 14), we will administer the **Goal Attainment Scale** (Appendix O), the **Phase Two Completion/Withdrawal Questionnaire** (Appendix P2) and we will re-administer the **CBE Study Intake Questionnaire** (Appendix N)**.**

At completion of Phase Three/end of the 22 month study (month 22), we will administer the **Phase Three Completion/Withdrawal Questionnaire** (Appendix P3).

If a participant decides to withdraw from the study prior to 8 months, we will ask him/her to complete the Phase One Completion/Withdrawal Questionnaire (Appendix P1) in order to capture the reasons for withdrawal, followed by the Version 2 Demographic Questionnaire (Appendix K2) upon exit from the study.

If a participant decides to withdraw from the study between 8 and 14 months, we will ask him/her to complete the Phase Two Completion/Withdrawal Questionnaire (Appendix P2) in order to capture the reasons for withdrawal, followed by the GAS (Appendix O) and the Version 2 Demographic Questionnaire (Appendix K2) upon exit from the study.

If a participant decides to withdraw from the study between 14 and 22 months, we will ask him/her to complete the Phase Three Completion/Withdrawal Questionnaire (Appendix P3) in order to capture the reasons for withdrawal, followed by the Version 2 Demographic Questionnaire (Appendix K2) upon exit from the study.

Objective 3 (Maintenance)
To assess engagement in CBE over time: Maintenance will be measured by assessing *physical activity* and *adherence* to the CBE intervention over 14 months. ***Physical Activity:*** We will measure physical activity by administering the *Rapid Assessment of Physical Activity (RAPA) Questionnaire* at each of the 12 time points (Appendix Q) and objectively using the Fitbit, a wireless activity monitor that will be used to track participant steps, distance and calories burned (73). The RAPA is a 9-item questionnaire that examines the numbers of frequency and intensity a participant spends in vigorous or moderate activity, including strength and flexibility within the last week. ***Adherence:*** We will document adherence by asking participants to complete a weekly online exercise log to track frequency, intensity, time and type of activity they engaged in (Appendix C). Participants will be asked to complete the weekly online log throughout the intervention (8-14 months) and post-intervention (14-22 months) to assess long-term engagement in exercise. We will administer the online exercise log using Fluid Surveys Software. At the end of each week, we will e-mail participants (Appendix R) with a link to the Weekly Online Exercise Log. The exercise log will take approximately 15 minutes to complete. Two days after we e-mail the weekly log to participants, we will send a 'Thank You/Reminder" (Appendix S) email thanking those participants who filled in the online log and reminding participants who have not yet completed the log to complete it. Responses will be directly stored on a secure server at the University of Toronto. Exercise log data will be downloaded from FluidSurveys to Microsoft excel for analysis. We will provide participants with a paper-based exercise log (Appendix D) that they can use to assist with filling out the weekly online log.

Objective 4 (Adoption and Implementation)
To evaluate the process of implementing the CBE intervention: We will conduct face-to-face interviews with a sample of 5 recreation providers (managers, fitness instructors) and 15 PHA participants at initiation, midway, and completion of the 6-month CBE intervention. We will explore anticipated concerns prior to the intervention (*initiation,* 8 months), insights on the strengths and challenges during the intervention (*midway,* 11 months), and reflections immediately post intervention (*post,* 14 months) on the overall process of translating the intervention within the community. Using a semi-structured interview guide (Appendix M), we will specifically explore the a) strengths and challenges of implementing the CBE intervention, b) accessibility

and feasibility of the intervention, and c) potential for long-term sustainability of the program. The interview guide will be revised throughout the 3 qualitative time points of the study. All interviews will be audio recorded and later transcribed verbatim. Strengths, challenges and lessons learned with the CBE intervention also will be continually documented through ongoing consultation with the Community Advisory Committee.

Objective #5 – HDQ Property Assessment

Objective 5a –Assessing Interpretability of the HDQ

We will administer measures related to the interpretability assessment of the HDQ at 8 time points. We will pilot anchor based methods to assess the interpretability of cross-sectional and change scores of the HDQ. Specifically, we will determine the minimally clinically important difference (MCID), the 'smallest difference in score in the domain of interest which participants perceive as beneficial (or harmful)' (74). Anchor based methods require an independent standard (or anchor) that is interpretable and correlated with the instrument assessed (75). The anchor in this study will be a Global Rating of Change (GRC) Scale to determine changes in disability within participants (evaluative) and between participants (discriminative). Distribution based methods rely on statistical distributions of scores based on the magnitude of effect and some measure of variability in the results. Distribution based methods are not appropriate on their own but can be useful in demonstrating consistency with anchor based methods (75).

Cross-Sectional Interpretability of HDQ scores

We will assess the cross-sectional meaning of HDQ scores using two approaches:

1) *Minimum, Moderate and Severe Disability*: We will administer the HDQ followed by a **Disability Rating Scale** (Appendix T) that asks participants, for each disability domain and overall, whether they consider their disability as 'minimum', 'moderate' or 'severe'. Mean HDQ scores and ranges in each of the three categories will be interpreted as minimum, moderate and severe disability respectively.

2) *Easily Recognizable Health States:* We will assess the interpretability of HDQ scores by comparing HDQ scores based on three easily recognizable health states. We will assume that the mean difference in domain scores between participants with varying employment status, self-reported health, and comorbidities (measured by the demographic and disease questionnaire) will constitute the MCID (76).

Longitudinal Interpretability of HDQ Scores – MCID

We will administer the HDQ bimonthly. At 8 time points, we will administer two **Global Rating of Change (GRC) scales (Appendices V and W)**; the first will assess the *amount of change* in disability (Appendix U) and the second will assess the *importance of change* in disability (Appendix V). This is because the amount of change is not interchangeable with importance of change (77-78).With the GRC scales, we will ask participants whether their health-related challenges (or disability) were the same, better or worse than baseline for each HDQ domain as well as overall disability. For example; "*Has there been a change in your physical symptoms and impairments since you last completed the HDQ?*" (Response options: worse, about the same, or better). A response of 'no change' will be assigned to zero. If the response was better or worse, then participants will be asked to describe how much they improved or worsened on a seven point ordinal scale. Better or worse responses will be scored: a tiny bit, almost the same (=1); a little bit (=2); somewhat (=3); moderately (=4); quite a bit (=5); a great deal (=6); a very great deal (=7). Improvement/deterioration ratings will be assigned a positive/negative value, respectively.

Objective 5b - Assessing the Responsiveness of the HDQ

We will determine the responsiveness of the HDQ by assessing the longitudinal validity of change scores. A recent systematic review showed the MOS-HIV and EQ-5D are responsive to change between groups and/or over time in PHAs (79) and disease-specific specific HRQL instruments are more responsive than generic

HRQL measures across a wide range of patients and interventions (80). Hence, we will administer the MOS-HIV and EQ-5D bimonthly as reference measures and assess a priori hypothesized correlations between HDQ change scores and change scores on the reference measures to assess responsiveness of the HDQ (Appendix W) (81-82).

DATA ANALYSIS

We will calculate descriptive statistics for demographic characteristics as frequencies and proportions for categorical variables and means and standard deviations (normally distributed data) and medians and interquartile ranges (IQR) (non-normally distributed data) for continuous variables.

Objective 1 (Reach): We will report the number and proportion of individuals who express interest to participate in the CBE intervention. For proportion, we will obtain an estimate based on the total number of eligible PHAs from the recruitment sites. For representativeness, we will assess the characteristics of participants who engage in the program in relation to the broader HIV community comparing the sample to epidemiological data from the Public Health Agency of Canada (83). We will report the number, proportion and characteristics of participants who are eligible and agree to participate, the number of participants who withdraw from the program, and the reasons for withdrawal over time. We will assess differences in characteristics of those who complete versus withdraw from the intervention using independent t-tests and Mann Whitney U tests.

Objective 2 (Effectiveness):

Assessing Physical Health Symptoms and Impairment
We will assess statistical difference in scores (pre-post) by conducting paired t-tests with $p<0.05$. We will conduct a repeated measures ANOVA (3 data points) with $p<0.05$ to test for differences in outcomes across the 3 time points.

Assessing Disability and Health Status
We will calculate descriptive statistics for demographic characteristics as frequencies and proportions for categorical variables and means and standard deviations (normally distributed data) and medians and interquartile ranges (IQR) (non-normally distributed data) for continuous variables.

For all self-reported questionnaires, we will calculate the proportion of missing responses within individual cases and for each item across all participants. Participants with >15% missing responses in every questionnaire will be removed from the dataset. Otherwise missing responses will be dealt with using mean imputation. We will assess the distribution of domain and total scores pre and post-CBE intervention as well as change scores. We will calculate domain and total scores for each time point (median and interquartile range (IQR)). We will calculate the proportion of items at the floor or ceiling of the scales in order to determine floor or ceiling effects, respectively.

We will calculate HDQ disability presence, severity and episodic (calculated for descriptive purposes only) across the six domain and total scores (transformed out of 100) for each time point. Higher scores indicate a higher presence, severity and episodic nature of disability (84). Given we are simultaneously assessing the properties of the HDQ, we will calculate median (IQR) change scores of the responsiveness measures (MOS-HIV; EQ5D), mental and cognitive health measures (PHQ-9; MOS-Cognitive Scale) and contextual factor measures (MOS-Social Support; Stigma Scale, Brief Cope, Mastery). Statistical difference in scores (pre-post) will be determined by paired t-tests with $p<0.05$. We will conduct a repeated measures ANOVA (3 data points) with $p<0.05$ to test for differences in outcomes across the 3 time points.

Assessing Performance on the Neurocognitive Assessments

We will calculate means and standard deviations (normally distributed data) and medians and interquartile ranges (IQR) (non-normally distributed data) for continuous variables.

We will calculate the median and interquartile range (IQR) for the total scores (as per the unit of measurement for each test) for each time point. For The Patient's Assessment of Own Functioning, we will calculate the proportion of items at the floor or ceiling of the scale in order to determine floor or ceiling effects, respectively.

The neurocognitive assessments will be individually scored. We will calculate change in sum of correctly repeated sequences over the 3 time points (WMS-III Spatial Span Test), change in number of symbols correctly written over the 3 time points (WAIS-R Digit Symbol Test), change in T scores for the three learning trials, delayed recall total, recognition and discriminability over the 3 time points (Hopkins Verbal Learning Test), change in time to completion over the 3 time points (The Grooved Pegboard Test; The Trail Making Test), change in the number of correct sequences obtained over the 3 time points (The Letter-Number Sequencing Test) and change in scores ranging from 0-5 over the 3 time points (The Patient's Assessment of Own Functioning (PAOF)) (66-71). Higher PAOF scores indicate poorer perceived cognitive functioning (71). Statistical difference in scores (pre-post) will be determined by paired t-tests with $p<0.05$. We will conduct a repeated measures ANOVA (3 data points) with $p<0.05$ to test for differences in outcomes across the 3 time points.

Assessing Experience, Benefits and Impact of Exercise
We will conduct a content analysis to explore perceptions of PHA participants on their a) current experience with exercise, b) anticipated benefits of exercise, and c) perceived impact of CBE on their health and disability over time (91). We will analyze the transcripts cross-sectionally (at each time point) as well as longitudinally to identify changes in participant perceptions over time (92). All transcripts will be analyzed using line-by-line coding and codes clustered into broader categories (93). When all interview data are analyzed, we will formulate a summary. We will use NVivo software to facilitate analysis (94). We will review and refine findings with the Community Advisory Committee.

Quantitative Analysis (ITS): We will compare patterns of responses to the health and disability outcomes for the sample population at baseline (0-8 months) intervention (8-14 months) and post-intervention phases (14-22 months) using time series regression analytical techniques (52). 1) We will graph the mean and standard deviation scores for all health and disability outcomes taken bimonthly to determine the structure or trend of the data. 2) We will test for presence of autocorrelation (extent the data are dependent on each other) using the Durbin-Watson statistic (85). 3) If present, then we will adjust for autocorrelation. We will use the Prais-Winston estimator, which uses the generalized least-squares method (86) to determine regression estimates that express the *level* (value of outcome at the beginning of a segment; intercept) and *trend* (rate of change of outcome; slope) of the outcomes across each phase in the series in relation to the intervention. We will adjust for seasonality of exercise using dummy variables to define seasons. We will specifically determine the slope and 95% confidence interval for each phase and significance in the change in slope. We will evaluate each comparison separately (baseline to intervention; intervention to post-intervention) to determine the effect of CBE (87, 88). Regression coefficients corresponding to two standardized effect sizes will be obtained for each comparison: a change in *level* (also called 'step change') and a change in *trend* before and after the intervention. A change in level is defined as the difference between the observed level at the first intervention time point and that predicted by the pre-intervention time trend, and a change in trend is defined as the difference between post- and pre-intervention slopes (103). Statistical analysis will be conducted using SAS statistical software (89).

Hypotheses (*for primary outcome of VO2max*): 1) We hypothesize there will be a significant change ($p<0.05$) in the *level and trend* of VO2max between the baseline and intervention phases suggesting an intervention effect; 2) We hypothesize there will be a significant change in the *trend* of VO2max (decreased slope)

between the intervention and post-intervention phase suggesting a leveling off of the intervention effect in the post-intervention (or self-monitoring phase).

Objective 3 (Maintenance): ***Physical Activity:*** We will calculate median (IQR) change scores of the Rapid Assessment of Physical Activity Questionnaire (RAPA). Statistical difference in scores (pre-post) will be determined by paired t-tests with $p<0.05$ for both the Aerobic scale and the Strength and Flexibility scale. We will conduct a repeated measures ANOVA (3 data points) with $p<0.05$ to test for differences in outcomes across the 3 time points.
Adherence: Using the online weekly log, we will measure adherence to exercise over the intervention and self-monitoring phases. We will calculate the proportion of exercise sessions attended. Adherence will be defined as engaging in 75% or more of the 3 weekly exercise sessions throughout (90). We will also calculate proportion of participants who achieved the Canadian Physical Activity Guidelines (CPAG) of moderate to vigorous activity of at least 150 minutes a week for each of the study weeks; we will also calculate the proportion of weeks in which the majority of participants achieved the CPAG guidelines. We will additionally test for associations between adherence and health and disability outcomes.

Objective 4 (Effectiveness) & Objective 4 (Implementation and Adoption): *Qualitative Data Analysis:* We will conduct a content analysis to explore perceptions of PHA participants on the impact of the CBE intervention (*obj 2*) and perceptions of both PHA participants and recreation providers on the process of implementing the CBE intervention and its long-term sustainability (*obj 4*) (91). We will analyze the transcripts cross-sectionally (at each time point) as well as longitudinally to identify changes in participant perceptions over time (92). All transcripts will be analyzed using line-by-line coding and codes clustered into broader categories (93). When all interview data are analyzed, we will formulate a summary of perceived impact and the influence of extrinsic and intrinsic contextual factors on exercise behaviour (*obj 2*) and strengths and challenges associated with the CBE intervention and recommendations for long-term implementation in the community (*obj 4*). We will use NVivo software to facilitate analysis (94). We will review and refine findings with the Community Advisory Committee. The quantitative and qualitative data pertaining to effectiveness (*obj 2*) will be analyzed separately but concurrently and will be combined at the point of interpretation with the Advisory Committee.

CBE Intervention – Assessing the CBE Implementation and Assessment Process
The research team and Community Advisory Committee will meet throughout all phases of the project (start-up, recruitment, intervention and data collection, analysis, and knowledge translation) to assess the process (strengths, challenges and recommendations for revision) related to the recruitment strategy, eligibility screening process, CBE implementation, retention in the study, and outcome assessment. During teleconference and face-to-face team meetings, these discussions will be formally documented. Throughout the study we will keep an ongoing *CBE Study Process Log* to document any reflections or comments from participants or members of the study team regarding the feasibility and process of the study implementation.

Objective #5 Analysis

Objective 5a - Interpretability: We will use a self-perceived severity of disability plus two anchor based approaches to assess interpretability because these have been shown to yield different MCIDs (95).

<u>Cross-Sectional Interpretability</u>: We will assess the cross-sectional meaning of HDQ scores by assessing self-perceived severity of disability and differences in baseline HDQ scores between easily recognizable health status (e.g. employment status, self-perceived health, presence of comorbidities) (96).
a) *<u>Minimum, Moderate and Severe Disability</u>*: We will calculate mean HDQ scores (total and domain) and ranges for each of the three self-reported categories identified by participants (minimum, moderate and severe disability) within each HDQ domain, and overall rating of disability. Each of these three categories will be interpreted as minimum, moderate and severe disability respectively.
b) *<u>Easily Recognizable Health States</u>*: We will calculate the mean difference in HDQ domain scores between participants who are working versus not working; completed the HDQ on a good day versus bad day; and

those living with 0-1 comorbidity versus ≥2 comorbidities to estimate an MCID. These anchor based methods will yield estimates of MCID for each of the HDQ domain and total scores of which we will establish a range to estimate the cross-sectional MCID.

Longitudinal Interpretability: We will pilot anchor-based approaches (Global Change Score; ROC curve) to establish the MCID of change scores of the HDQ.

Longitudinal Anchor Based Methods #1 (Global Rating Change Scale): We will calculate the proportion of participants who improved, worsened, or remained unchanged for all HDQ domain and total scores using the GRC '*amount of change*' and GRC '*importance of change*' scales (97).We will then assess mean change scores for patients who did not change (0=no change); had small change (+/-1 to +/-3); moderate change (+/-4 to +/-5) and large change (+/-6 to +/-7) in disability (98).This will result in two GRC Disability 15-point scales that document *amount* and *importance* in change in disability (-7 to +7: a very great deal worse to a very great deal better) (98).GRC scales will be the 'anchor' from which to define small, moderate and large amount (and important) changes in disability. Mean differences in HDQ scores for those who change from +/-1 to +/-3 will constitute a pilot preliminary MCID (98).Results will yield 14 MCID estimates for *amount of change* for the six domain and total scores of the HDQ, seven each for improvement and deterioration in disability. Similarly, 14 MCID estimates will be calculated for the *importance of change* based on the GRC scale (98).Finally, we will calculate the level of agreement (Kappa) between the 'amount of change' and 'importance of change' scores among those who improved and worsened. Agreement coefficients will be interpreted as large (>0.8), moderate (>0.5-0.8) or small (0.2-0.5) (99).

Longitudinal Anchor Based Methods #2 (Receiver Operating Characteristic (ROC) Curve): We will use ROC curves to determine the most accurate change score for distinguishing between 'improved' or 'not improved' groups related to disability. This is the cutoff point that best discriminates between participants reporting or denying any improvement (or worsening) in disability. The optimal cutoff point will be estimated based on the point that maximizes the sum of sensitivity (proportion of people improved disability correctly identified as such on the HDQ scores) and specificity (proportion of people who did not improve disability correctly identified as such). The area under the curve (AUC) is considered the probability of correctly identifying the improved patient from randomly selected pairs of improved and unimproved patients. The ROC curve will be based on the GRC scale response (no change, improved, worsened) (100-101).We will conduct a separate analysis for improvement and deterioration in disability. We will consider an AUC of 0.7 as adequate (101).A limitation of this approach is that the external standard for the ROC must be dichotomous (e.g. improved or not improved) which provides limited information on the magnitude of change in disability (102).Nevertheless, this approach reflects the ability of an instrument to identify both a change and no change in disability (102).

Objective 5b - Responsiveness: We will assess correlations of change scores in the HDQ with change scores of related measures that we hypothesize are related to disability (60). We will test 5 primary *a priori* hypotheses and 4 exploratory *a priori* hypotheses about predicted relationships between change scores of measures that relate to disability (MOS-HIV, and EQ-5D) [convergent construct longitudinal validity] and those that may relate but conceptually differ from disability (Social Support Scale, Stigma Scale) [divergent construct longitudinal validity] with change scores of the HDQ. We will also test 3 *a priori* hypotheses about predicted relationships between HDQ change scores and scores on the GRC presence and GRC importance scales [known groups longitudinal validity] (Appendix W). To maximize the use of available data, we will assess the hypotheses with i) month 8 (baseline CBE intervention) and month 10 scores, ii) month 8 (baseline CBE intervention) and month 12 scores, and iii) month 8 (baseline CBE intervention) and month 14 scores. Correlation coefficients of ≥ 0.30, ≥ 0.50 and ≥ 0.70, will be defined as 'weak', 'moderate,' and 'strong,' respectively (104). We will consider the HDQ to possess construct validity if results confirm at least 75% of the predetermined hypotheses (101).

References:

29. Lohr KN, Aaronson NK, Alonso J, Burnam MA, Patrick DL, Perrin EB, et al. Evaluating quality-of-life and health status instruments: development of scientific review criteria. Clin Ther. 1996;18(5):979-92. Epub 1996/09/01.
30. Mokkink LB, Terwee CB, Patrick DL, Alonso J, Stratford PW, Knol DL, et al. The COSMIN checklist for assessing the methodological quality of studies on measurement properties of health status measurement instruments: an international Delphi study. Qual Life Res. 2010;19(4):539-49. Epub 2010/02/20.
31. Finch EP, Brooks D, Stratford P, Mayo N. Physical Rehabilitation Outcome Measures: A Guide to Enhanced Clinical Decision Making. Toronto, Ontario: Canadian Physiotherapy Association; 2002.
32. Guyatt GH, Feeny DH, Patrick DL. Measuring health-related quality of life. Ann Intern Med. 1993;118(8):622-9. Epub 1993/04/15.
33. Dragan S, Li A, McCabe T, Silverstein E, Salbach N, O'Brien KK. Factors to Inform the Development of a Community-Based Exercise Program for Adults Living with HIV: Exploring the Perspectives of Adults Living with HIV, Recreation Providers and Rehabilitation Professionals. University of Toronto: Connaught New Researcher Award, University of Toronto; 2014.
34. Garber CE, Blissmer B, Deschenes MR, et al. American College of Sports Medicine position stand. Quantity and quality of exercise for developing and maintaining cardiorespiratory, musculoskeletal, and neuromotor fitness in apparently healthy adults: guidance for prescribing exercise. *Med Sci Sports Exerc.* Jul 2011;43(7):1334-1359.
35. Prochaska JO, DiClemente CC. Stages and processes of self-change of smoking: toward an integrative model of change. *Journal of consulting and clinical psychology.* Jun 1983;51(3):390-395.
36. Elzarrad MK, Eckstein ET, Glasgow RE. Applying chronic illness care, implementation science, and self-management support to HIV. *American journal of preventive medicine.* Jan 2013;44(1 Suppl 2):S99-107.
37. Canadian Working Group on HIV and Rehabilitation. E-Module for Evidence-Informed HIV Rehabilitation (e-module). 2011.
38. Fitbit Inc. Fitbit® Official Site. 2014; http://www.fitbit.com/ca/zip.
39. O`Brien KK, Bayoumi A.M., King K., Alexander R., Solomon P. Community Engagement in Health Status Instrument Development: Experience with the HIV Disability Questionnaire. *Progress in Community Health Partnerships: Research, Education and Action.* In Press.
40. O'Brien KK, Bayoumi AM, Stratford P, Solomon P. Which dimensions of disability does the HIV Disability Questionnaire (HDQ) measure? A factor analysis. *Disabil Rehabil.* Aug 13 2014:1-9.
41. World Health Organization. World Health Organizations Disability Assessment Schedule II (WHO-DAS-II). 2001.
42. Brooks R, Rabin R, Charro F. The measurement and valuation of health status using EQ-5D: A European perspective. Dordrecht, The Netherlands: Kluwer Academic Publishers; 2003.
43. Meesters JJ, Verhoef J, Liem IS, Putter H, Vliet Vlieland TP. Validity and responsiveness of the World Health Organization Disability Assessment Schedule II to assess disability in rheumatoid arthritis patients. Rheumatology (Oxford). 2010;49(2):326-33. Epub 2009/12/03.
44. Wu AW, Hanson KA, Harding G, Haider S, Tawadrous M, Khachatryan A, et al. Responsiveness of the MOS-HIV and EQ-5D in HIV-infected adults receiving antiretroviral therapies. Health Qual Life Outcomes. 2013;11:42. Epub 2013/03/19.
45. Mathews WC, May S. EuroQol (EQ-5D) measure of quality of life predicts mortality, emergency department utilization, and hospital discharge rates in HIV-infected adults under care. Health Qual Life Outcomes. 2007;5:5. Epub 2007/01/27.
46. Kroenke K, Spitzer RL, Williams JB. The PHQ-9: validity of a brief depression severity measure. *J Gen Intern Med.* Sep 2001;16(9):606-613.
47. Shacham E, Nurutdinova D, Satyanarayana V, Stamm K, Overton ET. Routine screening for depression: identifying a challenge for successful HIV care. *AIDS Patient Care STDS.* Nov 2009;23(11):949-955.
48. Monahan PO, Shacham E, Reece M, et al. Validity/reliability of PHQ-9 and PHQ-2 depression scales among adults living with HIV/AIDS in western Kenya. *J Gen Intern Med.* Feb 2009;24(2):189-197.
49. Wu AW, Revicki DA, Jacobson D, Malitz FE. Evidence for reliability, validity and usefulness of the Medical Outcomes Study HIV Health Survey (MOS-HIV). *Qual Life Res.* Aug 1997;6(6):481-493.

50. Revicki DA, Chan K, Gevirtz F. Discriminant validity of the Medical Outcomes Study cognitive function scale in HIV disease patients. *Qual Life Res.* Aug 1998;7(6):551-559.
51. Yu Y, Yang J, Shiu CS, Simoni JM, Xiao S, Chen W, Rao D, Wang M. Psychometrict testing of the Chinese version of the Medical Outcomes Study Social Support Survey among people living with HIV?AIDS in China. Applied Nursing Research. 2015; 28(4): 328-333.
52. Abeysinghe T, Balasooriya U, Tsui A. Small-sample forecasting regression or ARIMA models? *Journal of quantitative economics : Journal of the Indian Econometric Society.* 2003;1:103-113.
53. Vosvick M, Koopman C, Gore-Felton C, Thoresen C, Krumboltz J, Spiegel D. Relationship of functional quality of life to strategies for coping with the stress of living with HIV/AIDS. Psychosomatics. 2003;44(1):51-8. Epub 2003/01/08.
54. Carver CS. You want to measure coping but your protocol's too long: consider the brief COPE. Int J Behav Med. 1997;4(1):92-100. Epub 1997/01/01.
55. Carver CS, Scheier MF, Weintraub JK. Assessing coping strategies: a theoretically based approach. J Pers Soc Psychol. 1989;56(2):267-83. Epub 1989/02/01.
56. Pearlin LI. Pearlin Mastery Scale. Journal of Health and Social Behavior 1978;**19**(1):2-21.
57. Pearlin LI, Radabaugh CW. Economic strains and the coping function of alcohol. The American Journal of Sociology. 1976;82(3):652-63.
58. Skaff MM, Pearlin LI, Mullan JT. Transitions in the caregiving career: Effects on sense of mastery. Psychology and Aging. 1996;11(2):247-57.
59. Adams KB, Smyth KA, McClendon MJ. Psychosocial resources as moderators of the impact of spousal dementia caregiving on depression. The Journal of Applied Gerontology. 2005;24(5):475-89.
60. Turner HA, Pearlin LI, Mullan JT. Sources and determinants of social support for caregivers of persons with AIDS. J Health Soc Behav. 1998;39(2):137-51.
61. O'Brien KK, Davis AM, Strike C, Young NL, Bayoumi AM. Putting episodic disability into context: a qualitative study exploring factors that influence disability experienced by adults living with HIV/AIDS. *J Int AIDS Soc.* 2009;12(1):5.
62. Berger BE, Ferrans CE, Lashley FR. Measuring stigma in people with HIV: psychometric assessment of the HIV stigma scale. Res Nurs Health. 2001;24(6):518-29. Epub 2001/12/18.
63. Sherbourne CD, Stewart AL. The MOS social support survey. Soc Sci Med. 1991;32(6):705-14. Epub 1991/01/01.
64. Wright K, Naar-King S, Lam P, Templin T, Frey M. Stigma scale revised: Reliability and validity of a brief measure of stigma for HIV+ youth. Journal of Adolescent Health. 2007;40(1):96-8.
65. Bunn JY, Solomon SE, Miller C, Forehand R. Measurement of stigma in people with HIV: A reexamination of the HIV stigma scale. AIDS Education and Prevention. 2007;19(3):198-209.
66. Wechsler, D. WMS-III Administration and Scoring Manual. The Psychological Corporation, San Antonio; 1997.
67. Wechsler, D. WAIS-R Manual. Wechsler Adult Intelligence Scale-Revised. The Psychological Corporation, San Antonio; 1981.
68. Brandt. The Hopkins Verbal Learning Test: Development of a new verbal memory test with six equivalent forms. *The Clinical Neuropsychologist, 1991;5,* 125-142.
69. Lezak, M. Neuropsychological Assessment, 3rd Edition. Oxford University Press, London; 1995.
70. Matthews, C., and Klove, H. (1964). *Instruction manual for the Adult Neuropsychology Test Battery*. University of Wisconsin Medical School, Madison, WI.
71. Chelune, G., Heaton, R., & Lehman, R. Neuropsychological and Personality Correlates of Patients' Complaints of Disability*. Advances in Clinical Neuropsychology Volume,* 1986;*3*; 95-126.
72. Turner-Stokes L. Goal Attainment Scaling (GAS) in Rehabilitation: A Practical Guide. *Clinical Rehabilitation.* 2009: 23(4):362-370.
73. Fitbit Inc. Fitbit® Official Site. 2014; http://www.fitbit.com/ca/zip.
74. Jaeschke R, Singer J, Guyatt GH. Measurement of health status. Ascertaining the minimal clinically important difference. Control Clin Trials. 1989;10(4):407-15. Epub 1989/12/01.
75. Guyatt GH, Osoba D, Wu AW, Wyrwich KW, Norman GR. Methods to explain the clinical significance of health status measures. Mayo Clinic proceedings Mayo Clinic. 2002;77(4):371-83. Epub 2002/04/09.
76. Guyatt GH. Making sense of quality-of-life data. Med Care. 2000;38(9 Suppl):II175-9. Epub 2000/09/12.

77. Stratford PL, Riddle DL. Assessing the amount of change in an outcome measure is not the same as assessing the importance of change. Physiotherapy Canada. 2013;63(3):244-7.
78. Kamper SJ, Maher CG, Mackay G. Global Rating of Change Scales: A Review of Strengths and Weaknesses and Considerations for Design. *J Man Manip Ther.* 2009; 17(3): 163-170.
79. Swendeman D, Ingram BL, Rotheram-Borus MJ. Common elements in self-management of HIV and other chronic illnesses: an integrative framework. *AIDS Care.* Oct 2009;21(10):1321-1334
80. Wiebe S, Guyatt G, Weaver B, Matijevic S, Sidwell C. Comparative responsiveness of generic and specific quality-of-life instruments. J Clin Epidemiol. 2003;56(1):52-60. Epub 2003/02/19.
81. Mokkink LB, Terwee CB, Knol DL, et al. The COSMIN checklist for evaluating the methodological quality of studies on measurement properties: a clarification of its content. *BMC Med Res Methodol* 2010;10:22.
82. de Vet HC, Ader HJ, Terwee CB, et al. Are factor analytical techniques used appropriately in the validation of health status questionnaires? A systematic review on the quality of factor analysis of the SF-36. *Qual Life Res* 2005;14:1203-1218; discussion 1219-1221, 1223-1204.
83. Public Health Agency of Canada. Summary: Estimates of HIV Prevalence and Incidence in Canada. 2011.
84. O'Brien KK, Alexander, R., King, K., Murray, J., Tebeje, M., Bayoumi, A.M., Bereket, T., Swinton, M., Norman, G., Solomon, P. Developing a new HIV disability questionnaire: a community-integrated approach. . Physiotherapy Canada. 2010;62(Supplement 1):34-5.
85. Durbin J. Testing for serial correlation in least-squares regressions when some of the regressors are lagged dependent variables. *Econometrica.* 1970;38(3):410-421.
86. Prais SJ, Winston CB. Trend estimators and serial correlation. *Cowles Commission Discussion Paper.* 1954;383.
87. Michielutte R, Shelton B, Paskett ED, Tatum CM, Velez R. Use of an interrupted time-series design to evaluate a cancer screening program. *Health education research.* Oct 2000;15(5):615-623.
88. Zhang F, Wagner AK, Soumerai SB, Ross-Degnan D. Methods for estimating confidence intervals in interrupted time series analyses of health interventions. *J Clin Epidemiol.* Feb 2009;62(2):143-148.
89. *SAS Computer Software 9.2* [computer program]2008.
90. Hicks GE, Benvenuti F, Fiaschi V, et al. Adherence to a community-based exercise program is a strong predictor of improved back pain status in older adults: an observational study. *The Clinical journal of pain.* Mar-Apr 2012;28(3):195-203.
91. Hsieh HF, Shannon SE. Three approaches to qualitative content analysis. *Qual Health Res.* Nov 2005;15(9):1277-1288.
92. Molloy D, Woodfield K, Bacon J. *Longitudinal qualitative research approaches in evaluation studies*. London: Her Majesty's stationary office; 2002.
93. Strauss A, Corbin J. *Basics of Qualitative Research: Techniques and Procedures for Developing Grounded Theory.* Vol 2nd edition. Thousand Oaks, California: Sage Publications; 1998.
94. *NVivo qualitative data analysis software. Version 7* [computer program]: QSR International Pty. Ltd; 2006.
95. Kovacs FM, Abraira V, Royuela A, Corcoll J, Alegre L, Tomas M, et al. Minimum detectable and minimal clinically important changes for pain in patients with nonspecific neck pain. BMC musculoskeletal disorders. 2008;9:43. Epub 2008/04/12.
96. Guyatt GH. Making sense of quality-of-life data. Med Care. 2000;38(9 Suppl):II175-9. Epub 2000/09/12.
97. Guyatt GH, Osoba D, Wu AW, Wyrwich KW, Norman GR. Methods to explain the clinical significance of health status measures. Mayo Clinic proceedings Mayo Clinic. 2002;77(4):371-83. Epub 2002/04/09.
98. Jaeschke R, Singer J, Guyatt GH. Measurement of health status. Ascertaining the minimal clinically important difference. Control Clin Trials. 1989;10(4):407-15. Epub 1989/12/01.
99. Cohen J. Statistical Power analysis for the behavioral sciences. 2nd edition ed. New Jersey: Lawrence Erlbaum Associated; 1988.
100. Deyo RA, Diehr P, Patrick DL. Reproducibility and responsiveness of health status measures. Statistics and strategies for evaluation. Control Clin Trials. 1991;12(4 Suppl):142S-58S. Epub 1991/08/01.
101. Deyo RA, Centor RM. Assessing the responsiveness of functional scales to clinical change: an analogy to diagnostic test performance. Journal of chronic diseases. 1986;39(11):897-906. Epub 1986/01/01.

101. Terwee CB, Bot SD, de Boer MR, van der Windt DA, Knol DL, Dekker J, et al. Quality criteria were proposed for measurement properties of health status questionnaires. J Clin Epidemiol. 2007;60(1):34-42. Epub 2006/12/13.
102. Husted JA, Cook RJ, Farewell VT, Gladman DD. Methods for assessing responsiveness: a critical review and recommendations. J Clin Epidemiol. 2000;53(5):459-68. Epub 2000/05/17.
103. Ramsay CR, Matowe L, Grilli R, Grimshaw JM, Thomas RE. Interrupted time series designs in health technology assessment: lessons from two systematic reviews of behavior change strategies. *Int J Technol Assess Health Care.* Fall 2003;19(4):613-623.
104. Landis JR, Koch GG. The measurement of observer agreement for categorical data. Biometrics. 1977;33:159-74.

(b) Attach a copy of all questionnaires, interview guides and/or any other instruments.

(c) Include a **list of appendices** here for all additional materials submitted (e.g., Appendix A – Informed Consent; Appendix B – Interview Guide, etc.):

Appendix A – Additional Investigators
Appendix B – Log for Coaches
Appendix C – Online Exercise Log for Study Participants
Appendix D – Exercise Log for Study Participants (Pen-Pencil Version) – Optional to assist with online log
Appendix E – Overview of Research Procedure
Appendix F – Intervention and Assessment Timeline
Appendix G – Study Timeline
Appendix H – Episodic Disability Framework
Appendix I - Overview of Outcome Assessments, Procedures and Questionnaires
Appendix J – Self Reported Questionnaires
Appendix K1 –HIV Participant Demographic Questionnaire (Version 1 – Baseline)
Appendix K2 – HIV Participant Demographic Questionnaire (Version 2 –After Baseline)
Appendix L – Neurocognitive Assessment Administration Procedure
Appendix M – Qualitative Interview Guide
Appendix N - Intake CBE Intervention Questionnaire
Appendix O- Goal Attainment Scale
Appendix P1- Phase One Completion/Withdrawal Questionnaire
Appendix P2 - Phase Two Completion/Withdrawal Questionnaire
Appendix P3 - Phase Three Completion/Withdrawal Questionnaire
Appendix Q – Rapid Assessment of Physical Activity Questionnaire (RAPA)
Appendix R– E-Mail to Participants to Complete Online Exercise Log
Appendix S – Thank You/Reminder Email for Online Exercise Log
Appendix T– Disability Rating Scale
Appendix U - Global Rating of Change Scale-Amount of Change in Disability
Appendix V - Global Rating of Change Scale-Importance of Change in Disability
Appendix W - *A Priori* Hypotheses for HDQ Responsiveness Assessment (Longitudinal Validity)
Appendix X - Physical Activity Readiness Questionnaire (PAR-Q)
Appendix Y– Recruitment Poster
Appendix Z- Recruitment Brochure
Appendix AA - Recruitment Card
Appendix BB - Information Letter and Consent Form for Participants
Appendix CC - Outline of the Recruitment Process and Initial Assessments
Appendix DD – E-Mail Requesting Participation for Interviews
Appendix EE – Information Letter and Agreement Form to Participate in Interview - PHA
Appendix FF – Information Letter and Agreement Form to Participate in Interview – Recreation Provider

Appendix GG - Information Letter and Agreement Form to be Contacted about Future Phases of this Study
Appendix HH - E-Mail to Participants Requesting Permission to be Contacted about Future Phases of this Study
Appendix II – Treadmill Walking Test
Appendix JJ – One Mile Walk Test
Appendix KK – Recruitment E-Mail

13. PARTICIPANTS AND/OR DATA

(a) Describe the participants to be recruited, or the individuals about whom personally identifiable information will be collected. List the inclusion and exclusion criteria. Where the research involves extraction or collection of personally identifiable information, please describe from whom the information will be obtained, what it will include, and how permission to access the data is being sought. (Strategies for recruitment are to be described in section #15.) Where applicable, justify the sample size.

Participants will include men and women (18 years and older) living with HIV in Toronto who consider themselves medically stable and safe to engage in exercise and who are willing to participate in a 22 month study involving a 14 month CBE intervention at the YMCA. Interested individuals will complete the self-reported **Physical Activity Readiness Questionnaire** (PAR-Q) to self-assess whether they are safe to participate in exercise (103) (Appendix X). As per the scoring of the PAR-Q, if individuals respond 'yes' to one or more of the 7 items in the questionnaire, they will be asked to talk with their physician by phone or in person before they enroll in the study and complete the baseline assessment (see PAR-Q - Appendix X). If individuals answered 'no' to all PAR-Q items, according to the PAR-Q scoring they can be 'reasonably sure' that they can start becoming more physically active (beginning slowly and building gradually) and take part in a fitness appraisal. We will document any reported consultation by individuals with their physician about enrollment in the study (although written documentation from a physician will not be required for participation in the study). We will recommend all eligible participants inform their physician about their involvement in the study. All eligible participants will be asked to notify the study team if their health changes in a way that would result in a 'yes' response to any items of the PAR-Q. In these situations, individuals will be asked to consult their physician before continuing physical activity in the study. We will document participant consultation with their physician and/or any other members of the health provider team throughout the study. This is similar to our process piloted in the earlier pilot phase of this study.

To evaluate the *process* of CBE implementation, we will include recreation providers (managers and fitness instructors) from community organizations who engaged in the CBE implementation (objective #4).

Quantitative Assessment (ITS):
In ITS, sample size refers to the number of observational time points rather than number of participants. For a model of 12 time points, to detect both level and trend change, assuming an effect size of 1, equal pre and post intervention time periods, statistical significance $p<0.05$ and an autoregression error time series model with lag 1, and autocorrelation estimate of 0.3, we would expect a power of 0.80 (104-105). At least 3 data collection time points will be required to calculate a slope for the outcomes in each of the three phases (baseline, intervention and follow-up phases). We will aim to recruit approximately 120 adults living with HIV with the goal for 75 participants (62%) to complete the study; the number of recommended observations in ITS to achieve acceptable variability of the estimates at each time point (106). This is due to the observed ~61% retention rate observed in the pilot study (19 of the 31 eligible participants completed the study at 4 months). This is also what was determined feasible for the YMCA to accommodate given YMCA staff will be responsible for administering the fitness assessments and providing weekly coaching with participants. We will recruit through a combination of community organizations (Toronto PWA Foundation, AIDS Committee of Toronto), specialty hospital (Casey House), community (YMCA) and the Canadian Working Group on HIV and Rehabilitation in Toronto, Ontario. No recruitment or data collection will occur at St. Michael's Hospital, McMaster University or the University Health Network. Recruitment will occur via e-mail, posters, brochures and recruitment cards placed at the YMCA, Casey House, Toronto PWA, CWGHR and other community

collaborator organizations. We will tailor our recruitment to ensure diversity of our sample for gender, ethnocultural background, age, and length of time since diagnosis.

We will plan to recruit 120 participants across 4 monthly waves (30 participants per wave) to accommodate the intake and assessment capacity (fitness assessments) at the YMCA. We will closely monitor withdrawals throughout the baseline phase (8 months) and if needed propose to recruit an additional wave of up to 30 participants in order for ~100 participants to enter the intervention phase of the study.

Qualitative Interviews:
We will recruit a sub-sample of 15 PHAs who engaged in the CBE intervention and 5 fitness instructors and managers involved in the intervention to participate in 3 interviews. Previous work with PHAs and providers suggest this number of participants will enable us to reach saturation as it relates to the strengths and challenges of implementing the CBE intervention (107).

Reference:
103. Warburton DER, Jamnik VK, Bredin SSD, Gledhill N, on behalf of the PAR-Q+ Collaboration. The Physical Activity Readiness Questionnaire for Everyone (PAR-Q+) and Electronic Physical Activity
104. Zhang F, Wagner AK, Ross-Degnan D. Simulation-based power calculation for designing interrupted time series analyses of health policy interventions. *J Clin Epidemiol.* Nov 2011;64(11):1252-1261.
105. McLeod AI, Vingilis ER. Power computations in time series analyses for traffic safety interventions. *Accident; analysis and prevention.* May 2008;40(3):1244-1248.
106. Wagner AK, Soumerai SB, Zhang F, Ross-Degnan D. Segmented regression analysis of interrupted time series studies in medication use research. *Journal of clinical pharmacy and therapeutics.* Aug 2002;27(4):299-309.
107. O'Brien KK, Bayoumi AM, Bereket T, et al. Sensibility assessment of the HIV Disability Questionnaire. *Disabil Rehabil.* Apr 2013;35(7):566-577.

(b) Is there any group or individual-level vulnerability related to the research that needs to be mitigated (for example, difficulties understanding informed consent, history of exploitation by researchers, power differential between the researcher and the potential participant)?

Participants may include persons whom Kelly O'Brien, Ahmed Bayoumi, Patty Solomon, Aileen Davis, Ada Tang or Sean Rourke worked with as a colleague in a community-based research or educational capacity. The Knowledge Users in the study is the General Manager (Mehdi Zobeiry) and Program Manager (Katie Lowe) at the Central YMCA in Toronto. Study co-investigators, who have no relationship with the potential participant, will obtain consent. Interested participants will be invited to contact the study co-investigators or research coordinator who has no relationship with the potential participants, who will discuss the study in detail and if applicable, will obtain consent.

14. EXPERIENCE OF INVESTIGATORS WITH THIS TYPE OF RESEARCH

(a) Please provide a brief description of previous experience with this type of research by (i) the principal investigator, (ii) the research team and (iii) the people who will have direct contact with the participants. If there has not been previous experience, please describe how the principal investigator/research team will be prepared.

This research is being conducted by a team of researchers. Nominated Principal Applicant**, Kelly O'Brien**, is a physical therapist and Assistant Professor at the Department of Physical Therapy, University of Toronto and CIHR New Investigator. This research builds on her Cochrane Collaboration work on HIV and exercise, *HIV Health and Rehabilitation Survey*, and pilot work developing the CBE intervention. Kelly will be responsible for the overall coordination of the team, implementation of the research, analysis, hiring and supervision of staff, and overseeing the budget. Co-Applicant **Patty Solomon** is a Professor at McMaster University, who

brings 15 years of experience in HIV and rehabilitation research, qualitative research and knowledge translation (KT), has expertise in aging and disability, and will formulate the core research team. Co-Applicant **Ahmed Bayoumi** is a general internist and scientist at the Centre for Research on Inner City Health at St. Michael's Hospital (SMH) with a wealth of experience in HIV health services research. Ahmed is a clinician at the Positive Care Clinic at SMH, has been involved in the earlier phases of this research and will remain a member of the core research team. Co-Applicant **Aileen Davis** is a Professor at the University of Toronto and Senior Scientist in the Division of Health Care and Outcomes Research Unit at the University Health Network. She is a Clinical Epidemiologist and expert in complex chronic disease and evaluating interventions in population health. Dr. Davis will provide statistical expertise in ITS. Co-Applicant **Ada Tang** is a physical therapist and Assistant Professor at McMaster University with expertise in assessing the effect of CBE in people with stroke. Ada will advise on the intervention and provide expertise in the measurement of the health outcomes. **Sean Rourke** is a Research Scientist at the Centre for Research on Inner City Health at St. Michael's Hospital with extensive experience in community-based HIV/AIDS. As the head of the Neurobehavioural Research Unit, Sean has expertise in the treatment of neurobehavioural complications associated with HIV. Sean will be a member of the research team specifically directing the administration and analysis of neurocognitive screening assessments. Knowledge Users **Mehdi Zobeiry** (Principal Knowledge User) is the Vice President and General Manager and **Katie Lowe**, is the Program Manager at the Central Toronto YMCA. Mehdi and Katie bring a wealth of expertise on community-based exercise and health promotion through his work at the YMCA and are invested in the enhancement of their programs to better serve the HIV community. **Ayesha Nayar** is the Research Coordinator. She will have direct contact with the participants during the administration of the questionnaires.

(b) For projects that will involve community members (e.g., peer researchers) in the collection and/or analysis of data, please describe their status within the research team (e.g., are they considered employees, volunteers or participants?) and what kind of training they will receive?

All aspects of this program of research are guided by a Community Advisory Committee. This committee is comprised of people living with HIV, representatives from AIDS Service Organizations, the Canadian Working Group on HIV and Rehabilitation, Casey House, and the Ontario Ministry of Health and Long-Term Care. The committee has advised, and will continue to advise the investigators on all phases of this research, which is part of the larger HIV Disability Questionnaire Study. This committee will help to enhance the applicability of findings to individuals living with HIV and suggest implications this research might have on advancing HIV policy and practice.

15. RECRUITMENT OF PARTICIPANTS

- Where there is recruitment, please describe how, by whom, and from where the participants will be recruited
- Where participant observation is to be used, please explain the form of insertion of the researcher into the research setting (e.g., living in a community, visiting on a bi-weekly basis, attending organized functions)
- If relevant, describe any translation of recruitment materials, how this will occur and whether or not those people responsible for recruitment will speak the language of the participants.
- **Attach a copy of all posters, advertisements, flyers, letters, e-mail text, or telephone scripts to be used for recruitment.**

Participants

We will recruit 120 men and women living with HIV in Toronto from the Central Toronto YMCA, Casey House, Toronto PWA Foundation and CWGHR using e-mail (Appendix KK), recruitment posters (Appendix Y), brochures (Appendix Z) and recruitment cards (Appendix AA). Managers from the YMCA, Toronto PWA, Casey House and CWGHR will display recruitment posters at their facility. Additional participants may be recruited via snowball sampling by participants who know other individuals from the Toronto HIV community.

Interested PHAs will be invited to contact Kelly O'Brien's research lab at the University of Toronto by email or phone. At the end of the pilot study, 21 participants provided consent to be contacted about future phases of this study. We will contact these interested individuals to see if they would like to participate in the screening process.

Interested individuals will be asked to contact the research lab by email or telephone to set up an 'Initial CBE Study Screening Meeting' with the Research Coordinator to discuss eligibility and details associated with the study. During this meeting, the interested individual will complete the Physical Activity Readiness Questionnaire (PARQ) (Appendix X) and then thoroughly go over the eligibility criteria, including level of commitment, details of the study and the information letter and consent form with the Research Coordinator (Appendix BB). Eligibility (including willingness to commit to the 22 month CBE program) will be assessed during this meeting as we aim to maximize retention of participants in the study.

Potential participants will be informed that participation in the study is voluntary and that they have the right to withdraw at any time with no negative consequences. For the adults living with HIV who are members of the YMCA, CWGHR, Casey House or Toronto PWA, withdrawal will not affect the services that they receive at these sites. If participants withdraw, the information that we receive up to the point of withdrawal from the study may be used for data analysis regardless of its completeness unless the participant wishes to have it removed. If participants decide to withdrawal we will ask them to complete the final Phase One/Two/Three Completion/Withdrawal Questionnaire depending on which month they withdraw from the study. Participants also have the right to refrain from answering questions that they do not feel comfortable with as well as not participate in any physical aspect of the intervention that they are not comfortable with. This meeting will be conducted at the UofT, YMCA, or Toronto PWA. Interested individuals will have the opportunity to take this information away and take their time to decide whether they would like to participate in this study.

At the end of the screening meeting, interested and eligible individuals will be informed that they are able to sign the consent form at that time or take more time in deciding about their participation and schedule another meeting (face-to-face or via telephone) with the Research Coordinator to indicate consent at a later time. Individuals who decide to participate will be asked to sign a written informed consent. Participants can e-mail the Research Coordinator their signed consent form if and whenever ready. Participants will be asked to sign two copies of the consent form, one will be for the researchers and the other will be given to the participant.

<u>Assessment Session #1</u>: After consent, participants will meet with the Research Coordinator to complete the baseline self-reported questionnaires. At this time participants will complete the Intake CBE Questionnaire (Appendix N) and the Baseline Demographic Disease Questionnaire (Version 1) (Appendix K1), the other disability, health and contextual factor questionnaires (Appendix J).

<u>Assessment Session #2</u> (YMCA): Within the same week of completing the questionnaire assessment (baseline), the Research Coordinator will liaise with the YMCA fitness staff regarding incoming participants and schedule the initial Fitness Assessment.

Prior to the beginning of the intervention (8 months), the Research Coordinator will liaise with the YMCA fitness staff regarding the beginning of the study and will schedule the initial meeting between the participant and the YMCA fitness instructor depending on pre-determined availability of the YMCA staff and participant.

During this initial YMCA meeting, fitness instructors will work with participants to assess their goals and establish an individualized exercise program that will include aerobic, resistive, neuromotor and flexibility training. Participants will also complete the baseline assessment-physical assessment at the YMCA.

We may send up to three reminders to participants to set up the Screening Meeting. Participants who indicate that they are not interested or do not respond after these three requests, will not be further approached about participation. For all other assessment meetings, we will contact participants by phone or email (based on

their preference) to remind them of the date and time of their assessment appointment. If participants miss their appointment, we will follow up with three attempts to reschedule the appointment. If we do not hear back after these three attempts, we will wait two weeks and follow up two more times asking for participants to re-affirm their interest and willingness to participate in the study, and if they choose to withdrawal, to indicate any reasons for withdrawal of the study. Please see Appendix CC for an outline of the recruitment process and initial assessments.

We will plan to recruit 120 participants across 4 monthly waves (30 participants per wave) to accommodate intake and assessment capacity at the YMCA. We will closely monitor withdrawals throughout the baseline phase (8 months) and if needed propose to recruit an additional wave of up to 30 participants in order for ~100 participants to enter the intervention phase of the study.

PHA Participants and Recreation Provider Participants

We will also conduct face-to-face interviews with a sample of 5 recreation providers (managers, fitness instructors) at and 15 PHA participants at initiation, midway, and completion of the 6-month CBE intervention.

We will contact all Fitness Instructors and Managers involved in this study to reach our target sample size. For the qualitative interviews with PHA participants and recreation providers, we will make initial contact with a subsample of PHA participants, recreation providers and managers via e-mail (Appendix DD), or by telephone (depending on their stated preferred method). We will provide participants and recreation providers with a 'Qualitative Interview' Information Letter and Consent Form (Appendix EE and FF) and indicate they are able to sign the consent form at that time or take more time in deciding about their participation in these interviews and schedule another meeting (face-to-face or via telephone) with the Research Coordinator to indicate consent at a later time. Potential participants will be informed that participation in the study is voluntary and that they have the right to withdraw at any time with no negative consequences.

Individuals who agree to participate in the interviews will be asked to sign the Information Letter and Consent Form. Participants can e-mail the Research Coordinator their signed consent form if and whenever they are ready. Participants will be asked to sign two copies of the consent form, one will be for the researchers and the other will be given to the participant. We may send up to two reminder e-mails to participants who do not respond to the initial e-mail.

16. COMPENSATION

Please see U of T's Compensation and Reimbursement Guidelines.

(a) Will participants receive compensation for participation?

Financial	Yes ☐	No ☒
In-kind	Yes ☒	No ☐
Other	Yes ☐	No ☒

(b) If **Yes**, please provide details and justification for the amount or the value of the compensation offered.

We will offer each PHA participant a 14 month open access YMCA membership as a token of appreciation for their participation in the study. The membership will be provided in two waves. The first membership will be for 6 months (intervention phase) and the second membership will be for 8 months (post-intervention phase). We will not compensate participants in the baseline monitoring phase or for the bimonthly assessments. The value of the 14 month membership is $1028.02 (The YMCA community membership is $73.43 per month, all taxes included). Participants will be notified of this token at the initial screening meeting, prior to consenting to the study. At the end of the 14 months, the YMCA is committed to ensuring

that participants are able to sustain their access to the facility and participants may follow-up with the YMCA to determine flexible payment options in accordance with their community programs.

Participants also will be able to keep the Fitbit wireless activity monitor provided in Phase Two as a token of appreciation for their participation in the study.

The YMCA and CIHR study budgets do not possess the funds to support additional compensation for participation in the study. Providing additional monetary compensation may introduce a financial incentive to exercise which could potentially influence the adherence to exercise (Farooqui et al, 2014; Effects of financial incentives on motivating physical activity among older adults: results from a discrete choice experiment. *BMC Public Health*. 2014. 14:141. http://www.biomedcentral.com/1471-2458/14/141).

The aim of the study is to determine the translation of a CBE intervention into the 'real world' community setting to assess the long-term sustainability and adoption of CBE among people living with HIV. With this said, we aim to document the reasons for participation, retention and attrition throughout the exercise study, of which engagement in the outcome assessments will be considered. Responses will help to inform adaptations and considerations for the larger full study CBE intervention and protocol.

Participants and recreation providers who complete the interview sessions will receive a $25 E-Gift Card to Starbucks or Tim Hortons at the end of each interview.

(c) If **No**, please explain why compensation is not possible or appropriate.

NA

(d) Where there is a withdrawal clause in the research procedure, if participants choose to withdraw, how will compensation be affected?

Participants will be required to initiate the study (complete the baseline assessment), remain in the study for the baseline monitoring phase (0-8 months) and complete the bimonthly assessments in the baseline phase to receive the 6 month YMCA membership (for the intervention phase). Participants will be required to remain in the study for the intervention phase (8-14 months) and complete the bimonthly assessments in the intervention phase to receive the additional 8 month membership (for the post-intervention phase).

If participants choose to withdraw during the first 8 months, they will not receive the 14 month membership. If participants decide to withdraw during the intervention phase (8-14 months), they will still maintain their 6 month membership but will not receive the following 8 month membership. If participants decide to withdraw during the post-intervention self-monitoring exercise phase, they will still be able to maintain their 8 month YMCA membership. This is to ensure that we have enough funds in the study to recruit additional participants to the study if needed so that we may be able to attain the minimum sample size required for the analysis.

Given the 22 month timeframe of the study it is important to ensure that participants remain committed to attending the exercise sessions and assessments in order to accurately collect the data required to achieve our minimum sample size required for analysis (75 participants at study completion). Hence, in order to remain in the study, participants will be required to:

- Complete at least three of the four assessments (Fitness and Questionnaire Assessment) during Phase One to continue on to Phase Two. Complete at least three of the four assessments (Fitness and Questionnaire Assessments) during Phase Two to continue on to Phase Three.
- Provide at least 24 hours' notice of cancellation for any Fitness Assessments or exercise sessions with fitness instructors throughout the study. If participants are unable to provide this notice for up to 7 fitness assessments or exercise sessions throughout the 22 month study, you may be excluded from the study and your membership will be cancelled (if applicable). This is so that we can recruit additional participants to the study if needed.

- Attend exercise sessions three times a week (1.5 hours each) to the best of their ability.
- Attend the 6 educational sessions in Phase 2 to the best of their ability.

Note we understand the episodic and sometimes unpredictable nature of living with HIV and therefore understand last minute cancellations may occur due to health reasons. These circumstances will be taken into consideration with when evaluating ongoing eligibility for the study.

Participants will receive a $25 E-Gift Card at each interview session for a total of $75. If participants initiate but choose to withdrawal during an interview, they will still receive the $25 E-Gift Card associated with that interview.

SECTION C –DESCRIPTION OF THE RISKS AND BENEFITS OF THE PROPOSED RESEARCH

17. POSSIBLE RISKS

(a) Please indicate all potential risks to participants as individuals or as members of a community that may arise from this research:

(i) Physical risks (e.g., any bodily contact or administration of any substance): Yes ☒ No ☐

(ii) Psychological/emotional risks (e.g., feeling uncomfortable, embarrassed, or upset): Yes ☒ No ☐

(iii) Social risks (e.g., loss of status, privacy and/or reputation): Yes ☒ No ☐

(iv) Legal risks (e.g., apprehension or arrest, subpoena): Yes ☐ No ☒

(b) Please briefly describe each of the risks noted above and outline the steps that will be taken to manage and/or minimize them.

It is possible that participants will experience injury with exercise and the physical assessments. If this does occur, fitness staff from the YMCA will follow appropriate emergency procedures (in accordance with the YMCA general safety procedures and guidelines). In these situations, participants also will be asked to follow-up with their attending physician.

It is also possible that participants may find some of the questions on the questionnaires to be personal or sensitive in nature. Participants can choose not to complete any of the physical assessments or choose not to answer questions and may end the intervention at any time. If the participants find themselves becoming very upset during this study, the investigators will recommend discussing their feelings with their health care professional (e.g. physician), qualified counselor, or local public support group if available. If the participants have difficulty contacting a health care professional, qualified counselor or local community health center, they may contact the principal investigator for further assistance. In this situation, the study team will facilitate linkages to supportive services at the Toronto PWA Foundation which is a collaborator in this study.

Finally, given the individual and group nature of the CBE intervention, and the monthly self-management information sessions, it is possible that participants may feel a loss of privacy related while participating in part of the YMCA programs on site. During the consent process, we will indicate to participants that all individuals involved in this study including other participants, researchers, knowledge users, and the fitness instructors and assessment staff at the YMCA, will know that participants in this study are HIV positive.

18. POSSIBLE BENEFITS

- Describe any potential direct benefits to participants from their involvement in the project
- Describe any potential direct benefits to the community (e.g., capacity building)

- Comment on the potential benefits to the scientific/scholarly community or society that would justify involvement of participants in this study

Participants may experience direct health benefits from the CBE intervention. Participants may experience an overall improvement in health including cardiopulmonary fitness, strength, weight and body composition, flexibility, and quality of life, mental health symptoms and impairments (depression), and cognitive health symptoms and impairments.

Participants may also experience social benefits as they may find it rewarding to share their experiences and perspectives with other participants. This CBE intervention has the potential to foster social interaction, support, encouragement to exercise and promote emotional, cognitive and behavioural self-management strategies.

SECTION D – INFORMED CONSENT

19. CONSENT PROCESS

(a) Describe the process that will be used to obtain informed consent and explain how it will be recorded. Please note that it is the quality of the consent, not the form that is important. The goal is to ensure that potential participants understand to what they are consenting.

(b) If the research involves extraction or collection of personally identifiable information from or about a research participant, please describe how consent from the individuals or authorization from the data custodian (e.g., medical records department, district school board) will be obtained.

Initial recruitment will occur via e-mail (Appendix KK), posters (Appendix Y), brochures (Appendix Z) and recruitment cards (Appendix AA) placed at recruitment sites. Snowball recruitment may also occur.

Interested individuals will be asked to contact Kelly O'Brien's research lab by email or telephone to set up an 'Initial CBE Study Screening Meeting' with the Research Coordinator to discuss the eligibility and details associated with the study. This meeting will be conducted at the UofT, YMCA, or Toronto PWA. Interested individuals will have the opportunity to take this information away and take their time to decide whether they would like to participate in this study.

During this meeting, the interested individual will complete the Physical Activity Readiness Questionnaire (PARQ) (Appendix X) and then thoroughly go over the eligibility criteria, level of commitment, details of the study and the information letter and consent form with the Research Coordinator (Appendix BB). Eligibility (including willingness to commit to the 22 month CBE program) will be assessed during this meeting as we aim to retain participants.

Potential participants will be informed that participation in the study is voluntary and that they have the right to withdraw at any time with no negative consequences. For the adults living with HIV who are members of the YMCA, CWGHR, Casey House or Toronto PWA (recruitment sites), withdrawal will not affect the services that they receive at these sites. If participants withdraw, the information that we receive up to the point of withdrawal from the study may be used for data analysis regardless of its completeness unless the participant wishes to have it removed. Participants also have the right to refrain from answering questions that they do not feel comfortable with as well as not participate in any physical aspect of the intervention that they are not comfortable with.

Participants will be informed that they will need to initiate the study (complete the baseline assessment), remain in the study for the baseline monitoring phase (0-8 months) and complete the bimonthly assessments in the baseline phase to receive the 6 month YMCA membership (for the intervention phase). Participants will

be required to remain in the study for the intervention phase (8-14 months) and complete the bimonthly assessments in the intervention phase to receive the additional 8 month membership (for the post-intervention phase).

If participants choose to withdraw during the first 8 months, they will not receive the 14 month membership. If participants decide to withdraw during the intervention phase (8-14 months), they will still maintain their 6 month membership but will not receive the following 8 month membership. If participants decide to withdraw during the post-intervention self-monitoring exercise phase, they will still be able to maintain their 8 month YMCA membership. This is to ensure that we have enough funds in the study to recruit additional participants if needed so that we may be able to attain the minimum sample size required for the analysis.

Participants will also be informed that if they consent, their Coaches at the YMCA will receive their PARQ, Goal Attainment Scale (GAS) as well as phone number and/or e-mail address. The Coaches will use the PARQ and GAS to help inform the program that they develop for participants and use phone number and e-mail to contact the participant to schedule weekly fitness appointments and follow up.

At the end of the Initial CBE Screening Meeting, interested and eligible individuals will be informed that they are able to sign the consent form at that time or take more time in deciding about their participation and schedule another meeting (face-to-face or via telephone) with the Research Coordinator to indicate consent at a later time. Individuals who decide to participate will be asked to sign a written informed consent. Participants can e-mail the Research Coordinator their signed consent form if and whenever ready. Participants will be asked to sign two copies of the consent form, one will be for the researchers and the other will be given to the participant.

We may send up to three reminders to potential participants to set up the Screening Meeting. Participants who indicate that they are not interested or do not respond after these three requests, will not be further approached about participation.

Qualitative Interviews

For the qualitative interviews with PHA participants and recreation providers, we will make initial contact with a subsample of PHA participants and recreation providers via e-mail (Appendix DD), or by telephone (depending on their stated preferred method). We will provide participants and recreation providers with a 'Qualitative Interview' Information Letter and Consent Form (Appendix EE and FF) and indicate they are able to sign the consent form at that time or take more time in deciding about their participation in these interviews and schedule another meeting (face-to-face or via telephone) with the Research Coordinator to indicate consent at a later time. Individuals who agree to participate in the interviews will be asked to sign the Information Letter and Consent Form. Participants can e-mail the Research Coordinator their signed consent form if and whenever they are ready. Participants will be asked to sign two copies of the consent form, one will be for the researchers and the other will be given to the participant. We may send up to one reminder e-mail to participants who do not respond to the initial e-mail.

Contact with Participants for Future Phases of Study

We will ask participants if they are willing to be contacted for future phases of this study. This will occur during the final Self-Reported Questionnaire Assessment (for participants who remain in the study), or via email (for participants who withdraw prior to study completion or for those participants who do not attend the final assessment). We will provide participants with a 'Future Research Phase Contact' Information Letter and Consent Form (Appendix GG) and indicate they are able to sign the consent form at that time or take more time in deciding about their future participation and schedule another meeting (face-to-face or via telephone) with the Research Coordinator to indicate consent at a later time. Individuals who agree to be contacted regarding future phases of the study will be asked to sign a written informed consent and provide their preferred method of contact (email and/or telephone). Participants can e-mail the Research Coordinator their

signed consent form if and whenever they are ready. Participants will be asked to sign two copies of the consent form, one will be for the researchers and the other will be given to the participant.

For participants who have already withdrawn from the study or for those who do not attend their final assessment, the Research Coordinator will send them an e-mail (Appendix HH) asking if they would be willing to be contacted for future phases of the study. This e-mail will include the ‘agree to be contacted form’ (Appendix GG) as an attachment. We will let participants know that if they are willing to be contacted, they can either scan the signed consent form and send it to the Research Coordinator or schedule a face-to-face meeting with the Research Coordinator. We will include the phone number and email contact of the Research Coordinator in the email if any questions arise.

20 CONSENT DOCUMENTS

(a) **Attach a copy of the Information Letter/Consent Form.**
For details about the required elements in the information letter and consent form, please refer to our informed consent guide (**http://www.research.utoronto.ca/wp-content/uploads/2010/01/GUIDE-FOR-INFORMED-CONSENT-April-2010.pdf**)

Additional documentation regarding consent should be provided such as:

- **screening materials introductory letters, letters of administrative consent or authorization**

(b) If any of the information collected in the screening process - prior to full informed consent to participate in the study - is to be retained from those who are later excluded or refuse to participate in the study, please state how potential participants will be informed of this course of action and whether they will have the right to refuse to allow this information to be kept.

See Appendix BB for the Information Letter and Consent Form.

If participants withdraw from the study, the information that the researchers receive up to the point of withdrawal may be used for data analysis regardless of its completeness, unless the participant wishes to have it removed (as stated in the attached consent form). If participants decide to withdraw from the study we will ask them to complete the Phase One/Two/Three Completion/Withdrawal Questionnaire (depending on which month they withdraw from the study) to obtain final information on reasons for withdrawal and perceived changes in health status (they will have the option to decline to complete the questionnaires).

See Appendix EE and FF for the Information Letter and Agreement Form for the Qualitative Interviews.

See Appendix GG for the Information Letter and Agreement Form to be Contacted About Future Phases of this Study.

21. COMMUNITY AND/OR ORGANIZATIONAL CONSENT, OR CONSENT BY AN AUTHORIZED PARTY

(a) If the research is taking place within a community or an organization which requires that formal consent be sought prior to the involvement of individual participants, describe how consent will be obtained and attach any relevant documentation. If consent will not be sought, please provide a justification and describe any alternative forms of consultation that may take place.

The community organizations (YMCA, Casey House, Toronto PWA) are involved with this study as collaborators and support the following consent process as reviewed by the University of Toronto REB.

(b) If any or all of the participants are children and/or others who are not competent to consent, describe the process by which capacity/competency will be assessed, and the proposed alternate source of consent.

i) Submit a copy of the permission/information letter to be provided to the person(s) providing the alternative consent

ii) Describe the assent process for participants and attach the assent letter.

NA

22. DEBRIEFING and DISSEMINATION

(a) If deception or intentional non-disclosure will be used in the study, provide justification. Please consult the Guidelines for the Use of Deception and Debriefing in Research

NA

(b) Please provide a copy of the written debriefing form, if applicable.

NA

(c) If participants and/or communities will be given the option of withdrawing their data following the debriefing, please describe this process.

NA

(d) Please describe what information/feedback will be provided to participants and/or communities after their participation in the project is complete (e.g., report, poster presentation, pamphlet, etc.) and note how participants will be able to access this information.

Knowledge will be translated among PHAs, researchers, future and current health providers, government, community-based agencies, employment representatives, and policy-makers. Integrated KT with PHAs will be led by the Knowledge Users in a series of ‘HIV CBE Community Meetings’ and facilitated by Collaborator organizations. We will devise a quarterly newsletter summarizing updates from the study with key messages that will be made available on ASO Collaborator and CWGHR websites, helping organizations learn from the CBR process and use the research findings to inform future programming and services. Knowledge translation to academic and clinical audiences will occur among Researchers, Knowledge Users and Collaborators through publications in open access peer-reviewed scientific journals, podium and poster presentations at hospital rounds and scientific meetings, skills building symposia and lectures in health professional and UWW curricula. We will apply for a Café Scientifique to translate findings with community. We will also work with the *Learning Place for Community-Based Research in HIV/AIDS* (108) to establish an electronic case study reflecting on our experience partnering with the HIV and broader health community in this research. Further end-of-grant KT will occur through collaborations with national professional organizations including the Canada-UK HIV and Rehabilitation Research Collaborative (CUHRRC) (109) and the provincial Ministries of Health. Integration of this research will occur via CWGHR’s online courses, mentorship programs, Access to Rehabilitation Committee, and the openly accessible *E-Module for Evidence-Informed HIV Rehabilitation* (110). The Canadian AIDS Treatment Information Exchange can use results when updating the “Managing Your Health” guide for PHAs (111).

Further modes of dissemination will include podium/poster presentations, lectures in health professional and Universities Without Walls (UWW) curricula, presentations at ASOs, skills building symposia, and

newsletters. Further translation may occur through collaborations with CWGHR, the Canada-United Kingdom HIV and Rehabilitation Research Collaborative (CUHRRC), and YMCA centres across Canada to inform broader program and policy development for PHAs.

Ultimately, results will lead to the first known HIV-specific CBE intervention in Canada evaluated for effectiveness and successful translation within the HIV community.

References

108. Ontario HIV Treatment Network. The Learning Place for CBR in HIV/AIDS. 2014; http://tlp.ohtn.on.ca/.
109. Canada-United Kingdom HIV and Rehabilitation Research Collaborative (CUHRRC). 2014; http://cuhrrc.hivandrehab.ca/. Accessed May 19, 2014, 2014.
110. Canadian Working Group on HIV and Rehabilitation. E-Module for Evidence-Informed HIV Rehabilitation (e-module). 2014.
111. Canadian AIDS Treatment Information Exchange. Managing Your Health: 4th Edition. 2009.

23. PARTICIPANT WITHDRAWAL

(a) Where applicable, please describe how participants will be informed of their right to withdraw from the project and outline the procedures that will be followed to allow them to exercise this right.

During the ‘Initial CBE Study Screening Meeting,’ the Research Coordinator and interested individuals will discuss the details associated with the study. During this meeting, the interested individual will discuss their eligibility and review the details of the study with the Research Coordinator including the detail in the information letter and consent form (Appendix BB). On the information sheet and consent form, details about participants’ rights to withdraw are indicated.

Potential participants will be informed that participation in the study is voluntary and that they have the right to withdraw from the study at any point in time for any reason with no negative consequences. For the adults living with HIV who are members of the YMCA, Casey House, CWGHR or Toronto PWA, withdrawal will not affect the services that they receive at the YMCA, Casey House, CWGHR or Toronto PWA. If participants withdraw, the information that we receive up to the point of withdrawal from the study may be used for data analysis regardless of its completeness unless the participant wishes to have it removed. Participants also have the right to refrain from answering questions that they do not feel comfortable with as well as not participate in any physical aspect of the intervention or any assessments that they are not comfortable with.

To ensure retention in this study, we will contact participants a certain amount of times before considering them ‘lost to follow-up.’ We will attempt to contact participants three times over a period of two weeks. If we have not heard from them after 3 weeks, we will attempt to contact participants two times again. After four weeks, if we have not heard from the participant, we will contact them once again before considering them ‘lost to follow-up.’

(b) Indicate what will be done with the participant’s data and any consequences which withdrawal may have on the participant.

Participants have the right to withdraw from the study at any point in time for any reason with no negative consequences. For the adults living with HIV who are members of the YMCA, CWGHR, Casey House or Toronto PWA, withdrawal will not affect the services that they received at these sites. If participants withdraw, the information that we receive up to the point of withdrawal from the study may be used for data analysis regardless of its completeness unless the participant wishes to have it removed. Participants also have the

right to refrain from answering questions that they do not feel comfortable with as well as not participate in any physical aspect of the intervention or assessment that they are not comfortable with.

If an individual decides not to participate or is ineligible to participate, we will remove all data related to their personal identity (name, address, contact information) and destroy their PAR-Q questionnaire. However, we are interested in retaining information in general on the number of individuals who express interest in the study, and the number who are deemed ineligible (and reason if available) and the number who decide not to participate (with reason if applicable).

(c) If participants will not have the right to withdraw from the project at all, or beyond a certain point, please explain. Ensure this information is included in the consent process and consent form.

NA

SECTION E – CONFIDENTIALITY AND PRIVACY

24. CONFIDENTIALITY

Data security measures must be consistent with UT's *Data Security Standards for Personally Identifiable and Other Confidential Data in Research*. All identifiable electronic data that is being kept outside of a secure server environment must be encrypted, consistent with the standards described at: *http://www.utoronto.ca/security/UTORprotect/encryption_guidelines.htm:*

(a) Will the data be treated as confidential? Yes ☒ No ☐

(b) Describe the procedures to be used to protect the confidentiality of participants or informants, where applicable

Name, telephone and/or email address will be collected depending on participants preferred mode of communication. Email addresses will be collected for mailing the study information sheet and consent form. All information will remain strictly confidential and available only to study investigators, research staff, and the University of Toronto Research Ethics Board that reviewed this protocol, and other regulatory authorities for the purpose of monitoring this study, unless required by law. Participants will be matched with a fitness instructor at the YMCA and they will have access to participants' preferred mode of contact information collected at the intake questionnaire in order to be able to directly liaise with their respective participants about scheduling their fitness sessions.

Participants will be provided with the telephone number of Kelly O'Brien's Lab at UofT, where they will either speak to the research coordinator or leave a voicemail on the password-protected voicemail.

Participants' names and any other personal identifiers will be removed from all data associated with the assessments and a numerical code that will be assigned to the participant throughout the coursed of the study.

All data from the online exercise log will be held at the University of Toronto according to the institutional privacy protocols (e.g. in a locked cabinet inside a secured office; on a password protected computer).

(c) Describe any limitations to protecting the confidentiality of participants whether due to the law, the methods used, or other reasons (e.g., a duty to report)

NA

25. DATA SECURITY, RETENTION AND ACCESS

(a) Describe how data (including written records, video/audio recordings, artifacts and questionnaires) will be protected during the conduct of the research and dissemination of results.

Data related to this study will be held at the University of Toronto according to the institutional privacy protocols (e.g. in a locked cabinet or on a password protected computer inside a secured office). Members of the research team and Kelly O’Brien’s research coordinator will have access to the study data. Data collected at the YMCA will be stored on an encrypted USB key and subsequently transferred to the University of Toronto by the Research Coordinator. Data specific to neurocognitive outcomes will be transferred to St. Michael’s Hospital to Dr. Sean Rourke for analysis. We will pursue a Data Transfer and Privacy Agreement between SMH and University of Toronto similar to that obtained during the pilot phase of the study.

Computer files will be kept in a password-protected folder on the UofT server. The researchers will use a password-protected and encrypted ShareFile account to transfer and store electronic data. Any hard copy documents including questionnaires and consent forms will all be stored in a locked filing cabinet in the lab of Kelly O’Brien at the Rehabilitation Sciences Building at the University of Toronto. The document that will link the name of each participant to their assigned numeric code will be kept in on a password protected file on the UofT server accessible to the Coordinator and Kelly O’Brien.

The researchers will provide the Central Toronto YMCA with a hard copy of the document linking the name of the participant to their assigned numeric code to allow for the designated Coach to upload the documents (Coach Log, GAS) to ShareFile. The researchers will also provide the Central Toronto YMCA with a hard copy of the document with participant names, e-mail address and/or phone numbers to facilitate program development and contact between Coaches and participants.

Any printed and electronic data will be retained for 10 years in the same location that it was stored throughout the study following which they will be destroyed by the Principal Investigator.

All responses on the exercise log questionnaire entered will be downloaded and stored on a secure server at the University of Toronto. Electronic data will be stored on a password protected computer in a locked office at the University of Toronto, accessible only to the investigators and research coordinator. Files related to this study will be stored and shared among investigators on a secured and encrypted file share system, ShareFile (http://www.sharefile.com/about/). Computerized data will be backed up regularly. Electronic data will be destroyed 10 years after publication from this work.

Notes taken during the interviews will be stored in a locked filing cabinet in the office of the primary investigator at the Rehabilitation Sciences Building at the University of Toronto. No identifying information will be included when compiling and disseminating results. In the event that we want to include specific quotes, pseudonyms will be used.

Finally, as regular YMCA practice, the YMCA collects personal and emergency contact information from all of its members (including name, birthday, allergies or health concerns, gender, address, phone number, email address, emergency contact name and phone number). This information will be collected and stored at the Central Toronto YMCA as part of their general membership process, but is not part of the research study. The YMCA does not provide the information of their members to others. However they do communicate with members regarding YMCA promotions, or newsletters. All communication with YMCA members is within the new Canadian Anti-Spam Legislation (CASL).

(b) Explain how long data will be retained. (If applicable, referring to the standard data retention practice for your discipline) Provide details of their final disposal or storage. Provide a justification if you intend to store your data for an indefinite length of time. If the data may have archival value, discuss how participants will be informed of this possibility during the consent process.

Following the completion of the study, printed and electronic data will be retained for 10 years in the same location that it was stored throughout the study. Any personal information obtained on the CBE Intake Questionnaire will be destroyed after study completion. The Principal Investigator will be responsible for shredding the printed documents and deleting the electronic data.

(c) If participant anonymity or confidentiality is not appropriate to this research project, please explain.

NA

(d) If data will be shared with other researchers or users, please describe how and where the data will be stored and any restrictions that will be made regarding access.

NA

SECTION F – LEVEL OF RISK AND REVIEW TYPE

See the *Instructions for Ethics Review Protocol Submission Form* for detailed information about the Risk Matrix.

26. RISK MATRIX: REVIEW TYPE BY GROUP VULNERABILITY AND RESEARCH RISK

(a) Indicate the Risk Level for this project by checking the intersecting box

	Research Risk		
**Group Vulnerability**	**Low**	**Medium**	**High**
**Low**	**1** ☐	**1** ☐	**2** ☐
**Medium**	**1** ☐	**2** ☐	**3** ☐
**High**	**2** ☐	**3** ☒	**3** ☐

(b) Explain/justify the level of research risk and group vulnerability reported above:

Group vulnerability is considered high because participants will be adults living with HIV who may or may not have multiple comorbidities. Due to possible physical risk associated with participation paired with measures taken by the team to minimize physical/emotional/social risk, the Research Risk is assessed to be Medium.
