# Supplementary material for: Examining the impact of a community-based exercise intervention on cardiorespiratory fitness, cardiovascular health, strength, flexibility and physical activity among adults living with HIV: A three-phased intervention study
Source: PLoS One. 2021 Sep 24;16(9):e0257639. doi: 10.1371/journal.pone.0257639 (PMC8462727; doi:10.1371/journal.pone.0257639)
Supplement: Attachment — Submitted filename: Reviewer 1 feedback on HIV and CBE revised manuscript.pdf [file pone.0257639.s012.pdf]

PONE-D-21-15069R1
Reviewer 1 Comments on Revised Manuscript

To the PLOS One Editorial Team:

I have two pieces of feedback to the journal:

1. **When you provide a pdf of the track changes revision, the new line numbers referred to by the authors don't correspond to the line numbers in the track changes red line copy. So, what I have already spent hours on, takes even more time.**

2. **I was literally speechless when I saw the one paragraph "Reviewer 3" comments, stating that the manuscript was "perfect." They did not respect the authors enough to read and thoughtfully comment on their study. Or the other reviewers. Or the journal – it makes a complete mockery of the PLOS review process and I can only hope that it was some kind of computer error that you did not reject these alleged comments.**

3. **I am recommending the authors incorporate of some of their comments to me into the actual revision – see below. Otherwise I recommend the revised manuscript be published.**

To Dr. O'Brien and Co-Authors:

I think the revised manuscript is much stronger and would ask you only to incorporate some of your explanations to me below, into your actual revised manuscript. When I asked for clarifications, it was not so I could personally understand your meaning better, but so you could enlighten your readership on the point in question by adding this information to your revision.

So, if you could insert the following passages highlighted in yellow into an appropriate location (typically Methods or Limitations) in the manuscript, I think it would be stronger and more readable.

Reviewer #1:
4. I could not find whether participants were compensated for this? Did I miss that? If not, I wonder if it would have helped participation and statistical significance? It is a big study and I am surprised that compensation was not built in.

Response: Participants were not compensated for their engagement in the CBE intervention as this would have introduced a co-intervention with incentive to exercise. Participants received weekly personal training / supervision during Phase 2 (intervention) as well as a YMCA membership providing access to the YMCA throughout Phase 2 and Phase 3. Details of

receiving a YMCA membership and access to the fitness coach is included in the description of the Intervention. (Line 172-174; Line 197-198).

7. *Lines 127-128.*
I would like to see a short explanation of why you chose your secondary measures, You could have gone in a lot of different directions on this.

Response: Our study included primary and secondary outcomes of interest measured by objective and self-reported questionnaire assessments. This study reports on the primary outcome of interest (Vo2peak) and self-reported and objective physical health assessments related to the primary outcome of interest. The secondary outcomes focused on objective measures of cardiopulmonary fitness, strength, and flexibility, as they are related to the primary outcome of interest, Vo2peak, and commonly reported in previous systematic reviews on HIV and exercise. (Line 207-209)

8. *Lines 129. An explanation of why you chose the time periods of 8 mos, 6 mos and 8 mos. Would be helpful*
Response: We chose a 6-month CBE duration because it aligns with transtheoretical model evidence on the stages of behaviour change whereby the 'action' of practicing a new behaviour (exercise) lasts up to 24 weeks followed by the 'maintenance' stage during which commitment to sustaining the new behavior (self-monitored exercise) is solidified (Prochaska, 1997). The 8 month baseline and 8 month follow ensured equal timeframes prior to and after the intervention, ensured an equal number of data points in each phase (Baseline: T1-T4; Intervention: T5-T8; Follow-Up: T9-12). We include these details in our protocol referenced in the manuscript.

10. *-Lines 226 to 235 Please provide a rationale for the design of this Exercise Dose questionnaire. Was it adapted from an established validated instrument? Similarly the demographics questionnaire*
Response: Our questions about dosage in the demographic questionnaire were guided by the Canadian Physical Activity Guidelines (CPAG), which uses number of minutes engaged in moderate to vigorous aerobic physical activity in the past week as a unit of measurement. This was not from an existing validated instrument. The demographic questionnaire also was not a previous validated instrument. As a team, we identified constructs important for personal, health-related and exercise-related characteristics. We piloted the questionnaire prior to implementation with members of the team.

17. Limitations: Wasn't your internal validity affected by the wide variation of the types of exercise participants engaged in? IE group vs individual, spinning vs yoga. Did you control statistically for this?
Response: We did not control for different variations of community-based exercise in the study. This was not possible or desirable for the intent of our study. Our intent was to implement a

community-based exercise program that included a combination of aerobic, resistive, flexibility and balance exercise. We adopted a ‘guided adaptation’ approach whereby the intervention was adapted based on the ongoing assessment of the needs and ability of participants by the fitness coach (Line 629-632)
