# Supplementary material for: Examining the impact of a community-based exercise intervention on cardiorespiratory fitness, cardiovascular health, strength, flexibility and physical activity among adults living with HIV: A three-phased intervention study
Source: PLoS One. 2021 Sep 24;16(9):e0257639. doi: 10.1371/journal.pone.0257639 (PMC8462727; doi:10.1371/journal.pone.0257639)
Supplement: Attachment — Submitted filename: Response-to-Reviews-CBE-Vo2peak-SUBMITTED-Aug-29-21.pdf [file pone.0257639.s013.pdf]

Physical Therapy
UNIVERSITY OF TORONTO

Walid Kamal Abdelbasset, PhD
Academic Editor
PLOS ONE

August 28, 2021

PONE-D-21-15069R1 - Examining the impact of a community-based exercise intervention on cardiorespiratory fitness, cardiovascular health, strength, flexibility and physical activity among adults living with HIV: a three-phased intervention study

Dear Dr. Abdelbasset,

Thank you for the additional review of our manuscript. We uploaded a revised version of the manuscript with revisions highlighted in yellow along with an unmarked version of the revised manuscript. Our point-by-point response to the comments is below.

Declaration of Conflicting Interests: The author(s) declared no potential conflicts of interest with respect to the research, authorship, and/or publication of this article.

Funding: The author(s) disclosed receipt of the following financial support for the research, authorship, and/or publication of this article: This research is funded by the Canadian Institutes of Health Research (CIHR) HIV/AIDS Community-Based Research Initiative CIHR Funding Reference Number: 139685. Kelly K. O'Brien is supported by a Canada Research Chair in Episodic Disability and Rehabilitation from the Canada Research Chairs Program.

Please do not hesitate to contact me if you require any further information. Thank you for considering our manuscript for publication in *PLOS ONE.*

Sincerely,

Kelly O'Brien, PhD, PT
Associate Professor
Canada Research Chair in Episodic Disability and Rehabilitation
Department of Physical Therapy
500 University Avenue, Room 160
University of Toronto
kelly.obrien@utoronto.ca

Journal Requirements:

1. *Please review your reference list to ensure that it is complete and correct. If you have cited papers that have been retracted, please include the rationale for doing so in the manuscript text, or remove these references and replace them with relevant current references. Any changes to the reference list should be mentioned in the rebuttal letter that accompanies your revised manuscript. If you need to cite a retracted article, indicate the article's retracted status in the References list and also include a citation and full reference for the retraction notice.*

Response: Thank you. We reviewed all references and revised the reference list. There were two citations that reflected older citations (e.g. in press) with our referencing software – these have now been revised to reflect the current and accurate citations. In addition, we added reference #39 and #48 to reflect the requested additions from Reviewer 1 below.

We uploaded new figures after going through the PACE software. We revised the TREND checklist accordance to the new page numbers of the new revised (clean) manuscript.

Response:

Reviewer 1 Comments

Please see my attached comments. I have feedback for both the PLOS One editorial team and minor changes for the manuscript authors.

1. *I think the revised manuscript is much stronger and would ask you only to incorporate some of your explanations to me below, into your actual revised manuscript. When I asked for clarifications, it was not so I could personally understand your meaning better, but so you could enlighten your readership on the point in question by adding this information to your revision. So, if you could insert the following passages highlighted in yellow into an appropriate location (typically Methods or Limitations) in the manuscript, I think it would be stronger and more readable.*

Previous Review Comment 4. I could not find whether participants were compensated for this? Did I miss that? If not, I wonder if it would have helped participation and statistical significance? It is a big study and I am surprised that compensation was not built in.

Initial Response: *Participants were not compensated for their engagement in the CBE intervention as this would have introduced a co-intervention with incentive to exercise. Participants received weekly personal training / supervision during Phase 2 (intervention) as well as a YMCA membership providing access to the YMCA throughout Phase 2 and Phase 3. Details of receiving a YMCA membership and access to the fitness coach is included in the description of the Intervention. (Line 172-174; Line 197-198).*

Response: Details of what was involved in the intervention (provision of YMCA membership and weekly personal training) are included in the methods of the manuscript (Page 8 and 9). We added details of our rationale pertaining to compensation in the Discussion section (Page 30).

Previous Comment 7. Lines 127-128. I would like to see a short explanation of why you chose your secondary measures, You could have gone in a lot of different directions on this.

Initial Response: *Our study included primary and secondary outcomes of interest measured by objective and self-reported questionnaire assessments. This study reports on the primary outcome of interest (Vo2peak) and self-reported and objective physical health assessments related to the primary outcome of interest. The secondary outcomes focused on objective measures of cardiopulmonary fitness, strength, and flexibility, as they are related to the primary outcome of interest, Vo2peak, and commonly reported in previous systematic reviews on HIV and exercise. (Line 207-209).*

Response: The description of primary and secondary outcomes is indicated in the Methods of the manuscript. For instance, the following is included at the beginning of the Materials and Methods section, "Our primary outcome was cardiorespiratory fitness (peak oxygen consumption measured by $\dot{V}O_2$peak). Maximum or peak oxygen consumption ($\dot{V}O_2$peak) is a direct measure of cardiorespiratory fitness." and "Secondary outcomes included resting heart rate (beats/min), blood pressure (systolic and diastolic; mmHg), strength (upper and lower extremity muscle strength and endurance), flexibility (hamstrings) and self-reported physical activity. These secondary outcomes were chosen because they are related to the primary outcome of interest, $\dot{V}$O2peak, and commonly reported in previous systematic reviews on HIV and exercise." The following is also included in the Data Collection section: "We examined the primary outcome of interest ($\dot{V}O_2$peak) and secondary outcomes related to the primary outcome of interest as measured by objective and self-reported physical health assessments." (Page 6 and 9).

Previous Comment 8. Lines 129. An explanation of why you chose the time periods of 8 mos, 6 mos and 8 mos. Would be helpful

Initial Response: *We chose a 6-month CBE duration because it aligns with transtheoretical model evidence on the stages of behaviour change whereby the 'action' of practicing a new behaviour (exercise) lasts up to 24 weeks followed by the 'maintenance' stage during which commitment to sustaining the new behavior (self-monitored exercise) is solidified (Prochaska, 1997). The 8 month baseline and 8 month follow ensured equal timeframes prior to and after the intervention, ensured an equal number of data points in each phase (Baseline: T1-T4; Intervention: T5-T8; Follow-Up: T9-12). We include these details in our protocol referenced in the manuscript.*

Response: We add the following to the Intervention Section: "We chose a 6-month CBE duration because it aligns with transtheoretical model evidence on the stages of behaviour change whereby the 'action' of practicing a new behaviour (exercise) lasts up to 24 weeks followed by the 'maintenance' stage during which to solidify a commitment to sustaining the new behavior (self-monitored exercise)." (Page 8). We added the following to the Materials and Methods Section: "The 8 month baseline and 8 month follow-up ensured equal timeframes prior to and after the intervention, ensured an equal number of data points in each phase (Baseline: T1-T4; Intervention: T5-T8; Follow-Up: T9-12)." (Page 7)

Previous Comment 10. -Lines 226 to 235 Please provide a rationale for the design of this Exercise Dose questionnaire. Was it adapted from an established validated instrument? Similarly the demographics questionnaire

Initial Response: *Our questions about dosage in the demographic questionnaire were guided by the Canadian Physical Activity Guidelines (CPAG), which uses number of minutes engaged in moderate to vigorous aerobic physical activity in the past week as a unit of measurement. This was not from an existing validated instrument. The demographic questionnaire also was not a previous validated instrument. As a team, we identified constructs important for personal, health-related and exercise-related characteristics. We piloted the questionnaire prior to implementation with members of the team.*

Response: We added the above details to the Exercise Dose description: "We developed the weekly exercise, and demographic and health questionnaires as a team to include items that comprised personal, health and exercise-related characteristics. We piloted the weekly exercise questionnaire and demographic and health questionnaire prior to implementation with members of the team." (Page 12).

Previous Comment 17. Limitations: Wasn't your internal validity affected by the wide variation of the types of exercise participants engaged in? IE group vs individual, spinning vs yoga. Did you control statistically for this?

Initial Response: *We did not control for different variations of community-based exercise in the study. This was not possible or desirable for the intent of our study. Our intent was to implement a community-based exercise program that included a combination of aerobic, resistive, flexibility and balance exercise. We adopted a 'guided adaptation' approach whereby the intervention was adapted based on the ongoing assessment of the needs and ability of participants by the fitness coach (Line 629-632)*

Response: We do not consider our lack of control group as a limitation of our study, as this was not our intent given the implementation science study design. We address this in the Discussion –"Our study did not include a comparison group; instead we considered the Phase 1 baseline as a control or monitoring phase of the study from which to estimate an effect." (Page 29) and later on when we state: "In our study, we adopted a 'guided adaptation' approach whereby the intervention was tailored to the ongoing assessment of the needs and ability of participants by the fitness coach…. This aligned with our implementation science approach, to evaluate the impact of the intervention in the 'real world' setting." (Page 30)

Reviewer #3 Comments

The authors completed all the work for this paper, and it is worth publishing. The methods utilized here was appropriate, the results were correct, and the conclusion they finally gain was reliable. At last but at least, they reported that the effect of exercise intervention on HIV therapy was effective, which enlighted patients with hopeful treatment strategy. And again, as they mentioned in the ending, featured exercise therapeutic regimen needed to be elucidated. Based on its topic and the perfect work they have done, I strongly recommend this paper to be published in PLOS ONE.

Response: Thank you
